# Dithiolane quartets: thiol-mediated uptake enables cytosolic delivery in deep tissue†

**DOI:** 10.1039/d1sc04828g

**Published:** 2021-10-06

**Authors:** Rémi Martinent, Salman Tawffik, Javier López-Andarias, Dimitri Moreau, Quentin Laurent, Stefan Matile

**Affiliations:** Department of Organic Chemistry, University of Geneva Geneva Switzerland stefan.matile@unige.ch https://www.unige.ch/sciences/chiorg/matile/ +41 22 379 6523

## Abstract

The cytosolic delivery of various substrates in 3D multicellular spheroids by thiol-mediated uptake is reported. This is important because most orthodox systems, including polycationic cell-penetrating peptides, fail to deliver efficiently into deep tissue. The grand principles of supramolecular chemistry, that is the pH dependence of dynamic covalent disulfide exchange with known thiols on the transferrin receptor, are proposed to account for transcytosis into deep tissue, while the known but elusive exchange cascades along the same or other partners assure cytosolic delivery in kinetic competition. For quantitative detection in the cytosol, the 2D chloroalkane penetration assay (CAPA) is translated to 3D deep tissue. The targeted delivery of quantum dots, otherwise already troublesome in 2D culture, and the controlled release of mechanophores are realized to exemplify the power of thiol-mediated uptake into spheroids. As transporters, dithiolane quartets on streptavidin templates are introduced as modular motifs. Built from two amino acids only, the varied stereochemistry and peptide sequence are shown to cover maximal functional space with minimal structural change, *i.e.*, constitutional isomers. Reviving a classic in peptide chemistry, this templated assembly of β quartets promises to expand streptavidin biotechnology in new directions, while the discovery of general cytosolic delivery in deep tissue as an intrinsic advantage further enhances the significance and usefulness of thiol-mediated uptake.

## Abstract 2

Cytosolic delivery in multicellular 3D spheroids is shown to be an intrinsic advantage of thiol-mediated uptake, which is compatible with proteins and QDs, achieving targeting and controlled release.

## Graphical abstract

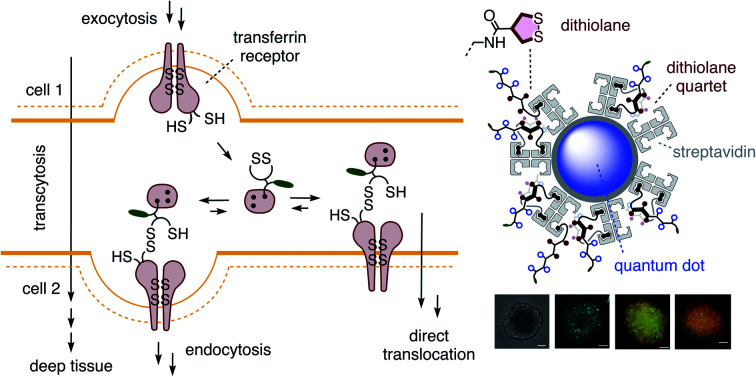

## Introduction

**Fig. 1:**
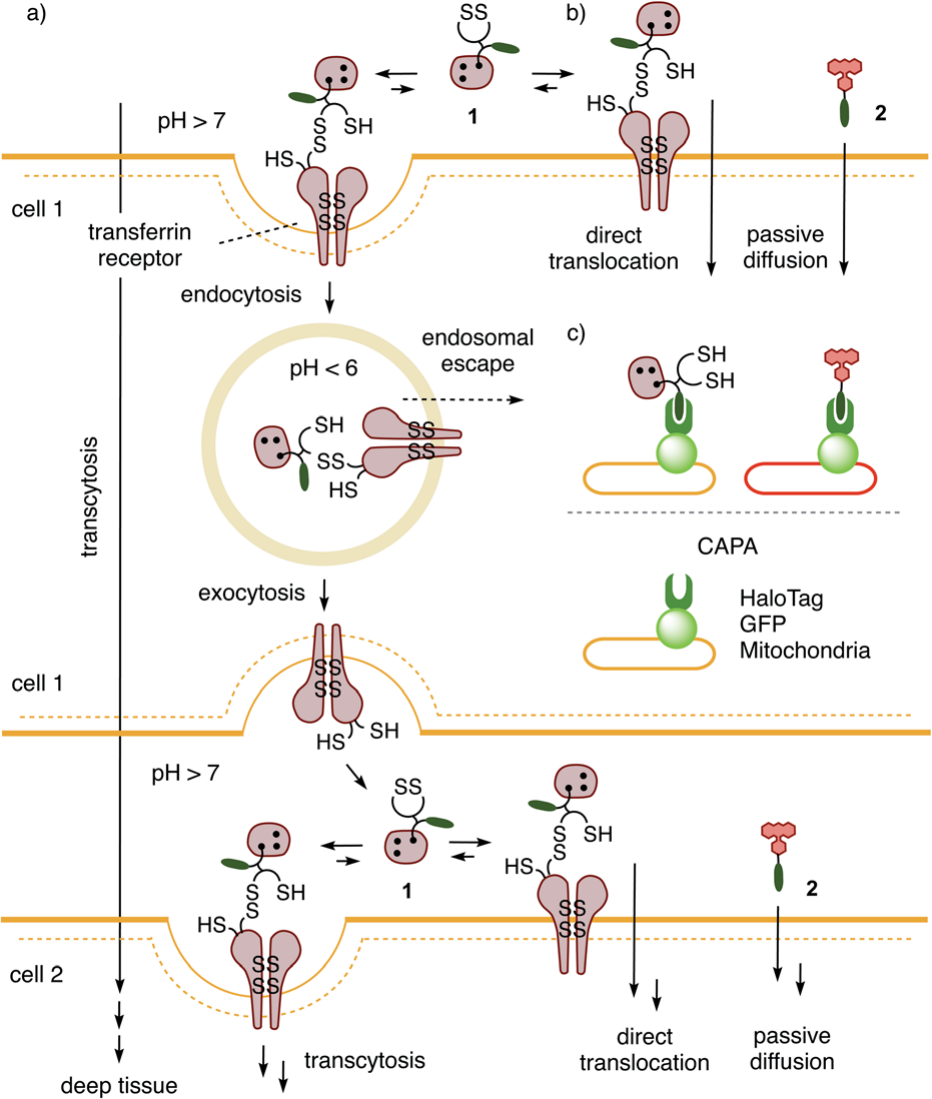
The proposed mechanism of (a) thiol-mediated transcytosis in kinetic competition with (b) thiol-mediated uptake by direct translocation of transporter **1** into HGM spheroids for (c) the quantitative detection of cytosolic delivery in deep tissue by labeling all unoccupied HaloTags with fluorescent reporter **2**.

The general delivery of substrates into the cytosol of cells is a persistent challenge in life sciences and calls for input from chemistry. A rich and diverse collection of transporters for delivery in 2D cell culture exists. Their general usefulness is difficult to judge because their activities often depend strongly on conditions (substrates, cells, localization, degradation, *etc.*), detection methods, and limited synthetic availability. What many existing transporters have in common is a poor ability to penetrate deep tissue.^1–13^ Besides some remarkable exceptions,^14–20^ this includes also the popular cell-penetrating peptides (CPPs).^7,8^ Access to deep tissue is challenging because it requires a balanced combination of transcytosis and release into the cytosol. Transcytosis is best known from the transferrin receptor (TfR), which enters cells by endocytosis and returns to the surface by exocytosis (Fig. 1a).^2,21–27^ This mechanism allows the TfR to efficiently penetrate tumors and cross the blood–brain barrier to deliver iron to the brain. The attachment of transferrin or ligands of other transcytosis receptors to operational transport systems has been shown to improve delivery into deep tissue.^2,21–27^

**Fig. 2:**
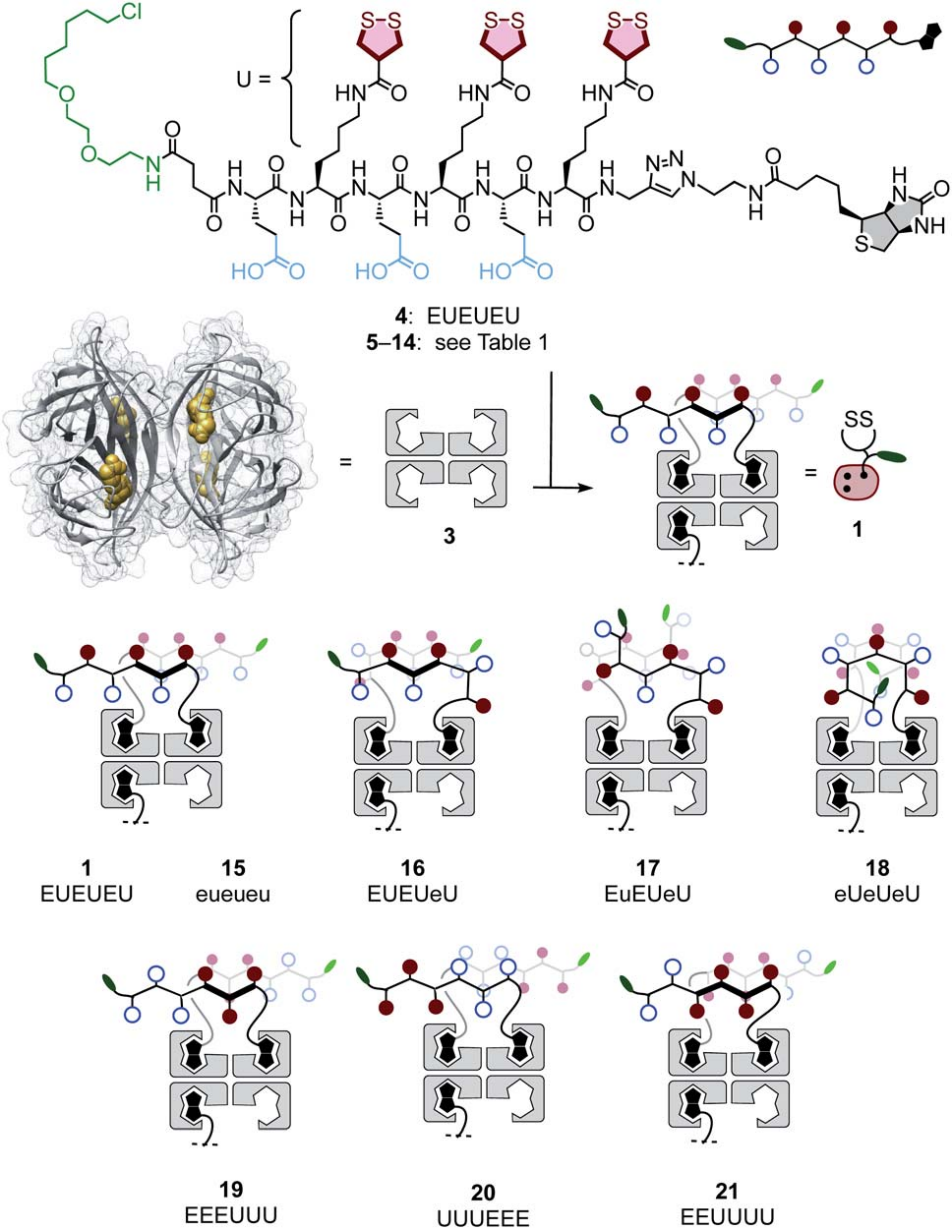
General structure of peptides **4–14** and complexes **1** and **15–21** with the schematic of possible peptide conformations. E: l-glutamate, e: d-glutamate, U: l-lysine–AspA conjugate, u: d-lysine–AspA conjugate.

Evidence is emerging that the TfR also participates in thiol-mediated uptake^28–32^ and viral entry,^28,33,34^ including oligonucleotide phosphorothioates^35^ and SARS-CoV-2,^28,34^ respectively (Fig. 1b). It was thus conceivable that thiol-mediated uptake intrinsically assures cytosolic delivery into deep tissue. Thiol-mediated uptake^28–39^ operates with transporters that are equipped with thiol-reactive motifs, such as poly- or oligochalcogenides, often disulfides, at best cyclic ones (COCs), for dynamic covalent exchange^40–49^ with cellular thiols. Dynamic covalent disulfide exchange of transporters such as **1** (ref. 50) with thiols C556 and C558 of the TfR and tether the transporters through at least one disulfide bond to the membrane protein.^29^ Then, direct translocation occurs by thiolate-disulfide exchange cascades along the TfR or other protein partners to deliver COCs like **1** into the cytosol (Fig. 1b). Coinciding TfR endocytosis brings the tethered COCs like **1** into endosomes. The punctate patterns characteristic of endosomes have been identified during COC uptake and co-localization with fluorescently-labeled TfRs.^29,51^ Unless assisted by enzymes,^52^ release from endosomes is less favorable because low pH^53^ inactivates dynamic covalent exchange. It was thus conceivable that transporters like **1** could return to the surface of the first cell, where the higher pH reactivates dynamic covalent release and exchange with TfRs on the next cell to assure continuing transcytosis in kinetic competition with direct translocation. Taken together, these facts imply that thiol-mediated uptake should excel in cytosolic delivery into deep tissue. Here, we report experimental evidence that this is correct and introduce the 3D CAPA method with reporter **2** for detection (Fig. 1c) as well as protein-templated dithiolane quartets as privileged scaffolds for transport (Fig. 2).

## Results and discussion

### Design and synthesis of transporters

Transporter **1** is a native streptavidin tetramer **3** that has three binding pockets filled with COC oligomers **4** (Fig. 2).^50^ The trifunctional hexapeptide **4** is equipped with biotin at the C and a chloroalkane at the N terminus for binding to streptavidin and HaloTags, respectively. The sequence contains two amino acids only: glutamates E are placed to hinder uptake by cationic-CPP-like or other mechanisms,^54^ and to assure solubility. Uptake amino acids U are lysines coupled with COCs. AspA was selected as the COC because its activity was the highest of the series in our introductory studies, and dynamic covalent exchange with TfRs and transit through endosomes together with TfRs have been demonstrated.^29,32,50,51^ Peptides **5–14** were designed, synthesized, and then complexed with streptavidin **3** to produce a collection of transporters **15–26** that covers wide functional space with minimal structural change (Schemes S1–S10,†Fig. 2 and Table 1). The availability of such a set of isostructural COC transporters with different activities was desirable to elaborate on the intrinsic ability of thiol-mediated uptake to deliver into deep tissue (*vide infra*).

**Fig. 3:**
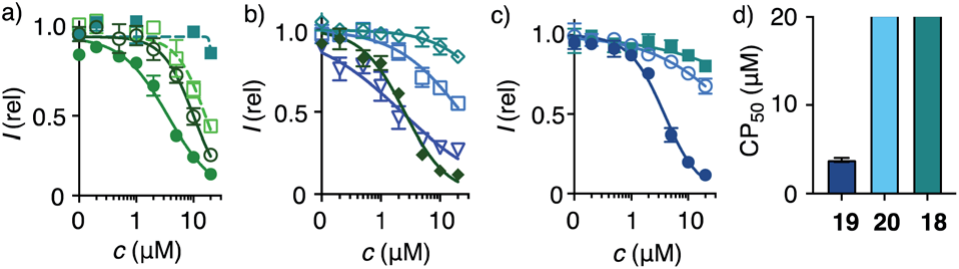
2D HC-CAPA dose–response curves for (a) **1** (filled circles), **15** (empty circles), **16** (empty squares), **17** (filled squares), (b) **21** (filled diamonds), **23** (empty diamonds), **25** (triangles), **26** (squares), (c) **18** (squares), **19** (filled circles), and **20** (empty circles), with (d) CP_50_ values for comparison with SP_50_ values (Fig. 4n).

**Table 1:** Cell-penetrating activity of transport systems^a^

	T^b^	P^c^	Sequence^d^	CP_50_^e^ (μM)	SP_50_^f^ (μM)
1	**1**	**4**	EUEUEU	3.6 ± 0.3	
2	**15**	**5**	eueueu	10.0 ± 0.6	
3	**16**	**6**	EUEUeU	16 ± 2	
4	**17**	**7**	EuEUeU	>20	
5	**18**	**8**	eUeUeU	>20	20 ± 8
6	**19**	**9**	EEEUUU	3.8 ± 0.2	5.3 ± 0.9
7	**20**	**10**	UUUEEE	>20	17 ± 6
8	**21**	**11**	EEUUUU	2.5 ± 0.2	
9	**22** g	**12**	UUUUUU	>20	
10	**23** g	**13**	EEEEEE	>20	
11	**24** g	**12** + **13**^h^		>20	
12	**25** g	**14**	KKKKKK	2.8 ± 0.3	
13	**26** g	**14** + **13**^h^		22 ± 4	

a From 2D and 3D CAPA in HGM cells.

b Transporters, composed of 1 : 3 streptavidin **3**/peptide, unless stated.

c Peptides. See Fig. 2 for general structures.

d Sequences of hexapeptide units. E: l-glutamate, U: l-*ε*-AspA–lysine, K: l-lysine. Lower-case letters represent d-isomers.

e Half maximal cell-penetration concentrations, obtained by Hill analysis of DRCs (Fig. 3).

f Half maximal spheroid-penetration concentrations, obtained by Hill analysis of DRCs (Fig. 4n).

g Streptavidin **3**/peptide 1 : 2.

h Equal equivalents of peptides were used.

### Thiol-mediated cytosolic delivery in 2D cultured cells

Cytosolic delivery into 2D cell culture was determined first using the chloroalkane penetration assay (CAPA).^55–57^ This assay has been introduced recently by Kritzer *et al.* as a reliable tool to quantitatively measure cytosolic delivery.^55–57^ The CAPA method operates with HGM cells, *i.e.* HeLa cells that stably express a fusion protein of HaloTag and GFP on mitochondria (Fig. 1c). Chloroalkylated transporters that reach the cytosol will react with these HaloTags, while all unused HaloTags will react with subsequently added chloroalkylated TAMRA **2**. The result is decreasing TAMRA fluorescence with increasing uptake of transporters. Automated high-content high-throughput imaging has been introduced to measure TAMRA fluorescence only inside of healthy cells and generate high-precision dose–response curves (DRCs, Fig. 3) while simultaneously reporting on cell viability.^50^ The uptake efficiency is reported as CP_50_ value, that is the transporter concentration needed to inhibit HaloTag labeling with **2** by 50%, determined by the Hill analysis of DRCs. In conventional 2D cell culture, complex **1** afforded CP_50_ = 3.6 ± 0.3 μM (Fig. 2, 3a, filled circles, Table 1).

With CP_50_ = 10.0 ± 0.6 μM, thiol-mediated uptake of the all-d eueueu complex **15** was only slightly worse than that of the all-l isomer **1**, suggesting that enantioselectivity and enzymatic peptide degradation contribute little to uptake (Fig. 2, 3a, empty circles, Table 1). One d amino acid in position 5 caused a similar decrease in CP_50_ = 16 ± 2 μM for EUEUeU **16** (Fig. 2, 3a, empty squares). However, all activity was lost upon two and three inversions of absolute configuration in EuEUeU **17** and eUeUeU **18** (Fig. 2, 3a and c, filled squares, Table 1). This pronounced diastereoselectivity was consistent with homochiral UXU/uxu triads as the consensus sequence. The formation of short antiparallel β sheets templated on the surface of streptavidin emerged as the most plausible active structure, producing a quartet of proximal dithiolanes with the dimensions of 5 × 7 Å (Fig. 2). Such β quartets are central to β sheet chemistry, known, for example, from the early TASP concept of Mutter, Dumy, Ulrich and coworkers,^49,58^ and from related functional motifs.^59,60^ The proximity of COCs in dithiolane β quartets was appealing with regard to the exchange cascades expected for thiol-mediated uptake.^28^

The dithiolane quartet hypothesis was in full agreement with introductory studies focusing on EU peptide length and complex stoichiometry.^50^ With regard to peptide stereochemistry covered in this study, the high activity of all-d eueueu **15** was consistent with the dithiolane quartet hypothesis and the absence of important enantioselectivity (Fig. 2). In EUEUeU **16**, the original β quartet won't form but can be replaced by an alternative β quartet, which could explain a reasonably preserved activity (Fig. 2). More stereochemical inversions in the isomers EuEUeU **17** and eUeUeU **18** formally roll peptides up into S-shaped and β helix-like pseudo-cycles (Fig. 2). These motifs block any possible β quartet formation, which was consistent with their inactivity.

Consistent with the dithiolane quartet hypothesis, EEEUUU **19** showed preserved activity (Fig. 2, 3c, filled circles). The inverse UUUEEE **20** was inactive because the repulsive tethers between the protein template and possible dithiolane quartets are too long (Fig. 2, 3c, empty circles). Beyond constitutional isomers, EEUUUU **21** was the most active of all transporters, perhaps because a second dithiolane quartet is available on the other face of the formal β sheet (Fig. 2, 3b, filled diamonds). However, this interpretation should not be overestimated because the CP_50_ = 2.5 ± 0.2 μM remained near those of single dithiolane quartets in **19** and **1**. Further increase of dithiolane units in UUUUUU **12** was detrimental because complex **22** was insoluble in water (the same for EUUUUU, not finalized). The complementary complex **23** with EEEEEE **13** was soluble but inactive, as expected without U (Fig. 3b, empty diamonds, Table 1). Consistent with the dithiolane quartet hypothesis, solubilization of all-U **12** with all-E **13** in mixed complex **24** did not restore activity although the EU ratio was as in **1** and **15–18**, and solubility remained low. For the same reason, all other mixed complexes tested also failed to improve their uniform counterparts (not shown).

With the β quartet hypothesis, two amino acids of different stereochemistry suffice for accessing activities ranging from the best to worst with constitutional isomers, even diastereomers. Such functional extremes in isostructural systems were important to explore delivery into 3D tissue reliably. Also important was the availability of a formally isostructural CPP control. In 2D cell culture, complex **25** with KKKKKK **14** was in the range of the best dithiolane quartets **1**, **19** and **21**, but the cooperativity in the DRCs of thiol-mediated uptake was much reduced (Table 1, Fig. 3b, triangles). Neutralization or overcompensation of the positive charges from K_6_**14** with E_6_**13** was considered for a more adequate comparison with multianionic COC quartets. However, already the mixed complex **26** was poorly active (CP_50_ > 20 μM, Table 1, Fig. 3b, squares).

### Thiol-mediated cytosolic delivery in spheroids

With the required set of transporters in hand, quantitative detection of cytosolic delivery in deep tissue was considered next. Multicellular spheroids or organoids are emerging as attractive models to study penetration across deep tissues and barriers. Uptake into spheroids is mostly monitored by attaching fluorophores to transporters.^1–26^ However, this convenient method is known as unreliable from studies with 2D cell culture because information of intracellular localization, including surface binding and endosomal capture, is missing, and intensities can change for reasons unrelated to transport. The introduction of CAPA has transformed the uptake field because it allows for unambiguous, reliable and quantitative detection of cytosolic delivery in 2D cell culture.^55–57^ It was thus of interest to use the same CAPA for uptake studies in 3D cell culture, where co-localization experiments are technically challenging for several reasons, and functional response from HaloTags stably expressed on mitochondria would thus be even more important to determine cytosolic delivery in deep tissue quantitatively and unambiguously.

Spheroids of HGM cells with an average size of 350–400 μm were prepared in cell-repellent U-bottom 96-well plates following standard procedures (Fig. 4a).^1–26^ In cross-sectional confocal spinning disc microscopy (CSDM) images taken 80 μm from the surface, GFP emission was homogeneously distributed (Fig. 4b). Similarly the homogeneous cross-sectional TAMRA emission upon addition of **2** was consistent with unhindered passive diffusion of the small, cationic CAPA probe across the spheroid (Fig. 4c, m, red circles, 1c). Incubation of the HGM spheroids with 5 μM **19** for 6 h before the addition of CAPA probe **2** did not affect GFP emission (Fig. 4e) but produced a gradually decreasing TAMRA emission from the center to the periphery (Fig. 4f). Line-scan analysis of the TAMRA intensity along the cross-section in the CSDM image gave an almost linear decrease (Fig. 4m, green squares). Repetition of the same experiment with 20 μM **19** resulted in almost complete disappearance of all fluorescence (Fig. 4i, m, dark blue circles). Line-scan analysis of cross-sectional CSDM images taken already after 2 h rather than 6 h incubation revealed a penetration gradient similar to the one observed with 5 μM **19** after 6 h (Fig. 4l, m, blue circles). Comparison of the line-scan analyses for 20 μM **19** after 2 h and 6 h incubation illustrated how transporter **19** penetrates the spheroids with time from the periphery to the center as expected for operational transcytosis (Fig. 4m, l *vs.* i). These results demonstrated that at concentrations *c* > SP_50_, the CP_50_ of spheroids (Table 1), transporter **19** enters the cytosol homogeneously throughout the spheroid within 6 hours. At *c* ∼ SP_50_, thiol-mediated delivery decreases linearly with increasing depth because the limited amount of transporter **19** provided is gradually consumed by kinetically competing direct translocation and irreversible reaction with the HaloTags on the mitochondria of the cells on the way into the spheroids.

**Fig. 4:**
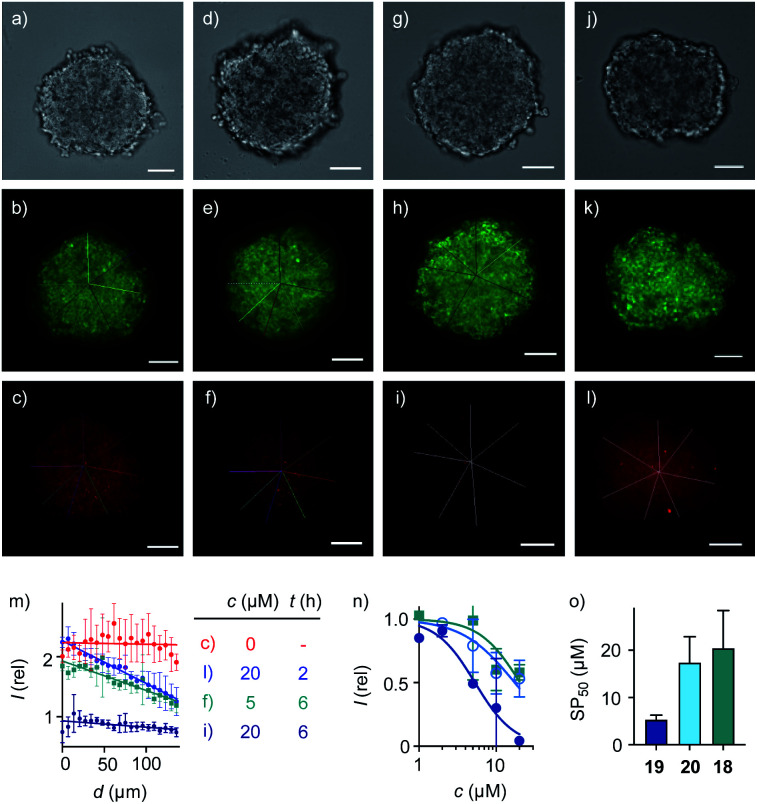
Cross-sectional CSDM images of HGM spheroids incubated with (a–c) **2** for 45 min; (d–f) 5 μM and (g–l) 20 μM **19** for 2 h (j–l) or 6 h (d–i) followed by **2** for 45 min, showing TL (a, d, g, j), GFP (b, e, h, k) and TAMRA channels (c, f, i, l) at 80 μm from the surface (scale bars: 100 μm). (m) Line-scan analysis of the TAMRA intensity corresponding to the cross-sections in c (red), f (green), i (dark blue) and l (blue), with intensities as a function of the distance from the center of the spheroid (error bars: standard deviation, *n* = 7). (n) 3D CAPA DRCs for **18** (squares), **19** (filled circles), and **20** (empty circles), with (o) SP_50_ values for comparison with CP_50_ values (Fig. 3d).

Quantitative DRCs for 3D CAPA integrating the entire spheroid gave for EEEUUU complex **19** an SP_50_ = 5.3 ± 0.9 μM that was nearly identical with the 2D CP_50_ = 3.8 ± 0.2 μM (Fig. 4n, o, *vs.*3c, d, Table 1). The decreasing activities of isomers **20** and **18** were cleanly translated from 2D to 3D, with preserved order. Compared to 2D CP_50_ > 20 μM, their weak activities even increased slightly to SP_50_ = 17 ± 6 μM for **20** and SP_50_ = 20 ± 8 μM for **18**, but errors naturally increased as well in the multicellular spheroids (Fig. 4n, o, *vs.*3c, d). Roughly identical DRCs for differently active transporters in 2D and 3D cell culture suggested that the uptake mechanisms are the same, that is a kinetic competition between thiol-mediated transcytosis and direct translocation (Fig. 1). Importantly, their similarity up to the shape of the DRC implied that the cytosolic delivery into deep tissue is an intrinsic characteristic of thiol-mediated uptake (Fig. 4n*vs.*3c).

**Fig. 5:**
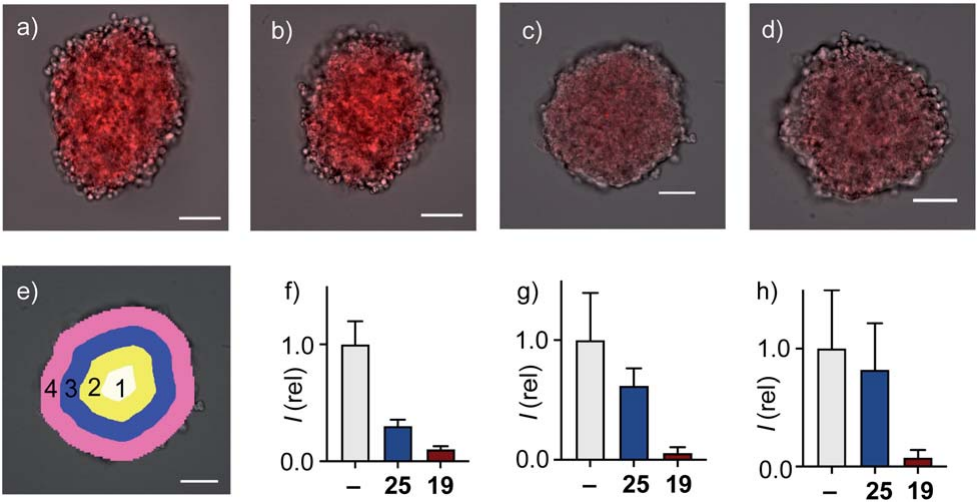
Merged cross-sectional CSDM images (TL: grey, TAMRA: red) of HGM spheroids incubated with (a) **2** for 45 min and (b) **25**, (c), **1** and (d) **19** (20 μM) for 6 h followed by **2** for 45 min. Images taken 80 μm from the bottom surface of the spheroids, scale bars: 100 μm. (e) Layer analysis exemplified with masks applied to (c). (f–h) Relative fluorescence from mask analysis in layers (f) 4, (g) 3 + 2 and (h) 1 of spheroids treated with nothing, **25** and **19** before adding **2**.

In 2D CAPA, the polycationic CPP complex **25** was as active as the best polyanionic dithiolane quartets **21**, **1** and **19** (Fig. 3b). In sharp contrast, CPP complex **25** failed to penetrate HGM spheroids (Fig. 5b). Emission of the CAPA probe **2** was suppressed only at the periphery (Fig. 5b), while dithiolane quartets **1** and **19** suppressed fluorescence throughout the spheroid (Fig. 5c and d). Layer analysis confirmed this impression quantitatively (Fig. 5e), revealing that CPP **25** reaches the cytosol only in the peripheral layer 4 (Fig. 5f), while activity in the middle layers 2–3 was weak and nearly negligible in the central layer 1 (Fig. 5g and h). Different activity in 3D cell culture (Fig. 5b–d) for isostructural transporters with similar activity in 2D (Fig. 3c) evinced that cytosolic delivery into deep tissue is a distinctive advantage of thiol-mediated uptake compared to classics such as CPPs.

### Thiol-mediated delivery of quantum dots in spheroids

**Fig. 6:**
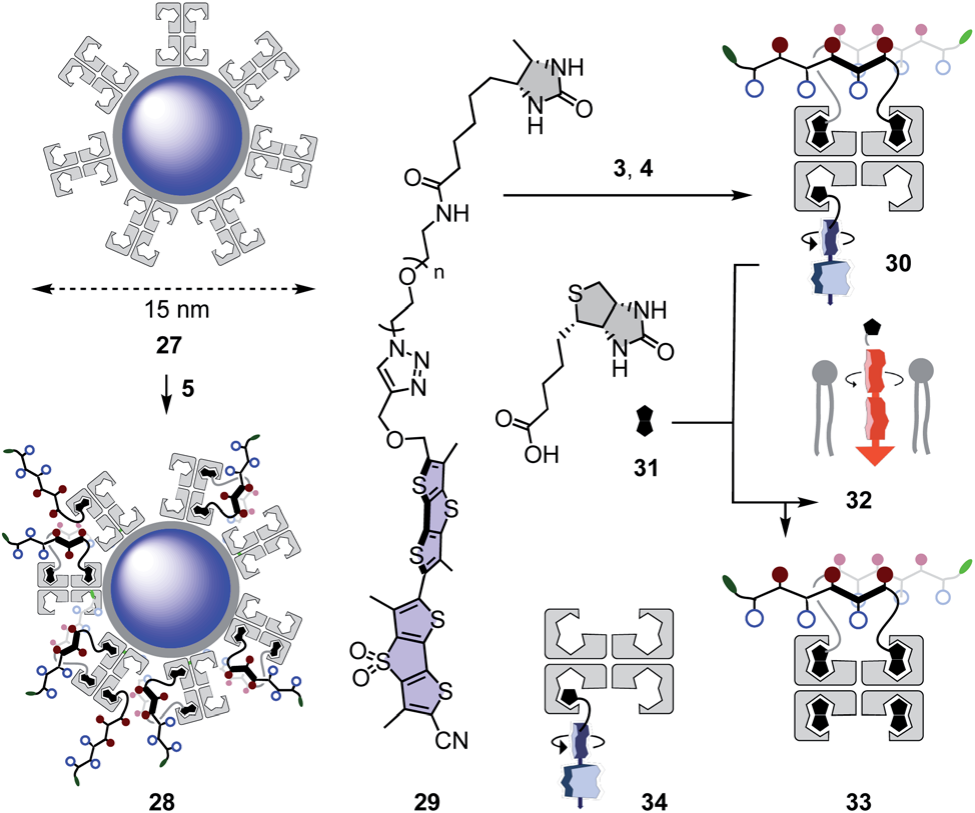
Schematic structures of non-penetrating QDs **27**, cell-penetrating QD eueueu complexes **28**, and SupraFlippers **29** complexed within cell-penetrating COC-streptavidin **30** and released with biotin **31** to label mitochondrial membranes **32** in spheroids; with side product **33** and non-penetrating control complex **34**.

Quantum dots (QDs) are notoriously difficult to deliver to the cytosol already in 2D culture.^61,62^ Most CPP conjugates end up trapped in endosomes. Thiol-mediated uptake has recently been shown with CPDs (cell-penetrating poly(disulfide)s)^61^ and diselenolanes^61,62^ to solve this problem in 2D culture. To probe for the even more ambitious delivery of QDs to the cytosol in multicellular spheroids, streptavidin-coated Qdot™ 605 **27** were loaded with 80 equivalents of eueueu peptide **5** (Fig. 6). This all-D peptide was selected among several candidates (Fig. 2) because aggregation of the resulting cell-penetrating system **28** was negligible. Aggregation of coated QDs is quite common, and more aggregation with L peptides than that with D enantiomers was interesting with regard to the diastereomeric active quartets formed with the chiral streptavidin templates.

**Fig. 7:**
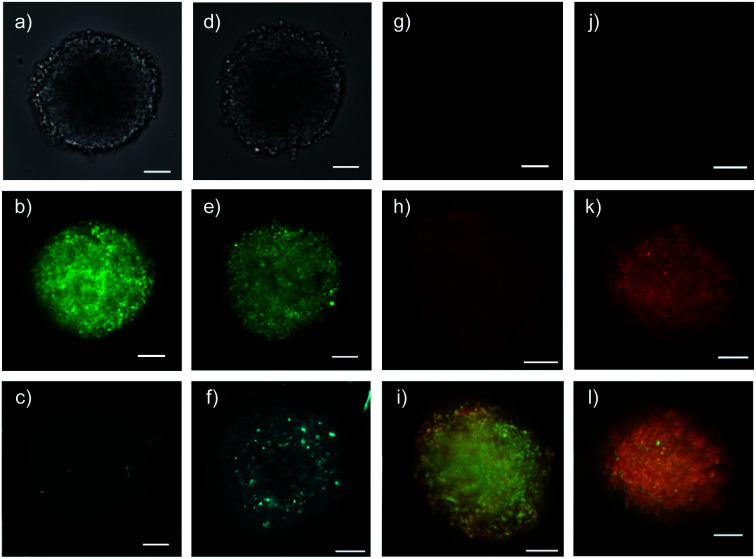
Cross-sectional CSDM images of control **27** (a–c) and COC-QD **28** (d–f, 10 nM) 6 h after the addition to HGM spheroids, showing TL (a and d), GFP (b and e) and QD channels (c and f), and of control **34** (g and j) and COC-flipper **30** (h, i, k, l, 5 μM) 4 h after the addition to HGM spheroids (g–i) and 2 h after subsequent addition of biotin **31** (j–l, 100 μM). Flipper emission (g, h, j, k) merged with GFP emission (i, l, green). All images at 80 μm from the spheroid surface (scale bars: 100 μm).

Six hours after the addition of control QDs **27** without COC transporters, the spheroid was intact (Fig. 7a) and the QD channel was essentially dark (Fig. 7c) compared to the internal standard in the GFP channel (Fig. 7b). This result confirmed the inability of protein-coated QDs to penetrate also HGM spheroids. In clear contrast, QDs **28** combined through streptavidin with dithiolane quartets produced brightly fluorescent spheroids (Fig. 7f). The fluorescence was very homogeneous, and the bright spots detected were minor compared to the images reported with other approaches.^1–26^ This homogeneous fluorescence reaching the center of the spheroids confirmed that spheroid penetration by thiol-mediated uptake is powerful also for most demanding substrates,^61^ and both the transporter and streptavidin are intact despite passing through endosomes multiple times.

### Thiol-mediated targeted delivery and controlled release of mechanophores in spheroids

Functional delivery into deep tissue with thiol-mediated uptake was probed with SupraFlippers **29** (Fig. 6).^63^ The mechanosensitive flipper probes have been introduced recently to image membrane tension in live cells. SupraFlippers **29** in particular were designed for the controlled release of the mechanophore in the membrane of interest within cells. They are equipped with a desthiobiotin, which binds to streptavidin **3** but not as strongly as biotin. Combined with a chloroalkylated dithiolane quartet in complex **30**, SupraFlippers should thus be delivered to the mitochondria in HGM spheroids. Addition of biotin **31** should then place the flipper into the surrounding mitochondrial membrane (**32**) for mechanosensing, and afford complex **33** as a side product. Because of the ease of expression of HaloTags, this approach allows us to target in principle any membrane of interest within cells.^64^

The addition of control flipper complex **34** did not produce any fluorescence in HGM spheroids (Fig. 7g). Consistent with efficient penetration of deep tissue, homogeneously fluorescent HGM spheroids were obtained from the addition of COC-flipper complex **30** (Fig. 7h). Flipper emission correlated well with GFP emission (green, Fig. 7i).

The addition of biotin **31** further increased the intensity of flipper emission (Fig. 7h vs. k, S11†). The change of color in the merged images of flipper and GFP emission confirmed that the increase in emission upon biotin addition occurred only with flippers and not with GFP (Fig. 7i *vs.* l). The selective increase of flipper emission was consistent with flipper release from complex **30** by ligand exchange with **31** and the formation of partially planarized, more emissive flippers **32** in the mitochondrial membranes, as previously confirmed by co-localization with GFP in 2D images.^63^

**Fig. 8:**
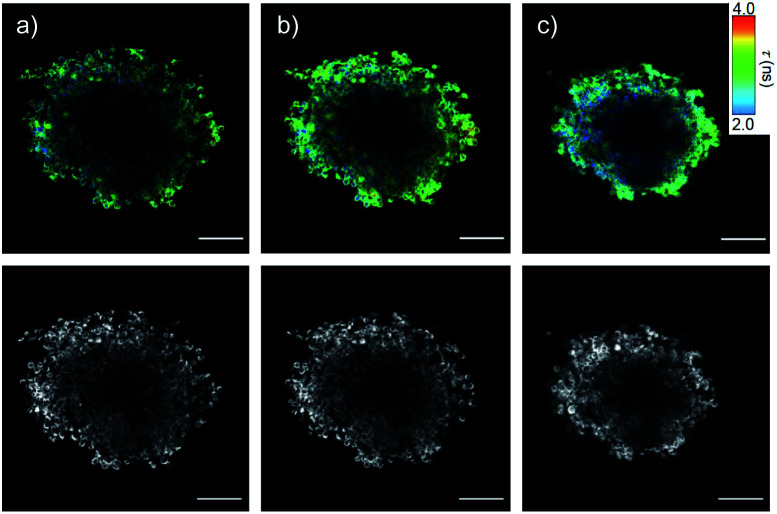
FLIM (top) and TL images (bottom) of COC-flipper **30** (5 μM) 4 h after addition to HGM spheroids (a), and 2 h after subsequent addition of biotin **31** (100 μM) before (b) and after hyperosmotic shock (NaCl, 1.0 M, c). Scale bars: 100 μm.

Preliminary results with fluorescence lifetime imaging microscopy (FLIM) confirmed detectability quite deep into spheroids (Fig. 8a, *τ*_av_ = 2.83 ns), increasing counts and lifetimes upon flipper release (Fig. 8b, *τ*_av_ = 2.95 ns), and decreasing lifetimes upon hyperosmotic stress (Fig. 8c, *τ*_av_ = 2.75 ns). Although average flipper lifetimes, sensitive to experimental conditions, were slightly shorter in 3D than in 2D culture, the decrease in response to tension was similar (3.3 to 3.1 ns in 2D (ref. 65)). This decrease is the key characteristic for operational mechanosensing within cells. Although there is much room for technical improvements for tension imaging by FLIM in spheroids, these results provide experimental support for the targeted delivery and spatio-temporal control of the release of SupraFlippers **29** on the mitochondria in the cytosol of multicellular spheroids by external chemical stimulation. Interestingly, only a few recent studies exist on substrate release in spheroids, triggered by changes in pH,^15,16^ disulfide reduction^14,19^ and enzymatic cleavage^13^ but not external chemical stimulation.

## Conclusions

We report that cytosolic delivery in deep tissue is a distinct, intrinsic property of thiol-mediated uptake, compatible with difficult substrates such as quantum dots, targeting and controlled release. Classical systems such as CPPs are confirmed not to meet this challenge convincingly. To secure these results, we also translated the CAPA method from 2D to 3D cell culture, which will be useful for the community. As for transport systems, the streptavidin-templated assembly of β quartets is introduced as a privileged motif, a finding that revives an old classic and expands streptavidin biotechnology in a new direction.

Cytosolic delivery into deep tissue is of broad interest in biology and medicine, from tumor treatment to crossing the blood–brain barrier. The here introduced thiol-mediated spheroid penetration is based on the grand principles of supramolecular chemistry. The pH dependence of dynamic covalent disulfide exchange with known surface thiols on the transferrin receptor conceivably accounts for thiol-mediated transcytosis, while dynamic covalent exchange cascades assure kinetically competing direct translocation. The transferrin receptor is known for canonical, transferrin-mediated transcytosis to deliver iron into the brain, but also to mediate thiol-mediated uptake and viral entry into cells. The perspectives emerging from this study are thus broad and substantial. They will further increase the general significance, scope, appreciation and use of thiol-mediated uptake.

## Experimental section

See the ESI.†

## Data availability

Data for this paper are available at Zenodo at https://doi.org/10.5281/zenodo.5515808.

## Author contributions

R. M. and S. T. synthesized the transporters; R. M., J. L.-A., D. M. and Q. L. developed spheroid imaging; S. M. directed the study, and all co-authors contributed to experiment design, data analysis and interpretation, and manuscript writing.

## Conflicts of interest

There are no conflicts to declare.

## Supplementary Material

SC-012-D1SC04828G-s001
