# Supplementary material for: Dithiolane quartets: thiol-mediated uptake enables cytosolic delivery in deep tissue
Source: Chem Sci. 2021 Oct 6;12(41):13922–9. doi: 10.1039/d1sc04828g (PMC8549803; doi:10.1039/d1sc04828g)
Supplement: SC-012-D1SC04828G-s001 [file SC-012-D1SC04828G-s001.pdf]

Electronic Supplementary Material (ESI) for Chemical Science.
This journal is © The Royal Society of Chemistry 2021

Electronic supplementary information

Dithiolane quartets: Thiol-mediated uptake enables cytosolic delivery in deep tissue

Rémi Martinent, Salman Tawffik, Javier López-Andarias, Dimitri Moreau, Quentin Laurent and Stefan Matile*

Department of Organic Chemistry, University of Geneva, Geneva, Switzerland

stefan.matile@unige.ch

Table of contents

1. Materials and methods S4

2. Synthesis S6

2.1. Peptide synthesis S6

2.1.1. Synthesis of peptide **5** S7

2.1.2. Synthesis of peptide **6** S14

2.1.3. Synthesis of peptide **7** S24

2.1.4. Synthesis of peptide **8** S30

2.1.5. Synthesis of peptide **9** S37

2.1.6. Synthesis of peptide **10** S46

2.1.7. Synthesis of peptide **11** S55

2.1.8. Synthesis of peptide **12** and **14** S62

2.1.9. Synthesis of peptide **13** S68

2.2. General procedure for streptavidin adducts formation S74

3. HC-CAPA S75

3.1. HGM cell line S75

3.2. 2D HC-CAPA S75

3.2.1. General HC-CAPA protocol S75

3.2.2. HC-CAPA analysis protocol S77

3.2.3. HC-CAPA results for uptake quantification S77

3.3. 3D HC-CAPA S78

3.3.1. General 3D HC-CAPA protocol S78

3.3.2. 3D HC-CAPA analysis protocol S79

3.3.3. 3D HC-CAPA results for uptake quantification S80

4. Delivery of QDs and SupraFlipper in spheroids S80

4.1. Quantum dot delivery in spheroids S80

4.1.1. Quantum dot adduct formation S80

4.1.2. Quantum dot delivery protocol S81

4.1.3. Quantum dot delivery analysis protocol S82

4.1.4. Quantum dot delivery quantification S82

4.2. SupraFlipper delivery and release in spheroids S83

4.2.1. SupraFlipper streptavidin complex formation S83

4.2.2. Delivery and release protocol S84

4.2.3. SupraFlipper delivery and release analysis protocol S84

4.2.4. SupraFlipper delivery and release quantification S85

4.2.5. FLIM in spheroids S86

5. References S87

6. NMR and HPLC-MS spectra S89

1. Materials and methods

As in ref. S1. Briefly, reagents for synthesis and commercially available final compounds were purchased from Sigma-Aldrich, Brunschwig, Alfa Aesar, Merck, TCI, Acros, Iris Biotech, and Click Chemistry Tools. Wild-type streptavidin was a generous gift from Prof. Thomas R. Ward (University of Basel). Analytical thin-layer chromatography (TLC) was performed on silica gel 60 F254 (Merck). Phosphate buffered saline (PBS, pH = 7.4), DMEM (GlutaMAX, 4.5 g/L D-glucose, with phenol red) medium, FluoroBrite DMEM (high D-Glucose) medium, Leibovitz's L-15 medium, Penicillin-Streptomycin, Fetal Bovine Serum, TrypLE Express Enzyme and Qdot™ 605 Streptavidin Conjugate were obtained from Thermo Fisher Scientific. μ-Plate 96-Well were obtained from Ibidi and Nunclon™ Sphera™ U-bottom 96-well sterile μ-Plates from Thermo Fischer Scientific. Fluorescence cellular imaging was performed using an IXM-C automated microscope from ImageXpress equipped with a Lumencor Aura III with 5 independently selectable solid-state light sources, bandpass filters and 5 objectives (4x to 60x). Sample preparation and washing on 96-well plates was performed using a Plate washer Biotek EL406® for the 2D cell cultures and with a Biotek MultiFlo™ FX for the spheroids. Fluorescence lifetime imaging microscopy (FLIM) was performed on a Leica SP8 DIVE FALCON microscope equipped with a white light laser (470nm - 670nm) and HyD detectors. Images were acquired at 20 MHz, using a 20X water immersion objective and maintaining the temperature of the chamber at 37 °C. Average lifetimes (τ_{av}) were calculated from a biexponential fit of the signal coming from the whole image. IR spectra were recorded on a Perkin Elmer Spectrum One FT-IR spectrometer (ATR, Golden Gate) and are reported as wavenumbers v in cm^{-1} with band intensities indicated as s (strong), m (medium), w (weak). ^{1}H and ^{13}C spectra were recorded on a Bruker 500 MHz spectrometer and are reported as chemical shifts (δ) in ppm relative to TMS (δ = 0). Spin multiplicities are reported as a singlet (s), doublet (d), triplet (t) and quartet (q) with coupling constants (J) given in Hz, or multiplet

(m). Broad peaks are marked as br. ^{1}H and ^{13}C resonances were assigned with the aid of additional information from 1D and 2D NMR spectra (H,H-COSY, DEPT 135, HSQC and HMBC). LC-MS were recorded using a Thermo Scientific Accela HPLC equipped with a Thermo C18 (5 cm x 2.1 mm, 1.9 μm particles) Hypersil gold column coupled with an LCQ Fleet three-dimensional ion trap mass spectrometer (ESI, Thermo Scientific) with a linear elution gradient either from 95% H_2O / 5% CH_3CN + 0.1% TFA to 10% H_2O / 90% CH_3CN + 0.1% TFA (B5) or from 70% H_2O / 30% CH_3CN + 0.1% TFA to 10% H_2O / 90% CH_3CN + 0.1% TFA (B30) in 4.0 min at a flow rate of 0.75 mL/min. HR ESI-MS for the characterization of new compounds were performed on a Xevo G2-S Tof (Waters) and are reported as mass-per-charge ratio *m/z* calculated and observed.

Abbreviations. AspA: Asparagusic acid; Boc: tert-butylcarbonyl; Calcd: Calculated; CAPA: Chloroalkane penetration assay; CSDM: Confocal scanning disc microscopy; CPS: Cell penetrating streptavidin; CP_{50}: Half maximal cell penetration concentration; DIPEA: Diisopropylethylamine; DMAP: 4-Dimethylaminopyridine; DMEM: Dulbecco's modified eagle medium; DMF: Dimethylformamide; DMSO: Dimethyl sulfoxide; EDCI: 1-Ethyl-3-(3-dimethylaminopropyl)carbodiimide; ESI: Electron spray ionization; EtOAc: Ethyl acetate; FBS: Fetal bovine serum; FLIM: Fluorescence lifetime microscopy; Fmoc: Fluorenylmethyloxycarbonyl; GFP: Green fluorescent protein; HATU: (1-[Bis(dimethylamino)methylene]-1H-1,2,3-triazolo[4,5-b]pyridinium 3-oxide hexafluorophosphate; HC: High-content; HGM cells: HeLa cells stably expressing a fusion protein of HaloTag and GFP on mitochondria; HRMS: High-resolution mass spectrometry; NHS: N-Hydroxysuccinimide; PBS: Phosphate-buffered saline; QD: Quantum dot; RP: Reverse phase; rt: Room temperature; TAMRA: 5-Carboxytetramethylrhodamine; TBTA: Tris(benzyltriazolylmethyl)amine; TFA: Trifluoroacetic acid; THF: Tetrahydrofuran; TIPS: Triisopropylsilane; TL: Transmitted light; TLC: Thin-layer chromatography.

2. Synthesis

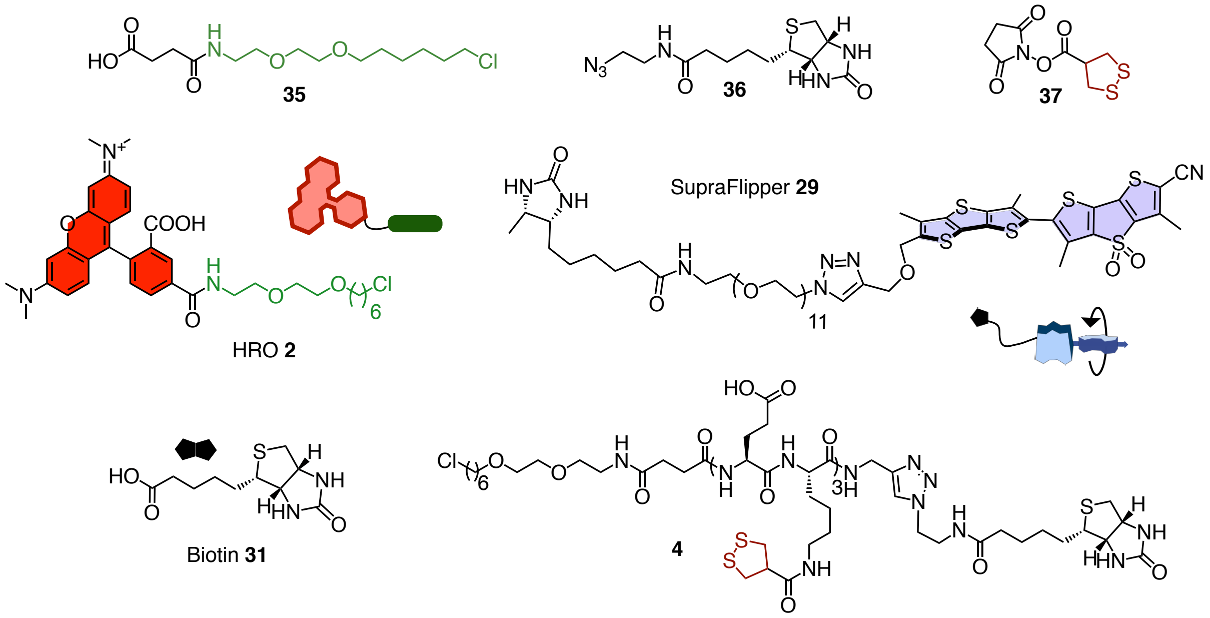

Figure S1 Structures of chloroalkane **35**, biotin-azide **36**, AspA-NHS **37**, HaloTag rhodamine HRO **2**, Supra Flipper **29**, Biotin **31** and peptide **4** with their respective schematic representations.

Compound 2 was prepared following a reported procedure described in ref. S2.

Compound 4 was prepared following a reported procedure described in ref. S1.

Compound 29 was prepared following a reported procedure described in ref. S3.

Compound 35 was prepared following a reported procedure described in ref. S4.

Compound 36 was prepared following a reported procedure described in ref. S5.

Compound 37 was prepared following a reported procedure described in ref. S6.

2.1. Peptide synthesis

General procedure for Fmoc-deprotection: A solution of *N*-Fmoc peptide in 2 M dimethylamine in THF was stirred at rt for 30 min. Solvent was removed *in vacuo*, crude was

dissolved in a minimal volume of CH_2Cl_2 and precipitated in pentane (x3). Crude was used in the next coupling reaction without further purification.

2.1.1. Synthesis of peptide 5

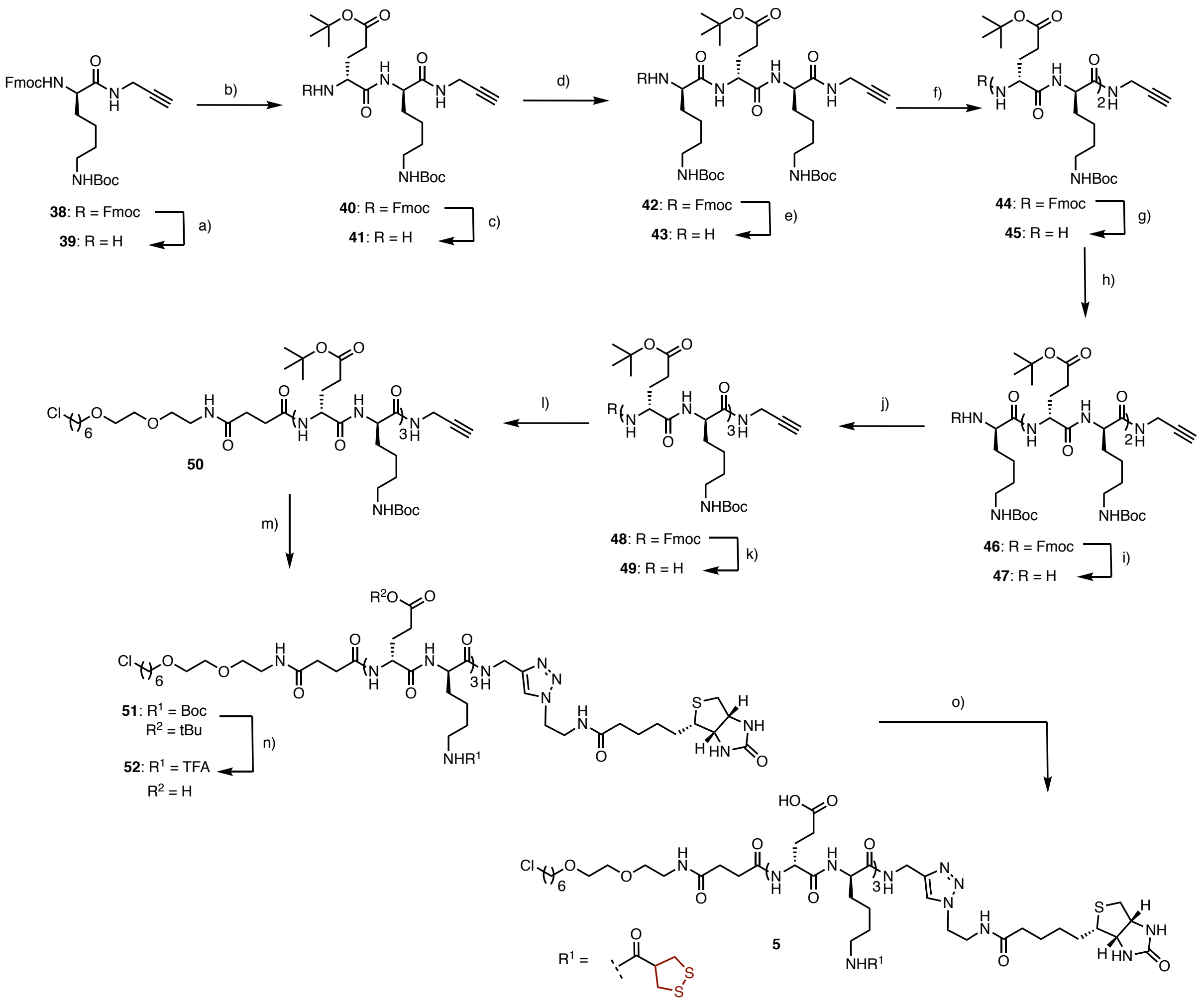

Scheme S1 (a) piperidine/DBU, CH_2Cl_2, rt, 30 min, 68%; (b) Fmoc-D-Glu(O*t*Bu)-OH, HATU, DIPEA, DMF, rt, 30 min, 89%; (c) 2 M dimethylamine in THF, rt, 30 min, 94%; (d) Fmoc-D-Lys(Boc)-OH, HATU, DIPEA, DMF, rt, 30 min, 96%; (e) 2 M dimethylamine in THF, rt, 30 min, 93%; (f) Fmoc-D-Glu(O*t*Bu)-OH, HATU, DIPEA, DMF, rt, 30 min, 89%; (g) 2 M dimethylamine in THF, rt, 30 min, *quant.*; (h) Fmoc-D-Lys(Boc)-OH, HATU, DIPEA, DMF, rt, 30 min, 91%; (i) 2 M dimethylamine in THF, rt, 30 min, 98%; (j) Fmoc-D-Glu(O*t*Bu)-OH, HATU, DIPEA, DMF, rt, 30 min, 94%; (k) 2 M dimethylamine in THF, rt, 30 min, 98%; (l)

35, HATU, DIPEA, DMF, rt, 30 min, 94%; (m) **36**, $CuSO_4 \cdot 5H_2O$, Na-ascorbate, TBTA, THF/H_2O, rt, 1 h, 76%; (n) TFA/TIPS/H_2O 95/2.5/2.5, rt, 30 min, 98%; (o) **37**, DIPEA, DMF, rt, 1 h, 32%.

Compound 38 was synthesized as described in ref. S8, using a D-amino acid derivative. $[\alpha]_D^{20}$ + 11 (*c* 0.50, MeOH); enantiomer: -11 (*c* 0.34, MeOH).[S7] Other spectroscopic data are identical to those of its enantiomer reported in ref. S8.

Compound 39 was synthesized as described in ref. S8, using the D-enantiomer **38** as starting material. Spectroscopic data are identical to those of its enantiomer reported in ref. S8.

Compound 40. To a solution of **39** (1.77 g, 6.25 mmol) in dry DMF (10 mL), was added a 30 seconds-premixed solution of HATU (2.83 g, 7.44 mmol), Fmoc-D-Glu(O*t*Bu)-OH (3.17 g, 7.44 mmol) and DIPEA (1.3 mL, 7.4 mmol) in dry DMF (10 mL). Reaction mixture was stirred at rt for 30 min. Reaction mixture was diluted with EtOAc and washed with $NaHCO_3$ sat. aq. solution (x3), 10% citric acid solution (x3), water (x1), 5% LiCl solution (x2) and brine. Organic phase was then dried with Na_2SO_4, filtered and concentrated. Crude was dissolved in a minimal amount of CH_2Cl_2 and precipitated in pentane (x3). Crude was then purified by flash column chromatography (BGB® Scorpius 80 g SiO_2 col., 50 mL/min, linear gradient 0 – 8% MeOH in CH_2Cl_2) to yield **40** as a colorless solid (3.83 g, 89%). $[\alpha]_D^{20}$ + 21 (*c* 0.50, MeOH); enantiomer: -21 (*c* 0.50, MeOH).[S1] Other spectroscopic data are identical to those of its enantiomer reported in ref. S1.

Compound 41 was prepared following the general procedure of Fmoc deprotection, starting from **40** (3.8 g, 5.5 mmol) in 25 mL of 2 M dimethylamine in THF, yielding **41** as a colorless solid (2.4 g, 94%).

Compound 42. To a solution of **41** (2.38 g, 5.07 mmol) in dry DMF (10 mL), was added a 30 seconds-premixed solution of HATU (2.31 g, 6.08 mmol), Fmoc-D-Lys(Boc)-OH (2.85

g, 6.08 mmol) and DIPEA (1.06 mL, 6.08 mmol) in dry DMF (10 mL). Reaction mixture was stirred at rt for 30 min. Reaction mixture was diluted with EtOAc and washed with $NaHCO_3$ sat. aq. solution (x3), 10% citric acid solution (x3), water (x1), 5% LiCl solution (x2) and brine. Organic phase was then dried with Na_2SO_4, filtered and concentrated. Crude was dissolved in a minimal amount of CH_2Cl_2 and precipitated in pentane (x3). Crude was then purified by flash column chromatography (BGB® Scorpius 80 g SiO_2 col., 50 mL/min, linear gradient 0 – 6% MeOH in CH_2Cl_2) to yield **42** as a colorless solid (4.49 g, 96%). $[\alpha]_D^{20}$ + 24 (*c* 0.50, MeOH); enantiomer: -27 (*c* 1.00, MeOH).[S1] Other spectroscopic data are identical to those of its enantiomer reported in ref. S1.

Compound 43 was prepared following the general procedure of Fmoc deprotection, starting from **42** (3.9 g, 4.2 mmol) in 20 mL of 2 M dimethylamine in THF, yielding **43** as a colorless solid (2.8 g, 93%).

Compound 44. To a solution of **43** (2.7 g, 3.9 mmol) in dry DMF (10 mL), was added a 30 seconds-premixed solution of HATU (1.8 g, 4.6 mmol), Fmoc-D-Glu(O*t*Bu)-OH (2.0 g, 4.6 mmol) and DIPEA (810 µL, 4.6 mmol) in dry DMF (10 mL). Reaction mixture was stirred at rt for 30 min. Reaction mixture was diluted with EtOAc and washed with $NaHCO_3$ sat. aq. solution (x3), 10% citric acid solution (x3), water (x1), 5% LiCl solution (x2) and brine. Organic phase was then dried with Na_2SO_4, filtered and concentrated. Crude was dissolved in a minimal amount of CH_2Cl_2 and precipitated in pentane (x3). Crude was then purified by flash column chromatography (BGB® Scorpius 80 g SiO_2 col., 50 mL/min, linear gradient 0 – 6% MeOH in CH_2Cl_2) to yield **44** as a colorless solid (3.8 g, 89%). $[\alpha]_D^{20}$ + 25 (*c* 0.50, MeOH); enantiomer: -27 (*c* 1.00, MeOH).[S1] Other spectroscopic data are identical to those of its enantiomer reported in ref. S1.

Compound 45 was prepared following the general procedure of Fmoc deprotection, starting from **44** (3.7 g, 3.3 mmol) in 20 mL of 2 M dimethylamine in THF, yielding **45** as a colorless solid (2.9 g, 100%).

Compound 46. To a solution of **45** (2.9 g, 3.3 mmol) in dry DMF (10 mL), was added a 30 seconds-premixed solution of HATU (1.5 g, 3.9 mmol), Fmoc-D-Lys(Boc)-OH (1.9 g, 3.9 mmol) and DIPEA (686 µL, 3.93 mmol) in dry DMF (10 mL). Reaction mixture was stirred at rt for 30 min. Reaction mixture was diluted with EtOAc and washed with $NaHCO_3$ sat. aq. solution (x3), 10% citric acid solution (x3), water (x1), 5% LiCl solution (x2) and brine. Organic phase was then dried with Na_2SO_4, filtered and concentrated. Crude was dissolved in a minimal amount of CH_2Cl_2 and precipitated in pentane (x3). Crude was then purified by flash column chromatography (BGB® Scorpius 80 g SiO_2 col., 50 mL/min, linear gradient 0 – 6% MeOH in CH_2Cl_2) to yield **46** as a colorless solid (4.0 g, 91%). $[\alpha]_D^{20}$ + 24 (*c* 0.50, MeOH); enantiomer: -20 (*c* 1.00, MeOH).[S1] Other spectroscopic data are identical to those of its enantiomer reported in ref. S1.

Compound 47 was prepared following the general procedure of Fmoc deprotection, starting from **46** (3.3 g, 2.5 mmol) in 15 mL of 2 M dimethylamine in THF, yielding **47** as a colorless solid (2.7 g, 98%).

Compound 48. To a solution of **47** (2.6 g, 2.4 mmol) in dry DMF (10 mL), was added a 30 seconds-premixed solution of HATU (1.1 g, 2.8 mmol), Fmoc-D-Glu(O*t*Bu)-OH (1.2 g, 2.8 mmol) and DIPEA (494 µL, 2.8 mmol) in dry DMF (10 mL). Reaction mixture was stirred at rt for 30 min. Reaction mixture was diluted with EtOAc and washed with $NaHCO_3$ sat. aq. solution (x3), 10% citric acid solution (x3), water (x1), 5% LiCl solution (x2) and brine. Organic phase was then dried with Na_2SO_4, filtered and concentrated. Crude was dissolved in a minimal amount of CH_2Cl_2 and precipitated in pentane (x3). Crude was then purified by flash column chromatography (BGB® Scorpius 80 g SiO_2 col., 50 mL/min, linear gradient 0 – 6%

MeOH in CH_2Cl_2) to yield **48** as a colorless solid (3.4 g, 94%). $[\alpha]_D^{20}$ + 16 (*c* 0.50, MeOH); enantiomer: -14 (*c* 1.00, MeOH).[S1] Other spectroscopic data are identical to those of its enantiomer reported in ref. S1.

Compound 49 was prepared following the general procedure of Fmoc deprotection, starting from **48** (1.2 g, 0.8 mmol) in 10 mL of 2 M dimethylamine in THF, yielding **49** as a colorless solid (1.0 g, 98%).

Compound 50. To a solution of **49** (1.0 g, 0.8 mmol) in dry DMF (6 mL), was added a 30 seconds-premixed solution of HATU (350 mg, 0.920 mmol), **35** (298 mg, 0.920 mmol) and DIPEA (161 µL, 0.920 mmol) in dry DMF (6 mL). Reaction mixture was stirred at rt for 30 min. Reaction mixture was diluted with EtOAc and washed with $NaHCO_3$ sat. aq. solution (x3), 10% citric acid solution (x3), water (x1), 5% LiCl solution (x2) and brine. Organic phase was then dried with Na_2SO_4, filtered and concentrated. Crude was dissolved in a minimal amount of CH_2Cl_2 and precipitated in pentane (x3). Crude was then purified by flash column chromatography (BGB® Scorpius 25 g SiO_2 col., 40 mL/min, linear gradient 0 – 8% MeOH in CH_2Cl_2) to yield **50** as a colorless solid (1.15 g, 94%). $[\alpha]_D^{20}$ – 2.5 (*c* 0.50, MeOH); enantiomer: + 4.9 (*c* 1.00, MeOH).[S1] Compound spectroscopic data are identical to its enantiomer reported in ref. S1.

Compound 51. To a solution of **50** (500 mg, 0.312 mmol) in THF/H_2O 2/1 (9 mL), **36** (116 mg, 0.371 mmol), $CuSO_4{\cdot}5H_2O$ (80 mg, 0.31 mmol), sodium ascorbate (122 mg, 0.62 mmol) and TBTA (16 mg, 0.031 mmol) were added and the reaction mixture was stirred at rt for 1 h. Solvent was removed *in vacuo*, H_2O and CH_2Cl_2 were added, phases were separated, and aqueous phase was extracted by CH_2Cl_2 (x3). Organic phases were collected together, washed with brine (x1), dried over Na_2SO_4, filtered and concentrated. Crude was purified by flash column chromatography (BGB® Scorpius 25g, 25 mL/min, linear gradient 0 – 10% MeOH in CH_2Cl_2) to yield **51** as a colorless solid (452 mg, 76%). R_f (CH_2Cl_2 + 10% MeOH):

0.3; Mp: 198.6 – 199.7 °C; $[\alpha]_D^{20}$ + 3.5 (*c* 0.50, MeOH); IR (neat): 3280 (w), 2931 (w), 1694 (s), 1626 (s), 1522 (s), 1448 (m), 1365 (s), 1248 (s), 1152 (s), 626 (m); ^{1}H NMR (500 MHz, CD_3OD): 7.91 (s, 1H), 4.66 (d, $^2J_{H-H}$ = 15.4 Hz, 1H), 4.54 – 4.48 (m, 3H), 4.37 (d, $^2J_{H-H}$ = 15.4 Hz, 1H), 4.34 (dd, $^3J_{H-H}$ = 7.9, 4.4 Hz, 1H), 4.21 (dd, $^3J_{H-H}$ = 9.8, 4.6 Hz, 1H), 4.13 (dd, $^3J_{H-H}$ = 9.8, 4.6 Hz, 1H), 4.06 (t, $^3J_{H-H}$ = 7.6 Hz, 2H), 4.01 (t, $^3J_{H-H}$ = 7.8 Hz, 1H), 3.93 (dd, $^3J_{H-H}$ = 9.5, 5.5 Hz, 1H), 3.73 – 3.62 (m, 2H), 3.62 – 3.58 (m, 6H), 3.56 (t, $^3J_{H-H}$ = 6.6 Hz, 2H), 3.50 (t, $^3J_{H-H}$ = 6.6 Hz, 2H), 3.46 – 3.35 (m, 2H), 3.22 (ddd, $^3J_{H-H}$ = 8.6, 6.1, 4.4 Hz, 1H), 3.09 – 2.97 (m, 6H), 2.95 (dd, $^2J_{H-H}$ = 12.7, $^3J_{H-H}$ = 5.0 Hz, 1H), 2.87 – 2.77 (m, 1H), 2.70 (d, $^2J_{H-H}$ = 12.7 Hz, 1H), 2.63 – 2.50 (m, 3H), 2.49 – 2.38 (m, 4H), 2.36 – 2.25 (m, 2H), 2.25 – 2.07 (m, 5H), 2.04 – 1.91 (m, 5H), 1.90 – 1.68 (m, 8H), 1.65 – 1.32 (m, 20H), 1.45 (s, 9H), 1.44 (s, 18H), 1.43 (s, 9H), 1.42 (s, 18H); ^{13}C NMR (126 MHz, CD_3OD): 176.8 (C), 176.6 (C), 176.6 (C), 176.5 (C), 176.3 (2xC), 175.1 (C), 175.0 (C), 174.9 (C), 173.7 (C), 173.4 (C), 173.2 (C), 166.1 (C), 158.4 (C), 158.4 (C), 158.3 (C), 147.0 (C), 124.6 (CH), 81.9 (2xC), 81.6 (C), 79.8 (2xC), 79.7 (C), 72.3 (CH_2), 71.3 (CH_2), 71.2 (CH_2), 70.5 (CH_2), 63.3 (CH), 61.6 (CH), 58.1 (CH), 57.8 (CH), 57.0 (2xCH), 57.0 (CH), 56.3 (CH), 56.2 (CH), 50.5 (CH_2), 45.7 (CH_2), 41.3 (CH_2), 41.3 (CH_2), 41.1 (2xCH_2), 40.7 (CH_2), 40.5 (CH_2), 36.7 (CH_2), 36.1 (CH_2), 33.8 (CH_2), 33.2 (CH_2), 32.7 (CH_2), 32.4 (2xCH_2), 32.2 (CH_2), 31.9 (CH_2), 31.7 (CH_2), 31.0 (CH_2), 30.6 (CH_2), 30.6 (CH_2), 30.5 (CH_2), 30.5 (CH_2), 29.6 (CH_2), 29.5 (CH_2), 28.9 (3xCH_3), 28.9 (6xCH_3), 28.6 (3xCH_3), 28.5 (3xCH_3), 28.5 (3xCH_3), 27.8 (CH_2), 27.7 (CH_2), 27.4 (CH_2), 26.9 (CH_2), 26.7 (CH_2), 26.5 (CH_2), 24.8 (2xCH_2), 24.6 (CH_2).

Compound 52. A solution of **51** (349 mg, 182 µmol) in TFA/H_2O/TIPS 95/2.5/2.5 (5 mL) was stirred at rt for 30 min. Solvent was removed *in vacuo*, crude was dissolved in small amount of MeOH and was precipitated in Et_2O (x3) to yield **52** as a colorless TFA salt (321 mg, 98%). Crude was used in the next reaction without further purification.

Compound 5. To a solution of **52** (40 mg, 22 μmol) in DMF (800 μL), **37** (22 mg, 89 μmol) and DIPEA (31 μL, 178 μmol) were added and reaction mixture was stirred at rt for 1 h. Crude reaction mixture was purified by RP flash chromatography (Biotage® SNAP Ultra 12 g C18, 12 mL/min, linear gradient 20 – 50% CH_3CN in H_2O with 0.1% TFA) to yield **5** as a colorless solid (13 mg, 32%). ^{1}H NMR (500 MHz, DMSO-d_6/D_2O 60/1): 7.82 (s, 1H), 4.37 (t, $^3J_{H\text{-}H}$ = 6.2 Hz, 2H), 4.33 (d, $^2J_{H\text{-}H}$ = 15.2 Hz, 1H), 4.32 – 4.28 (m, 1H), 4.25 (d, $^2J_{H\text{-}H}$ = 15.2 Hz, 1H), 4.22 – 4.09 (m, 6H), 3.61 (t, $^3J_{H\text{-}H}$ = 6.6 Hz, 2H), 3.51 – 3.42 (m, 6H), 3.40 – 3.33 (m, 10H), 3.21 – 3.07 (m, 12H), 3.06 – 2.98 (m, 6H), 2.82 (dd, $^2J_{H\text{-}H}$ = 12.5, $^3J_{H\text{-}H}$ = 5.1 Hz, 1H), 2.58 (d, $^2J_{H\text{-}H}$ = 12.5 Hz, 1H), 2.41 – 2.30 (m, 4H), 2.29 – 2.17 (m, 6H), 2.06 – 2.00 (m, 2H), 1.97 – 1.84 (m, 3H), 1.80 – 1.55 (m, 10H), 1.54 – 1.42 (m, 6H), 1.40 – 1.33 (m, 9H), 1.33 – 1.19 (m, 10H); ^{13}C NMR (126 MHz, , DMSO-d_6): 174.0 (C), 173.9 (C), 173.9 (C), 172.7 (C), 172.7 (C), 172.1 (C), 172.0 (C), 171.9 (C), 171.7 (C), 171.5 (C), 171.3 (C), 171.0 (C), 170.3 (C), 170.3 (2xC), 162.8 (C), 144.7 (C), 123.0 (CH), 70.3 (CH_2), 69.7 (CH_2), 69.5 (CH_2), 69.1 (CH_2), 61.0 (CH), 59.2 (CH), 55.4 (CH_2), 53.2 (CH), 52.7 (CH), 52.7 (CH), 52.5 (CH), 52.1 (CH), 52.0 (CH), 51.5 (3xCH), 48.7 (CH_2), 45.5 (CH_2), 42.2 (6xCH_2), 38.8 (CH_2), 38.8 (CH_2), 38.7 (CH_2), 38.7 (CH_2), 38.6 (2xCH_2), 35.1 (CH_2), 34.3 (CH_2), 32.1 (CH_2), 31.6 (CH_2), 31.4 (CH_2), 30.8 (CH_2), 30.7 (CH_2), 30.5 (CH_2), 30.2 (2xCH_2), 30.1 (CH_2), 29.1 (CH_2), 28.7 (3xCH_2), 28.2 (CH_2), 28.1 (CH_2), 27.1 (CH_2), 26.9 (CH_2), 26.8 (CH_2), 26.2 (CH_2), 25.2 (CH_2), 25.0 (CH_2), 23.1 (CH_2), 22.8 (CH_2), 22.8 (CH_2); HRMS (ESI, +ve) calcd for $C_{74}H_{118}ClN_{17}O_{21}S_7$ ($[M + H]^+$): 1840.6495, found: 1840.6527; LC-MS (ESI, B30): R_t 1.66 min, 1842 (50, $[M+H]^+$), 921 (100, $[M+2H]^{2+}$).

2.1.2. Synthesis of peptide 6

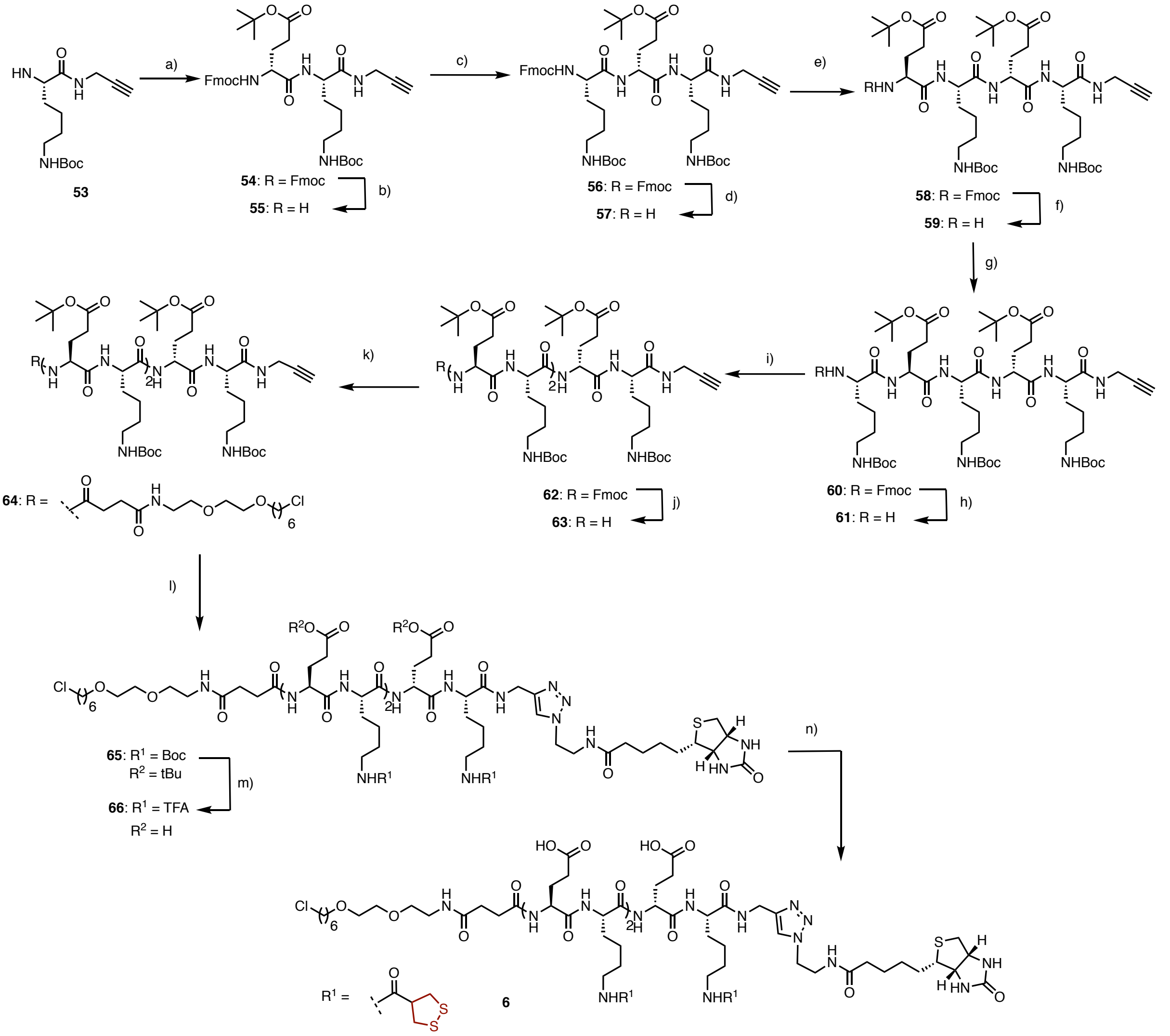

Scheme S2 (a) Fmoc-D-Glu(O*t*Bu)-OH, HATU, DIPEA, DMF, rt, 1 h, 85%; (b) 2 M dimethylamine in THF, rt, 30 min, 83%; (c) Fmoc-Lys(Boc)-OH, HATU, DIPEA, DMF, rt, 1 h, 90%; (d) 2 M dimethylamine in THF, rt, 30 min, 99%; (e) Fmoc-Glu(O*t*Bu)-OH, HATU, DIPEA, DMF, rt, 30 min, 96%; (f) 2 M dimethylamine in THF, rt, 30 min, 90%; (g) Fmoc-Lys(Boc)-OH, HATU, DIPEA, DMF, rt, 30 min, 86%; (h) 2 M dimethylamine in THF, rt, 30 min, 96%; (i) Fmoc-Glu(O*t*Bu)-OH, HATU, DIPEA, DMF, rt, 30 min, 93%; (j) 2 M dimethylamine in THF, rt, 30 min, 98%; (k) **35**, HATU, DIPEA, DMF, rt, 30 min, 90%; (l) **36**,

$CuSO_4 \cdot 5H_2O$, Na-ascorbate, TBTA, THF/H_2O, rt, 1 h, 86%; (m) TFA/TIPS/H_2O 95/2.5/2.5, rt, 30 min, 91%; (n) **37**, DIPEA, DMF, rt, 1 h, 17%.

Compound 53 was prepared following a reported procedure described in ref. S8.

Compound 54. To a solution of **53** (2.11 g, 7.44 mmol) in dry DMF (10 mL), was added a 30 seconds-premixed solution of HATU (3.80 g, 8.92 mmol), Fmoc-D-Glu(O*t*Bu)-OH (3.40 g, 8.92 mmol) and DIPEA (1.56 mL, 8.92 mmol) in dry DMF (10 mL). Reaction mixture was stirred at rt for 30 min. Reaction mixture was diluted with EtOAc and washed with $NaHCO_3$ sat. aq. solution (x3), 10% citric acid solution (x3), water (x1), 5% LiCl solution (x2) and brine. Organic phase was then dried with Na_2SO_4, filtered and concentrated. Crude was dissolved in a minimal amount of CH_2Cl_2 and precipitated in pentane (x3). Crude was then purified by flash column chromatography (BGB® Scorpius 80 g SiO_2 col., 40 mL/min, linear gradient 0 – 8% MeOH in CH_2Cl_2) to yield **54** as a colorless solid (4.39 g, 85%). R_f (CH_2Cl_2 + 10% MeOH): 0.6; Mp: 139.8 – 140.9 °C; $[\alpha]_D^{20}$ – 0.7 (*c* 0.50, MeOH); IR (neat): 3290 (m), 2933 (w), 1721 (m), 1685 (s), 1635 (s), 1525 (s), 1447 (m), 1366 (m), 1251 (s), 1157 (s), 738 (s), 638 (m); ^1H NMR (400 MHz, CD_3OD): 7.80 (d, $^3J_{H\text{-}H}$ = 7.4 Hz, 2H), 7.67 (d, $^3J_{H\text{-}H}$ = 7.4 Hz, 1H), 7.66 (d, $^3J_{H\text{-}H}$ = 7.4 Hz, 1H),7.39 (t, $^3J_{H\text{-}H}$ = 7.4 Hz, 2H), 7.34 – 7.28 (m, 2H), 4.45 – 4.36 (m, 2H), 4.30 (dd, $^3J_{H\text{-}H}$ = 9.4, 4.7 Hz, 1H), 4.23 (t, $^3J_{H\text{-}H}$ = 6.9 Hz, 1H), 4.10 (dd, $^3J_{H\text{-}H}$ = 8.5, 5.9 Hz, 1H), 3.93 (dd, $^2J_{H\text{-}H}$ = 17.5, $^4J_{H\text{-}H}$ = 2.5 Hz, 1H), 3.86 (dd, $^2J_{H\text{-}H}$ = 17.5, $^4J_{H\text{-}H}$ = 2.5 Hz, 1H), 3.08 – 2.93 (m, 2H), 2.47 (t, $^4J_{H\text{-}H}$ = 2.5 Hz, 1H), 2.32 (t, $^3J_{H\text{-}H}$ = 7.5 Hz, 1H), 2.06 – 1.97 (m, 1H), 1.95 – 1.79 (m, 2H), 1.70 – 1.57 (m, 1H), 1.53 – 1.30 (m, 4H), 1.45 (s, 9H), 1.42 (s, 9H); ^{13}C NMR (101 MHz, CD_3OD): 174.6 (C), 173.8 (C), 173.7 (C), 158.6 (C), 158.5 (C), 145.3 (C), 145.2 (C), 142.6 (2xC), 128.8 (2xCH), 128.2 (CH), 128.2 (CH), 126.3 (CH), 126.2 (CH), 121.0 (CH), 120.9 (CH), 81.9 (C), 80.4 (C), 79.9 (C), 72.2 (C), 68.1 (CH_2), 56.1 (CH), 54.5 (CH),

48.4 (CH), 41.1 (CH_2), 32.6 (CH_2), 32.4 (CH_2), 30.4 (CH_2), 29.5 (CH_2), 28.8 (3xCH_3), 28.4 (3xCH_3), 28.0 (CH_2), 24.2 (CH_2).

Compound 55 was prepared following the general procedure of Fmoc deprotection, starting from **54** (4.0 g, 5.2 mmol) in 15 mL of 2 M dimethylamine in THF, yielding **55** as a colorless solid (2.27 g, 83%).

Compound 56. To a solution of **55** (1.86 g, 3.97 mmol) in dry DMF (10 mL), was added a 30 seconds-premixed solution of HATU (1.80 g, 4.76 mmol), Fmoc-Lys(Boc)-OH (2.20 g, 4.76 mmol) and DIPEA (809 µL, 4.76 mmol) in dry DMF (10 mL). Reaction mixture was stirred at rt for 30 min. Reaction mixture was diluted with EtOAc and washed with $NaHCO_3$ sat. aq. solution (x3), 10% citric acid solution (x3), water (x1), 5% LiCl solution (x2) and brine. Organic phase was then dried with Na_2SO_4, filtered and concentrated. Crude was dissolved in a minimal amount of CH_2Cl_2 and precipitated in pentane (x3). Crude was then purified by flash column chromatography (BGB® Scorpius 80 g SiO_2 col., 40 mL/min, linear gradient 0 – 8% MeOH in CH_2Cl_2) to yield **56** as a colorless solid (3.27 g, 90%). R_f (CH_2Cl_2 + 10% MeOH): 0.5; Mp: 175.1 – 175.7 °C; $[\alpha]_D^{20}$ – 3.8 (*c* 0.50, MeOH); IR (neat): 3286 (m), 2934 (w), 1683 (s), 1631 (s), 1519 (s), 1450 (m), 1366 (m), 1249 (s), 1156 (s), 738 (m), 638 (m); ^{1}H NMR (400 MHz, CD_3OD): 7.80 (d, $^3J_{H\text{-}H}$ = 7.4 Hz, 2H), 7.66 (d, $^3J_{H\text{-}H}$ = 7.4 Hz, 2H), 7.39 (t, $^3J_{H\text{-}H}$ = 7.4 Hz, 2H), 7.31 (t, $^3J_{H\text{-}H}$ = 7.4 Hz, 2H), 4.43 (dd, $^3J_{H\text{-}H}$ = 10.5, 6.8 Hz, 1H), 4.35 (dd, $^3J_{H\text{-}H}$ = 9.4, 5.2 Hz, 1H), 4.27 – 4.20 (m, 2H), 4.00 (dd, $^3J_{H\text{-}H}$ = 8.2, 6.2 Hz, 1H), 3.93 (d, $^4J_{H\text{-}H}$ = 2.5 Hz, 2H), 3.05 (t, $^3J_{H\text{-}H}$ = 6.7 Hz, 2H), 2.99 – 2.83 (m, 2H), 2.49 (t, $^4J_{H\text{-}H}$ = 2.5 Hz, 1H), 2.31 (t, $^3J_{H\text{-}H}$ = 8.0 Hz, 2H), 2.22 – 2.10 (m, 1H), 1.93 – 1.55 (m, 5H), 1.54 – 1.24 (m, 8H), 1.43 (s, 18H), 1.41 (s, 9H); ^{13}C NMR (101 MHz, CD_3OD): 175.5 (C), 173.9 (C), 173.7 (C), 173.6 (C), 158.6 (2xC), 158.4 (C), 145.2 (C), 145.2 (C), 142.6 (C), 142.6 (C), 128.8 (CH), 128.8 (CH), 128.3 (CH), 128.2 (CH), 126.3 (CH), 126.2 (CH), 121.0 (CH), 120.9 (CH), 81.9 (C), 80.5 (C), 79.9 (C), 79.9 (C), 72.3 (CH), 68.1 (CH_2), 57.0 (CH), 54.7 (CH), 54.1 (CH), 48.4 (CH), 41.1 (CH_2),

41.1 (CH_2), 32.7 (CH_2), 32.4 (CH_2), 32.3 (CH_2), 30.6 (CH_2), 30.4 (CH_2), 29.5 (CH_2), 28.8 (6xCH_3), 28.4 (3xCH_3), 27.7 (CH_2), 24.2 (CH_2), 24.1 (CH_2).

Compound 57 was prepared following the general procedure of Fmoc deprotection, starting from **56** (3.2 g, 3.5 mmol) in 20 mL of 2 M dimethylamine in THF, yielding **57** as a colorless solid (2.4 g, 99%).

Compound 58. To a solution of **57** (1.45 g, 2.08 mmol) in dry DMF (5 mL), was added a 30 seconds-premixed solution of HATU (949 mg, 2.50 mmol), Fmoc-Glu(O*t*Bu)-OH (1.06 g, 2.50 mmol) and DIPEA (435 µL, 2.50 mmol) in dry DMF (5 mL). Reaction mixture was stirred at rt for 30 min. Reaction mixture was diluted with EtOAc and washed with $NaHCO_3$ sat. aq. solution (x3), 10% citric acid solution (x3), water (x1), 5% LiCl solution (x2) and brine. Organic phase was then dried with Na_2SO_4, filtered and concentrated. Crude was dissolved in a minimal amount of CH_2Cl_2 and precipitated in pentane (x3). Crude was then purified by flash column chromatography (BGB® Scorpius 40 g SiO_2 col., 40 mL/min, linear gradient 0 – 8% MeOH in CH_2Cl_2) to yield **58** as a colorless solid (2.21 g, 96%). R_f (CH_2Cl_2 + 10% MeOH): 0.5; Mp: 172.9 – 173.6 °C; $[\alpha]_D^{20}$ - 7.9 (*c* 0.50, MeOH); IR (neat): 3287 (m), 2931 (w), 1725 (m), 1688 (s), 1631 (s), 1524 (s), 1448 (m), 1365 (m), 1250 (s), 1152 (s), 737 (m), 640 (m); ^1H NMR (400 MHz, CD_3OD): 7.80 (d, $^3J_{H-H}$ = 7.4 Hz, 2H), 7.67 (d, $^3J_{H-H}$ = 7.4 Hz, 1H), 7.66 (d, $^3J_{H-H}$ = 7.4 Hz, 1H),7.39 (t, $^3J_{H-H}$ = 7.4 Hz, 2H), 7.31 (t, J = 7.4 Hz, 2H), 4.44 (dd, $^3J_{H-H}$ = 10.5, 7.2 Hz, 1H), 4.38 (dd, $^3J_{H-H}$ = 10.5, 7.2 Hz, 1H), 4.36 – 4.25 (m, 2H), 4.23 (t, $^3J_{H-H}$ = 6.7 Hz, 2H), 4.09 (dd, $^3J_{H-H}$ = 8.9, 5.3 Hz, 1H), 3.93 (d, $^4J_{H-H}$ = 2.5 Hz, 2H), 3.02 (t, $^3J_{H-H}$ = 6.9 Hz, 4H), 2.53 (t, $^4J_{H-H}$ = 2.5 Hz, 1H), 2.42 – 2.23 (m, 4H), 2.22 – 2.09 (m, 1H), 2.11 – 1.99 (m, 1H), 2.00 – 1.79 (m, 4H), 1.77 – 1.62 (m, 2H), 1.53 – 1.30 (m, 8H), 1.45 (s, 9H), 1.42 (s, 9H), 1.41 (s, 9H), 1.41 (s, 9H); ^{13}C NMR (101 MHz, CD_3OD): 174.7 (C), 174.6 (C), 174.0 (C), 173.9 (C), 173.8 (C), 173.7 (C), 158.8 (C), 158.5 (2xC), 145.4 (C), 145.1 (C), 142.6 (C), 142.6 (C), 128.8 (CH), 128.8 (CH), 128.2 (2xCH), 126.3 (2xCH), 121.0 (2xCH), 81.9 (2xC), 80.6

(C), 79.8 (2xC), 72.3 (CH), 68.2 (CH_2), 56.1 (CH), 55.2 (CH), 54.8 (CH), 54.3 (CH), 48.4 (CH), 41.2 (2xCH_2), 32.7 (2xCH_2), 32.4 (CH_2), 32.0 (CH_2), 30.5 (CH_2), 30.4 (CH_2), 29.6 (CH_2), 28.8 (3xCH_3), 28.8 (3xCH_3), 28.4 (3xCH_3), 28.4 (3xCH_3), 28.2 (CH_2), 27.7 (CH_2), 24.3 (CH_2), 24.2 (CH_2).

Compound 59 was prepared following the general procedure of Fmoc deprotection, starting from **58** (2.20 g, 1.99 mmol) in 10 mL of 2 M dimethylamine in THF, yielding **59** as a colorless solid (1.59 g, 90%).

Compound 60. To a solution of **59** (790 mg, 0.896 mmol) in dry DMF (4 mL), was added a 30 seconds-premixed solution of HATU (409 mg, 1.08 mmol), Fmoc-Lys(Boc)-OH (504 mg, 1.08 mmol) and DIPEA (187 µL, 1.08 mmol) in dry DMF (4 mL). Reaction mixture was stirred at rt for 30 min. Reaction mixture was diluted with EtOAc and washed with $NaHCO_3$ sat. aq. solution (x3), 10% citric acid solution (x3), water (x1), 5% LiCl solution (x2) and brine. Organic phase was then dried with Na_2SO_4, filtered and concentrated. Crude was dissolved in a minimal amount of CH_2Cl_2 and precipitated in pentane (x3). Crude was then purified by flash column chromatography (BGB® Scorpius 40 g SiO_2 col., 40 mL/min, linear gradient 0 – 8% MeOH in CH_2Cl_2) to yield **60** as a colorless solid (1.03 g, 86%). R_f (CH_2Cl_2 + 19% MeOH): 0.5; Mp: 170.5 – 171.2 °C; $[\alpha]_D^{20}$ - 8.8 (*c* 0.50, MeOH); IR (neat): 3287 (m), 2931 (w), 1723 (m), 1686 (s), 1630 (s), 1522 (s), 1448 (m), 1365 (m), 1248 (s), 1156 (s), 737 (m), 620 (m); ^{1}H NMR (400 MHz, CD_3OD): 7.80 (d, $^3J_{H-H}$ = 7.4 Hz, 2H), 7.68 (d, $^3J_{H-H}$ = 7.4 Hz, 2H), 7.40 (t, $^3J_{H-H}$ = 7.4 Hz, 2H), 7.32 (t, $^3J_{H-H}$ = 7.4 Hz, 1H), 4.42 (dd, $^3J_{H-H}$ = 10.4, 7.1 Hz, 1H), 4.36 (dd, $^3J_{H-H}$ = 10.4, 7.1 Hz, 1H), 4.30 (dd, $^3J_{H-H}$ = 9.3, 5.2 Hz, 2H), 4.24 (t, $^3J_{H-H}$ = 6.9 Hz, 1H), 4.19 (dd, $^3J_{H-H}$ = 8.4, 6.0 Hz, 1H), 4.12 – 4.07 (m, 1H), 3.97 (dd, $^2J_{H-H}$ = 17.5, , $^4J_{H-H}$ = 2.6 Hz, 1H), 3.93 (dd, $^2J_{H-H}$ = 17.5, , $^4J_{H-H}$ = 2.6 Hz, 1H), 3.04 (t, $^3J_{H-H}$ = 6.8 Hz, 2H), 2.99 (t, $^3J_{H-H}$ = 6.8 Hz, 4H), 2.56 (t, $^4J_{H-H}$ = 2.6 Hz, 1H), 2.44 – 2.25 (m, 4H), 2.23 – 2.02 (m, 2H), 2.00 – 1.87 (m, 2H), 1.87 – 1.72 (m, 3H), 1.71 – 1.59 (m, 3H), 1.53 – 1.29 (m, 18H), 1.44

(s, 9H), 1.43 (s, 9H), 1.42 (s, 27H); ^{13}C NMR (101 MHz, CD_3OD): 175.6 (C), 174.6 (C), 174.1 (C), 173.8 (C), 173.7 (2xC), 173.7 (C), 159.0 (C), 158.6 (C), 158.4 (2xC), 145.3 (C), 145.2 (C), 142.6 (C), 142.6 (C), 128.9 (2xCH), 128.2 (2xCH), 126.3 (2xCH), 121.0 (2xCH), 81.9 (2xC), 81.9 (C), 80.6 (C), 79.9 (2xC), 72.3 (CH), 68.2 (CH_2), 57.1 (CH), 55.3 (CH), 54.5 (2xCH), 54.3 (CH), 48.4 (CH), 41.2 (CH_2), 41.2 (CH_2), 41.1 (CH_2), 32.7 (3xCH_2), 32.5 (2xCH_2), 32.2 (CH_2), 30.6 (CH_2), 30.5 (CH_2), 30.4 (CH_2), 29.6 (CH_2), 28.9 (9xCH_3), 28.4 (6xCH_3), 27.8 (2xCH_2), 24.2 (3xCH_2).

Compound 61 was prepared following the general procedure of Fmoc deprotection, starting from **60** (974 mg, 0.731 mmol) in 8 mL of 2 M dimethylamine in THF, yielding **61** as a colorless solid (778 mg, 98%).

Compound 62. To a solution of **61** (740 mg, 0.666 mmol) in dry DMF (4 mL), was added a 30 seconds-premixed solution of HATU (304 mg, 0.800 mmol), Fmoc-Glu(O*t*Bu)-OH (340 mg, 0.800 mmol) and DIPEA (139 µL, 0.800 mmol) in dry DMF (4 mL). Reaction mixture was stirred at rt for 30 min. Reaction mixture was diluted with EtOAc and washed with $NaHCO_3$ sat. aq. solution (x3), 10% citric acid solution (x3), water (x1), 5% LiCl solution (x2) and brine. Organic phase was then dried with Na_2SO_4, filtered and concentrated. Crude was dissolved in a minimal amount of CH_2Cl_2 and precipitated in pentane (x3). Crude was then purified by flash column chromatography (BGB® Scorpius 40 g SiO_2 col., 40 mL/min, linear gradient 0 – 8% MeOH in CH_2Cl_2) to yield **62** as a colorless solid (941 mg, 93%). R_f (CH_2Cl_2 + 10% MeOH): 0.5; Mp: 189.4 – 190.5 °C; $[\alpha]_D^{20}$ - 12.4 (*c* 0.50, MeOH); IR (neat): 3271 (m), 2927 (w), 1733 (s), 1628 (s), 1526 (s), 1448 (m), 1365 (s), 1228 (s), 1153 (s), 737 (m), 653 (m); ^{1}H NMR (400 MHz, CD_3OD): 7.80 (d, $^3J_{H-H}$ = 7.4 Hz, 2H), 7.66 (d, $^3J_{H-H}$ = 7.2 Hz, 2H), 7.39 (t, $^3J_{H-H}$ = 7.4 Hz, 2H), 7.31 (t, $^3J_{H-H}$ = 7.4 Hz, 2H), 4.46 (dd, $^3J_{H-H}$ = 10.6, 6.9 Hz, 1H), 4.38 (dd, $^3J_{H-H}$ = 10.6, 6.9 Hz, 1H), 4.34 – 4.19 (m, 4H), 4.23 (t, $^3J_{H-H}$ = 6.5 Hz, 1H), 4.11 (dd, $^3J_{H-H}$ = 8.8, 5.4 Hz, 1H), 3.96 (d, $^4J_{H-H}$ = 2.5 Hz, 2H), 3.09 – 2.94 (m, 6H), 2.53 (t, $^4J_{H-H}$ = 2.5

Hz, 1H), 2.44 – 2.24 (m, 6H), 2.21 – 2.01 (m, 3H), 2.01 – 1.87 (m, 3H), 1.86 – 1.78 (m, 3H), 1.77 – 1.62 (m, 3H), 1.52 – 1.38 (m, 12H), 1.45 (s, 9H), 1.44 (s, 9H), 1.42 (s, 18H), 1.41 (s, 9H), 1.41 (s, 9H); ^{13}C NMR (101 MHz, CD_3OD): 174.6 (C), 174.0 (C), 173.9 (2xC), 173.9 (C), 173.7 (2xC), 173.7 (2xC), 158.7 (C), 158.4 (2xC), 158.4 (C), 145.3 (C), 145.2 (C), 142.7 (2xC), 128.8 (2xCH), 128.2 (2xCH), 126.2 (CH), 126.2 (CH), 121.0 (2xCH), 81.9 (C), 81.9 (C), 81.9 (C), 80.6 (C), 79.9 (3xC), 72.3 (CH), 68.2 (CH_2), 56.4 (CH), 55.4 (CH), 54.8 (CH), 54.6 (2xCH), 54.5 (CH), 48.5 (CH), 41.3 (3xCH_2), 32.9 (CH_2), 32.8 (3xCH_2), 32.6 (CH_2), 32.3 (CH_2), 30.5 (2xCH_2), 30.5 (CH_2), 29.6 (CH_2), 28.9 (6xCH_3), 28.9 (3xCH_3), 28.5 (6xCH_3), 28.5 (3xCH_3), 28.1 (CH_2), 27.8 (2xCH_2), 24.3 (CH_2), 24.2 (2xCH_2).

Compound 63 was prepared following the general procedure of Fmoc deprotection, starting from **62** (870 mg, 0.573 mmol) in 8 mL of 2 M dimethylamine in THF, yielding **63** as a colorless solid (725 mg, 98%).

Compound 64. To a solution of **63** (670 mg, 0.517 mmol) in dry DMF (4 mL), was added a 30 seconds-premixed solution of HATU (236 mg, 0.620 mmol), **35** (201 mg, 0.620 mmol) and DIPEA (108 µL, 0.620 mmol) in dry DMF (4 mL). Reaction mixture was stirred at rt for 30 min. Reaction mixture was diluted with EtOAc and washed with $NaHCO_3$ sat. aq. solution (x3), 10% citric acid solution (x3), water (x1), 5% LiCl solution (x2) and brine. Organic phase was then dried with Na_2SO_4, filtered and concentrated. Crude was dissolved in a minimal amount of CH_2Cl_2 and precipitated in pentane (x3). Crude was then purified by flash column chromatography (BGB® Scorpius 25 g SiO_2 col., 40 mL/min, linear gradient 0 – 8% MeOH in CH_2Cl_2) to yield **64** as a colorless solid (744 mg, 90%). R_f (CH_2Cl_2 + 10% MeOH): 0.5; Mp: 181.7 – 182.2 °C; $[\alpha]_D^{20}$ + 0.6 (*c* 0.50, MeOH); IR (neat): 3296 (m), 2935 (m), 1692 (s), 1625 (s), 1515 (s), 1452 (m), 1367 (s), 1248 (s), 1151 (s), 847 (m), 645 (m); ^1H NMR (400 MHz, CD_3OD): 4.32 (dd, $^3J_{H\text{-}H}$ = 9.6, 4.8 Hz, 1H), 4.24 (dd, $^3J_{H\text{-}H}$ = 9.6, 5.2 Hz, 2H), 4.19 (dd, $^3J_{H\text{-}H}$ = 9.3, 5.2 Hz, 2H), 4.13 (dd, $^3J_{H\text{-}H}$ = 8.9, 5.2 Hz, 1H), 3.96 (d, $^4J_{H\text{-}H}$ = 2.5 Hz, 2H), 3.64

– 3.58 (m, 4H), 3.56 (t, $^3J_{H-H}$ = 6.6 Hz, 2H), 3.55 (t, $^3J_{H-H}$ = 5.6 Hz, 2H), 3.49 (t, $^3J_{H-H}$ = 6.5 Hz, 2H), 3.44 – 3.33 (m, 2H), 3.10 – 2.97 (m, 6H), 2.81 – 2.70 (m, 1H), 2.57 (t, $^4J_{H-H}$ = 2.5 Hz, 1H), 2.56 – 2.48 (m, 2H), 2.48 – 2.38 (m, 4H), 2.38 – 2.25 (m, 3H), 2.22 – 2.10 (m, 3H), 2.10 – 1.87 (m, 6H), 1.87 – 1.73 (m, 4H), 1.72 – 1.56 (m, 3H), 1.55 – 1.40 (m, 16H), 1.45 (s, 9H), 1.45 (s, 9H), 1.45 (s, 9H), 1.43 (s, 9H), 1.43 (s, 18H); ^{13}C NMR (101 MHz, CD_3OD): 176.6 (C), 176.1 (C), 175.8 (C), 174.9 (C), 174.7 (C), 174.5 (C), 174.0 (C), 173.8 (C), 173.7 (2xC), 173.4 (C), 158.4 (2xC), 158.4 (C), 81.9 (C), 81.8 (C), 81.7 (C), 80.6 (C), 79.8 (3xC), 72.3 (CH), 72.3 (CH_2), 71.3 (CH_2), 71.2 (CH_2), 70.6 (CH_2), 57.0 (CH), 56.5 (CH), 55.6 (CH), 55.2 (CH), 54.9 (CH), 54.5 (CH), 45.7 (CH_2), 41.3 (3xCH_2), 40.6 (CH_2), 33.8 (CH_2), 32.8 (CH_2), 32.8 (CH_2), 32.7 (CH_2), 32.5 (CH_2), 32.3 (CH_2), 32.2 (CH_2), 31.9 (CH_2), 31.3 (CH_2), 30.6 (CH_2), 30.5 (2xCH_2), 30.4 (CH_2), 29.6 (CH_2), 28.9 (9xCH_3), 28.5 (6xCH_3), 28.5 (3xCH_3), 27.7 (CH_2), 27.5 (CH_2), 27.5 (CH_2), 27.4 (CH_2), 26.5 (CH_2), 24.8 (CH_2), 24.5 (CH_2), 24.3 (CH_2).

Compound 65. To a solution of **64** (577 mg, 0.360 mmol) in THF/H_2O 2/1 (9 mL), **36** (135 mg, 0.432 mmol), $CuSO_4 \cdot 5H_2O$ (90 mg, 0.36 mmol), sodium ascorbate (143 mg, 0.721 mmol) and TBTA (19 mg, 0.0036 mmol) were added and the reaction mixture was stirred at rt for 1 h. Solvent was removed *in vacuo*, H_2O and CH_2Cl_2 were added, phases were separated, and aqueous phase was extracted by CH_2Cl_2 (x3). Organic phases were collected together, washed with brine (x1), dried over Na_2SO_4, filtered and concentrated. Crude was purified by flash column chromatography (BGB® Scorpius 25g, 25 mL/min, linear gradient 0 – 10% MeOH in CH_2Cl_2) to yield **65** as a colorless solid (595 mg, 86%). R_f (CH_2Cl_2 + 10% MeOH): 0.1; Mp: 184.2 – 184.8 °C; $[\alpha]_D^{20}$ + 13.7 (*c* 0.50, MeOH); IR (neat): 3300 (m), 2931 (m), 1692 (s), 1626 (s), 1518 (s), 1450 (m), 1365 (m), 1248 (s), 1151 (s); ^{1}H NMR (500 MHz, DMSO-d_6): 8.35 (t, $^3J_{H-H}$ = 5.7 Hz, NH), 8.22 (d, $^3J_{H-H}$ = 6.8 Hz, NH), 8.05 – 7.97 (m, 4xNH), 7.95 (t, $^3J_{H-H}$ = 5.7 Hz, NH), 7.83 (s, 1H), 7.76 (d, $^3J_{H-H}$ = 7.8 Hz, NH), 7.65 (d, $^3J_{H-H}$ = 7.3 Hz, NH), 6.74 – 6.65 (m, 2H), 6.42 (s, NH), 6.36 (s, NH), 4.37 (t, $^3J_{H-H}$ = 6.2 Hz, 2H), 4.33 – 4.27 (m,

3H), 4.27 – 4.21 (m, 1H), 4.21 – 4.10 (m, 6H), 3.61 (t, $^{3}J_{H-H}$ = 6.6 Hz, 2H), 3.51 – 3.43 (m, 6H), 3.41 – 3.35 (m, 4H), 3.20 – 3.16 (m, 2H), 3.09 (ddd, $^{3}J_{H-H}$ = 8.5, 6.3, 4.4 Hz, 1H), 2.90 – 2.82 (m, 6H), 2.82 (dd, $^{2}J_{H-H}$ = 12.5, $^{3}J_{H-H}$ = 5.1 Hz, 2H), 2.57 (d, $^{2}J_{H-H}$ = 12.5 Hz, 1H), 2.40 – 2.30 (m, 4H), 2.25 (t, $^{3}J_{H-H}$ = 8.0 Hz, 2H), 2.23 – 2.12 (m, 4H), 2.04 (t, $^{3}J_{H-H}$ = 7.1 Hz, 2H), 1.97 – 1.84 (m, 3H), 1.78 – 1.56 (m, 10H), 1.53 – 1.43 (m, 7H), 1.40 – 1.26 (m, 20H), 1.38 (s, 9H), 1.37 (s, 9H), 1.37 (s, 9H), 1.36 (s, 27H); ^{13}C NMR (126 MHz, DMSO-d_6): 172.7 (C), 172.5 (C), 171.9 (C), 171.9 (C), 171.8 (C), 171.6 (C), 171.6 (C), 171.6 (C), 171.5 (C), 171.3 (C), 171.0 (C), 170.8 (C), 162.7 (C), 155.5 (2xC), 155.5 (C), 144.6 (C), 122.9 (CH), 79.6 (C), 79.6 (C), 79.6 (C), 77.3 (3xC), 70.2 (CH_2), 69.6 (CH_2), 69.4 (CH_2), 69.0 (CH_2), 61.0 (CH), 59.2 (CH), 55.4 (CH_2), 53.2 (CH), 53.0 (CH), 52.7 (CH), 52.4 (CH), 51.9 (CH), 51.8 (CH), 48.6 (CH), 45.3 (CH_2), 39.8 (2xCH_2), 39.8 (CH_2), 39.8 (CH_2), 38.9 (CH_2), 38.6 (CH_2), 35.0 (CH_2), 34.3 (CH_2), 32.0 (CH_2), 31.6 (CH_2), 31.5 (CH_2), 31.3 (CH_2), 31.2 (CH_2), 31.0 (CH_2), 30.8 (CH_2), 30.7 (CH_2), 30.5 (CH_2), 29.2 (CH_2), 29.2 (CH_2), 29.1 (CH_2), 29.0 (CH_2), 28.3 (9xCH_3), 28.1 (CH_2), 28.0 (CH_2), 27.7 (3xCH_3), 27.7 (6xCH_3), 27.3 (CH_2), 26.8 (CH_2), 26.8 (CH_2), 26.1 (CH_2), 25.1 (CH_2), 24.9 (CH_2), 23.0 (CH_2), 22.7 (CH_2), 22.6 (CH_2).

Compound 66. A solution of **65** (150 mg, 78.4 µmol) in TFA/H_2O/TIPS 95/2.5/2.5 (2 mL) was stirred at rt for 30 min. Solvent was removed *in vacuo*, crude was dissolved in small amount of MeOH and was precipitated in Et_2O (x3) to yield **66** as a colorless TFA salt (128 mg, 91%). Crude was used in the next reaction without further purification.

Compound 6. To a solution of **101** (40 mg, 22 µmol) in DMF (800 µL), **37** (22 mg, 89 µmol) and DIPEA (31 µL, 178 µmol) were added and reaction mixture was stirred at rt for 1 h. Crude reaction mixture was purified by RP flash chromatography (Biotage® SNAP Ultra 12 g C18, 12 mL/min, linear gradient 20 – 50% CH_3CN in H_2O with 0.1% TFA) to yield **6** as a colorless solid (7 mg, 17%). ^{1}H NMR (500 MHz, DMSO-d_6/D_2O 60/1): 7.81 (s, 1H), 4.37 (t, $^{3}J_{H-H}$ = 6.2 Hz, 2H), 4.33 – 4.26 (m, 3H), 4.23 (dd, $^{3}J_{H-H}$ = 8.5, 5.5 Hz, 1H), 4.21 – 4.10 (m,

6H), 3.60 (t, $^3J_{H-H}$ = 6.6 Hz, 2H), 3.46 – 3.40 (m, 6H) 3.39 – 3.32 (m, 10H), 3.20 – 3.12 (m, 8H), 3.12 – 3.07 (m, 4H), 3.06 – 2.96 (m, 6H), 2.82 (dd, $^2J_{H-H}$ = 12.5, $^3J_{H-H}$ = 5.1 Hz, 1H), 2.58 (d, $^2J_{H-H}$ = 12.5 Hz, 1H), 2.40 – 2.31 (m, 4H), 2.28 (t, $^3J_{H-H}$ = 8.0 Hz, 2H), 2.24 – 2.16 (m, 4H), 2.03 (t, $^3J_{H-H}$ = 7.2 Hz, 2H), 1.96 – 1.86 (m, 3H), 1.80 – 1.57 (m, 10H), 1.56 – 1.42 (m, 7H), 1.41 – 1.33 (m, 8H), 1.32 – 1.19 (m, 10H); ^{13}C NMR (126 MHz, DMSO-d_6): 173.9 (C), 173.9 (C), 173.8 (C), 172.6 (C), 172.5 (C), 172.0 (C), 171.9 (C), 171.8 (C), 171.7 (C), 171.4 (C), 171.1 (C), 170.9 (C), 170.2 (C), 170.2 (2xC), 162.7 (C), 144.6 (C), 123.0 (CH), 70.2 (CH_2), 69.6 (CH_2), 69.4 (CH_2), 69.0 (CH_2), 61.0 (CH), 59.2 (CH), 55.4 (CH), 53.1 (CH), 52.9 (CH), 52.7 (CH), 52.3 (CH), 52.1 (CH), 51.9 (CH), 51.5 (CH), 51.5 (2xCH), 48.7 (CH_2), 45.4 (CH_2), 42.1 (6xCH_2), 39.8 (CH_2), 38.9 (CH_2), 38.8 (2xCH_2), 38.7 (CH_2), 38.6 (CH_2), 35.1 (CH_2), 34.3 (CH_2), 32.0 (CH_2), 31.5 (CH_2), 31.4 (CH_2), 30.8 (CH_2), 30.7 (CH_2), 30.5 (CH_2), 30.2 (CH_2), 30.1 (CH_2), 30.0 (CH_2), 29.1 (CH_2), 28.6 (2xCH_2), 28.4 (CH_2), 28.1 (CH_2), 28.0 (CH_2), 27.3 (CH_2), 26.8 (CH_2), 26.8 (CH_2), 26.1 (CH_2), 25.1 (CH_2), 24.9 (CH_2), 23.0 (CH_2), 22.7 (2xCH_2); HRMS (ESI, +ve) calcd for $C_{74}H_{118}ClN_{17}O_{21}S_7$ ([M + H]$^+$): 1840.6495, found: 1840.6527; LC-MS (ESI, B30): R_t 1.62 min, 1842 (50, [M+H]$^+$), 921 (100, [M+2H]$^{2+}$).

2.1.3. Synthesis of peptide 7

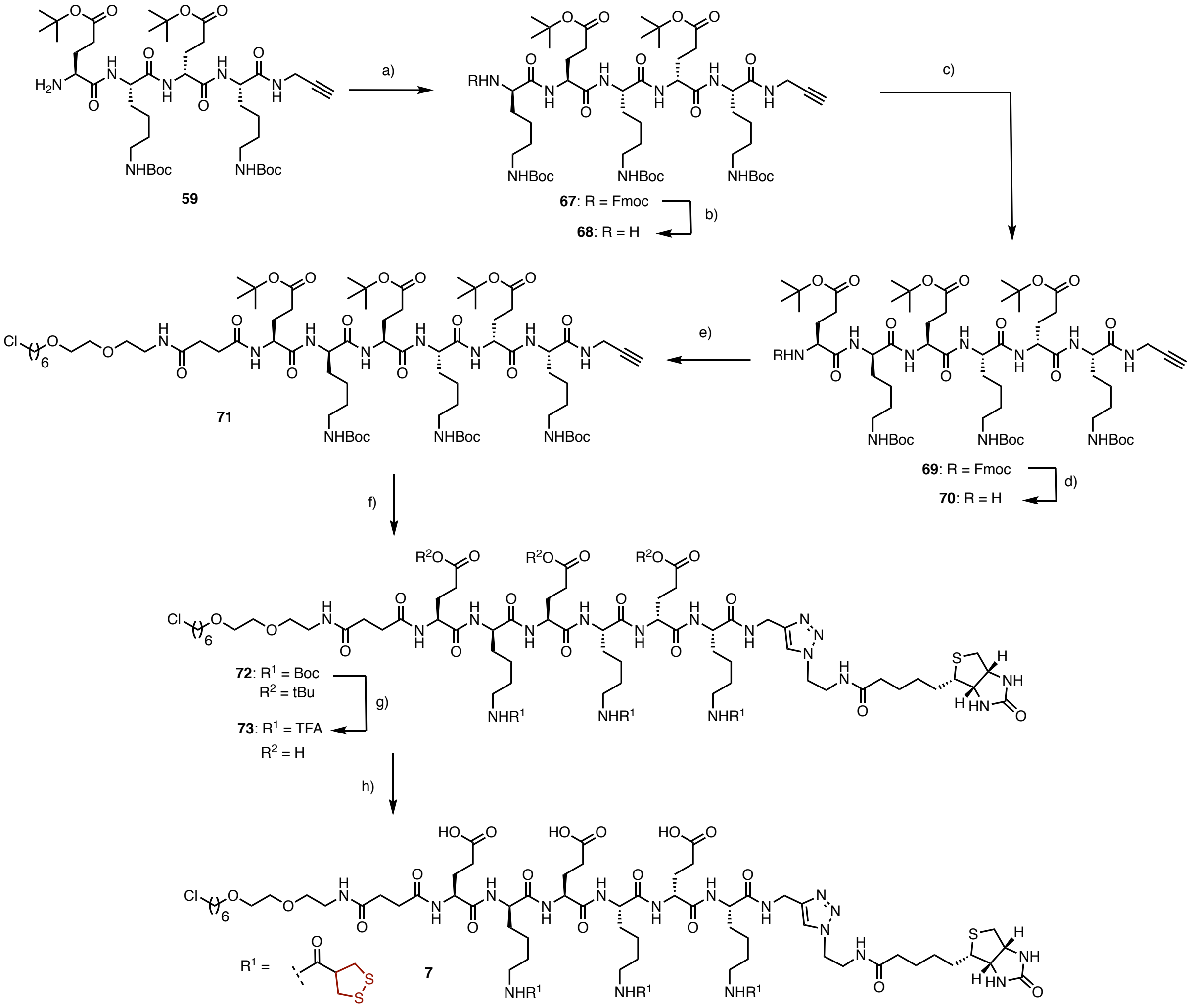

Scheme S3 (a) Fmoc-D-Lys(Boc)-OH, HATU, DIPEA, DMF, rt, 30 min, 91%; (b) 2 M dimethylamine in THF, rt, 30 min, 100%; (c) Fmoc-Glu(O*t*Bu)-OH, HATU, DIPEA, DMF, rt, 30 min, 78%; (d) 2 M dimethylamine in THF, rt, 30 min, 84%; (e) **35**, HATU, DIPEA, DMF, rt, 30 min, 82%; (f) **36**, $CuSO_4 \cdot 5H_2O$, Na-ascorbate, TBTA, THF/H_2O, rt, 1 h, 75%; (g) TFA/TIPS/H_2O 95/2.5/2.5, rt, 30 min, 100%; (h) **37**, DIPEA, DMF, rt, 1 h, 22%.

Compound 67. To a solution of **59** (800 mg, 0.907 mmol) in dry DMF (4 mL), was added a 30 seconds-premixed solution of HATU (414 mg, 1.09 mmol), Fmoc-D-Lys(Boc)-OH (510 mg, 1.09 mmol) and DIPEA (190 µL, 1.09 mmol) in dry DMF (4 mL). Reaction mixture

was stirred at rt for 30 min. Reaction mixture was diluted with EtOAc and washed with $NaHCO_3$ sat. aq. solution (x3), 10% citric acid solution (x3), water (x1), 5% LiCl solution (x2) and brine. Organic phase was then dried with Na_2SO_4, filtered and concentrated. Crude was dissolved in a minimal amount of CH_2Cl_2 and precipitated in pentane (x3). Crude was then purified by flash column chromatography (BGB® Scorpius 25 g SiO_2 col., 40 mL/min, linear gradient 0 – 8% MeOH in CH_2Cl_2) to yield **67** as a colorless solid (1.10 g, 91%). R_f (CH_2Cl_2 + 10% MeOH): 0.5; Mp: 167.0 – 167.4 °C; $[\alpha]_D^{20}$ – 7.8 (*c* 0.50, MeOH); IR (neat): 3291 (m), 2927 (m), 1639 (s), 1627 (s), 1522 (s), 1452 (m), 1248 (s), 1169 (s), 1012 (m), 655 (m), 551 (m); ^{1}H NMR (400 MHz, CD_3OD): 7.79 (d, $^3J_{H\text{-}H}$ = 7.4 Hz, 2H), 7.70 (d, $^3J_{H\text{-}H}$ = 7.4 Hz, 1H), 7.66 (d, $^3J_{H\text{-}H}$ = 7.4 Hz, 1H), 7.39 (t, $^3J_{H\text{-}H}$ = 7.4 Hz, 2H), 7.31 (t, $^3J_{H\text{-}H}$ = 7.4 Hz, 2H), 4.45 (dd, $^3J_{H\text{-}H}$ = 10.3, 6.5 Hz, 1H), 4.39 – 4.21 (m, 5H), 4.13 (dd, $^3J_{H\text{-}H}$ = 8.9, 5.9 Hz, 1H), 4.02 (t, $^3J_{H\text{-}H}$ = 7.2 Hz, 1H), 3.93 (d, $^4J_{H\text{-}H}$ = 2.5 Hz, 2H), 3.09 – 2.93 (m, 6H), 2.52 (t, $^4J_{H\text{-}H}$ = 2.5 Hz, 1H), 2.42 – 2.26 (m, 4H), 2.26 – 2.07 (m, 2H), 2.01 – 1.62 (m, 8H), 1.55 – 1.30 (m, 12H), 1.43 (s, 27H), 1.41 (s, 9H), 1.40 (s, 9H); ^{13}C NMR (101 MHz, CD_3OD): 175.9 (C), 174.9 (C), 174.0 (C), 173.9 (C), 173.8 (C), 173.7 (C), 173.7 (C), 158.8 (C), 158.6 (C), 158.4 (2xC), 145.6 (C), 145.1 (C), 142.6 (C), 142.6 (C), 128.8 (CH), 128.8 (CH), 128.3 (CH), 128.2 (CH), 126.4 (CH), 126.3 (CH), 120.9 (2xCH), 81.9 (C), 81.9 (C), 80.6 (C), 79.9 (C), 79.9 (C), 79.9 (C), 72.2 (CH), 68.4 (CH_2), 56.9 (CH), 55.9 (CH), 54.7 (CH), 54.5 (CH), 54.3 (CH), 48.4 (CH), 41.2 (CH_2), 41.1 (2xCH_2), 32.7 (2xCH_2), 32.4 (CH_2), 32.3 (CH_2), 31.8 (CH_2), 30.6 (CH_2), 30.5 (CH_2), 30.4 (CH_2), 29.6 (CH_2), 28.8 (9xCH_3), 28.4 (6xCH_3), 27.6 (CH_2), 27.6 (CH_2), 24.5 (CH_2), 24.2 (2xCH_2).

Compound 68 was prepared following the general procedure of Fmoc deprotection, starting from **67** (1.0 g, 0.750 mmol) in 8 mL of 2 M dimethylamine in THF, yielding **68** as a colorless solid (830 mg, 100%).

Compound 69. To a solution of **68** (805 mg, 0.725 mmol) in dry DMF (4 mL), was added a 30 seconds-premixed solution of HATU (331 mg, 0.870 mmol), Fmoc-Glu(O*t*Bu)-OH (370 mg, 0.870 mmol) and DIPEA (152 µL, 0.870 mmol) in dry DMF (4 mL). Reaction mixture was stirred at rt for 30 min. Reaction mixture was diluted with EtOAc and washed with $NaHCO_3$ sat. aq. solution (x3), 10% citric acid solution (x3), water (x1), 5% LiCl solution (x2) and brine. Organic phase was then dried with Na_2SO_4, filtered and concentrated. Crude was dissolved in a minimal amount of CH_2Cl_2 and precipitated in pentane (x3). Crude was then purified by flash column chromatography (BGB® Scorpius 25 g SiO_2 col., 40 mL/min, linear gradient 0 – 8% MeOH in CH_2Cl_2) to yield **69** as a colorless solid (862 mg, 78%). R_f (CH_2Cl_2 + 10% MeOH): 0.5; Mp: 187.3 – 188.2 °C; $[\alpha]_D^{20}$ – 4.8 (*c* 0.50, MeOH); IR (neat): 3283 (m), 2935 (w), 1712 (m), 1688 (s), 1624 (s), 1522 (s), 1454 (m), 1365 (m), 1246 (s), 1151 (s), 737 (m), 640 (m); ^{1}H NMR (500 MHz, CD_3OD): 7.80 (d, $^3J_{H\text{-}H}$ = 7.4 Hz, 2H), 7.68 (d, $^3J_{H\text{-}H}$ = 7.4 Hz, 2H), 7.39 (t, $^3J_{H\text{-}H}$ = 7.4 Hz, 2H), 7.32 (t, $^3J_{H\text{-}H}$ = 7.4 Hz, 2H), 4.40 (dd, $^3J_{H\text{-}H}$ = 10.5, 7.4 Hz, 1H), 4.36 (dd, $^3J_{H\text{-}H}$ = 10.5, 7.4 Hz, 1H), 4.32 – 4.21 (m, 6H), 4.19 – 4.10 (m, 2H), 3.99 – 3.90 (m, 2H), 3.10 – 2.92 (m, 6H), 2.54 (t, $^4J_{H\text{-}H}$ = 2.5 Hz, 1H), 2.43 – 2.24 (m, 6H), 2.21 – 2.01 (m, 3H), 2.00 – 1.75 (m, 7H), 1.73 – 1.64 (m, 2H), 1.55 – 1.30 (m, 12H), 1.45 (s, 18H), 1.42 (s, 18H), 1.41 (s, 9H), 1.40 (s, 9H); ^{13}C NMR (126 MHz, CD_3OD): 174.9 (C), 174.8 (C), 174.7 (C), 174.1 (C), 173.9 (C), 173.9 (C), 173.8 (C), 173.7 (C), 173.7 (C), 158.6 (2xC), 158.5 (C), 158.4 (C), 145.3 (C), 145.2 (C), 142.6 (C), 142.6 (C), 128.9 (2xCH), 128.3 (CH), 128.3 (CH), 126.4 (CH), 126.3 (CH), 121.0 (2xCH), 81.9 (C), 81.8 (C), 81.8 (C), 80.6 (C), 79.8 (3xC), 72.3 (CH), 68.3 (CH_2), 55.8 (CH), 55.5 (CH), 55.2 (CH), 54.8 (CH), 54.8 (CH), 54.5 (CH), 48.4 (CH), 41.2 (3xCH_2), 32.7 (CH_2), 32.7 (CH_2), 32.6 (CH_2), 32.4 (CH_2), 31.9 (CH_2), 31.7 (CH_2), 30.6 (CH_2), 30.5 (CH_2), 30.4 (CH_2), 29.6 (CH_2), 28.9 (3xCH_3), 28.8 (6xCH_3), 28.4 (3xCH_3), 28.4 (6xCH_3), 27.9 (CH_2), 27.8 (CH_2), 27.5 (CH_2), 24.5 (CH_2), 24.3 (2xCH_2).

Compound 70 was prepared following the general procedure of Fmoc deprotection, starting from **69** (800 mg, 0.527 mmol) in 8 mL of 2 M dimethylamine in THF, yielding **70** as a colorless solid (573 mg, 84%).

Compound 71. To a solution of **70** (500 mg, 0.386 mmol) in dry DMF (3 mL), was added a 30 seconds-premixed solution of HATU (176 mg, 0.463 mmol), **35** (150 mg, 0.463 mmol) and DIPEA (81 µL, 0.46 mmol) in dry DMF (3 mL). Reaction mixture was stirred at rt for 30 min. Reaction mixture was diluted with EtOAc and washed with $NaHCO_3$ sat. aq. solution (x3), 10% citric acid solution (x3), water (x1), 5% LiCl solution (x2) and brine. Organic phase was then dried with Na_2SO_4, filtered and concentrated. Crude was dissolved in a minimal amount of CH_2Cl_2 and precipitated in pentane (x3). Crude was then purified by flash column chromatography (BGB® Scorpius 25 g SiO_2 col., 40 mL/min, linear gradient 0 – 8% MeOH in CH_2Cl_2) to yield **71** as a colorless solid (505 mg, 82%). R_f (CH_2Cl_2 + 10% MeOH): 0.5; Mp: 211.2 – 212.1 °C; $[\alpha]_D^{20}$ – 15.6 (*c* 0.50, MeOH); IR (neat): 3279 (m), 2935 (m), 1723 (s), 1690 (s), 1623 (s), 1520 (s), 1450 (m), 1365 (s), 1233 (m), 1150 (s), 704 (m); ^{1}H NMR (500 MHz, CD_3OD): 4.35 – 4.27 (m, 3H), 4.27 – 4.24 (m, 1H), 4.18 (dd, $^3J_{H-H}$ = 9.2, 5.7 Hz, 1H), 4.14 (dd, $^3J_{H-H}$ = 8.4, 6.5 Hz, 1H), 3.97 (d, $^4J_{H-H}$ = 2.5 Hz, 2H), 3.62 – 3.57 (m, 4H), 3.56 (t, $^3J_{H-H}$ = 6.6 Hz, 2H), 3.53 (t, $^3J_{H-H}$ = 5.6 Hz, 2H), 3.49 (t, $^3J_{H-H}$ = 6.6 Hz, 2H), 3.36 (t, $^3J_{H-H}$ = 5.6 Hz, 2H), 3.10 – 2.98 (m, 6H), 2.58 (t, $^4J_{H-H}$ = 2.5 Hz, 1H), 2.57 – 2.47 (m, 4H), 2.42 – 2.25 (m, 6H), 2.23 – 2.06 (m, 3H), 2.02 – 1.82 (m, 7H), 1.81 – 1.66 (m, 4H), 1.66 – 1.57 (m, 2H), 1.53 – 1.37 (m, 14H), 1.45 (s, 9H), 1.45 (s, 18H), 1.43 (s, 27H); ^{13}C NMR (126 MHz, CD_3OD): 175.4 (C), 175.2 (C), 174.9 (C), 174.6 (C), 174.5 (C), 174.2 (C), 173.9 (2xC), 173.8 (C), 173.8 (C), 173.7 (C), 173.7 (C), 158.5 (C), 158.4 (2xC), 81.9 (C), 81.8 (C), 81.8 (C), 80.7 (C), 79.8 (3xC), 72.4 (CH_2), 72.3 (CH_2), 71.3 (CH_2), 71.2 (CH_2), 70.7 (CH), 55.8 (CH), 55.6 (CH), 54.7 (2xCH), 54.4 (CH), 54.2 (CH), 45.7 (CH_2), 41.2 (CH_2), 41.2 (CH_2), 41.2 (CH_2), 40.5 (CH_2), 33.8 (CH_2), 32.8 (CH_2), 32.7 (CH_2), 32.6 (CH_2), 32.4 (CH_2), 32.3 (CH_2), 31.9

(CH_2), 31.8 (CH_2), 31.4 (CH_2), 30.6 (CH_2), 30.6 (CH_2), 30.5 (CH_2), 30.4 (CH_2), 29.6 (CH_2), 28.9 (9xCH_3), 28.5 (9xCH_3), 27.8 (CH_2), 27.8 (CH_2), 27.5 (2xCH_2), 26.5 (CH_2), 24.6 (CH_2), 24.4 (CH_2), 24.3 (CH_2).

Compound 72. To a solution of **71** (400 mg, 0.250 mmol) in THF/H_2O 2/1 (9 mL), **36** (93 mg, 0.30 mmol), $CuSO_4 \cdot 5H_2O$ (62 mg, 0.25 mmol), sodium ascorbate (99 mg, 0.50 mmol) and TBTA (13 mg, 25 µmol) were added and the reaction mixture was stirred at rt for 1 h. Solvent was removed *in vacuo*, H_2O and CH_2Cl_2 were added, phases were separated, and aqueous phase was extracted by CH_2Cl_2 (x3). Organic phases were collected together, washed with brine (x1), dried over Na_2SO_4, filtered and concentrated. Crude was purified by flash column chromatography (BGB® Scorpius 25g, 25 mL/min, linear gradient 0 – 10% MeOH in CH_2Cl_2) to yield **72** as a colorless solid (361 mg, 75%). R_f (CH_2Cl_2 + 10% MeOH): 0.1; Mp: 197.2 – 197.9 °C; $[\alpha]_D^{20}$ + 0.06 (*c* 0.50, MeOH); IR (neat): 3279 (w), 2927 (w), 1690 (s), 1622 (s), 1520 (s), 1452 (m), 1365 (m), 1250 (s), 1151 (s), 694 (m), 551 (m); ^{1}H NMR (500 MHz, DMSO-d_6): 8.38 (t, $^3J_{H-H}$ = 5.7 Hz, NH), 8.11 (d, $^3J_{H-H}$ = 7.3 Hz, NH), 8.09 – 8.02 (m, 4xNH), 7.94 (t, $^3J_{H-H}$ = 5.7 Hz, NH), 7.91 – 7.87 (m, 2xNH), 7.83 (s, 1H), 6.75 – 6.67 (m, 2xNH), 6.41 (s, NH), 6.35 (s, NH), 4.36 (t, $^3J_{H-H}$ = 6.2 Hz, 2H), 4.32 – 4.26 (m, 4H), 4.25 – 4.07 (m, 6H), 3.60 (t, $^3J_{H-H}$ = 6.6 Hz, 2H), 3.51 – 3.42 (m, 6H), 3.38 (t, $^3J_{H-H}$ = 6.1 Hz, 2H), 3.35 (t, $^3J_{H-H}$ = 6.6 Hz, 2H), 3.20 – 3.14 (m, 2H), 3.08 (ddd, $^3J_{H-H}$ = 8.4, 6.3, 4.4 Hz, 1H), 2.88 – 2.78 (m, 7H), 2.56 (d, $^2J_{H-H}$ = 12.4 Hz, 1H), 2.37 – 2.27 (m, 4H), 2.23 – 2.11 (m, 6H), 2.03 (t, $^3J_{H-H}$ = 7.1 Hz, 2H), 1.96 – 1.81 (m, 3H), 1.75 – 1.65 (m, 5H), 1.65 – 1.51 (m, 6H), 1.51 – 1.43 (m, 6H), 1.41 – 1.25 (m, 20H), 1.37 (s, 9H), 1.36 (s, 9H), 1.35 (s, 9H), 1.35 (s, 27H); ^{13}C NMR (126 MHz, DMSO-d_6): 172.5 (C), 171.9 (C), 171.7 (C), 171.7 (C), 171.6 (C), 171.6 (C), 171.6 (C), 171.5 (C), 171.4 (C), 171.3 (C), 171.0 (C), 170.8 (C), 162.7 (C), 155.5 (3xC), 144.6 (C), 123.0 (CH), 79.7 (C), 79.6 (2xC), 77.3 (C), 77.3 (2xC), 70.2 (CH_2), 69.6 (CH_2), 69.4 (CH_2), 69.1 (CH_2), 61.0 (CH), 59.2 (CH), 55.4 (CH_2), 53.0 (CH), 52.9 (CH), 52.4 (CH), 52.1 (CH), 51.7 (CH),

51.6 (CH), 48.6 (CH), 45.3 (CH_2), 39.8 (CH_2), 39.8 (3xCH_2), 38.9 (CH_2), 38.6 (CH_2), 35.0 (CH_2), 34.3 (CH_2), 32.0 (CH_2), 31.6 (3xCH_2), 31.2 (CH_2), 31.2 (CH_2), 31.0 (CH_2), 30.7 (CH_2), 30.6 (CH_2), 29.2 (CH_2), 29.1 (2xCH_2), 29.0 (CH_2), 28.3 (9xCH_3), 28.1 (CH_2), 28.0 (CH_2), 27.7 (3xCH_3), 27.7 (6xCH_3), 27.5 (CH_2), 27.1 (CH_2), 26.9 (CH_2), 26.1 (CH_2), 25.1 (CH_2), 24.9 (CH_2), 22.7 (CH_2), 22.6 (2xCH_2).

Compound 73. A solution of **72** (150 mg, 78.4 μmol) in TFA/H_2O/TIPS 95/2.5/2.5 (2 mL) was stirred at rt for 30 min. Solvent was removed *in vacuo*, crude was dissolved in small amount of MeOH and was precipitated in Et_2O (x3) to yield **73** as a colorless TFA salt (140 mg, 100%). Crude was used in the next reaction without further purification.

Compound 7. To a solution of **73** (40 mg, 22 μmol) in DMF (800 μL), 37 (22 mg, 89 μmol) and DIPEA (31 μL, 178 μmol) were added and reaction mixture was stirred at rt for 1 h. Crude reaction mixture was purified by RP flash chromatography (Biotage® SNAP Ultra 12 g C18, 12 mL/min, linear gradient 20 – 50% CH_3CN in H_2O with 0.1% TFA) to yield **7** as a colorless solid (9 mg, 22%). ^1H NMR (500 MHz, DMSO-d_6/D_2O 60/1): 7.82 (s, 1H), 4.37 (t, $^3J_{H-H}$ = 6.2 Hz, 2H), 4.33 – 4.24 (m, 5H), 4.23 – 4.12 (m, 5H), 3.60 (t, $^3J_{H-H}$ = 6.6 Hz, 2H), 3.55 – 3.49 (m, 6H), 3.39 – 3.34 (m, 10H), 3.20 – 3.14 (m, 8H), 3.13 – 3.07 (m, 4H), 3.06 – 2.96 (m, 6H), 2.82 (dd, $^2J_{H-H}$ = 12.5, $^3J_{H-H}$ = 5.1 Hz, 1H), 2.58 (d, $^2J_{H-H}$ = 12.5 Hz, 1H), 2.38 – 2.29 (m, 4H), 2.27 – 2.15 (m, 6H), 2.03 (t, $^3J_{H-H}$ = 7.8 Hz, 2H), 1.98 – 1.84 (m, 3H), 1.80 – 1.51 (m, 11H), 1.51 – 1.43 (m, 6H), 1.42 – 1.33 (m, 8H), 1.32 – 1.17 (m, 10H); ^{13}C NMR (126 MHz, DMSO-d_6): 173.9 (C), 173.9 (C), 173.8 (C), 172.6 (C), 172.0 (C), 171.8 (2xC), 171.6 (C), 171.5 (C), 171.4 (C), 171.1 (C), 170.9 (C), 170.2 (C), 170.2 (C), 170.2 (C), 162.7 (C), 144.6 (C), 123.0 (CH), 70.2 (CH_2), 69.6 (CH_2), 69.4 (CH_2), 69.1 (CH_2), 61.0 (CH), 59.2 (CH), 55.4 (CH), 52.9 (CH), 52.7 (CH), 52.3 (CH), 52.3 (CH), 51.9 (CH), 51.7 (CH), 51.5 (3xCH), 48.7 (CH_2), 45.4 (CH_2), 42.1 (6xCH_2), 39.8 (CH_2), 38.9 (CH_2), 38.7 (2xCH_2), 38.7 (CH_2), 38.6 (CH_2), 35.1 (CH_2), 34.3 (CH_2), 32.0 (CH_2), 31.5 (2xCH_2), 31.3 (CH_2), 30.6 (CH_2), 30.6 (CH_2),

30.1 (CH_2), 30.1 (CH_2), 30.0 (CH_2), 29.1 (CH_2), 28.6 (CH_2), 28.5 (CH_2), 28.4 (CH_2), 28.1 (CH_2), 28.0 (CH_2), 27.4 (CH_2), 27.1 (CH_2), 26.9 (CH_2), 26.1 (CH_2), 25.1 (CH_2), 24.9 (CH_2), 22.8 (CH_2), 22.7 (CH_2), 22.7 (CH_2); HRMS (ESI, +ve) calcd for $C_{74}H_{118}ClN_{17}O_{21}S_7$ ($[M + H]^+$): 1840.6495, found: 1840.6527; LC-MS (ESI, B30): R_t 1.59 min, 1842 (50, $[M+H]^+$), 921 (100, $[M+2H]^{2+}$).

2.1.4. Synthesis of peptide 8

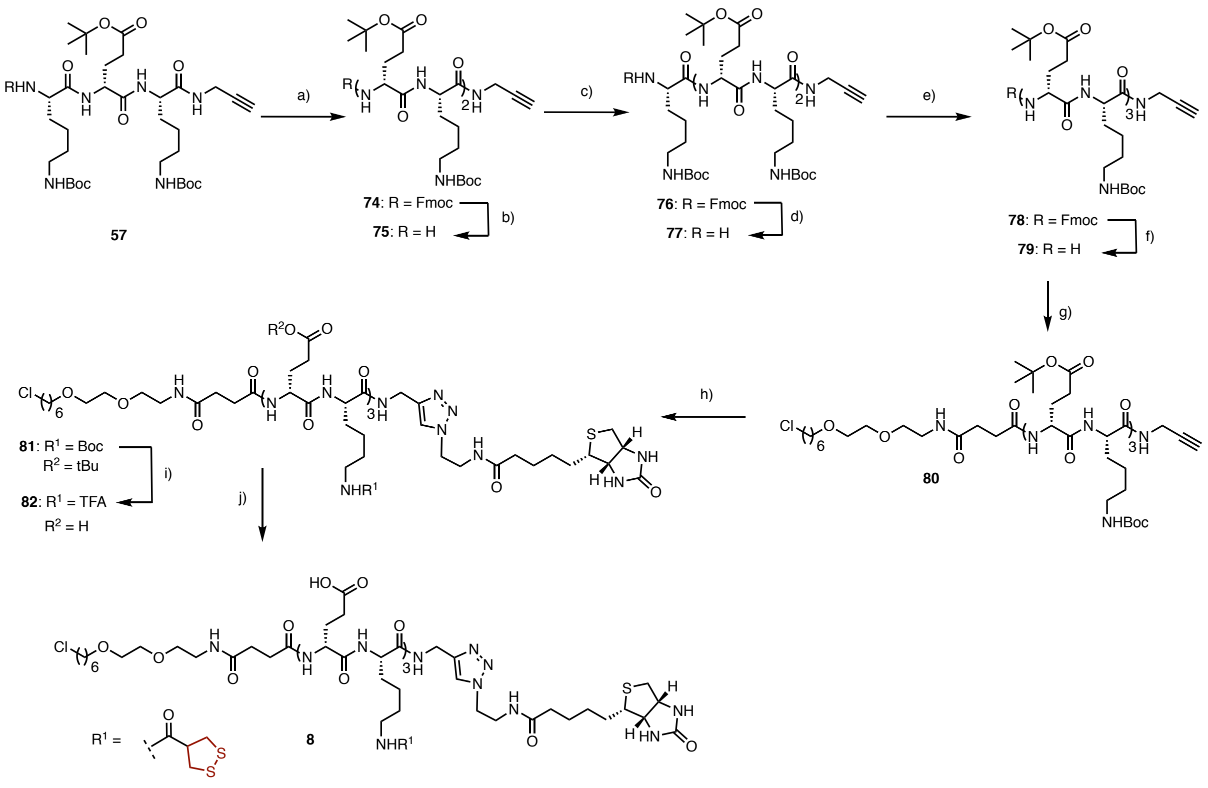

Scheme S4 (a) Fmoc-D-Glu(O*t*Bu)-OH, HATU, DIPEA, DMF, rt, 1 h, 91%; (b) 2 M dimethylamine in THF, rt, 30 min, 100%; (c) Fmoc-Lys(Boc)-OH, HATU, DIPEA, DMF, rt,1 h, 79%; (d) 2 M dimethylamine in THF, rt, 30 min, 100%; (e) Fmoc-D-Glu(O*t*Bu)-OH, HATU, DIPEA, DMF, rt, 1 h, 87%; (f) 2 M dimethylamine in THF, rt, 30 min, 99%; (g) **35**, HATU, DIPEA, DMF, rt, 3 h, 89%; (h) **36**, $CuSO_4 \cdot 5H_2O$, Na-ascorbate, TBTA, THF/H_2O, rt, 1 h, 80%; (i) TFA/TIPS/H_2O 95/2.5/2.5, rt, 30 min, 86%; (j) **37**, DIPEA, DMF, rt, 1 h, 38%.

Compound 74. To a solution of **57** (2.3 g, 3.3 mmol) in dry DMF (10 mL), was added a 30 seconds-premixed solution of HATU (1.5 g, 4.0 mmol), Fmoc-D-Glu(O*t*Bu)-OH (1.7 g, 4.0 mmol) and DIPEA (700 µL, 4.0 mmol) in dry DMF (10 mL). Reaction mixture was stirred at rt for 30 min. Reaction mixture was diluted with EtOAc and washed with $NaHCO_3$ sat. aq. solution (x3), 10% citric acid solution (x3), water (x1), 5% LiCl solution (x2) and brine. Organic phase was then dried with Na_2SO_4, filtered and concentrated. Crude was dissolved in a minimal amount of CH_2Cl_2 and precipitated in pentane (x3). Crude was then purified by flash column chromatography (BGB® Scorpius 80 g SiO_2 col., 40 mL/min, linear gradient 0 – 8% MeOH in CH_2Cl_2) to yield **74** as a colorless solid (3.3 g, 91%). R_f (CH_2Cl_2 + 10% MeOH): 0.5; Mp: 167.5 – 168.2 °C; $[\alpha]_D^{20}$ – 6.0 (*c* 0.50, MeOH); IR (neat): 3281 (w), 2935 (w), 1727 (m), 1683 (s), 1633 (s), 1521 (s), 1448 (m), 1366 (m), 1250 (m), 1154 (s), 737 (m), 624 (m); ^{1}H NMR (400 MHz, CD_3OD): 7.79 (d, $^3J_{H\text{-}H}$ = 7.5 Hz, 2H), 7.67 (d, $^3J_{H\text{-}H}$ = 7.4 Hz, 2H), 7.39 (t, $^3J_{H\text{-}H}$ = 7.4 Hz, 2H), 7.31 (t, $^3J_{H\text{-}H}$ = 7.5 Hz, 2H), 4.47 (dd, $^3J_{H\text{-}H}$ = 10.6, 6.8 Hz, 1H), 4.37 (dd, $^3J_{H\text{-}H}$ = 10.6, 6.8 Hz, 1H), 4.31 – 4.18 (m, 4H), 4.08 (dd, $^3J_{H\text{-}H}$ = 8.3, 6.0 Hz, 1H), 4.00 (dd, $^2J_{H\text{-}H}$ = 17.5, $^4J_{H\text{-}H}$ = 2.5 Hz, 1H), 3.93 (dd, $^2J_{H\text{-}H}$ = 17.5, $^4J_{H\text{-}H}$ = 2.5 Hz, 1H), 3.11 – 2.91 (m, 4H), 2.54 (t, $^4J_{H\text{-}H}$ = 2.5 Hz, 1H), 2.32 (t, $^3J_{H\text{-}H}$ = 7.5 Hz, 2H), 2.31 – 2.19 (m, 2H), 2.16 – 1.96 (m, 2H), 1.99 – 1.77 (m, 4H), 1.77 – 1.59 (m, 2H), 1.55 – 1.32 (m, 8H), 1.45 (s, 9H), 1.42 (s, 9H), 1.41 (s, 9H), 1.37 (s, 9H); ^{13}C NMR (101 MHz, CD_3OD): 174.9 (C), 174.6 (C), 173.9 (C), 173.8 (C), 173.8 (C), 173.6 (C), 158.5 (C), 158.5 (C), 158.5 (C), 145.2 (C), 145.2 (C), 142.6 (2xC), 128.8 (2xCH), 128.2 (CH), 128.2 (CH), 126.3 (CH), 126.2 (CH), 121.0 (CH), 120.9 (CH), 81.9 , 81.8 (C), 80.5 (C), 79.9 (C), 79.8 (C), 72.3 (CH), 68.0 (CH_2), 56.1 (CH), 55.1 (CH), 54.7 (CH), 54.5 (CH), 48.5 (CH), 41.2 (2xCH_2), 32.6 (CH_2), 32.5 (CH_2), 32.3 (CH_2), 31.9 (CH_2), 30.5 (CH_2), 30.4 (CH_2), 29.6 (CH_2), 28.8 (6xCH_3), 28.4 (3xCH_3), 28.4 (3xCH_3), 28.0 (CH_2), 27.5 (CH_2), 24.3 (CH_2), 24.2 (CH_2).

Compound 75 was prepared following the general procedure of Fmoc deprotection, starting from **74** (3.1 g, 2.8 mmol) in 15 mL of 2 M dimethylamine in THF, yielding **75** as a colorless solid (2.5 g, 100%).

Compound 76. To a solution of **75** (2.4 g, 2.7 mmol) in dry DMF (10 mL), was added a 30 seconds-premixed solution of HATU (1.52 g, 3.24 mmol), Fmoc-Lys(Boc)-OH (1.23 g, 3.24 mmol) and DIPEA (566 µL, 3.24 mmol) in dry DMF (10 mL). Reaction mixture was stirred at rt for 30 min. Reaction mixture was diluted with EtOAc and washed with $NaHCO_3$ sat. aq. solution (x3), 10% citric acid solution (x3), water (x1), 5% LiCl solution (x2) and brine. Organic phase was then dried with Na_2SO_4, filtered and concentrated. Crude was dissolved in a minimal amount of CH_2Cl_2 and precipitated in pentane (x3). Crude was then purified by flash column chromatography (BGB® Scorpius 80 g SiO_2 col., 40 mL/min, linear gradient 0 – 6% MeOH in CH_2Cl_2) to yield **76** as a colorless solid (2.82 g, 79%). R_f (CH_2Cl_2 + 10% MeOH): 0.5; Mp: 180.7 – 181.0 °C; $[\alpha]_D^{20}$ + 1.7 (*c* 0.50, MeOH); IR (neat): 3312 (w), 2973 (w), 1721 (s), 1686 (s), 1631 (s), 1518 (s), 1444 (m), 1366 (s), 1230 (s), 1161 (s), 737 (m), 618 (m); ^{1}H NMR (400 MHz, CD_3OD): 7.80 (d, $^3J_{H-H}$ = 7.5 Hz, 2H), 7.67 (d, $^3J_{H-H}$ = 7.5, 1H), 7.66 (d, $^3J_{H-H}$ = 7.5, 1H),7.39 (t, $^3J_{H-H}$ = 7.5 Hz, 2H), 7.31 (t, $^3J_{H-H}$ = 7.5, 2H), 4.43 (dd, $^3J_{H-H}$ = 10.4, 6.9 Hz, 1H), 4.38 – 4.27 (m, 4H), 4.22 (t, $^3J_{H-H}$ = 7.0 Hz, 1H), 4.12 (t, $^3J_{H-H}$ = 7.3 Hz, 1H), 4.04 (dd, $^3J_{H-H}$ = 8.0, 6.5 Hz, 1H), 3.96 (d, $^4J_{H-H}$ = 2.5 Hz, 2H), 3.08 – 2.90 (m, 6H), 2.56 (t, $^4J_{H-H}$ = 2.5 Hz, 1H), 2.37 – 2.25 (m, 4H), 2.24 – 2.11 (m, 2H), 1.99 – 1.57 (m, 8H), 1.49 – 1.38 (m, 12H), 1.44 (s, 9H), 1.43 (s, 9H), , 1.42 (s, 18H), 1.41 (s, 18H); ^{13}C NMR (126 MHz, CD_3OD): 175.7 (C), 174.6 (C), 174.0 (C), 173.8 (C), 173.7 (C), 173.7(C), 173.6 (C), 158.6 (C), 158.5 (C), 158.4 (C), 158.4 (C), 145.3 (C), 145.1 (C), 142.6(C), 142.6 (C), 128.9 (CH), 128.8 (CH), 128.3 (CH), 128.2 (CH), 126.4 (CH), 126.3 (CH), 121.0 (CH), 121.0 (CH), 81.9 (C), 81.9 (C), 80.5 (C), 79.9 (C), 79.8 2x(C), 72.4 (CH), 68.1 (CH_2), 56.9 (CH), 55.6 (CH), 54.7 (CH), 54.3 (CH), 54.2 (CH), 48.4 (CH), 41.2 (CH_2), 41.2 (CH_2), 41.1 (CH_2), 32.7 (CH_2), 32.6 (2xCH_2),

32.3 (CH_2), 31.7 (CH_2), 30.6 (CH_2), 30.5 (CH_2), 30.4 (CH_2), 29.6 (9xCH_3), 28.8 (6xCH_3), 28.4 (CH_2), 28.4 (CH_2), 27.7 (CH_2), 27.5 (CH_2), 24.3 (2xCH_2), 24.2 (CH_2).

Compound 77 was prepared following the general procedure of Fmoc deprotection, starting from **76** (2.77 g, 2.07 mmol) in 25 mL of 2 M dimethylamine in THF, yielding **77** as a colorless solid (2.29 g, 100%).

Compound 78. To a solution of **77** (2.2 g, 2.0 mmol) in dry DMF (10 mL), was added a 30 seconds-premixed solution of HATU (901 mg, 2.37 mmol), Fmoc-D-Glu(O*t*Bu)-OH (1.00 g, 4.37 mmol) and DIPEA (415 µL, 2.37 mmol) in dry DMF (10 mL). Reaction mixture was stirred at rt for 30 min. Reaction mixture was diluted with EtOAc and washed with $NaHCO_3$ sat. aq. solution (x3), 10% citric acid solution (x3), water (x1), 5% LiCl solution (x2) and brine. Organic phase was then dried with Na_2SO_4, filtered and concentrated. Crude was dissolved in a minimal amount of CH_2Cl_2 and precipitated in pentane (x3). Crude was then purified by flash column chromatography (BGB® Scorpius 80 g SiO_2 col., 40 mL/min, linear gradient 0 – 6% MeOH in CH_2Cl_2) to yield **78** as a colorless solid (2.63 g, 87%). R_f (CH_2Cl_2 + 10% MeOH): 0.4; Mp: 195.1 – 196.4 °C; $[\alpha]_D^{20}$ – 3.1 (*c* 0.50, MeOH); IR (neat): 3292 (w), 2934 (w), 1682 (s), 1631 (s), 1519 (s), 1450 (m), 1366 (m), 1249 (s), 1155 (s), 739 (m), 620 (m); ^1H NMR (400 MHz, CD_3OD): 7.80 (d, $^3J_{H\text{-}H}$ = 7.4 Hz, 2H), 7.67 (d, $^3J_{H\text{-}H}$ = 7.4 Hz, 1H), 7.66 (d, $^3J_{H\text{-}H}$ = 7.4 Hz, 1H),7.39 (t, $^3J_{H\text{-}H}$ = 7.4 Hz, 2H), 7.32 (t, $^3J_{H\text{-}H}$ = 7.4 Hz, 2H), 4.45 (dd, $^3J_{H\text{-}H}$ = 10.5, 7.1 Hz, 1H), 4.36 – 4.24 (m, 4H), 4.24 – 4.09 (m, 4H), 3.94 (s, 2H), 3.09 – 2.93 (m, 6H), 2.56 (s, 1H), 2.40 – 2.24 (m, 6H), 2.21 – 2.07 (m, 2H), 2.07 – 1.77 (m, 7H), 1.77 – 1.62 (m, 3H), 1.53 – 1.37 (m, 12H), 1.46 (s, 9H), 1.43 (s, 27H), 1.41 (s, 9H), 1.40 (s, 9H); ^{13}C NMR (101 MHz, CD_3OD): 175.0 (C), 174.9 (C), 174.5 (C), 174.1 (C), 173.9 (C), 173.8 (2xC), 173.7 (C), 173.6 (C), 158.5 (4xC), 145.3 (C), 145.2 (C), 142.6 (2xC), 128.8 (CH), 128.2 (CH), 126.4 (CH), 126.3 (CH), 121.0 (CH), 120.9 (CH), 81.9 (C), 81.9 (C), 81.8 (C), 80.6 (C), 79.8 (2xC), 79.8 (C), 72.4 (CH), 68.1 (CH_2), 56.0 (CH), 55.3 (CH), 55.2 (CH), 54.8 (CH), 54.6 (CH), 54.5 (CH),

48.4 (CH), 41.2 (2xCH_2), 41.2 (CH_2), 32.6 (3xCH_2), 32.5 (CH_2), 32.0 (CH_2), 31.8 (CH_2), 30.5 (3xCH_2), 29.6 (CH_2), 28.8 (9xCH_3), 28.5 (3xCH_3), 28.4 (3xCH_3), 28.4 (3xCH_3), 28.3 (CH_2) , 27.9(CH_2), 27.3 (CH_2), 24.4 (CH_2), 24.3 (CH_2), 24.2 (CH_2).

Compound 79 was prepared following the general procedure of Fmoc deprotection, starting from **78** (1.0 g, 0.66 mmol) in 15 mL of 2 M dimethylamine in THF, yielding **79** as a colorless solid (850 mg, 99%).

Compound 80. To a solution of **79** (680 mg, 0.520 mmol) in dry DMF (10 mL), was added a 30 seconds-premixed solution of HATU (240 mg, 0.631 mmol), **35** (204 mg, 0.630 mmol) and DIPEA (110 µL, 0.630 mmol) in dry DMF (10 mL). Reaction mixture was stirred at rt for 3 h. Reaction mixture was diluted with EtOAc and washed with $NaHCO_3$ sat. aq. solution (x3), 10% citric acid solution (x3), water (x1), 5% LiCl solution (x2) and brine. Organic phase was then dried with Na_2SO_4, filtered and concentrated. Crude was dissolved in a minimal amount of CH_2Cl_2 and precipitated in pentane (x3). Crude was then purified by flash column chromatography (BGB® Scorpius 25 g SiO_2 col., 40 mL/min, linear gradient 0 – 6% MeOH in CH_2Cl_2) to yield **80** as a colorless solid (740 mg, 89%). R_f (CH_2Cl_2 + 10% MeOH): 0.4; Mp: 217.2 – 218.2 °C; $[\alpha]_D^{20}$ – 0.2 (*c* 0.50, MeOH); IR (neat): 3285 (m), 2934 (m), 1712 (m), 1688 (s), 1627 (s), 1519 (s), 1451 (m), 1366 (m), 1249 (m), 1154 (s), 737 (m), 638 (m); ^{1}H NMR (500 MHz, CD_3OD): 4.38 – 4.20 (m, 5H), 4.17 (dd, $^3J_{H\text{-}H}$ = 8.8, 5.9 Hz, 1H), 3.97 (d, $^4J_{H\text{-}H}$ = 2.5 Hz, 2H), 3.63 – 3.58 (m, 4H), 3.56 (t, $^3J_{H\text{-}H}$ = 6.7 Hz, 2H), 3.54 (t, $^3J_{H\text{-}H}$ = 5.9 Hz, 2H), 3.49 (t, $^3J_{H\text{-}H}$ = 6.7 Hz, 2H), 3.36 (t, $^3J_{H\text{-}H}$ = 5.9 Hz, 2H), 3.10 – 2.97 (m, 6H), 2.58 (t, $^4J_{H\text{-}H}$ = 2.5 Hz, 1H), 2.57 – 2.48 (m, 4H), 2.40 – 2.27 (m, 6H), 2.24 – 2.05 (m, 3H), 2.03 – 1.82 (m, 6H), 1.81 – 1.73 (m, 4H), 1.73 – 1.65 (m, 4H), 1.64 – 1.57 (m, 2H), 1.55 – 1.37 (m, 18H), 1.45 (s, 27H), 1.43 (s, 27H); ^{13}C NMR (126 MHz, CD_3OD): 175.3 (C), 175.0 (C), 174.7 (C), 174.6 (C), 174.6 (C), 174.1 (C), 173.8 (2xC), 173.8 (C), 173.8 (C), 173.7 (C), 158.4 (3xC), 81.9 (C), 81.8 (C), 81.8 (C), 80.6 (C), 79.8 (3xC), 72.4 (CH), 72.3 (CH_2), 71.3 (CH_2), 71.2 (CH_2), 70.6

(CH_2), 55.7 (CH), 55.4 (CH), 54.7 (3xCH), 54.3 (CH), 45.7 (CH_2), 41.2 (3xCH_2), 40.5 (CH_2), 33.8 (CH_2), 32.7 (CH_2), 32.7 (2xCH_2), 32.6 (CH_2), 32.1 (CH_2), 31.9 (CH_2), 31.9 (CH_2), 31.5 (CH_2), 30.5 (4xCH_2), 30.5 (CH_2), 30.4 (CH_2), 29.6 (CH_2), 28.9 (9xCH_3), 28.5 (6xCH_3), 28.5 (3xCH_3), 28.0 (CH_2), 27.8 (CH_2), 27.7 (CH_2), 27.4 (CH_2), 26.5 (CH_2), 24.4 (CH_2), 24.4 (CH_2), 24.3 (CH_2).

Compound 81. To a solution of **80** (208 mg, 0.130 mmol) in THF/H_2O 2/1 (9 mL), **36** (50 mg, 0.16 mmol), $CuSO_4 \cdot 5H_2O$ (32 mg, 0.16 mmol), sodium ascorbate (52 mg, 0.26 mmol) and TBTA (7 mg, 0.01 mmol) were added and the reaction mixture was stirred at rt for 1 h. Solvent was removed *in vacuo*, H_2O and CH_2Cl_2 were added, phases were separated, and aqueous phase was extracted by CH_2Cl_2 (x3). Organic phases were collected together, washed with brine (x1), dried over Na_2SO_4, filtered and concentrated. Crude was purified by flash column chromatography (BGB® Scorpius 25g, 25 mL/min, linear gradient 0 – 8% MeOH in CH_2Cl_2) to yield **81** as a colorless solid (199 mg, 80%). R_f (CH_2Cl_2 + 10% MeOH): 0.3; Mp: 193.1 – 194.7 °C; $[\alpha]_D^{20}$ + 11.6 (*c* 0.50, MeOH); IR (neat): 3283 (m), 2936 (m), 1690 (s), 1624 (s), 1519 (s), 1452 (m), 1365 (m), 1248 (s), 1151 (s), 847 (m), 649 (m); ^{1}H NMR (400 MHz, CD_3OD): 7.84 (s, 1H), 4.54 – 4.47 (m, 4H), 4.44 (d, $^2J_{H\text{-}H}$ = 15.4 Hz, 1H), 4.37 – 4.14 (m, 7H), 3.68 – 3.62 (m, 2H), 3.62 – 3.57 (m, 4H), 3.56 (t, $^3J_{H\text{-}H}$ = 6.6 Hz, 2H), 3.53 (t, $^3J_{H\text{-}H}$ = 5.5 Hz, 2H), 3.49 (t, $^3J_{H\text{-}H}$ = 6.5 Hz, 2H), 3.36 (t, $^3J_{H\text{-}H}$ = 5.5 Hz, 2H), 3.23 (ddd, $^3J_{H\text{-}H}$ = 8.7, 6.1, 4.5 Hz, 1H), 3.07 – 2.99 (m, 6H), 2.95 (dd, $^2J_{H\text{-}H}$ = 12.7, $^3J_{H\text{-}H}$ = 5.0 Hz, 1H), 2.72 (d, $^2J_{H\text{-}H}$ = 12.7 Hz, 1H), 2.61 – 2.46 (m, 4H), 2.42 – 2.25 (m, 6H), 2.22 – 2.05 (m, 5H), 2.00 – 1.83 (m, 6H), 1.82 – 1.69 (m, 7H), 1.67 – 1.56 (m, 6H), 1.53 – 1.32 (m, 16H), 1.45 (s, 9H), 1.45 (s, 9H), 1.45 (s, 9H), 1.43 (s, 27H); ^{13}C NMR (126 MHz, CD_3OD): 175.0 (C), 173.9 (C), 173.6 (C), 173.3 (C), 173.2 (C), 173.1 (C), 173.0 (C), 172.8 (C), 172.6 (C), 172.4 (2xC), 172.3 (C), 164.7 (C), 157.0 (C), 123.4 (CH), 80.5 (2xC), 80.4 (C), 78.4 (3xC), 70.8 (CH_2), 69.9 (CH_2), 69.8 (CH_2), 69.2 (CH_2), 61.9 (CH), 60.2 (CH), 55.7 (CH), 54.2 (CH), 54.0 (CH), 53.8 (CH), 53.3 (2xCH),

53.1 (CH), 49.2 (CH_2), 44.3 (CH_2), 39.8 (4xCH_2), 39.1 (CH_2), 39.1 (CH_2), 35.2 (CH_2), 34.5 (CH_2), 32.4 (CH_2), 31.3 (CH_2), 31.2 (2xCH_2), 30.8 (CH_2), 30.7 (CH_2), 30.6 (CH_2), 30.5 (CH_2), 30.2 (CH_2), 29.1 (CH_2), 29.1 (2xCH_2), 29.1 (CH_2), 28.2 (CH_2), 28.0 (CH_2), 27.5 (9xCH_3), 27.1 (6xCH_3), 27.1 (3xCH_3), 26.5 (CH_2), 26.3 (CH_2), 26.2 (CH_2), 26.0 (CH_2), 25.3 (CH_2), 25.1 (CH_2), 23.0 (2xCH_2), 23.0 (CH_2).

Compound 82. A solution of **81** (185 mg, 96.6 µmol) in TFA/H_2O/TIPS 95/2.5/2.5 (2 mL) was stirred at rt for 30 min. Solvent was removed *in vacuo*, crude was dissolved in small amount of MeOH and was precipitated in Et_2O (x3) to yield **82** as a colorless TFA salt (149 mg, 86%). Crude was used in the next reaction without further purification.

Compound 8. To a solution of **82** (40 mg, 22 µmol) in DMF (800 µL), **37** (22 mg, 89 µmol) and DIPEA (31 µL, 178 µmol) were added and reaction mixture was stirred at rt for 1 h. Crude reaction mixture was purified by RP flash chromatography (Biotage® SNAP Ultra 12 g C18, 12 mL/min, linear gradient 20 – 50% CH_3CN in H_2O with 0.1% TFA) to yield **8** as a colorless solid (15.6 mg, 38%). ^{1}H NMR (500 MHz, DMSO-d_6/D_2O 60/1): 7.84 (s, 1H), 4.37 (t, $^3J_{H-H}$ = 6.2 Hz, 2H), 4.33 – 4.26 (m, 4H), 4.25 – 4.17 (m, 4H), 4.16 – 4.11 (m, 2H), 3.61 (t, $^3J_{H-H}$ = 6.6 Hz, 2H), 3.51 – 3.45 (m, 6H), 3.40 – 3.34 (m, 10H), 3.21 – 3.13 (m, 8H), 3.12 – 3.09 (m, 1H), 3.10 (t, $^3J_{H-H}$ = 7.3 Hz, 2H), 3.08 – 2.95 (m, 6H), 2.82 (dd, $^3J_{H-H}$ = 12.5, $^4J_{H-H}$ = 5.1 Hz, 1H), 2.58 (d, $^3J_{H-H}$ = 12.5 Hz, 1H), 2.42 – 2.27 (m, 4H), 2.26 – 2.14 (m, 6H), 2.04 (t, $^3J_{H-H}$ = 7.1 Hz, 2H), 2.01 – 1.84 (m, 3H), 1.81 – 1.60 (m, 8H), 1.59 – 1.41 (m, 9H), 1.40 – 1.33 (m, 8H), 1.33 – 1.17 (m, 11H); ^{13}C NMR (126 MHz, DMSO-d_6): 173.9 (C), 173.8 (3xC), 172.6 (C) , 172.1 (C), 171.9 (C), 171.7 (2xC), 171.6 (C), 171.4 (C), 171.1 (C), 170.9 (C), 170.3 (C), 170.2 (2xC), 162.8 (C), 144.6 (C), 123.1 (CH), 70.3 (CH_2), 69.6 (CH_2), 69.4 (CH_2), 69.1 (CH_2), 61.0 (CH), 59.2 (CH), 55.4 (CH), 52.9 (CH), 52.5 (CH), 52.3 (CH), 52.3 (CH), 52.2 (CH), 52.0 (CH), 51.5 (3xCH), 48.7 (CH_2), 45.5 (CH_2), 42.1 (6xCH_2), 39.6 (CH_2), 38.6 (CH_2), 38.4 (CH_2), 38.4 (CH_2), 38.4 (CH_2), 38.3 (CH_2), 35.1 (CH_2), 34.2 (CH_2), 32.1 (CH_2), 31.5 (CH_2), 31.5

(CH2), 31.0 (CH2), 30.6 (CH2), 30.5 (CH2), 30.1 (CH2), 30.0 (CH2), 30.0 (CH2), 29.1 (CH2), 28.5 (2xCH2), 28.5 (CH2), 28.2 (CH2), 28.0 (CH2), 27.3 (CH2), 27.0 (CH2), 26.9 (CH2), 26.2 (CH2), 25.2 (CH2), 25.0 (CH2), 22.8 (CH2), 22.7 (CH2), 22.7 (CH2); HRMS (ESI, +ve) calcd for $C_{74}H_{118}ClN_{17}O_{21}S_7$ ($[M + H]^+$): 1840.6495, found: 1840.6439; LC-MS (ESI, B30): R_t 1.58 min, 1842 (50, $[M+H]^+$), 921 (100, $[M+2H]^{2+}$).

2.1.5. Synthesis of peptide 9

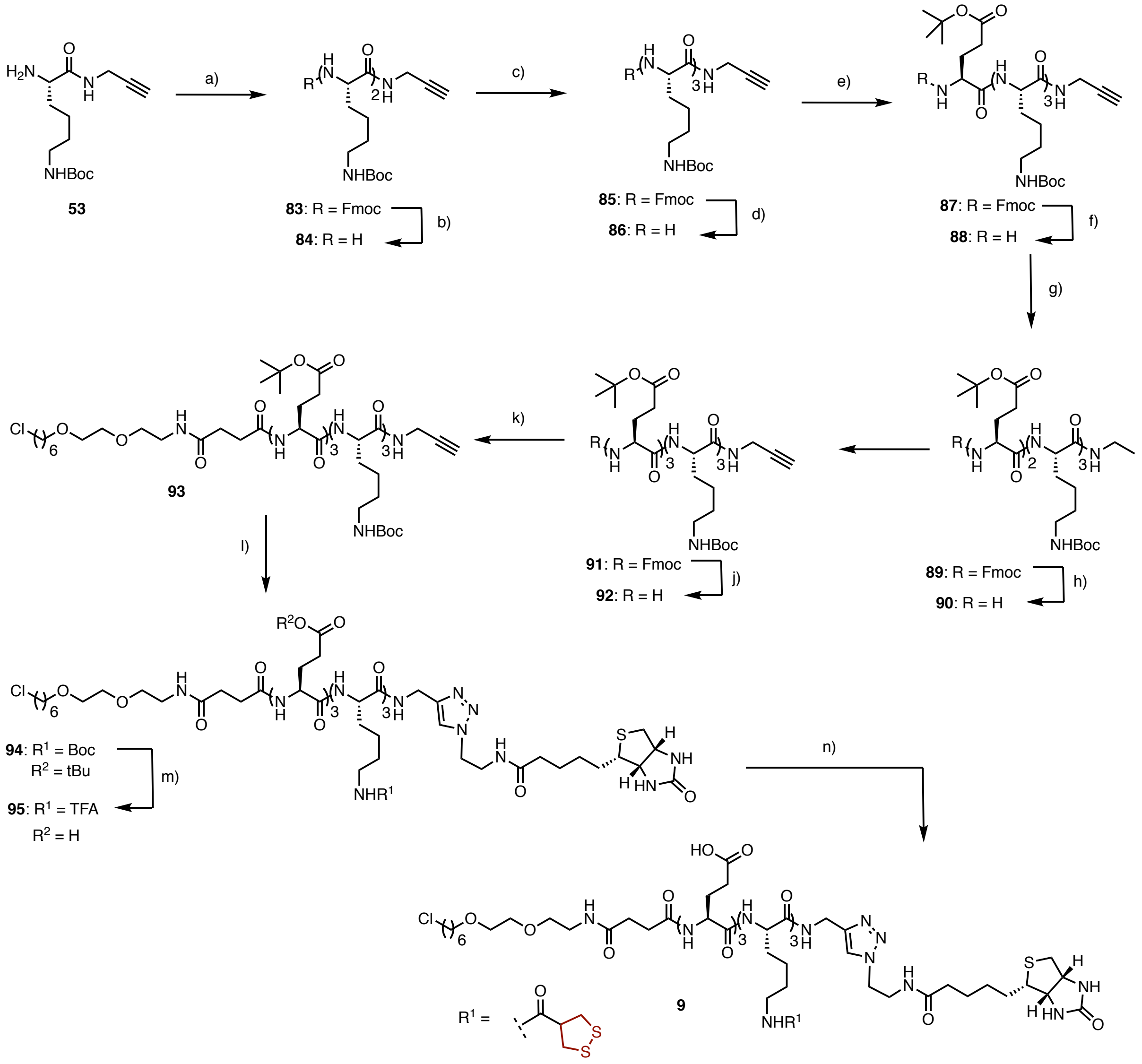

Scheme S5 (a) Fmoc-Lys(Boc)-OH, EDCI·HCl, DMAP, CH_2Cl_2/DMF, rt, 15 h, 64%; (b) 2 M dimethylamine in THF, rt, 30 min, 85%; (c) Fmoc-Lys(Boc)-OH, EDCI·HCl, DMAP,

CH_2Cl_2/DMF, rt, 15 h, 69%; (d) 2 M dimethylamine in THF, rt, 30 min, 100%; (e) Fmoc-Glu(O*t*Bu)-OH, EDCI·HCl, DMAP, CH_2Cl_2/DMF, rt, 15 h, 87%; (f) 2 M dimethylamine in THF, rt, 30 min, 99%; (g) Fmoc-Glu(O*t*Bu)-OH, EDCI·HCl, DMAP, CH_2Cl_2/DMF, rt, 15 h, 85%; (h) 2 M dimethylamine in THF, rt, 30 min, 86%; (i) Fmoc-Glu(O*t*Bu)-OH, EDCI·HCl, DMAP, CH_2Cl_2/DMF, rt, 15 h, 33%; (j) 2 M dimethylamine in THF, rt, 30 min, *quant.*; (k) **35**, EDCI·HCl, DMAP, CH_2Cl_2/DMF, rt, 15 h, 90%; (l) **36**, $CuSO_4 \cdot 5H_2O$, Na-ascorbate, TBTA, THF/H_2O, rt, 1 h, 53%; (m) TFA/TIPS/H_2O 95/2.5/2.5, rt, 30 min, 94%; (n) **37**, DIPEA, DMF, rt, 1 h, 40%.

Compound 83. To a solution of **53** (1.5 g, 5.3 mmol) in CH_2Cl_2 (200 mL) were added Fmoc-Lys(Boc)-OH (2.98 mg, 6.36 mmol), EDCI·HCl (1.22 g, 6.36 mmol), and DMAP (203 mg, 1.66 mmol), and the reaction mixture was stirred at rt for 15 h. Product precipitated in CH_2Cl_2. Precipitate was filtered and was purified by flash column chromatography (Claricep® 100 g, 50 mL/min, linear gradient 0 – 10% MeOH in CH_2Cl_2) to yield **83** as a colorless solid (2.5 g, 64%). R_f (CH_2Cl_2 + 10% MeOH): 0.8; Mp: 159.2 – 160.5 °C; $[\alpha]_D^{20}$ – 18.7 (*c* 0.50, MeOH); IR (neat): 3324 (w), 2936 (w), 2447 (w), 1683 (s), 1635 (s), 1535 (m), 1246 (m), 1166 (s), 995 (m), 738 (s), 666 (m); ^{1}H NMR (400 MHz, CD_3OD): 7.80 (d, $^3J_{H-H}$ = 7.5 Hz, 2H), 7.68 (d, $^3J_{H-H}$ = 7.5 Hz, 1H), 7.66 (d, $^3J_{H-H}$ = 7.5 Hz, 1H), 7.39 (t, $^3J_{H-H}$ = 7.5 Hz, 2H), 7.31 (t, $^3J_{H-H}$ = 7.5 Hz, 2H), 4.43 – 4.35 (m, 2H), 4.31 (dd, $^3J_{H-H}$ = 8.9, 5.4 Hz, 2H), 4.23 (t, $^3J_{H-H}$ = 6.8 Hz, 1H), 4.07 (dd, $^3J_{H-H}$ = 8.6, 5.4 Hz, 1H), 3.99 (dd, $^2J_{H-H}$ = 17.5, $^4J_{H-H}$ = 2.5 Hz, 2H), 3.92 (dd, $^2J_{H-H}$ = 17.5, $^4J_{H-H}$ = 2.5 Hz, 2H), 3.08 – 2.96 (m, 4H), 2.57 (t, $^4J_{H-H}$ = 2.5 Hz, 2H), 1.85 – 1.70 (m, 2H), 1.71 – 1.57 (m, 2H), 1.53 – 1.29 (m, 8H), 1.43 (s, 9H), 1.39 (s, 9H); ^{13}C NMR (101 MHz, CD_3OD): 174.9 (C), 173.8 (C), 158.6 (C), 158.6 (C), 158.5 (C), 145.3 (C), 145.2 (C), 142.6 (2xC), 128.8 (2xCH), 128.2 (CH), 128.2 (CH), 126.2 (2xCH), 120.9 (2xCH), 80.4 (C), 79.9 (2xC), 72.4 (CH), 68.0 (CH_2), 56.5 (CH), 54.3 (CH), 48.4 (CH), 41.1 (CH_2), 41.0 (CH_2),

32.7 (2xCH_2), 30.6 (CH_2), 30.4 (CH_2), 29.5 (CH_2), 28.8 (3xCH_3), 28.8 (3xCH_3), 24.1 (CH_2), 24.1 (CH_2).

Compound 84 was prepared following the general procedure of Fmoc deprotection, starting from **83** (2.5 g, 3.4 mmol) in 15 mL of 2 M dimethylamine in THF, yielding **84** as a colorless solid (1.5 g, 85%).

Compound 85. To a solution of **84** (1.5 g, 2.9 mmol) in CH_2Cl_2/DMF (200 mL/ 10 mL) were added Fmoc-Lys(Boc)-OH (1.64 g, 3.50 mmol), EDCI·HCl (671 mg, 3.50 mmol), and DMAP (110 mg, 0.87 mmol), and the reaction mixture was stirred at rt for 15 h. The organic phase was washed with H_2O (x2) and brine (x1), 5% LiCl (x2), dried over Na_2SO_4, filtered and concentrated. Crude was purified by flash column chromatography (Claricep® 100 g, 50 mL/min, linear gradient 0 – 10% MeOH in CH_2Cl_2) to yield **85** as a colorless solid (1.91 g, 69%). R_f (CH_2Cl_2 + 10% MeOH): 0.7; Mp: 173.1 – 174.2 °C; $[\alpha]_D^{20}$ – 23.4 (*c* 0.50, MeOH); IR (neat): 3300 (m), 2935 (m), 1684 (s), 1635 (s), 1529 (s), 1454 (m), 1252 (s), 1167 (s), 737 (m), 647 (m); ^{1}H NMR (400 MHz, CD_3OD): 7.80 (d, $^3J_{H-H}$ = 7.5 Hz, 2H), 7.68 (d, $^3J_{H-H}$ = 7.4 Hz, 1H), 7.66 (d, $^3J_{H-H}$ = 7.4 Hz, 1H), 7.39 (t, $^3J_{H-H}$ = 7.4 Hz, 2H), 7.31 (t, $^3J_{H-H}$ = 7.4 Hz, 2H), 4.45 – 4.25 (m, 4H), 4.22 (t, $^3J_{H-H}$ = 6.8 Hz, 1H), 4.08 (t, $^3J_{H-H}$ = 7.2 Hz, 1H), 3.99 (dd, $^2J_{H-H}$ = 17.5, $^4J_{H-H}$ = 2.5 Hz, 1H), 3.92 (dd, $^2J_{H-H}$ = 17.5, $^4J_{H-H}$ = 2.5 Hz, 1H), 3.11 – 2.88 (m, 6H), 2.58 (t, $^4J_{H-H}$ = 2.5 Hz, 1H), 1.90 – 1.72 (m, 3H), 1.71 – 1.59 (m, 3H), 1.57 – 1.33 (m, 12H), 1.43 (s, 9H), 1.42 (s, 9H), 1.39 (s, 9H). ^{13}C NMR (101 MHz, CD_3OD): 173.8 (C), 172.7 (C), 172.3 (C), 157.2 (C), 157.2 (C), 157.1 (2xC), 143.9 (C), 143.8 (C), 141.2 (2xC), 127.4 (2xCH), 126.8 (2xCH), 124.9 (2xCH), 119.5 (2xCH), 79.0 (C), 78.5 (3xC), 71.0 (CH), 66.6 (CH_2), 55.2 (CH), 53.3 (CH), 53.1 (CH), 47.0 (CH), 39.8 (CH_2), 39.7 (CH_2), 39.7 (CH_2), 31.4 (2xCH_2), 31.1 (CH_2), 29.2 (CH_2), 29.1 (CH_2), 29.0 (CH_2), 28.1 (CH_2), 27.4 (6xCH_3), 27.4 (3xCH_3), 22.8 (CH_2), 22.7 (2xCH_2).

Compound 86 was prepared following the general procedure of Fmoc deprotection, starting from **85** (1.90 g, 1.97 mmol) in 16 mL of 2 M dimethylamine in THF, yielding **86** as a colorless solid (1.55 g, 100%).

Compound 87. To a solution of **86** (1.45 g, 1.97 mmol) in CH_2Cl_2/DMF (150 mL/ 10 mL) were added Fmoc-Glu(O*t*Bu)-OH (1.02 g, 2.40 mmol), EDCI·HCl (460 mg, 2.40 mmol), and DMAP (76 mg, 0.62 mmol), and the reaction mixture was stirred at rt for 15 h. The organic phase was washed with H_2O (x2) and brine (x1), 5% LiCl (x2), dried over Na_2SO_4, filtered and concentrated. Crude was purified by flash column chromatography (Claricep® 80 g, 50 mL/min, linear gradient 0 – 8% MeOH in CH_2Cl_2) to yield **87** as a colorless solid (1.98 g, 87%). R_f (CH_2Cl_2 + 10% MeOH): 0.4; Mp: 170.3 – 171.0 °C; $[\alpha]_D^{20}$ – 23.1 (*c* 0.50, MeOH); IR (neat): 3287 (w), 2931 (w), 2430 (w), 1677 (s), 1629 (s), 1526 (s), 1423 (s), 1250 (m), 1157 (s), 733 (m), 570 (m); ^{1}H NMR (400 MHz, CD_3OD): 7.80 (d, $^3J_{H-H}$ = 7.5 Hz, 2H), 7.67 (d, $^3J_{H-H}$ = 7.5 Hz, 2H), 7.39 (t, $^3J_{H-H}$ = 7.5 Hz, 2H), 7.32 (t, $^3J_{H-H}$ = 7.5 Hz, 2H), 4.47 – 4.39 (m, 1H), 4.38 – 4.20 (m, 5H), 4.11 (t, $^3J_{H-H}$ = 6.8 Hz, 1H), 3.99 (dd, $^2J_{H-H}$ = 17.5, $^4J_{H-H}$ = 2.5 Hz, 1H), 3.92 (dd, $^2J_{H-H}$ = 17.5, $^4J_{H-H}$ = 2.5 Hz, 1H), 3.06 – 2.93 (m, 6H), 2.58 (t, $^4J_{H-H}$ = 2.5 Hz, 1H), 2.34 (t, $^3J_{H-H}$ = 7.5 Hz, 2H), 2.12 – 1.96 (m, 1H), 1.96 – 1.74 (m, 4H), 1.73 – 1.54 (m, 3H), 1.52 – 1.37 (m, 12H), 1.45 (s, 9H), 1.42 (s, 18H), 1.40 (s, 9H); ^{13}C NMR (101 MHz, CD_3OD): 174.6 (C), 174.4 (C), 174.1 (C), 173.9 (C), 173.8 (C), 158.6 (C), 158.5 (3xC), 145.3 (C), 145.2 (C), 142.6 (2xC), 128.8 (2xCH), 128.2 (2xCH), 126.3 (CH), 126.2 (CH), 121.0 (2xCH), 81.9 (C), 80.4 (C), 79.8, (3xC) 72.4 (CH), 68.1 (CH_2), 56.2 (CH), 55.0 (2xCH), 54.6 (CH), 48.4 (CH_2), 41.2 (CH_2), 41.2 (2xCH_2), 32.7 (3xCH_2), 32.4 (CH_2), 30.5 (3xCH_2), 29.5 (CH_2), 28.9 (6xCH_3), 28.8 (3xCH_3), 28.4 (3xCH_3), 28.2 (CH_2), 24.2 (2xCH_2), 24.1 (CH_2).

Compound 88 was prepared following the general procedure of Fmoc deprotection, starting from **87** (1.98 g, 1.72 mmol) in 20 mL of 2 M dimethylamine in THF, yielding **88** as a colorless solid (1.57 g, 99%).

Compound 89. To a solution of **88** (1.5 g, 1.6 mmol) in CH_2Cl_2/DMF (200 mL/ 10 mL) were added Fmoc-Glu(O*t*Bu)-OH (817 mg, 1.92 mmol), EDCI·HCl (368 mg, 1.92 mmol), and DMAP (61 mg, 0.48 mmol), and the reaction mixture was stirred at rt for 15 h. The organic phase was washed with H_2O (x2) and brine (x1), 5% LiCl (x2), dried over Na_2SO_4, filtered and concentrated. Crude was purified by flash column chromatography (Claricep® 80 g, 50 mL/min, linear gradient 0 – 8% MeOH in CH_2Cl_2) to yield **89** as a colorless solid (1.81 g, 85%). R_f (CH_2Cl_2 + 10% MeOH): 0.4; Mp: 195.5 – 196.7 °C; $[\alpha]_D^{20}$ – 18.3 (*c* 0.50, MeOH); IR (neat): 3271 (w), 2939 (w), 2488 (w), 1692 (s), 1622 (s), 1518 (m), 1440 (m), 1367 (m), 1250 (s), 1149 (s), 738 (m), 570 (m); ^{1}H NMR (400 MHz, CD_3OD): 7.81 (d, $^3J_{H-H}$ = 7.4 Hz, 2H), 7.68 (d, $^3J_{H-H}$ = 7.4 Hz, 2H), 7.40 (t, $^3J_{H-H}$ = 7.4 Hz, 2H), 7.32 (t, $^3J_{H-H}$ = 7.4 Hz, 2H), 4.50 – 4.40 (m, 1H), 4.36 (dd, $^3J_{H-H}$ = 10.4, 6.9 Hz, 1H), 4.31 – 4.18 (m, 5H), 4.08 (t, $^3J_{H-H}$ = 5.7 Hz, 1H), 3.99 (dd, $^2J_{H-H}$ = 17.5, $^4J_{H-H}$ = 2.5 Hz, 1H), 3.93 (dd, $^2J_{H-H}$ = 17.5, $^4J_{H-H}$ = 2.5 Hz, 1H), 3.08 – 2.94 (m, 6H), 2.57 (t, $^4J_{H-H}$ = 2.5 Hz, 1H), 2.36 (t, $^3J_{H-H}$ = 7.1 Hz, 4H), 2.13 – 2.00 (m, 2H), 1.99 – 1.88 (m, 2H), 1.87 – 1.77 (m, 3H), 1.76 – 1.63 (m, 3H), 1.54 – 1.31 (m, 12H), 1.45 (s, 18H), 1.42 (s, 27H); ^{13}C NMR (101 MHz, CD_3OD): 175.1 (C), 174.7 (C), 174.3 (C), 174.2 (C), 174.1 (C), 173.8 (C), 173.8 (C), 158.9 (C), 158.4 (3xC), 145.2 (2xC), 142.6 (2xC), 128.9 (2xCH), 128.2 (2xCH), 126.3 (CH), 126.2 (CH), 121.0 (2xCH), 81.9 (C), 81.9 (2xC), 80.5 (C), 79.8 (2xC), 72.3 (CH), 68.2 (CH_2), 56.6 (CH), 55.5 (CH), 55.2 (2xCH), 54.7 (CH), 48.1 (CH), 41.2 (CH_2), 41.2 (2xCH_2), 32.7 (CH_2), 32.6 (3xCH_2), 32.3 (CH_2), 32.1 (CH_2), 30.5 (3xCH_2), 29.5 (CH_2), 28.9 (9xCH_3), 28.4 (3xCH_3), 28.4 (3xCH_3), 27.9 (CH_2), 27.6 (CH_2), 24.3 (2xCH_2), 24.2 (CH_2).

Compound 90 was prepared following the general procedure of Fmoc deprotection, starting from **59** (1.8 g, 1.6 mmol) in 15 mL of 2 M dimethylamine in THF, yielding **60** as a colorless solid (1.53 g, 86%).

Compound 91. To a solution of **90** (1.45 g, 1.31 mmol) in CH_2Cl_2/DMF (200 mL/ 10 mL) were added Fmoc-Glu(O*t*Bu)-OH (663 mg, 1.56 mmol), EDCI·HCl (299 mg, 1.56 mmol), and DMAP (50 mg, 0.41 mmol), and the reaction mixture was stirred at rt for 15 h. The organic phase was washed with H_2O (x2) and brine (x1), 5% LiCl (x2), dried over Na_2SO_4, filtered and concentrated. Crude was purified by flash column chromatography (Claricep® 80 g, 50 mL/min, linear gradient 0 – 6% MeOH in CH_2Cl_2) to yield **91** as a colorless solid (660 mg, 33%). R_f (CH_2Cl_2 + 10% MeOH): 0.5; Mp: 206.7 – 208.1 °C; $[\alpha]_D^{20}$ – 18.1 (*c* 0.50, MeOH); IR (neat): 3291 (w), 2935 (w), 2426 (w), 1688 (s), 1625 (s), 1522 (m), 1446 (m), 1367 (m), 1241 (m), 1152 (s), 849 (m), 733 (m), 564 (m); ^{1}H NMR (400 MHz, CD_3OD): 7.81 (d, $^3J_{H\text{-}H}$ = 7.4 Hz, 2H), 7.69 (d, $^3J_{H\text{-}H}$ = 7.4 Hz, 2H), 7.40 (t, $^3J_{H\text{-}H}$ = 7.4 Hz, 2H), 7.32 (t, $^3J_{H\text{-}H}$ = 7.4 Hz, 2H), 4.53 – 4.45 (m, 1H), 4.39 (dd, $^3J_{H\text{-}H}$ = 10.5, 6.8 Hz, 1H), 4.31 – 4.15 (m, 6H), 4.05 (t, $^3J_{H\text{-}H}$ = 7.3 Hz, 1H), 4.02 – 3.90 (m, 2H), 3.10 – 2.96 (m, 6H), 2.56 (t, $^4J_{H\text{-}H}$ = 2.5 Hz, 1H), 2.47 – 2.27 (m, 6H), 2.14 – 1.91 (m, 6H), 1.90 – 1.65 (m, 6H), 1.53 – 1.33 (m, 12H), 1.45 (s, 9H), 1.42 (s, 9H), 1.42 (s, 27H), 1.39 (s, 9H); ^{13}C NMR (101 MHz, CD_3OD): 174.8 (C), 174.7 (C), 174.5 (C), 174.4 (C), 174.1 (C), 174.1 (C), 173.9 (C), 173.9 (C), 173.8 (C), 159.1 (C), 158.4 (3xC), 145.2 (2xC), 142.7 (2xC), 128.9 (2xCH), 128.2 (2xCH), 126.3(CH), 126.2 (CH), 121.0 (2xCH), 82.0 (C), 81.9 (C), 81.9 (C), 80.5 (C), 79.8 (3xC), 72.2 (CH), 68.3 (CH_2), 57.0 (2xCH), 56.1 (CH), 56.0 (CH), 55.8 (CH), 54.9 (CH), 48.2 (CH), 41.3 (CH_2), 32.8 (CH_2), 32.6 (CH_2), 32.6 (2xCH_2), 32.2 (CH_2), 32.0 (CH_2), 30.6 (3xCH_2), 29.6 (CH_2), 28.9 (9xCH_3), 28.5 (9xCH_3), 28.4 (CH_2), 27.2 (CH_2), 27.0 (CH_2), 24.5 (2xCH_2), 24.3 (CH_2).

Compound 92 was prepared following the general procedure of Fmoc deprotection, starting from **91** (620 mg, 0.41 mmol) in 10 mL of 2 M dimethylamine in THF, yielding **92** as a colorless solid (550 mg, *quant.*).

Compound 93. To a solution of **92** (530 mg, 0.41 mmol) in DMF (10 mL) were added **35** (146 mg, 0.45 mmol), EDCI·HCl (82 mg, 0.45 mmol), and DMAP (15 mg, 0.12 mmol), and

the reaction mixture was stirred at rt for 15 h. EtOAc was added and the organic phase was washed with 10% citric acid (x2), H_2O (x2) and brine (x1), 5% LiCl (x2), dried over Na_2SO_4, filtered and concentrated. Crude was purified by flash column chromatography (BGB® Scorpius 40 g, 50 mL/min, linear gradient 0 – 8% MeOH in CH_2Cl_2) to yield **93** as a colorless solid (588 mg, 90%). R_f (CH_2Cl_2 + 10% MeOH): 0.4; Mp: 211.0 – 212.9 °C; $[\alpha]_D^{20}$ + 3.9 (*c* 0.50, MeOH); IR (neat): 3291 (w), 2944 (w), 1714 (m), 1626 (s), 1518 (s), 1365 (m), 1244 (m), 1152 (s), 690 (m), 573 (m); ^{1}H NMR (500 MHz, CD_3OD): 4.16 (dd, $^3J_{H\text{-}H}$ = 10.0, 4.4 Hz, 1H), 4.08 – 3.94 (m, 5H), 3.90 (dd, $^2J_{H\text{-}H}$ = 17.4, $^4J_{H\text{-}H}$ = 2.5 Hz, 1H), 3.85 (dd, $^2J_{H\text{-}H}$ = 17.4, $^4J_{H\text{-}H}$ = 2.5 Hz, 1H), 3.52 – 3.42 (m, 6H), 3.46 (t, $^3J_{H\text{-}H}$ = 6.7 Hz, 2H), 3.39 (t, $^3J_{H\text{-}H}$ = 6.6 Hz, 2H), 3.37 – 3.24 (m, 2H), 3.00 – 2.87 (m, 6H), 2.75 – 2.64 (m, 1H), 2.49 – 2.41 (m, 2H), 2.43 (t, $^4J_{H\text{-}H}$ = 2.5 Hz, 1H), 2.40 – 2.18 (m, 7H), 2.14 – 2.00 (m, 3H), 1.99 – 1.84 (m, 3H), 1.83 – 1.64 (m, 8H), 1.54 – 1.25 (m, 18H), 1.35 (s, 9H), 1.34 (s, 18H), 1.32 (s, 27H); ^{13}C NMR (126 MHz, CD_3OD): 176.6 (C), 176.0 (C), 176.0 (C), 175.9 (C), 175.9 (C), 175.0 (C), 174.8 (C), 174.2 (C), 173.6 (2xC), 173.4 (C), 158.4 (C), 158.3 (C), 158.3 (C), 81.9 (C), 81.8 (C), 81.8 (C), 80.6 (C), 79.8 (C), 79.7 (C), 79.7 (C), 72.3 (CH_2), 72.0 (CH), 71.3 (CH_2), 71.2 (CH_2), 70.7 (CH_2), 57.2 (CH), 57.1 (CH), 56.8 (CH), 56.4 (CH), 56.4 (CH), 55.3 (CH), 45.7 (CH_2), 41.3 (CH_2), 41.3 (2xCH_2), 40.6 (CH_2), 33.8 (CH_2), 32.8 (CH_2), 32.7 (CH_2), 32.4 (CH_2), 32.4 (CH_2), 32.4 (CH_2), 32.1 (CH_2), 31.9 (CH_2), 31.8 (CH_2), 30.7 (CH_2), 30.5(CH_2), 30.5 (2xCH_2), 29.6 (CH_2), 28.9 (3xCH_3), 28.9 (6xCH_3), 28.5 (3xCH_3), 28.5 (3xCH_3), 28.5 (3xCH_3), 27.7 (CH_2), 27.3 (CH_2), 27.1 (CH_2), 26.7 (CH_2), 26.5 (CH_2), 24.8 (CH_2), 24.7 (CH_2), 24.5 (CH_2).

Compound 94. To a solution of **93** (350 mg, 0.220 mmol) in THF/H_2O 2/1 (9 mL), **36** (81 mg, 0.26 mmol), $CuSO_4 \cdot 5H_2O$ (65 mg, 0.22 mmol), sodium ascorbate (103 mg, 0.520 mmol) and TBTA (14 mg, 0.026 mmol) were added and the reaction mixture was stirred at rt for 1 h. Solvent was removed *in vacuo*, H_2O and CH_2Cl_2 were added, phases were separated, and aqueous phase was extracted by CH_2Cl_2 (x3). Organic phases were collected together,

washed with brine (x1), dried over Na_2SO_4, filtered and concentrated. Crude was purified by flash column chromatography (BGB® Scorpius 25g, 25 mL/min, linear gradient 0 – 8% MeOH in CH_2Cl_2) to yield **94** as a colorless solid (225 mg, 53%). R_f (CH_2Cl_2 + 10% MeOH): 0.3; Mp: 167.2 – 169.4 °C; $[\alpha]_D^{20}$ +16.0 (*c* 0.50, MeOH); IR (neat): 3283 (m), 2923 (m), 1688 (m), 1625 (s), 1519 (s), 1365 (m), 1246 (m), 1151 (s), 704 (m), 575 (m); ^{1}H NMR (500 MHz, CD_3OD): 7.91 (s, 1H), 4.64 (d, $^2J_{H\text{-}H}$ = 15.5 Hz, 1H), 4.54 – 4.49 (m, 3H), 4.39 (d, $^2J_{H\text{-}H}$ = 15.5 Hz, 1H), 4.34 (dd, $^3J_{H\text{-}H}$ = 7.9, 4.5 Hz, 1H), 4.21 (dd, $^3J_{H\text{-}H}$ = 10.1, 4.5 Hz, 1H), 4.12 – 4.01 (m, 4H), 3.94 (dd, $^3J_{H\text{-}H}$ = 9.2, 5.6 Hz, 1H), 3.73 – 3.62 (m, 2H), 3.61 – 3.52 (m, 6H), 3.56 (t, $^3J_{H\text{-}H}$ = 6.6 Hz, 2H), 3.49 (t, $^3J_{H\text{-}H}$ = 6.6 Hz, 2H), 3.47 – 3.34 (m, 2H), 3.23 (ddd, $^3J_{H\text{-}H}$ = 8.6, 6.1, 4.4 Hz, 1H), 3.08 – 2.98 (m, 6H), 2.95 (dd, $^2J_{H\text{-}H}$ = 12.7, $^3J_{H\text{-}H}$ = 5.0 Hz, 1H), 2.86 – 2.76 (m, 1H), 2.71 (d, $^2J_{H\text{-}H}$ = 12.7 Hz, 1H), 2.60 – 2.51 (m, 2H), 2.50 – 2.29 (m, 7H), 2.24 – 2.15 (m, 4H), 2.14 – 1.93 (m, 4H), 1.93 – 1.67 (m, 10H), 1.66 – 1.52 (m, 8H), 1.51 – 1.40 (m, 14H), 1.45 (s, 27H), 1.44 (s, 9H), 1.42 (s, 18H); ^{13}C NMR (126 MHz, CD_3OD): 176.6 (C), 176.5 (C), 176.3 (C), 176.2 (3xC), 175.6 (C), 174.9 (C), 174.9 (C), 173.6 (2xC), 173.3 (C), 166.1 (C), 158.4 (C), 158.3 (C), 158.3 (C), 147.0 (C), 124.7 (CH), 81.9 (C), 81.9 (C), 81.8 (C), 79.8 (C), 79.7 (2xC), 72.3 (CH_2), 71.3 (CH_2), 71.2 (CH_2), 70.7 (CH_2), 63.3 (CH), 61.6 (CH), 57.7 (CH), 57.4 (CH), 57.1 (CH), 57.0 (CH), 57.0 (CH), 56.8 (CH), 56.1 (CH), 50.6 (CH_2), 45.7 (CH_2), 41.3 (2xCH_2), 41.2 (CH_2), 40.6 (CH_2), 40.5 (CH_2), 36.7 (CH_2), 36.1 (CH_2), 33.8 (CH_2), 32.8 (CH_2), 32.7 (CH_2), 32.5 (CH_2), 32.4 (CH_2), 32.2 (CH_2), 32.1 (CH_2), 31.9 (CH_2), 31.7 (CH_2), 30.7 (CH_2), 30.6 (2xCH_2), 30.5 (CH_2), 29.7 (CH_2), 29.4 (CH_2), 28.9 (6xCH_3), 28.9 (3xCH_3), 28.5 (3xCH_3), 28.5 (3xCH_3), 28.5 (3xCH_3), 27.7 (CH_2), 27.3 (CH_2), 27.0 (CH_2), 26.8 (CH_2), 26.6 (CH_2), 26.5 (CH_2), 24.9 (CH_2), 24.9 (CH_2), 24.7 (CH_2).

Compound 95. A solution of **94** (172 mg, 0.089 mmol) in TFA/H_2O/TIPS 95/2.5/2.5 (5 mL) was stirred at rt for 30 min. Solvent was removed *in vacuo*, crude was dissolved in

small amount of MeOH and was precipitated in Et_2O (x3) to yield **95** as a colorless TFA salt (151 mg, 94%). Crude was used in the next reaction without further purification.

Compound 9. To a solution of **95** (40 mg, 22 µmol) in DMF (800 µL), **37** (22 mg, 89 µmol) and DIPEA (31 µL, 178 µmol) were added and reaction mixture was stirred at rt for 1 h. Crude reaction mixture was purified by RP flash chromatography (Biotage® SNAP Ultra 12 g C18, 12 mL/min, linear gradient 20 – 50% CH_3CN in H_2O with 0.1% TFA) to yield **9** as a colorless solid (16.3 mg, 40%). ^{1}H NMR (500 MHz, DMSO-d_6/D_2O/TFA 300/5/1): 7.83 (s, 1H), 4.37 (t, $^3J_{H-H}$ = 6.2 Hz, 2H), 4.34 – 4.28 (m, 2H), 4.26 (d, $^3J_{H-H}$ = 15.2 Hz, 1H), 4.21 – 4.10 (m, 7H), 3.61 (t, $^3J_{H-H}$ = 6.6 Hz, 2H), 3.52 – 3.43 (m, 6H), 3.40 – 3.33 (m, 10H), 3.20 – 3.07 (m, 12H), 3.06 – 2.97 (m, 6H), 2.82 (dd, $^2J_{H-H}$ = 12.5, $^3J_{H-H}$ = 5.1 Hz, 1H), 2.58 (d, $^2J_{H-H}$ = 12.5 Hz, 1H), 2.42 – 2.19 (m, 10H), 2.04 (t, $^3J_{H-H}$ = 7.1 Hz, 2H), 1.99 – 1.86 (m, 3H), 1.85 – 1.56 (m, 9H), 1.56 – 1.43 (m, 8H), 1.42 – 1.34 (m, 8H), 1.33 – 1.19 (m, 10H); ^{13}C NMR (126 MHz, DMSO-d_6): 173.9 (3xC), 172.6 (3xC), 171.9 (C), 171.8 (C), 171.7 (C), 171.5 (2xC), 171.2 (C), 170.3 (3xC), 162.8 (C), 144.6 (C), 123.0 (CH), 70.3 (CH_2), 69.6 (CH_2), 69.4 (CH_2), 69.1 (CH_2), 61.0 (CH), 59.2 (CH), 55.4 (CH), 52.7 (CH), 52.6 (2xCH), 52.4 (2xCH), 52.0 (CH), 51.5 (CH), 51.5 (2xCH), 48.7 (CH_2), 45.5 (CH_2), 42.2 (3xCH_2), 42.1 (3xCH_2), 40.3 (CH_2), 38.8 (CH_2), 38.7 (CH_2), 38.7 (CH_2), 38.7 (CH_2), 38.6 (CH_2), 35.1 (CH_2), 34.2 (CH_2), 32.1 (CH_2), 31.6 (CH_2), 31.3 (2xCH_2), 30.7 (CH_2), 30.5 (CH_2), 30.2 (CH_2), 30.1 (CH_2), 30.1 (CH_2), 29.1 (CH_2), 28.7 (CH_2), 28.6 (2xCH_2), 28.2 (CH_2), 28.0 (CH_2), 26.9 (CH_2), 26.7 (CH_2), 26.6 (CH_2), 26.2 (CH_2), 25.2 (CH_2), 25.0 (CH_2), 22.8 (CH_2), 22. 8 (CH_2), 22.7 (CH_2); HRMS (ESI, +ve) calcd for $C_{74}H_{118}ClN_{17}O_{21}S_7$ ([M + H]$^+$): 1840.6495, found: 1840.6527; LC-MS (ESI, B30): R_t 1.57 min, 1842 (50, [M+H]$^+$), 921 (100, [M+2H]$^{2+}$).

2.1.6. Synthesis of peptide 10

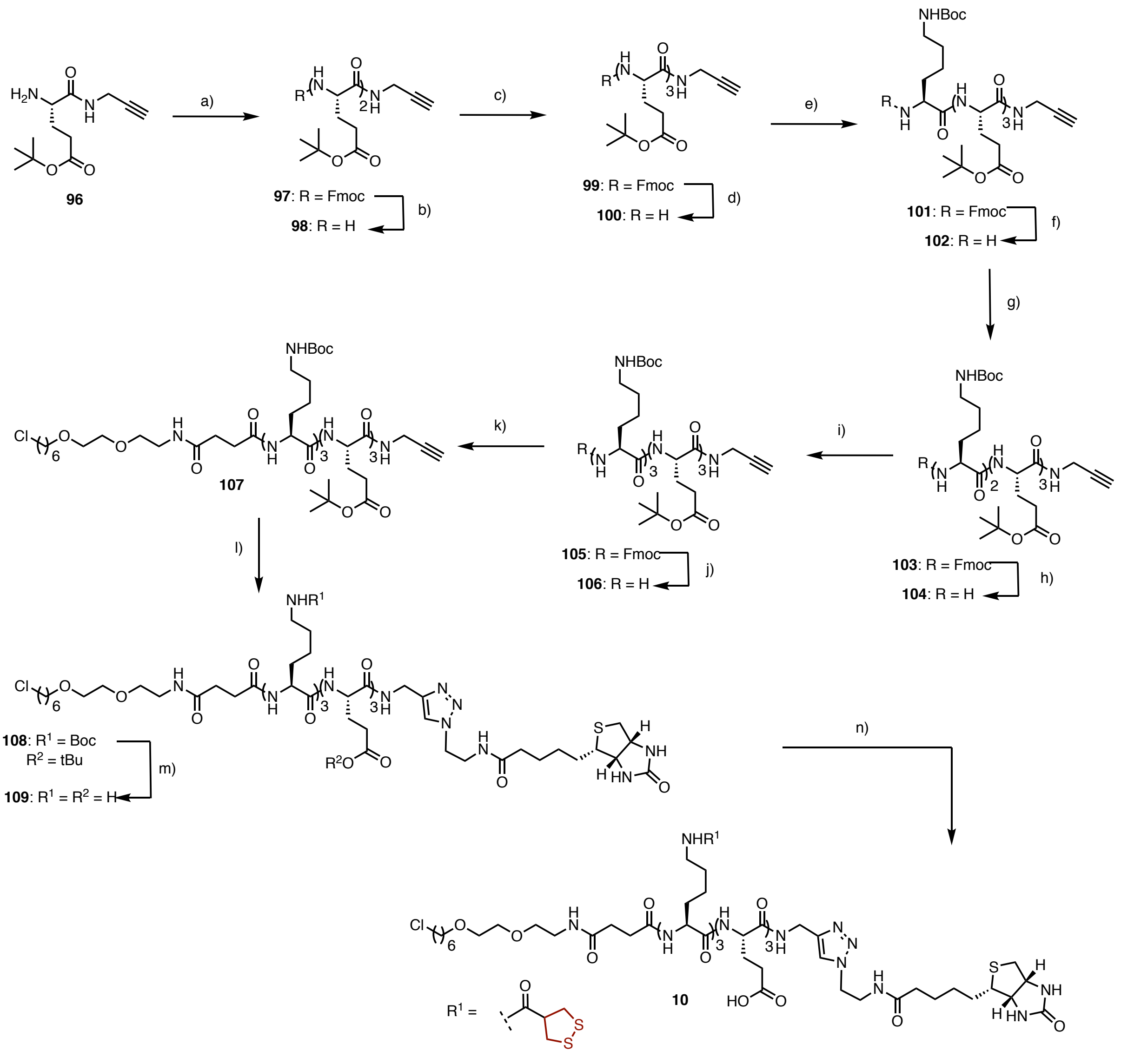

Scheme S6 (a) Fmoc-Glu(O*t*Bu)-OH, EDCI·HCl, DMAP, CH_2Cl_2/DMF, rt, 15 h, 80%; (b) 2 M dimethylamine in THF, rt, 30 min, 97%; (c) Fmoc-Glu(O*t*Bu)-OH, EDCI·HCl, DMAP, CH_2Cl_2/DMF, rt, 15 h, 70%; (d) 2 M dimethylamine in THF, rt, 30 min, 97%; (e) Fmoc-Lys(Boc)-OH, EDCI·HCl, DMAP, CH_2Cl_2/DMF, rt, 15 h, 69%; (f) 2 M dimethylamine in THF, rt, 30 min, 74%; g) Fmoc-Lys(Boc)-OH, EDCI·HCl, DMAP, CH_2Cl_2/DMF, rt, 15 h, 84%; (h) 2 M dimethylamine in THF, rt, 30 min, 92%; i) Fmoc-Lys(Boc)-OH, EDCI·HCl, DMAP, CH_2Cl_2/DMF, rt, 15 h, 85%; (j) 2 M dimethylamine in THF, rt, 30 min, 100%; (k) **35**, EDCI·HCl, DMAP, CH_2Cl_2/DMF, rt, 15 h, 89%; (l) **36**, $CuSO_4 \cdot 5H_2O$, Na-ascorbate, TBTA,

THF/H_2O, rt, 1 h, 60%; (m) $TFA/TIPS/H_2O$ 95/2.5/2.5, rt, 30 min, *quant.*; (n) **37**, DIPEA, DMF, rt, 1 h, 46%.

Compound 96 was prepared following a reported procedure described in ref. S9.

Compound 97. To a solution of **96** (2.40 g, 10.0 mmol) in CH_2Cl_2 (200 mL) were added Fmoc-Glu(O*t*Bu)-OH (5.10 g, 12.0 mmol), EDCI·HCl (2.30 g, 12.0 mmol), and DMAP (381 mg, 3.00 mmol), and the reaction mixture was stirred at rt for 4 h. The organic phase was washed by H_2O (x2) and brine (x1), dried over Na_2SO_4, filtered and concentrated. Crude was purified by flash column chromatography (Biotage® SNAP Ultra 100 g, 50 mL/min, linear gradient 0 – 10% MeOH in CH_2Cl_2) to yield **97** as a colorless solid (5.20 g, 80%). R_f (CH_2Cl_2 + 10% MeOH): 0.7; Mp: 138.2 – 139.4 °C; $[\alpha]_D^{20}$ -24 (*c* 0.50, MeOH); IR (neat): 3281 (m), 2977 (m), 2456 (w), 1724 (s), 1636 (s); 1529 (m), 1447 (m), 1251 (m), 1151 (s), 740 (m), 650 (m); ^{1}H NMR (400 MHz, CD_3OD): 7.79 (d, $^3J_{H\text{-}H}$ = 7.5 Hz, 2H), 7.72 – 7.59 (m, 2H), 7.39 (t, $^3J_{H\text{-}H}$ = 7.5 Hz, 2H), 7.31 (t, $^3J_{H\text{-}H}$ = 7.5 Hz, 2H), 4.46 – 4.32 (m, 3H), 4.23 (t, $^3J_{H\text{-}H}$ = 6.8 Hz, 1H), 4.12 (dd, $^3J_{H\text{-}H}$ = 8.8, 5.5 Hz, 1H), 4.01 – 3.89 (m, 2H), 2.55 (t, $^4J_{H\text{-}H}$ = 2.5 Hz, 1H), 2.39 – 2.22 (m, 4H), 2.15 – 1.98 (m, 2H), 1.97 – 1.80 (m, 2H), 1.45 (s, 9H), 1.40 (s, 9H); ^{13}C NMR (101 MHz, CD_3OD): 174.3 (C), 174.0 (C), 173.9, (C) 173.1 (C), 158.6 (C), 145.3 (C), 145.2 (C), 142.6 (2xC), 128.8 (2xCH), 128.2 (2xCH), 126.2 (2xCH), 120.9 (2xCH), 81.9 (C), 81.8 (C), 80.4 (C), 72.4 (CH), 68.1 (CH_2), 55.9 (CH), 53.7 (CH), 48.4 (CH), 32.7 (CH_2), 32.4 (CH_2), 29.5 (CH_2), 28.4 (3xCH_3), 28.3 (3xCH_3), 28.3 (2xCH_2), 28.2 (CH_2).

Compound 98 was prepared following the general procedure of Fmoc deprotection, starting from **97** (4.10 g, 6.33 mmol) in 20 mL of 2 M dimethylamine in THF, yielding **98** as a colorless solid (2.60 g, 97%).

Compound 99. To a solution of **98** (2.58 g, 6.06 mmol) in CH_2Cl_2/DMF (200 mL/ 10 mL) were added Fmoc-Glu(O*t*Bu)-OH (3.10 g, 7.27 mmol), EDCI·HCl (1.40 g, 7.27 mmol),

and DMAP (279 mg, 2.28 mmol), and the reaction mixture was stirred at rt for 15 h. The organic phase was washed by H_2O (x2) and brine (x1), dried over Na_2SO_4, filtered and concentrated. Crude was purified by flash column chromatography (Claricep® 80 g, 50 mL/min, linear gradient 0 – 8% MeOH in CH_2Cl_2) to yield **99** as a colorless solid (3.6 g, 70%). R_f (CH_2Cl_2 + 10% MeOH): 0.4; Mp: 162.8 – 163.5 °C; $[\alpha]_D^{20}$ -26.6 (*c* 0.50, MeOH); IR (neat): 3311 (w), 2983 (w), 2462 (w), 1727 (s), 1632 (s), 1524 (m), 1448 (m), 1367 (m), 1252 (m), 1149 (s), 740 (m), 620 (m); ^1H NMR (500 MHz, CD_3OD): 7.81 (t, $^3J_{H-H}$ = 7.4, Hz, 2H), 7.69 (d, $^3J_{H-H}$ = 7.4 Hz, 1H), 7.67 (d, $^3J_{H-H}$ = 7.4 Hz, 1H), 7.41 (t, $^3J_{H-H}$ = 7.4 Hz, 2H), 7.33 (t, $^3J_{H-H}$ = 7.4 Hz, 2H), 4.45 – 4.33 (m, 4H), 4.25 (t, $^3J_{H-H}$ = 6.9 Hz, 1H), 4.14 (dd, $^3J_{H-H}$ = 8.8, 5.5 Hz, 1H), 4.00 (dd, $^2J_{H-H}$ = 17.5, $^4J_{H-H}$ = 2.6 Hz, 1H), 3.95 (dd, $^2J_{H-H}$ = 17.5, $^4J_{H-H}$ = 2.6 Hz, 1H), 2.58 (t, $^4J_{H-H}$ = 2.5 Hz, 1H), 2.42 – 2.24 (m, 6H), 2.16 – 2.04 (m, 3H), 1.99 – 1.86 (m, 3H), 1.47 (s, 9H), 1.43 (s, 9H), 1.42 (s, 9H); ^{13}C NMR (126 MHz, CD_3OD): 174.6 (C), 174.1 (C), 173.9 (C), 173.8 (C), 173.5 (C), 173.1 (C), 158.6 (C), 145.3 (C), 145.2 (C), 142.6 (C), 142.6 (C), 128.8 (CH), 128.8 (CH), 128.2 (2xCH), 126.3 (CH), 126.3 (CH), 120.9 (2xCH), 81.9 (C), 81.8 (C), 80.4 (C), 72.3 (CH), 68.1 (CH_2), 56.0 (CH), 54.3 (CH), 53.9 (CH), 48.4 (CH), 32.7 (CH_2), 32.6 (CH_2), 32.5 (CH_2), 29.5 (CH_2), 28.4 (3xCH_3), 28.4 (3xCH_3), 28.3 (3xCH_3), 28.3 (CH_2), 28.2 (CH_2), 27.9 (CH_2).

Compound 100 was prepared following the general procedure of Fmoc deprotection, starting from **99** (2.80 g, 3.36 mmol) in 15 mL of 2 M dimethylamine in THF, yielding **100** as a colorless solid (2.0 g, 97%).

Compound 101. To a solution of **100** (1.95 g, 3.20 mmol) in CH_2Cl_2/DMF (100 mL/ 10 mL) were added Fmoc-Lys(Boc)-OH (1.80 g, 3.84 mmol), EDCI·HCl (736 mg, 3.84 mmol), and DMAP (122 mg, 1.00 mmol), and the reaction mixture was stirred at rt for 15 h. Solvents were removed *in vacuo*. EtOAc was added and the organic phase was washed by 10% citric acid solution (x2), H_2O (x2) and brine (x1), dried over Na_2SO_4, filtered and concentrated.

Crude was purified by flash column chromatography (Claricep® 80 g, 50 mL/min, linear gradient 0 – 8% MeOH in CH_2Cl_2) to yield **101** as a colorless solid (2.33 g, 69%). R_f (CH_2Cl_2 + 10% MeOH): 0.4; Mp: 163.2 – 164.3 °C; $[\alpha]_D^{20}$ -23.5 (*c* 0.50, MeOH); IR (neat): 3284 (w), 2974 (w), 2436 (w), 1726 (s), 1628 (s), 1523 (m), 1451 (m), 1367 (m), 1250 (m), 1152 (s), 741 (m), 621 (m); ^{1}H NMR (400 MHz, CD_3OD): 7.80 (d, $^3J_{H-H}$ = 7.4 Hz, 2H), 7.68 (d, $^3J_{H-H}$ = 7.4 Hz, 2H), 7.39 (t, $^3J_{H-H}$ = 7.4 Hz, 2H), 7.31 (t, $^3J_{H-H}$ = 7.4 Hz, 2H), 4.47 – 4.27 (m, 5H), 4.23 (t, $^3J_{H-H}$ = 6.7 Hz, 1H), 4.03 (dd, $^3J_{H-H}$ = 8.2, 5.3 Hz, 1H), 3.97 (dd, $^2J_{H-H}$ = 17.4, $^4J_{H-H}$ = 2.6 Hz, 1H), 3.93 (dd, $^2J_{H-H}$ = 17.4, $^4J_{H-H}$ = 2.6 Hz, 1H), 3.04 (t, $^3J_{H-H}$ = 6.8 Hz, 2H), 2.56 (t, $^4J_{H-H}$ = 2.6 Hz, 1H), 2.43 – 2.22 (m, 6H), 2.18 – 2.03 (m, 3H), 1.99 – 1.85 (m, 3H), 1.82 – 1.62 (m, 2H), 1.54 – 1.41 (m, 4H), 1.43 (s, 9H), 1.42 (s, 9H), 1.41 (s, 9H), 1.41 (s, 9H); ^{13}C NMR (101 MHz, CD_3OD): 175.7 (C), 174.1 (2xC), 173.9 (C), 173.8 (C), 173.6 (C), 173.2 (C), 159.0 (C), 158.6 (C), 145.3 (C), 145.2 (C), 142.6 (2xC), 128.8 (2xCH), 128.2 (2xCH), 126.3 (2xCH), 121.0 (2xCH), 81.9 (C), 81.8 (C), 81.8 (C), 80.4 (C), 79.9 (C), 72.3 (CH), 68.1 (CH_2), 57.2 (CH), 54.8 (CH), 54.4 (CH), 54.0 (CH), 48.4 (CH), 41.0 (CH_2), 32.7 (2xCH_2), 32.6 (CH_2), 32.4 (CH_2), 30.6 (CH_2), 29.6 (CH_2), 28.8 (3xCH_3), 28.4 (9xCH_3), 28.2 (CH_2), 28.0 (CH_2), 27.6 (CH_2), 24.1 (CH_2).

Compound 102 was prepared following the general procedure of Fmoc deprotection, starting from **101** (2.30 g, 2.16 mmol) in 10 mL of 2 M dimethylamine in THF, yielding **102** as a colorless solid (1.44 g, 74%).

Compound 103. To a solution of **102** (1.30 g, 1.55 mmol) in CH_2Cl_2/DMF (100 mL/ 10 mL) were added Fmoc-Lys(Boc)-OH (871 mg, 1.86 mmol), EDCI·HCl (356 mg, 1.86 mmol), and DMAP (57 mg, 0.47 mmol), and the reaction mixture was stirred at rt for 15 h. The organic phase was washed by 10% citric acid solution (x2), H_2O (x2) and brine (x1), dried over Na_2SO_4, filtered and concentrated. Crude was purified by flash column chromatography (Biotage® SNAP Ultra 50 g, 50 mL/min, linear gradient 0 – 10% MeOH in CH_2Cl_2) to yield

103 as a colorless solid (1.7 g, 85%). R_f (CH_2Cl_2 + 10% MeOH): 0.4; Mp: 188.2 – 189.4 °C; $[\alpha]_D^{20}$ -23.8 (*c* 0.50, MeOH); IR (neat): 3271 (w), 2934 (w), 1715 (s), 1625 (s), 1513 (s), 1449 (m), 1366 (s), 1248 (s), 1150 (s), 846 (w), 740 (m), 618 (m); ^{1}H NMR (400 MHz, CD_3OD): 7.80 (d, $^3J_{H-H}$ = 7.4 Hz, 2H), 7.68 (d, $^3J_{H-H}$ = 7.4 Hz, 2H), 7.40 (t, $^3J_{H-H}$ = 7.4 Hz, 2H), 7.31 (t, $^3J_{H-H}$ = 7.4 Hz, 2H), 4.47 (dd, $^3J_{H-H}$ = 10.7, 6.8 Hz, 1H), 4.38 (dd, $^3J_{H-H}$ = 10.7, 6.8 Hz, 1H), 4.35 – 4.21 (m, 3H), 4.23 (t, $^3J_{H-H}$ = 6.8 Hz, 2H), 4.04 (dd, $^3J_{H-H}$ = 8.4, 5.7 Hz, 1H), 4.02 – 3.90 (m, 2H), 3.10 – 2.97 (m, 4H), 2.56 (t, $^4J_{H-H}$ = 2.5 Hz, 1H), 2.44 – 2.22 (m, 6H), 2.20 – 2.04 (m, 3H), 2.04 – 1.87 (m, 3H), 1.84 – 1.63 (m, 4H), 1.52 – 1.40 (m, 8H), 1.43 (s, 18H), 1.41 (s, 9H), 1.41 (s, 9H), 1.41 (s, 9H) ; ^{13}C NMR (101 MHz, CD_3OD): 176.0 (C), 175.1 (C), 174.2 (C), 173.9 (2xC), 173.9 (C), 173.8 (C), 173.7 (C), 173.2 (C), 159.0 (C), 158.5 (C), 158.5 (C), 145.2 (C), 145.2 (C), 142.6 (2xC), 128.8 (2xCH), 128.2 (2xCH), 126.3 (CH), 126.2 (CH), 121.0 (2xCH), 81.9 (C), 81.8 (C), 81.7 (C), 80.4 (C), 79.9 (2xC), 72.3 (CH), 68.1 (CH_2), 57.3 (CH), 55.9 (CH), 55.1 (CH), 54.6 (CH), 54.0 (CH), 48.4 (CH), 41.1 (CH_2), 41.0 (CH_2), 32.8 (CH_2), 32.7 (CH_2), 32.7 (CH_2), 32.4 (CH_2), 32.0 (CH_2), 30.6 (2xCH_2), 30.5 (CH_2), 29.6 (CH_2), 28.9 (3xCH_3), 28.8 (3xCH_3), 28.4 (9xCH_3), 28.2 (CH_2), 28.0 (CH_2), 27.7 (CH_2), 24.3 (CH_2), 24.2 (2xCH_2).

Compound 104 was prepared following the general procedure of Fmoc deprotection, starting from **103** (1.60 g, 1.24 mmol) in 10 mL of 2 M dimethylamine in THF, yielding **104** as a colorless solid (1.22 g, 92%).

Compound 105. To a solution of **104** (1.2 g, 1.1 mmol) in CH_2Cl_2/DMF (100 mL/ 10 mL) were added Fmoc-Lys(Boc)-OH (609 mg, 1.30 mmol), EDCI·HCl (249 mg, 1.30 mmol), and DMAP (40 mg, 0.33 mmol), and the reaction mixture was stirred at rt for 15 h. The organic phase was washed by 10% citric acid solution (x2), H_2O (x2) and brine (x1), 5% LiCl (x2), dried over Na_2SO_4, filtered and concentrated. Crude was purified by flash column chromatography (Claricep® 80 g, 50 mL/min, linear gradient 0 – 10% MeOH in CH_2Cl_2) to

yield **105** as a colorless solid (1.42 g, 85%). R_f (CH_2Cl_2 + 10% MeOH): 0.5; Mp: 201.7 – 203.0 °C; $[\alpha]_D^{20}$ -17.3 (*c* 0.50, MeOH); IR (neat): 3295 (w), 2981 (w), 1692 (s), 1621 (s), 1505 (s), 1246 (s), 1149 (s), 733 (m), 618 (m); ^{1}H NMR (500 MHz, CD_3OD): 7.81 (d, $^3J_{H-H}$ = 7.5 Hz, 2H), 7.69 (d, $^3J_{H-H}$ = 7.5 Hz, 1H),), 7.67 (d, $^3J_{H-H}$ = 7.5 Hz, 1H), 7.41 (t, $^3J_{H-H}$ = 7.5 Hz, 2H), 7.33 (t, $^3J_{H-H}$ = 7.5 Hz, 2H), 4.55 – 4.42 (m, 1H), 4.38 – 4.33 (m, 1H), 4.33 (dd, $^3J_{H-H}$ = 9.7, 4.7 Hz, 1H), 4.26 (t, $^3J_{H-H}$ = 6.8 Hz, 2H), 4.23 – 4.15 (m, 2H), 4.15 – 4.09 (m, 1H), 4.06 – 3.99 (m, 1H), 4.01 – 3.89 (m, 2H), 3.07 – 2.94 (m, 6H), 2.55 (t, $^4J_{H-H}$ = 2.5 Hz, 1H), 2.46 – 2.25 (m, 6H), 2.19 – 1.89 (m, 6H), 1.86 – 1.63 (m, 6H), 1.57 – 1.37 (m, 13H), 1.43 (s, 18H), 1.43 (s, 9H), 1.42 (s, 9H), 1.41 (s, 18H); ^{13}C NMR (126 MHz, CD_3OD): 176.2 (C), 175.5 (2xC), 174.7 (C), 174.0 (C), 173.9 (C), 173.8 (C), 173.8 (C), 173.3 (C), 159.2 (C), 158.6 (C), 158.5 (C), 158.4 (C), 145.2 (C), 145.1 (C), 142.7 (C), 142.6 (C), 128.9 (2xCH), 128.3 (CH), 128.3 (CH), 126.3 (CH), 126.2 (CH), 121.1 (2xCH), 81.8 (C), 81.8 (C), 81.7 (C), 80.5 (C), 79.9 (C), 79.9 (2xC), 72.2 (CH), 68.4 (CH_2), 57.8 (CH), 56.5 (2xCH), 55.5 (CH), 54.9 (CH), 54.2 (CH), 48.3 (CH), 41.1 (CH_2), 41.1 (CH_2), 41.0 (CH_2), 32.8 (2xCH_2), 32.7 (CH_2), 32.3 (CH_2), 31.9 (CH_2), 31.8 (CH_2), 30.6 (2xCH_2), 30.5 (CH_2), 29.6 (CH_2), 28.9 (9xCH_3), 28.5 (6xCH_3), 28.4 (3xCH_3), 28.1 (CH_2), 27.9 (CH_2), 27.5 (CH_2), 24.3 (2xCH_2), 24.2 (CH_2).

Compound 106 was prepared following the general procedure of Fmoc deprotection, starting from **105** (700 mg, 0.46 mmol) in 10 mL of 2 M dimethylamine in THF, yielding **106** as a colorless solid (600 mg, 100%).

Compound 107. To a solution of **106** (600 mg, 1.1 mmol) in CH_2Cl_2/DMF (40 mL/ 5 mL) were added **35** (178 mg, 0.55 mmol), EDCI·HCl (105 mg, 0.55 mmol), and DMAP (17 mg, 0.14 mmol), and the reaction mixture was stirred at rt for 15 h. The organic phase was washed with H_2O (x2) and brine (x1), 5% LiCl (x2), dried over Na_2SO_4, filtered and concentrated. Crude was purified by flash column chromatography (Scorpius® 25 g, 25 mL/min, linear gradient 0 – 10% MeOH in CH_2Cl_2) to yield **107** as a colorless solid (655 mg,

89%).R_f (CH_2Cl_2 + 10% MeOH): 0.4; Mp: decomposition > 220 °C; $[\alpha]_D^{20}$ +3.2 (*c* 0.50, MeOH); IR (neat): 3270 (w), 2934 (w), 1692 (m), 1621 (s), 1518 (s), 1365 (m), 1248 (m), 1148 (s), 697 (m), 648 (m); ^{1}H NMR (400 MHz, CD_3OD): 4.32 (dd, $^3J_{H\text{-}H}$ = 10.2, 4.4 Hz, 1H), 4.21 (t, $^3J_{H\text{-}H}$ = 7.3 Hz, 1H), 4.13 – 4.02 (m, 4H), 3.99 (dd, $^2J_{H\text{-}H}$ = 17.3, $^4J_{H\text{-}H}$ = 2.5 Hz, 1H), 3.95 (dd, $^2J_{H\text{-}H}$ = 17.3, $^4J_{H\text{-}H}$ = 2.5 Hz, 1H), 3.63 – 3.53 (m, 8H), 3.49 (t, $^3J_{H\text{-}H}$ = 6.6 Hz, 2H), 3.46 – 3.37 (m, 2H), 3.10 – 2.96 (m, 6H), 2.87 – 2.73 (m, 1H), 2.62 – 2.49 (m, 3H), 2.52 (t, $^4J_{H\text{-}H}$ = 2.5 Hz, 1H), 2.48 – 2.30 (m, 6H), 2.23 – 1.99 (m, 6H), 1.99 – 1.70 (m, 8H), 1.67 – 1.57 (m, 2H), 1.55 – 1.41 (m, 16H), 1.44 (s, 18H), 1.44 (s, 36H); ^{13}C NMR (101 MHz, CD_3OD): 176.8 (C), 176.8 (C), 176.7 (C), 176.6 (C), 175.6 (C), 174.9 (C), 174.5 (C), 173.9 (C), 173.8 (C), 173.7 (C), 173.6 (C), 158.5 (C), 158.4 (C), 158.4 (C), 81.6 (C) , 81.6 (C), 81.5 (C), 80.6 (C), 79.9 (2xC), 79.8 (C), 72.3 (CH), 72.0 (CH_2), 71.3 (CH_2), 71.2 (CH_2), 70.5 (CH_2), 57.9 (CH), 57.5 (2xCH), 56.6 (CH), 55.6 (CH), 54.5 (CH), 45.7 (CH_2), 41.2 (CH_2), 41.1 (CH_2), 41.0 (CH_2), 40.7 (CH_2), 33.8 (CH_2), 33.0 (CH_2), 32.9 (CH_2), 32.8 (CH_2), 32.2 (CH_2), 32.0 (CH_2), 31.8 (CH_2), 31.5 (CH_2), 31.1 (CH_2), 30.7 (CH_2), 30.6 (CH_2), 30.6 (CH_2), 30.6 (CH_2), 30.6 (CH_2), 29.6 (CH_2), 28.9 (6xCH_3), 28.9 (3xCH_3), 28.5 (3xCH_3), 28.5 (3xCH_3), 28.5 (3xCH_3), 27.9 (CH_2), 27.7 (CH_2), 27.7 (CH_2), 27.2 (CH_2), 26.5 (CH_2), 24.7 (CH_2), 24.6 (CH_2), 24.4 (CH_2).

Compound 108. To a solution of **107** (515 mg, 0.322 mmol) in THF/H_2O 2/1 (9 mL), **36** (119 mg, 0.381 mmol), $CuSO_4\cdot 5H_2O$ (80 mg, 0.32 mmol), sodium ascorbate (127 mg, 0.641 mmol) and TBTA (17 mg, 0.032 mmol) were added and the reaction mixture was stirred at rt for 1 h. Solvent was removed *in vacuo*, H_2O and CH_2Cl_2 were added, phases were separated, and aqueous phase was extracted by CH_2Cl_2 (x3). Organic phases were collected together, washed with brine (x1), dried over Na_2SO_4, filtered and concentrated. Crude was purified by flash column chromatography (BGB® Scorpius 25g, 25 mL/min, linear gradient 0 – 10% MeOH in CH_2Cl_2) to yield **108** as a sticky oil (370 mg, 60%). R_f (CH_2Cl_2 + 10% MeOH): 0.2; $[\alpha]_D^{20}$ +15.9 (*c* 0.50, MeOH); IR (neat): 3260 (w), 2935 (w), 2310 (w), 1688 (s), 1619 (s), 1523

(s), 1431 (m), 1364 (m), 1279 (m), 1140 (m), 1029 (m), 693 (m), 615 (m); ^{1}H NMR (400 MHz, CD_3OD): 7.95 (s, 1H), 4.64 (d, $^2J_{H-H}$ = 15.6 Hz, 1H), 4.56 – 4.47 (m, 3H), 4.39 (d, $^2J_{H-H}$ = 15.6 Hz, 1H), 4.34 (dd, $^3J_{H-H}$ = 7.9, 4.4 Hz, 1H), 4.27 (dd, $^3J_{H-H}$ = 10.2, 4.5 Hz, 1H), 4.14 (dd, $^3J_{H-H}$ = 9.6, 5.1 Hz, 1H), 4.09 – 4.00 (m, 2H), 4.00 – 3.93 (m, 2H), 3.74 – 3.53 (m, 12H), 3.49 (t, $^3J_{H-H}$ = 6.6 Hz, 2H), 3.47 – 3.32 (m, 2H), 3.22 (ddd, $^3J_{H-H}$ = 8.6, 6.2, 4.4 Hz, 1H), 3.10 – 2.98 (m, 6H), 2.94 (dd, $^2J_{H-H}$ = 12.7, $^3J_{H-H}$ = 5.0 Hz, 1H), 2.87 – 2.76 (m, 1H), 2.70 (d, $^2J_{H-H}$ = 12.7 Hz, 1H), 2.64 – 2.28 (m, 9H), 2.25 – 2.02 (m, 8H), 2.18 (t, $^3J_{H-H}$ = 7.4 Hz, 2H), 2.00 – 1.67 (m, 10H), 1.66 – 1.26 (m, 32H), 1.44 (s, 18H), 1.44 (s, 18H), 1.43 (s, 9H), 1.42 (s, 9H); ^{13}C NMR (101 MHz, CD_3OD): 177.1 (C), 177.0 (C), 176.8 (C), 176.6 (C), 176.5 (C), 175.9 (C), 175.1 (C), 175.0 (C), 174.3 (C), 173.8 (C), 173.7 (C), 173.5 (C), 166.1 (C), 158.5 (C), 158.4 (C), 158.4 (C), 146.7 (C), 124.8 (CH), 81.7 (C), 81.6 (C), 81.5 (C), 79.9 (2xC), 79.8 (C), 72.3 (CH_2), 71.4 (CH_2), 71.2 (CH_2), 70.5 (CH_2), 63.3 (CH), 61.7 (CH), 58.2 (CH), 57.8 (CH), 57.6 (CH), 57.1 (CH), 56.9 (CH), 56.3 (CH), 55.2 (CH), 50.7 (CH_2), 45.7 (CH_2), 41.2 (CH_2), 41.1 (2xCH_2), 41.0 (CH_2), 40.7 (CH_2), 40.5 (CH_2), 36.7 (CH_2), 36.0 (CH_2), 33.8 (CH_2), 33.0 (CH_2), 33.0 (CH_2), 32.8 (CH_2), 32.2 (CH_2), 32.0 (CH_2), 31.8 (CH_2), 31.5 (CH_2), 31.0 (CH_2), 30.7 (CH_2), 30.6 (CH_2), 30.6 (CH_2), 30.6 (CH_2), 29.6 (CH_2), 29.4 (CH_2), 28.9 (6xCH_3), 28.9 (3xCH_3), 28.6 (6xCH_3), 28.5 (3xCH_3), 27.7 (2xCH_2), 27.6 (CH_2), 27.1 (CH_2), 26.7 (CH_2), 26.5 (CH_2), 24.7 (CH_2), 24.6 (CH_2), 24.5 (CH_2).

Compound 109. A solution of **108** (120 mg, 0.627 mmol) in TFA/H_2O/TIPS 95/2.5/2.5 (1 mL) was stirred at rt for 30 min. Solvent was removed *in vacuo*, crude was dissolved in small amount of MeOH and was precipitated in Et_2O (x3) to yield **109** as a colorless TFA salt (110 mg, 98%). Crude was used in the next reaction without further purification.

Compound 10. To a solution of **109** (40 mg, 22 μmol) in DMF (1.6 mL), **37** (22 mg, 89 μmol) and DIPEA (31 μL, 178 μmol) were added and reaction mixture was stirred at rt for 1 h. Crude reaction mixture was purified by RP flash chromatography (Biotage® SNAP Ultra

12 g C18, 12 mL/min, linear gradient 20 – 60% CH_3CN in H_2O with 0.1% TFA) to yield **10** as a colorless solid (18.9 mg, 46%). ^{1}H NMR (500 MHz, DMSO-d_6/D_2O/TFA 300/5/1): 7.85 (s, 1H), 4.36 (t, $^3J_{H\text{-}H}$ = 6.1 Hz, 2H), 4.36 – 4.23 (m, 3H), 4.22 – 4.16 (m, 3H), 4.15 – 4.04 (m, 4H), 3.61 (t, $^3J_{H\text{-}H}$ = 6.6 Hz, 2H), 3.52 – 3.43 (m, 6H), 3.41 – 3.32 (m, 10H), 3.21 – 3.07 (m, 12H), 3.06 – 3.00 (m, 6H), 2.82 (dd, $^2J_{H\text{-}H}$ = 12.5, $^3J_{H\text{-}H}$ = 5.1 Hz, 1H), 2.58 (d, $^2J_{H\text{-}H}$ = 12.5 Hz, 1H), 2.42 – 2.16 (m, 10H), 2.04 (t, $^3J_{H\text{-}H}$ = 7.5 Hz, 2H), 1.97 – 1.83 (m, 3H), 1.83 – 1.73 (m, 3H), 1.73 – 1.56 (m, 7H), 1.55 – 1.43 (m, 7H), 1.43 – 1.34 (m, 8H), 1.33 – 1.24 (m, 10H); ^{13}C NMR (126 MHz, DMSO-d_6/D_2O 60/1): 174.0 (C), 173.9 (C), 173.8 (C), 172.8 (C), 172.6 (2xC), 172.2 (C), 171.9 (2xC), 171.2 (C), 171.0 (C), 170.9 (C), 170.3 (C), 170.3 (C), 170.2 (C), 162.8 (C), 144.6 (C), 123.0 (CH), 70.3 (CH_2), 69.6 (CH_2), 69.4 (CH_2), 69.0 (CH_2), 60.9 (CH), 59.1 (CH), 55.4 (CH), 53.5 (CH), 53.3 (CH), 52.8 (CH), 52.1 (CH), 52.0 (2xCH), 51.5 (3xCH), 48.7 (CH_2), 45.4 (CH_2), 42.1 (6xCH_2), 39.6 (CH_2), 38.8 (CH_2), 38.7 (2xCH_2), 38.6 (CH_2), 38.6 (CH_2), 35.0 (CH_2), 34.3 (CH_2), 32.1 (CH_2), 31.0 (CH_2), 31.0 (CH_2), 30.6 (2xCH_2), 30.5 (CH_2), 30.1 (CH_2), 30.1 (CH_2), 30.0 (CH_2), 29.1 (CH_2), 28.7 (CH_2), 28.6 (2xCH_2), 28.1 (CH_2), 28.0 (CH_2), 27.1 (CH_2), 27.1 (CH_2), 27.0 (CH_2), 26.2 (CH_2), 25.2 (CH_2), 25.0 (CH_2), 23.1 (CH_2), 22.9 (CH_2), 22.8 (CH_2); HRMS (ESI, +ve) calcd for $C_{74}H_{118}ClN_{17}O_{21}S_7$ ($[M + H]^+$): 1840.6495, found: 1840.6439; LC-MS (ESI, B30): R_t 1.73 min, 1842 (50, $[M+H]^+$), 921 (100, $[M+2H]^{2+}$).

2.1.7. Synthesis of peptide 11

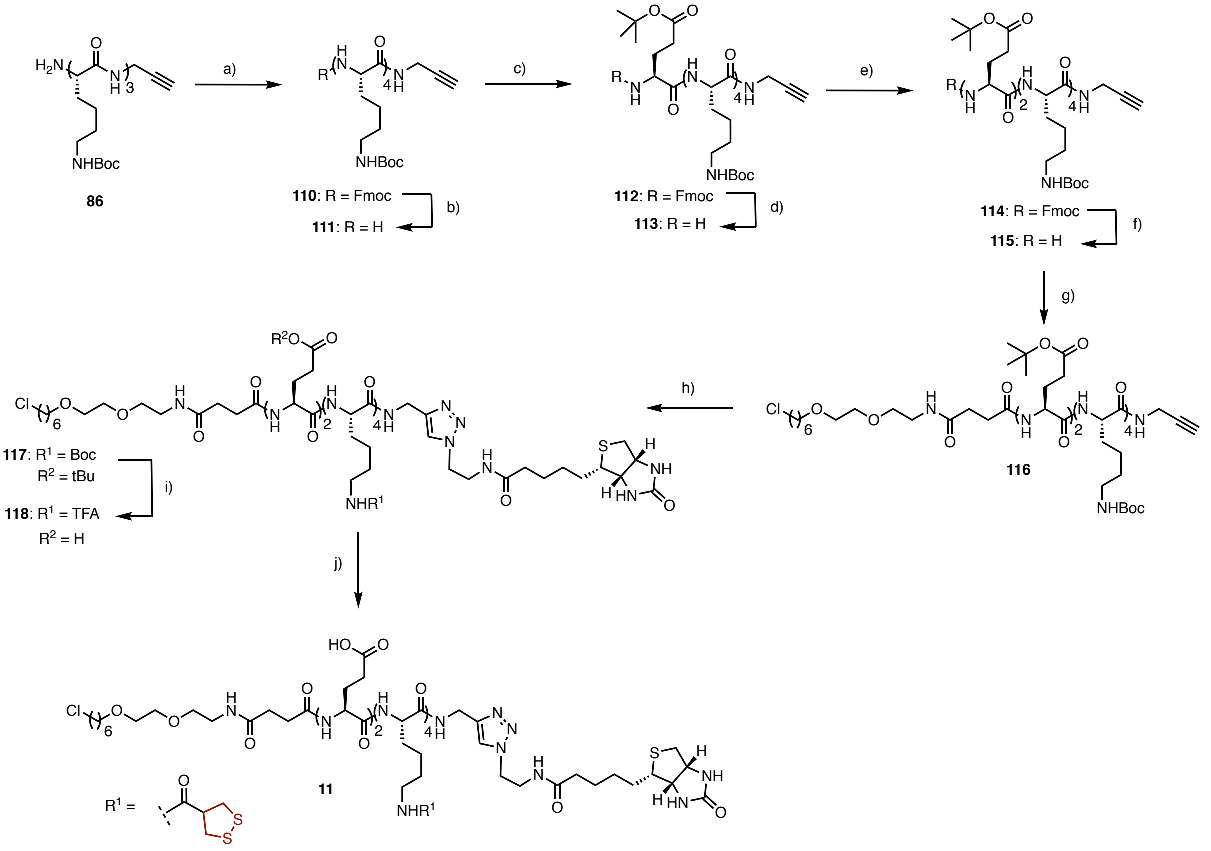

Scheme S7 (a) Fmoc-Lys(Boc)-OH, HATU, DIPEA, DMF, rt, 30 min, 92%; (b) 2 M dimethylamine in THF, rt, 30 min, 92%; (c) Fmoc-Glu(O*t*Bu)-OH, HATU, DIPEA, DMF, rt, 30 min, 78%; (d) 2 M dimethylamine in THF, rt, 30 min, 99%; (e) Fmoc-Glu(O*t*Bu)-OH, HATU, DIPEA, DMF, rt, 30 min, 90%; (f) 2 M dimethylamine in THF, rt, 30 min, 99%; (g) **35**, HATU, DIPEA, DMF, rt, 30 min, 84%; (h) **36**, $CuSO_4{\cdot}5H_2O$, Na-ascorbate, TBTA, THF/H_2O, rt, 1 h, 69%; (i) TFA/TIPS/H_2O 95/2.5/2.5, rt, 30 min, 87%; (j) **37**, DIPEA, DMF, rt, 1 h, 39%.

Compound 110. To a solution of **86** (700 mg, 0.946 mmol) in dry DMF (3 mL), was added a 30 seconds-premixed solution of HATU (432 mg, 1.14 mmol), Fmoc-Lys(Boc)-OH (532 mg, 1.14 mmol) and DIPEA (198 µL, 1.14 mmol) in dry DMF (3 mL). Reaction mixture was stirred at rt for 30 min. Reaction mixture was diluted with EtOAc and washed with

$NaHCO_3$ sat. aq. solution (x3), 10% citric acid solution (x3), water (x1), 5% LiCl solution (x2) and brine. Organic phase was then dried with Na_2SO_4, filtered and concentrated. Crude was dissolved in a minimal amount of CH_2Cl_2 and precipitated in pentane (x3). Crude was then purified by flash column chromatography (BGB® Scorpius 25 g SiO_2 col., 40 mL/min, linear gradient 0 – 8% MeOH in CH_2Cl_2) to yield **110** as a colorless solid (1.03 g, 92%). R_f (CH_2Cl_2 + 10% MeOH): 0.5; Mp: 183.7 – 184.8 °C; $[\alpha]_D^{20}$ – 17.9 (*c* 0.50, MeOH); IR (neat): 3300 (w), 2935 (w), 1683 (s), 1623 (s), 1529 (s), 1450 (m), 1363 (m), 1248 (s), 1165 (s), 737 (m), 603 (m); ^{1}H NMR (400 MHz, CD_3OD): 7.80 (d, $^3J_{H\text{-}H}$ = 7.5 Hz, 2H), 7.67 (d, $^3J_{H\text{-}H}$ = 7.5 Hz, 2H), 7.39 (t, $^3J_{H\text{-}H}$ = 7.5 Hz, 2H), 7.32 (t, $^3J_{H\text{-}H}$ = 7.5 Hz, 2H), 4.49 – 4.18 (m, 6H), 4.06 (br s, 1H), 3.99 (d, $^2J_{H\text{-}H}$ = 17.6 Hz, 1H), 3.92 (d, $^2J_{H\text{-}H}$ = 17.6 Hz, 1H), 3.12 – 2.89 (m, 8H), 2.58 (br s, 1H), 1.88 – 1.60 (m, 8H), 1.59 – 1.27 (m, 16H), 1.42 (s, 27H), 1.40 (s, 9H); ^{13}C NMR (101 MHz, CD_3OD): 175.3 (C), 174.4 (C), 174.1 (C), 173.8 (C), 158.7 (C), 158.5 (C), 158.5 (3xC), 145.3 (C), 145.2 (C), 142.6 (2xC), 128.8 (2xCH), 128.2 (2xCH), 126.3 (2xCH), 121.0 (2xCH), 80.4 (C), 79.9 (4xC), 72.4 (CH), 68.1 (CH_2), 56.9 (CH), 54.9 (2xCH), 54.5 (CH), 48.4 (CH), 41.2 (4xCH_2), 32.7 (2xCH_2), 32.5 (2xCH_2), 30.6 (CH_2), 30.5 (3xCH_2), 29.5 (CH_2), 28.8 (12xCH_3), 24.2 (CH_2), 24.1 (3xCH_2).

Compound 111 was prepared following the general procedure of Fmoc deprotection, starting from **110** (1.03 g, 0.865 mmol) in 8 mL of 2 M dimethylamine in THF, yielding **111** as a colorless solid (774 mg, 92%).

Compound 112. To a solution of **111** (460 mg, 0.475 mmol) in dry DMF (2 mL), was added a 30 seconds-premixed solution of HATU (217 mg, 0.570 mmol), Fmoc-Glu(O*t*Bu)-OH (242 mg, 0.570 mmol) and DIPEA (99 µL, 0.57 mmol) in dry DMF (3 mL). Reaction mixture was stirred at rt for 30 min. Reaction mixture was diluted with EtOAc and washed with $NaHCO_3$ sat. aq. solution (x3), 10% citric acid solution (x3), water (x1), 5% LiCl solution (x2) and brine. Organic phase was then dried with Na_2SO_4, filtered and concentrated. Crude was

dissolved in a minimal amount of CH_2Cl_2 and precipitated in pentane (x3). Crude was then purified by flash column chromatography (BGB® Scorpius 25 g SiO_2 col., 40 mL/min, linear gradient 0 – 8% MeOH in CH_2Cl_2) to yield **112** as a colorless solid (512 mg, 78%). R_f (CH_2Cl_2 + 10% MeOH): 0.5; Mp: 201.8 – 202.2 °C; $[\alpha]_D^{20}$ – 22.9 (*c* 0.50, MeOH); IR (neat): 3279 (m), 2930 (m), 1719 (m), 1685 (s), 1630 (s), 1523 (s), 1449 (m), 1365 (m), 1249 (s), 1167 (s), 738 (m), 650 (m); ^{1}H NMR (500 MHz, CD_3OD): 7.81 (d, $^3J_{H-H}$ = 7.4 Hz, 2H), 7.67 (d, $^3J_{H-H}$ = 7.4 Hz, 2H), 7.40 (t, $^3J_{H-H}$ = 7.4 Hz, 2H), 7.32 (t, $^3J_{H-H}$ = 7.4 Hz, 2H), 4.49 – 4.40 (m, 1H), 4.39 – 4.31 (m, 1H), 4.31 – 4.20 (m, 5H), 4.14 – 4.06 (m, 1H), 3.99 (dd, $^2J_{H-H}$ = 17.5, $^4J_{H-H}$ = 2.5 Hz, 1H), 3.93 (dd, $^2J_{H-H}$ = 17.5, $^4J_{H-H}$ = 2.5 Hz, 1H), 3.08 – 2.96 (m, 8H), 2.58 (t, $^4J_{H-H}$ = 2.5 Hz, 1H), 2.35 (t, $^3J_{H-H}$ = 7.6 Hz, 2H), 2.09 – 2.00 (m, 1H), 1.96 – 1.87 (m, 1H), 1.86 – 1.76 (m, 4H), 1.74 – 1.65 (m, 4H), 1.56 – 1.24 (m, 16H), 1.45 (s, 9H), 1.42 (s, 27H), 1.40 (s, 9H); ^{13}C NMR (126 MHz, CD_3OD): 174.9 (C), 174.7 (C), 174.6 (C), 174.2 (C), 173.9 (C), 173.8 (C), 158.7 (C), 158.5 (C), 145.2 (C), 145.2 (C), 142.6 (2xC), 128.9 (2xCH), 128.2 (2xCH), 126.3 (CH), 126.2 (CH), 121.0 (2xCH), 81.9 (C), 80.5 (C), 79.8 (4xC), 72.3 (C), 68.2 (CH_2), 56.4 (CH), 55.5 (CH), 55.3 (CH), 55.0 (CH), 54.7 (CH), 48.4 (CH), 41.2 (2xCH_2), 41.2 (2xCH_2), 32.7 (3xCH_2), 32.4 (CH_2), 32.3 (CH_2), 30.6 (2xCH_2), 30.5 (2xCH_2), 29.5 (CH_2), 28.9 (9xCH_3), 28.8 (3xCH_3), 28.4 (3xCH_3), 28.1 (CH_2), 24.4 (CH_2), 24.2 (3xCH_2).

Compound 113 was prepared following the general procedure of Fmoc deprotection, starting from **112** (488 mg, 0.355 mmol) in 6 mL of 2 M dimethylamine in THF, yielding **113** as a colorless solid (406 mg, 99%).

Compound 114. To a solution of **113** (400 mg, 0.347 mmol) in dry DMF (3 mL), was added a 30 seconds-premixed solution of HATU (158 mg, 0.416 mmol), Fmoc-Glu(O*t*Bu)-OH (177 mg, 0.416 mmol) and DIPEA (73 µL, 0.42 mmol) in dry DMF (3 mL). Reaction mixture was stirred at rt for 30 min. Reaction mixture was diluted with EtOAc and washed with $NaHCO_3$ sat. aq. solution (x3), 10% citric acid solution (x3), water (x1), 5% LiCl solution (x2)

and brine. Organic phase was then dried with Na_2SO_4, filtered and concentrated. Crude was dissolved in a minimal amount of CH_2Cl_2 and precipitated in pentane (x3). Crude was then purified by flash column chromatography (BGB® Scorpius 25 g SiO_2 col., 40 mL/min, linear gradient 0 – 8% MeOH in CH_2Cl_2) to yield **114** as a colorless solid (487 mg, 90%). R_f (CH_2Cl_2 + 10% MeOH): 0.5; Mp: 225.4 – 225.9 °C; $[\alpha]_D^{20}$ – 21.9 (*c* 0.50, MeOH); IR (neat): 3276 (m), 2932 (m), 1687 (s), 1627 (s), 1520 (s), 1451 (m), 1365 (m), 1248 (s), 1165 (s), 619 (m); ^1H NMR (500 MHz, CD_3OD): 7.81 (d, $^3J_{H-H}$ = 7.4 Hz, 2H), 7.69 (d, $^3J_{H-H}$ = 7.4 Hz, 1H), 7.67 (d, $^3J_{H-H}$ = 7.4 Hz, 1H), 7.40 (t, $^3J_{H-H}$ = 7.4 Hz, 2H), 7.32 (t, $^3J_{H-H}$ = 7.4 Hz, 2H), 4.52 – 4.43 (m, 1H), 4.36 (dd, $^3J_{H-H}$ = 10.3, 7.0 Hz, 1H), 4.30 – 4.12 (m, 6H), 4.08 – 4.03 (m, 1H), 4.01 – 3.91 (m, 2H), 3.07 – 2.94 (m, 8H), 2.56 (t, $^4J_{H-H}$ = 2.5 Hz, 1H), 2.45 – 2.31 (m, 4H), 2.12 – 2.00 (m, 2H), 1.99 – 1.91 (m, 2H), 1.89 – 1.62 (m, 8H), 1.56 – 1.32 (m, 16H), 1.45 (s, 9H), 1.42 (s, 9H), 1.42 (s, 36H); ^{13}C NMR (126 MHz, CD_3OD): 175.4 (C), 175.1 (C), 175.0 (C), 174.7 (C), 174.4 (C), 174.2 (C), 173.9 (C), 173.8 (C), 159.0 (C), 158.4 (4xC), 145.2 (C), 145.1 (C), 142.6 (C), 142.6 (C), 128.9 (CH), 128.8 (CH), 128.3 (CH), 128.2 (CH), 126.3 (CH), 126.2 (CH), 121.0 (CH), 120.9 (CH), 82.0 (C), 81.9 (C), 80.5 (C), 79.8 (C), 79.8 (2xC), 79.8 (C), 72.3 (CH), 68.3 (CH_2), 57.0 (CH), 56.1 (CH), 55.9 (CH), 55.7 (CH), 55.5 (CH), 54.9 (CH), 48.3 (CH), 41.2 (3xCH_2), 41.1 (CH_2), 32.7 (CH_2), 32.6 (2xCH_2), 32.3 (CH_2), 32.0 (CH_2), 32.0 (CH_2), 30.6 (3xCH_2), 30.5 (CH_2), 29.6 (CH_2), 28.9 (12xCH_3), 28.5 (6xCH_3), 27.8 (CH_2), 27.3 (CH_2), 24.5 (CH_2), 24.4 (2xCH_2), 24.3 (CH_2).

Compound 115 was prepared following the general procedure of Fmoc deprotection, starting from **114** (435 mg, 0.279 mmol) in 6 mL of 2 M dimethylamine in THF, yielding **115** as a colorless solid (370 mg, 99%).

Compound 116. To a solution of **115** (230 mg, 0.172 mmol) in dry DMF (2 mL), was added a 30 seconds-premixed solution of HATU (78 mg, 0.21 mmol), **35** (67 mg, 0.21 mmol) and DIPEA (36 µL, 0.21 mmol) in dry DMF (2 mL). Reaction mixture was stirred at rt for 30

min. Reaction mixture was diluted with EtOAc and washed with $NaHCO_3$ sat. aq. solution (x3), 10% citric acid solution (x3), water (x1), 5% LiCl solution (x2) and brine. Organic phase was then dried with Na_2SO_4, filtered and concentrated. Crude was dissolved in a minimal amount of CH_2Cl_2 and precipitated in pentane (x3). Crude was then purified by flash column chromatography (BGB® Scorpius 25 g SiO_2 col., 40 mL/min, linear gradient 0 – 8% MeOH in CH_2Cl_2) to yield **116** as a colorless solid (238 mg, 84%). R_f (CH_2Cl_2 + 10% MeOH): 0.4; Mp: 251.2 – 253.0 °C; $[\alpha]_D^{20}$ + 1.7 (*c* 0.50, MeOH); IR (neat): 3279 (m), 2936 (m), 1690 (br s), 1623 (s), 1521 (s), 1449 (m), 1365 (m), 1247 (m), 1164 (s), 645 (m); ^{1}H NMR (500 MHz, CD_3OD): 4.27 (dd, $^3J_{H-H}$ = 10.0, 4.4 Hz, 1H), 4.16 (t, $^3J_{H-H}$ = 7.0 Hz, 1H), 4.14 – 4.02 (m, 4H), 3.99 (dd, $^2J_{H-H}$ = 17.4, $^4J_{H-H}$ = 2.5 Hz, 1H), 3.94 (dd, $^2J_{H-H}$ = 17.4, $^4J_{H-H}$ = 2.5 Hz, 1H), 3.62 – 3.57 (m, 6H), 3.56 (t, $^3J_{H-H}$ = 6.7 Hz, 2H), 3.54 (t, $^3J_{H-H}$ = 5.8 Hz, 2H), 3.49 (t, $^3J_{H-H}$ = 6.6 Hz, 2H), 3.43 (dt, $^2J_{H-H}$ = 12.6, $^3J_{H-H}$ = 4.7 Hz, 1H), 3.35 (dt, $^2J_{H-H}$ = 12.6, $^3J_{H-H}$ = 4.7 Hz, 1H), 3.10 – 2.96 (m, 8H), 2.83 – 2.73 (m, 1H), 2.60 – 2.51 (m, 2H), 2.53 (t, $^4J_{H-H}$ = 2.5 Hz, 1H), 2.50 – 2.36 (m, 5H), 2.26 – 2.12 (m, 2H), 2.11 – 1.98 (m, 2H), 1.92 – 1.81 (m, 6H), 1.81 – 1.73 (m, 4H), 1.65 – 1.57 (m, 2H), 1.56 – 1.37 (m, 20H), 1.45 (s, 9H), 1.44 (s, 9H), 1.43 (s, 9H), 1.42 (s, 27H); ^{13}C NMR (126 MHz, CD_3OD): 176.8 (C), 176.3 (C), 176.1 (C), 176.0 (C), 175.9 (C), 175.0 (C), 174.8 (C), 174.2 (C), 173.6 (C), 173.6 (C), 158.4 (C), 158.4 (C), 158.4 (C), 158.3 (C), 81.9 (C), 81.8 (C), 80.6 (C), 79.9 (C), 79.8 (C), 79.7 (C), 79.7 (C), 72.3 (CH_2), 72.1 (CH), 71.3 (CH_2), 71.2 (CH_2), 70.7 (CH_2), 57.2 (CH), 57.1 (CH), 57.0 (CH), 56.7 (CH), 56.4 (CH), 55.3 (CH), 45.7 (CH_2), 41.3 (CH_2), 41.3 (CH_2), 41.3 (CH_2), 41.2 (CH_2), 40.7 (CH_2), 33.8 (CH_2), 32.8 (CH_2), 32.5 (CH_2), 32.4 (CH_2), 32.2 (CH_2), 32.1 (CH_2), 31.8 (CH_2), 31.8 (CH_2), 31.7 (CH_2), 30.8 (CH_2), 30.7 (CH_2), 30.6 (CH_2), 30.5 (CH_2), 30.5 (CH_2), 29.6 (CH_2), 28.9 (6xCH_3), 28.9 (6xCH_3), 28.5 (3xCH_3), 28.5 (3xCH_3), 27.8 (CH_2), 27.3 (CH_2), 26.7 (CH_2), 26.5 (CH_2), 24.7 (CH_2), 24.7 (CH_2), 24.7 (CH_2), 24.5 (CH_2).

Compound 117. To a solution of **116** (195 mg, 0.119 mmol) in THF/H_2O 2/1 (9 mL), **36** (45 mg, 0.14 mmol), $CuSO_4 \cdot 5H_2O$ (30 mg, 0.12 mmol), sodium ascorbate (47 mg, 0.24 mmol) and TBTA (6.3 mg, 12 μmol) were added and the reaction mixture was stirred at rt for 1 h. Solvent was removed *in vacuo*, H_2O and CH_2Cl_2 were added, phases were separated, and aqueous phase was extracted by CH_2Cl_2 (x3). Organic phases were collected together, washed with brine (x1), dried over Na_2SO_4, filtered and concentrated. Crude was purified by flash column chromatography (BGB® Scorpius 12 g, 25 mL/min, linear gradient 0 – 10% MeOH in CH_2Cl_2) to yield **117** as a colorless solid (160 mg, 69%). R_f (CH_2Cl_2 + 10% MeOH): 0.2; Mp: 201.2 – 201.9 °C; $[\alpha]_D^{20}$ + 14.9 (*c* 0.50, MeOH); IR (neat): 3280 (m), 2934 (m), 1691 (s), 1624 (s), 1520 (s), 1453 (m), 1365 (m), 1248 (s), 1164 (s), 702 (m), 647 (m); ^{1}H NMR (500 MHz, CD_3OD): 7.91 (s, 1H), 4.63 (d, $^2J_{H\text{-}H}$ = 15.3 Hz, 1H), 4.53 – 4.48 (m, 3H), 4.38 (d, $^2J_{H\text{-}H}$ = 15.3 Hz, 1H), 4.34 (dd, $^3J_{H\text{-}H}$ = 7.9, 4.4 Hz, 1H), 4.22 (dd, $^3J_{H\text{-}H}$ = 9.7, 4.4 Hz, 1H), 4.12 – 4.03 (m, 3H), 4.01 – 3.91 (m, 2H), 3.73 – 3.61 (m, 2H), 3.61 – 3.58 (m, 4H), 3.56 (t, $^3J_{H\text{-}H}$ = 6.6 Hz, 2H), 3.54 (t, $^3J_{H\text{-}H}$ = 5.5 Hz, 2H), 3.49 (t, $^3J_{H\text{-}H}$ = 6.6 Hz, 2H), 3.46 – 3.42 (m, 1H), 3.38 – 3.33 (m, 1H), 3.25 – 3.20 (m, 1H), 3.09 – 2.98 (m, 8H), 2.95 (dd, $^2J_{H\text{-}H}$ = 12.7, $^3J_{H\text{-}H}$ = 5.0 Hz, 1H), 2.84 – 2.74 (m, 1H), 2.71 (d, $^2J_{H\text{-}H}$ = 12.7 Hz, 1H), 2.60 – 2.51 (m, 2H), 2.51 – 2.36 (m, 5H), 2.18 (t, $^3J_{H\text{-}H}$ = 7.3 Hz, 4H), 2.08 – 2.00 (m, 2H), 1.94 – 1.69 (m, 10H), 1.66 – 1.54 (m, 4H), 1.53 – 1.36 (m, 24H), 1.45 (s, 18H), 1.43 (s, 9H), 1.42 (s, 27H); ^{13}C NMR (126 MHz, CD_3OD): 176.8 (C), 176.6 (C), 176.5 (2xC), 176.3 (C), 176.2 (2xC), 175.5 (C), 174.9 (C), 173.5 (C), 173.5 (C), 166.1 (C), 158.4 (C), 158.4 (C), 158.3 (C), 158.3 (C), 147.0 (C), 125.0 (CH), 81.9 (C), 81.8 (C), 79.9 (C), 79.8 (C), 79.7 (C), 79.7 (C), 72.3 (CH_2), 71.3 (CH_2), 71.2 (CH_2), 70.7 (CH_2), 63.3 (CH), 61.6 (CH), 57.6 (2xCH), 57.3 (CH), 57.0 (2xCH), 56.9 (CH), 56.1 (CH), 50.6 (CH_2), 45.7 (CH_2), 41.3 (2xCH_2), 41.3 (CH_2), 41.2 (CH_2), 41.1 (CH_2), 40.7 (CH_2), 40.5 (CH_2), 36.7 (CH_2), 36.1 (CH_2), 33.8 (CH_2), 32.8 (CH_2), 32.4 (CH_2), 32.3 (CH_2), 32.2 (CH_2), 32.1 (CH_2), 31.8 (CH_2), 31.7 (CH_2), 31.6 (CH_2), 30.8 (CH_2), 30.7 (CH_2), 30.6 (2xCH_2), 30.6

(CH_2), 29.7 (CH_2), 29.4 (CH_2), 28.9 (6xCH_3), 28.9 (6xCH_3), 28.5 (3xCH_3), 28.5 (3xCH_3), 27.8 (CH_2), 27.3 (CH_2), 26.8 (CH_2), 26.6 (CH_2), 26.5 (CH_2), 24.9 (CH_2), 24.8 (CH_2), 24.8 (CH_2), 24.7 (CH_2).

Compound 118. A solution of **117** (127 mg, 64.9 μmol) in TFA/H_2O/TIPS 95/2.5/2.5 (2 mL) was stirred at rt for 30 min. Solvent was removed *in vacuo*, crude was dissolved in small amount of MeOH and was precipitated in Et_2O (x3) to yield **118** as a colorless TFA salt (107 mg, 87%). Crude was used in the next reaction without further purification.

Compound 11. To a solution of **118** (25 mg, 13 μmol) in DMF (600 μL), **37** (16 mg, 66 μmol) and DIPEA (18 μL, 105 μmol) were added and reaction mixture was stirred at rt for 1 h. Crude reaction mixture was purified by RP flash chromatography (Biotage® SNAP Ultra 12 g C18, 12 mL/min, linear gradient 20 – 50% CH_3CN in H_2O with 0.1% TFA) to yield **11** as a colorless solid (10 mg, 39%). ^{1}H NMR (400 MHz, DMSO-d_6/D_2O/TFA 300/5/2): 7.83 (s, 1H), 4.37 (t, $^3J_{H-H}$ = 6.2 Hz, 2H), 4.35 – 4.29 (m, 2H), 4.25 (d, $^2J_{H-H}$ = 15.3 Hz, 1H), 4.19 – 4.09 (m, 7H), 3.61 (t, $^3J_{H-H}$ = 6.6 Hz, 2H), 3.50 – 3.42 (m, 6H), 3.40 – 3.31 (m, 12H), 3.20 – 3.07 (m, 15H), 3.05 – 2.99 (m, 8H), 2.85 – 2.78 (d, $^2J_{H-H}$ = 12.6 Hz, $^3J_{H-H}$ = 5.1 Hz, 1H), 2.58 (d, $^2J_{H-H}$ = 12.6 Hz, 1H), 2.43 – 2.21 (m, 8H), 2.03 (t, $^3J_{H-H}$ = 7.4 Hz, 2H), 1.99 – 1.88 (m, 2H), 1.87 – 1.74 (m, 2H), 1.74 – 1.58 (m, 7H), 1.58 – 1.42 (m, 10H), 1.42 – 1.32 (m,11H), 1.32 – 1.18 (m, 10H); ^{13}C NMR (126 MHz, DMSO-d_6): 173.9 (3xC), 172.6 (C), 172.6 (C), 172.0 (C), 171.8 (C), 171.7 (C), 171.6 (C), 171.5 (C), 171.4 (C), 171.4 (C), 170.2 (C), 170.2 (C), 170.2 (C), 162.7 (C), 144.5 (C), 122.9 (CH), 70.2 (CH_2), 69.6 (CH_2), 69.4 (CH_2), 69.1 (CH_2), 61.0 (CH), 59.2 (CH), 55.4 (CH), 52.8 (CH), 52.7 (CH), 52.7 (CH), 52.6 (CH), 52.5 (2xCH), 51.5 (4xCH), 48.7 (CH_2), 45.4 (CH_2), 42.1 (4xCH_2), 42.1 (2xCH_2), 42.1 (2xCH_2), 39.6 (CH_2), 38.9 (CH_2), 38.8 (3xCH_2), 38.7 (CH_2), 38.6 (CH_2), 35.1 (CH_2), 34.3 (CH_2), 32.0 (CH_2), 31.7 (CH_2), 31.4 (CH_2), 31.3 (CH_2), 31.2 (CH_2), 30.7 (CH_2), 30.5 (CH_2), 30.2 (CH_2), 30.1 (CH_2), 29.1 (CH_2), 28.6 (4xCH_2), 28.1 (CH_2), 28.0 (CH_2), 26.8 (CH_2), 26.6 (CH_2), 26.1 (CH_2), 25.1 (CH_2),

24.9 (CH_2), 22.8 (CH_2), 22.8 (CH_2), 22.7 2x(CH_2); HRMS (ESI, +ve) calcd for $C_{79}H_{127}ClN_{18}O_{20}S_9$ ($[M + 2H]^{2+}$): 986.3398, found: 986.3509; LC-MS (ESI, B30): R_t 1.91 min, 1917 (50, $[M+H]^+$), 987 (100, $[M+2H]^{2+}$), 987 (15, $[M+3H]^{3+}$).

2.1.8. Synthesis of peptides 12 and 14

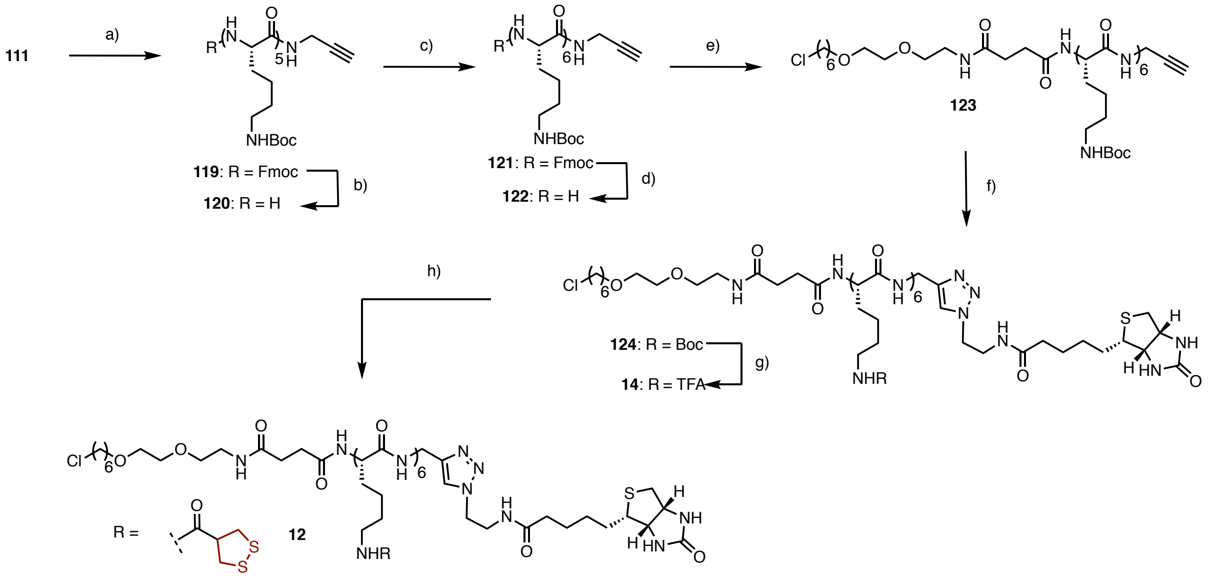

Scheme S8 (a) Fmoc-Lys(Boc)-OH, HATU, DIPEA, DMF, rt, 30 min, 92%; (b) 2 M dimethylamine in THF, rt, 30 min, 96%; (c) Fmoc-Lys(Boc)-OH, HATU, DIPEA, DMF, rt, 30 min, 90%; (d) 2 M dimethylamine in THF, rt, 30 min, 100%; (e) **35**, HATU, DIPEA, DMF, rt, 30 min, 87%; (f) **36**, $CuSO_4{\cdot}5H_2O$, Na-ascorbate, TBTA, THF/H_2O, rt, 1 h, 87%; (g) TFA/TIPS/H_2O 95/2.5/2.5, rt, 30 min, 91%; (h) **37**, DIPEA, DMF, rt, 2 h, 77%.

Compound 119. To a solution of **111** (670 mg, 0.692 mmol) in dry DMF (3 mL), was added a 30 seconds-premixed solution of HATU (316 mg, 0.830 mmol), Fmoc-Lys(Boc)-OH (389 mg, 0.830 mmol) and DIPEA (145 µL, 0.830 mmol) in dry DMF (3 mL). Reaction mixture was stirred at rt for 30 min. Reaction mixture was diluted with EtOAc and washed with $NaHCO_3$ sat. aq. solution (x3), 10% citric acid solution (x3), water (x1), 5% LiCl solution (x2) and brine. Organic phase was then dried with Na_2SO_4, filtered and concentrated. Crude was

dissolved in a minimal amount of CH_2Cl_2 and precipitated in pentane (x3). Crude was then purified by flash column chromatography (BGB® Scorpius 25 g SiO_2 col., 40 mL/min, linear gradient 0 – 8% MeOH in CH_2Cl_2) to yield **119** as a colorless solid (905 mg, 92%). R_f (CH_2Cl_2 + 10% MeOH): 0.5; Mp: 208.5 – 209.3 °C; $[\alpha]_D^{20}$ – 21.3 (*c* 0.50, MeOH); IR (neat): 3308 (m), 2939 (m), 1686 (s), 1630 (s), 1524 (s), 1446 (m), 1365 (m), 1275 (m), 1250 (s), 1169 (s), 735 (m); ^{1}H NMR (500 MHz, CD_3OD): 7.81 (d, $^3J_{H-H}$ = 7.5 Hz, 2H), 7.68 (d, $^3J_{H-H}$ = 7.5 Hz, 2H), 7.40 (t, $^3J_{H-H}$ = 7.5 Hz, 2H), 7.32 (t, $^3J_{H-H}$ = 7.45 Hz, 2H), 4.49 – 4.40 (m, 1H), 4.36 (t, $^3J_{H-H}$ = 8.6 Hz, 2H), 4.32 – 4.20 (m, 4H), 4.08 – 4.02 (m, 1H), 3.99 (dd, $^2J_{H-H}$ = 17.5, $^4J_{H-H}$ = 2.5 Hz, 1H), 3.93 (dd, $^2J_{H-H}$ = 17.5, $^4J_{H-H}$ = 2.5 Hz, 1H), 3.09 – 2.96 (m, 10H), 2.58 (t, $^4J_{H-H}$ = 2.5 Hz, 1H), 1.86 – 1.75 (m, 5H), 1.74 – 1.63 (m, 5H), 1.51 – 1.34 (m, 20H), 1.42 (s, 36H), 1.40 (s, 9H); ^{13}C NMR (126 MHz, CD_3OD): 175.6 (C), 174.6 (C), 174.5 (C), 174.2 (C), 173.8 (C), 158.8 (C), 158.6 (C), 158.5 (4xC), 145.3 (C), 145.2 (C), 142.6 (2xC), 128.8 (2xCH), 128.2 (2xCH), 126.3 (CH), 126.2 (CH), 121.0 (2xCH), 80.5 (C), 79.8 (5xC), 72.4 (CH), 68.1 (CH_2), 57.1 (CH), 55.4 (CH), 55.3 (CH), 55.0 (CH), 54.6 (CH), 48.4 (CH), 41.2 (4xCH_2), 41.1 (CH_2), 32.7 (CH_2), 32.5 (CH_2), 32.4 (CH_2), 32.4 (CH_2), 32.2 (CH_2), 30.6 (CH_2), 30.5 (4xCH_2), 29.5 (CH_2), 28.9 (15xCH_3), 24.2 (2xCH_2), 24.2 (3xCH_2).

Compound 120 was prepared following the general procedure of Fmoc deprotection, starting from **119** (850 mg, 0.599 mmol) in 8 mL of 2 M dimethylamine in THF, yielding **120** as a colorless solid (690 mg, 96%).

Compound 121. To a solution of **120** (620 mg, 0.518 mmol) in dry DMF (3 mL), was added a 30 seconds-premixed solution of HATU (236 mg, 0.622 mmol), Fmoc-Lys(Boc)-OH (291 mg, 0.622 mmol) and DIPEA (108 µL, 0.622 mmol) in dry DMF (3 mL). Reaction mixture was stirred at rt for 30 min. Reaction mixture was diluted with EtOAc and washed with $NaHCO_3$ sat. aq. solution (x3), 10% citric acid solution (x3), water (x1), 5% LiCl solution (x2) and brine. Organic phase was then dried with Na_2SO_4, filtered and concentrated. Crude was

dissolved in a minimal amount of CH_2Cl_2 and precipitated in pentane (x3). Crude was then purified by flash column chromatography (BGB® Scorpius 25 g SiO_2 col., 40 mL/min, linear gradient 0 – 8% MeOH in CH_2Cl_2) to yield **121** as a colorless solid (764 mg, 90%). R_f (CH_2Cl_2 + 10% MeOH): 0.5; Mp: 227.9 – 228.7 °C; $[\alpha]_D^{20}$ – 22.7 (*c* 0.50, MeOH); IR (neat): 3283 (w), 2931 (w), 1688 (s), 1628 (s), 1526 (s), 1448 (m), 1365 (m), 1365 (m), 1250 (s), 1171 (s), 740 (m), 633 (m); ^{1}H NMR (500 MHz, CD_3OD): 7.81 (d, $^3J_{H\text{-}H}$ = 7.5 Hz, 2H), 7.68 (d, $^3J_{H\text{-}H}$ = 7.5 Hz, 2H), 7.40 (t, $^3J_{H\text{-}H}$ = 7.5 Hz, 2H), 7.32 (t, $^3J_{H\text{-}H}$ = 7.5 Hz, 2H), 4.51 – 4.41 (m, 1H), 4.36 (dd, $^3J_{H\text{-}H}$ = 10.5, 6.9 Hz, 1H), 4.32 – 4.14 (m, 6H), 4.07 – 3.99 (m, 1H), 3.99 (dd, $^2J_{H\text{-}H}$ = 17.6, $^4J_{H\text{-}H}$ = 2.5 Hz, 1H), 3.94 (dd, $^2J_{H\text{-}H}$ = 17.6, $^4J_{H\text{-}H}$ = 2.5 Hz, 1H), 3.08 – 2.96 (m, 12H), 2.57 (t, $^4J_{H\text{-}H}$ = 2.5 Hz, 1H), 1.86 – 1.75 (m, 6H), 1.75 – 1.65 (m, 6H), 1.51 – 1.34 (m, 24H), 1.42 (s, 45H), 1.40 (s, 9H); ^{13}C NMR (126 MHz, CD_3OD): 175.8 (C), 174.9 (C), 174.8 (2xC), 174.3 (C), 173.8 (C), 158.9 (C), 158.6 (C), 158.4 (5xC), 145.2 (C), 145.2 (C), 142.6 (2xC), 128.9 (2xCH), 128.2 (2xCH), 126.3 (CH), 126.2 (CH), 121.0 (2xCH), 80.5 (C), 79.9 (C), 79.8 (5xC), 72.3 (CH), 68.2 (CH_2), 55.9 (CH), 55.6 (CH), 55.3 (CH), 54.7 (3xCH), 48.4 (CH), 41.2 (3xCH_2), 41.2 (2xCH_2), 41.1 (CH_2), 32.6 (2xCH_2), 32.4 (2xCH_2), 32.2 (2xCH_2), 30.7 (CH_2), 30.6 (CH_2), 30.6 (CH_2), 30.5 (3xCH_2), 29.5 (CH_2), 28.9 (18xCH_3), 24.3 (2xCH_2), 24.2 (4xCH_2).

Compound 122 was prepared following the general procedure of Fmoc deprotection, starting from **121** (700 mg, 0.425 mmol) in 8 mL of 2 M dimethylamine in THF, yielding **122** as a colorless solid (605 mg, 100%).

Compound 123. To a solution of **122** (540 mg, 0.379 mmol) in dry DMF (3 mL), was added a 30 seconds-premixed solution of HATU (173 mg, 0.455 mmol), **35** (147 mg, 0.455 mmol) and DIPEA (79 µL, 0.46 mmol) in dry DMF (3 mL). Reaction mixture was stirred at rt for 30 min. Reaction mixture was diluted with EtOAc and washed with $NaHCO_3$ sat. aq. solution (x3), 10% citric acid solution (x3), water (x1), 5% LiCl solution (x2) and brine.

Organic phase was then dried with Na_2SO_4, filtered and concentrated. Crude was dissolved in a minimal amount of CH_2Cl_2 and precipitated in pentane (x3). Crude was then purified by flash column chromatography (BGB® Scorpius 25 g SiO_2 col., 40 mL/min, linear gradient 0 – 8% MeOH in CH_2Cl_2) to yield **123** as a colorless solid (572 mg, 87%). R_f (CH_2Cl_2 + 10% MeOH): 0.5; Mp: 241.2 – 242.3 °C; $[\alpha]_D^{20}$ – 0.5 (*c* 0.50, MeOH); IR (neat): 3296 (m), 2935 (m), 1688 (s), 1626 (s), 1518 (s), 1452 (m), 1365 (m), 1248 (s), 1168 (s), 706 (m), 645 (m); ^1H NMR (400 MHz, DMSO-d_6/D_2O 60/1): 4.18 – 3.93 (m, 6H), 3.85 – 3.78 (m, 2H), 3.57 (t, $^3J_{H-H}$ = 6.6 Hz, 2H), 3.48 – 3.40 (m, 4H), 3.39 – 3.30 (m, 4H), 3.17 (t, $^3J_{H-H}$ = 5.8 Hz, 2H), 2.96 (t, $^4J_{H-H}$ = 2.5 Hz, 1H), 2.90 – 2.77 (m, 12H), 2.47 – 2.20 (m, 4H), 1.73 – 1.40 (m, 18H), 1.37 – 1.15 (m, 28H), 1.33 (s, 54H); ^{13}C NMR (126 MHz, DMSO-d_6): 173.2 (C), 172.8 (C), 172.4 (C), 172.2 (C), 172.0 (C), 171.8 (C), 171.5 (C), 171.5 (C), 155.7 (C), 155.7 (3xC), 155.6 (4xC), 155.3 (C), 81.0 (C), 77.6 (5xC), 77.5 (C), 73.0 (CH), 70.3 (CH_2), 69.7 (CH_2), 69.5 (CH_2), 69.1 (CH_2), 54.0 (CH), 53.7 (CH), 53.2 (CH), 53.1 (CH), 52.9 (CH), 52.5 (CH), 45.5 (CH_2), 41.0 (CH_2), 38.8 (6xCH_2), 32.1 (CH_2), 31.7 (CH_2), 31.5 (CH_2), 31.4 (CH_2), 31.2 (CH_2), 31.1 (CH_2), 30.8 (2xCH_2), 30.6 (CH_2), 29.4 (5xCH_2), 29.3 (CH_2), 29.2 (CH_2), 28.4 (15xCH_3), 28.3 (3xCH_3), 28.1 (CH_2), 26.2 (CH_2), 25.0 (CH_2), 23.2 (CH_2), 22.9 (CH_2), 22.9 (CH_2), 22.9 (CH_2), 22.8 (CH_2), 22.7 (CH_2).

Compound 124. To a solution of **123** (200 mg, 0.116 mmol) in THF/H_2O 2/1 (3 mL), **36** (43 mg, 0.14 mmol), $CuSO_4 \cdot 5H_2O$ (29 mg, 0.14 mmol), sodium ascorbate (46 mg, 0.23 mmol) and TBTA 63 mg, 12 µmol) were added and the reaction mixture was stirred at rt for 1 h. Solvent was removed *in vacuo*, H_2O and CH_2Cl_2 were added, phases were separated, and aqueous phase was extracted by CH_2Cl_2 (x3). Organic phases were collected together, washed with brine (x1), dried over Na_2SO_4, filtered and concentrated. Crude was purified by flash column chromatography (BGB® Scorpius 25g, 25 mL/min, linear gradient 0 – 10% MeOH in CH_2Cl_2) to yield **124** as a colorless solid (205 mg, 87%). R_f (CH_2Cl_2 + 10% MeOH): 0.5; Mp:

218.6 – 219.5 °C; $[\alpha]_D^{20}$ + 14.0 (*c* 0.50, MeOH); IR (neat): 3271 (m), 2935 (m), 1690 (s), 1625 (s), 1524 (s), 1454 (m), 1248 (s), 1169 (s), 1055 (s), 1014 (s), 704 (m), 628 (m); ^{1}H NMR (400 MHz, DMSO-d_6/D_2O 60/1): 7.78 (s, 1H), 4.38 – 4.28 (m, 4H), 4.24 (d, $^2J_{H-H}$ = 15.4 Hz, 1H), 4.14 (dd, $^3J_{H-H}$ = 7.8, 4.4 Hz, 1H), 4.11 – 4.03 (m, 2H), 4.02 – 3.91 (m, 4H), 3.57 (t, $^3J_{H-H}$ = 6.5 Hz, 2H), 3.50 – 3.40 (m, 6H), 3.39 – 3.31 (m, 4H), 3.17 (t, $^3J_{H-H}$ = 5.7 Hz, 2H), 3.11 – 3.04 (m, 1H), 2.91 – 2.77 (m, 13H), 2.57 (d, $^2J_{H-H}$ = 12.9 Hz, 1H), 2.45 – 2.21 (m, 4H), 2.01 (t, $^3J_{H-H}$ = 7.4 Hz, 2H), 1.75 – 1.41 (m, 20H), 1.37 – 1.13 (m, 30H), 1.32 (s, 54H); ^{13}C NMR (126 MHz, DMSO-d_6): 173.7 (C), 173.3 (C), 173.1 (C), 173.0 (C), 172.9 (C), 172.5 (C), 172.2 (C), 171.9 (C), 171.8 (C), 163.1 (C), 155.9 (3xC), 155.9 (3xC), 144.9 (C), 123.2 (CH), 77.8 (C), 77.8 (2xC), 77.7 (3xC), 70.4 (CH_2), 69.8 (CH_2), 69.6 (CH_2), 69.2 (CH_2), 61.3 (CH), 59.4 (CH), 55.6 (CH), 54.4 (CH), 54.2 (CH), 53.6 (CH), 53.6, (CH) 53.3 (CH), 53.0 (CH), 48.9 (CH_2), 45.6 (CH_2), 40.0 (5xCH_2), 40.0 (CH_2), 39.0 (CH_2), 39.0 (CH_2), 35.3 (CH_2), 34.5 (CH_2), 32.2 (CH_2), 31.7 (CH_2), 31.4 (CH_2), 31.3 (CH_2), 31.1 (CH_2), 31.1 (CH_2), 30.9 (CH_2), 30.6 (2xCH_2), 29.4 (5xCH_2), 29.4 (CH_2), 29.2 (CH_2), 28.5, 28.3 (CH_2), 28.2 (CH_2), 26.3 (CH_2), 25.4 (CH_2), 25.1 (CH_2), 23.3 (CH_2), 23.0 (2xCH_2), 23.0 (CH_2), 22.9 (2xCH_2).

Compound 14. A solution of **124** (176 mg, 86.1 µmol) in TFA/H_2O/TIPS 95/2.5/2.5 (2 mL) was stirred at rt for 30 min. Solvent was removed *in vacuo*, crude was dissolved in small amount of MeOH and was precipitated in Et_2O (x3) to yield **14** as a colorless TFA salt (166 mg, 91%).^{1}H NMR (500 MHz, DMSO-d_6): 8.41 (br s, NH), 8.23 (br s, NH), 8.03 (t, $^3J_{H-H}$ = 5.8 Hz, NH), 7.87 (s, 1H), 7.81 (s, 18xNH), 6.45 (s, NH), 6.41 (s, NH), 4.38 (t, $^3J_{H-H}$ = 6.3 Hz, 2H), 4.35 – 4.24 (m, 3H), 4.24 – 4.08 (m, 7H), 3.62 (t, $^3J_{H-H}$ = 6.6 Hz, 2H), 3.51 – 3.43 (m, 6H), 3.42 – 3.34 (m, 4H), 3.22 – 3.13 (m, 2H), 3.10 (ddd, $^3J_{H-H}$ = 8.5, 6.3, 4.4 Hz, 1H), 2.82 (dd, $^2J_{H-H}$ = 12.4, $^3J_{H-H}$ = 5.1 Hz, 1H), 2.80 – 2.70 (m, 13H), 2.58 (d, $^2J_{H-H}$ = 12.4 Hz, 1H), 2.40 – 2.29 (m, 4H), 2.05 (t, $^3J_{H-H}$ = 7.4 Hz, 2H), 1.74 – 1.43 (m, 32H), 1.41 – 1.21 (m, 18H); ^{13}C NMR (126 MHz, DMSO-d_6): 172.7 (3xC), 172.2 (C), 171.9 (C), 171.7 (2xC), 171.5 (C), 171.4

(C), 162.8 (C), 144.3 (C), 123.0 (CH), 70.2 (CH_2), 69.6 (CH_2), 69.4 (CH_2), 69.0 (CH_2), 61.0 (CH), 59.2 (CH), 55.4 (CH), 53.1 (CH), 52.7 (2xCH), 52.3 (3xCH), 48.6 (CH_2), 45.4 (CH_2), 39.8 (CH_2), 39.6 (CH_2), 38.8 (CH_2), 38.6 (5xCH_2), 38.6 (CH_2), 35.1 (CH_2), 34.2 (CH_2), 32.0 (CH_2), 31.4 (CH_2), 31.3 (CH_2), 31.2 (CH_2), 31.1 (CH_2), 30.8 (CH_2), 30.6 (2xCH_2), 30.4 (CH_2), 29.0 (CH_2), 28.1 (CH_2), 28.0 (CH_2), 26.6 (3xCH_2), 26.6 (3xCH_2), 26.1 (CH_2), 25.2 (CH_2), 24.9 (CH_2), 22.4 (2xCH_2), 22.2 (4xCH_2); HRMS (ESI, +ve) calcd for $C_{65}H_{121}ClN_{20}O_{12}S$ ([M + 2H]$^{2+}$): 721.4514, found: 721.4502.

Compound 12. To a solution of **14** (15 mg, 7.1 μmol) in CH_2Cl_2/MeOH (400 μL), **37** (14 mg, 56 μmol) and DIPEA (8.6 μL, 49 μmol) were added and reaction mixture was stirred at rt for 2 h. Crude reaction mixture was precipitated in pentane/CH_2Cl_2 (x5) to yield **12** as a colorless solid (12 mg, 77%). ^{1}H NMR (500 MHz, DMSO-d_6): 8.28 (t, $^3J_{H-H}$ = 5.6 Hz, NH), 8.23 (d, $^3J_{H-H}$ = 6.4 Hz, NH), 8.12 – 8.01 (m, 6xNH), 8.00 – 7.93 (m, 2xNH), 7.84 (s, 1H), 7.76 (s, NH), 7.69 (d, $^3J_{H-H}$ = 7.3 Hz, NH), 7.67 (d, $^3J_{H-H}$ = 7.3 Hz, NH), 6.42 (s, NH), 6.36 (s, NH), 4.37 (t, $^3J_{H-H}$ = 6.2 Hz, 2H), 4.34 – 4.23 (m, 3H), 4.20 – 4.02 (m, 7H), 3.62 (t, $^3J_{H-H}$ = 6.6 Hz, 2H), 3.51 – 3.42 (m, 6H), 3.42 – 3.35 (m, 16H), 3.21 – 3.09 (m, 21H), 3.09 – 2.96 (m, 12H), 2.82 (dd, $^2J_{H-H}$ = 12.4, $^3J_{H-H}$ = 5.1 Hz, 1H), 2.58 (d, $^2J_{H-H}$ = 12.4 Hz, 3H), 2.46 – 2.28 (m, 2H), 2.04 (t, $^3J_{H-H}$ = 7.3 Hz, 2H), 1.76 – 1.58 (m, 10H), 1.59 – 1.43 (m, 10H), 1.43 – 1.33 (m, 16H), 1.33 – 1.22 (m, 14H), ^{13}C NMR (126 MHz, DMSO-d_6): 172.9 (C), 172.6 (2xC), 172.1 (C), 171.9 (C), 171.8 (C), 171.6 (C), 171.5 (C), 171.4 (C), 170.3 (2xC), 170.2 (3xC), 162.7 (C), 144.5 (C), 122.9 (CH), 70.2 (CH_2), 69.6 (CH_2), 69.4 (CH_2), 69.0 (CH_2), 61.0 (CH), 59.2 (CH), 55.4 (CH), 53.6 (CH), 53.3 (CH), 52.8 (CH), 52.8 (CH), 52.6 (CH), 52.5 (CH), 51.5 (6xCH), 48.7 (CH_2), 45.4 (CH_2), 42.1 (12xCH_2), 39.8 (CH_2), 38.9 (CH_2), 38.8 (5xCH_2), 38.7 (CH_2), 38.7 (CH_2), 35.1 (CH_2), 34.3 (CH_2), 32.0 (CH_2), 31.6 (CH_2), 31.3 (2xCH_2), 31.1 (CH_2), 30.9 (CH_2), 30.7 (2xCH_2), 30.5 (CH_2), 29.1 (CH_2), 28.7 (3xCH_2), 28.6 (3xCH_2), 28.1 (CH_2), 28.0 (CH_2), 26.1 (CH_2), 25.2 (CH_2), 24.9 (CH_2), 23.1 (CH_2), 22.9 (CH_2), 22.8 (CH_2), 22.8 (CH_2),

22.7 (2xCH$_2$); HRMS (ESI, +ve) calcd for $C_{89}H_{145}ClN_{20}O_{18}S_{13}$ ([M + 2H]$^{2+}$): 1117.3625, found: 1117.3591.

2.1.9. Synthesis of peptide 13

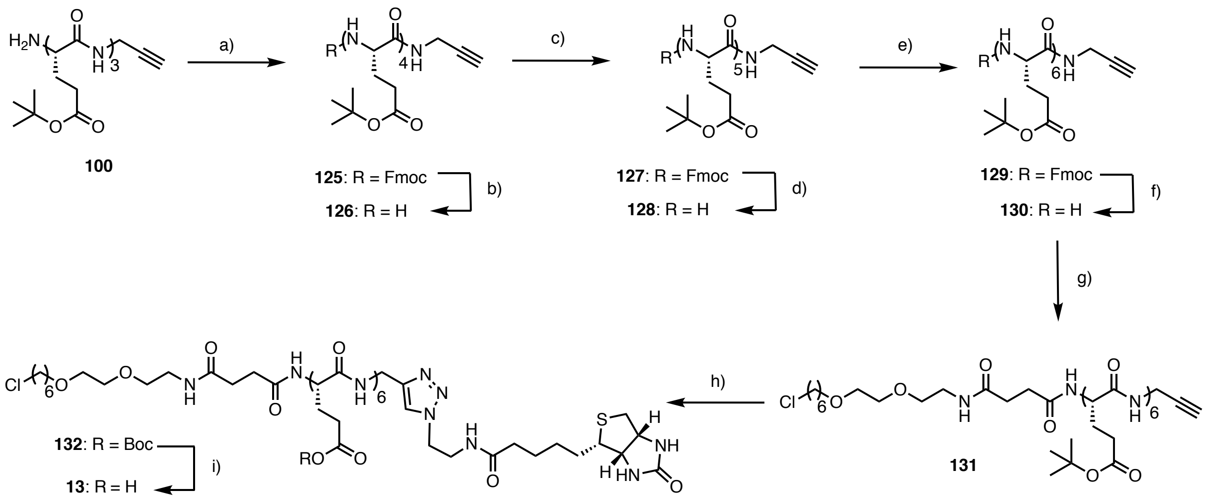

Scheme S9 (a) Fmoc-Glu(O*t*Bu)-OH, HATU, DIPEA, DMF, rt, 30 min, 88%; (b) 2 M dimethylamine in THF, rt, 30 min, *quant.*; (c) Fmoc-Glu(O*t*Bu)-OH, HATU, DIPEA, DMF, rt, 30 min, 79%; (d)) 2 M dimethylamine in THF, rt, 30 min, *quant.*; (e) Fmoc-Glu(O*t*Bu)-OH, HATU, DIPEA, DMF, rt, 30 min, 90%; (f) 2 M dimethylamine in THF, rt, 30 min, *quant.*; (g) **35**, HATU, DIPEA, DMF, rt, 30 min, 77%; (h) **36**, $CuSO_4{\cdot}5H_2O$, Na-ascorbate, TBTA, THF/H$_2$O, rt, 1 h, 83%; (i) TFA/TIPS/H$_2$O 95/2.5/2.5, rt, 30 min, 76%.

Compound 125. To a solution of **100** (919 mg, 1.51 mmol) in dry DMF (4 mL), was added a 30 seconds-premixed solution of HATU (686 mg, 1.81 mmol), Fmoc-Glu(O*t*Bu)-OH (768 mg, 1.81 mmol) and DIPEA (315 µL, 1.81 mmol) in dry DMF (4 mL). Reaction mixture was stirred at rt for 30 min. Reaction mixture was diluted with EtOAc and washed with NaHCO$_3$ sat. aq. solution (x3), 10% citric acid solution (x3), water (x1), 5% LiCl solution (x2) and brine. Organic phase was then dried with Na$_2$SO$_4$, filtered and concentrated. Crude was dissolved in a minimal amount of CH$_2$Cl$_2$ and precipitated in pentane (x3). Crude was then

purified by flash column chromatography (BGB® Scorpius 25 g SiO_2 col., 40 mL/min, linear gradient 0 – 8% MeOH in CH_2Cl_2) to yield **125** as a colorless solid (1.34 g, 88%). R_f (CH_2Cl_2 + 10% MeOH): 0.8; Mp: 197.9 – 198.4 °C; $[\alpha]_D^{20}$ – 25.5 (*c* 0.50, MeOH); IR (neat): 3281 (m), 2979 (m), 1728 (s), 1628 (s), 1519 (s), 1451 (m), 1366 (m), 1254 (m), 1148 (s), 847 (m), 740 (m), 686 (m); ^1H NMR (400 MHz, CD_3OD): 7.80 (d, $^3J_{H-H}$ = 7.5 Hz, 2H), 7.68 (d, $^3J_{H-H}$ = 7.5 Hz, 2H), 7.40 (t, $^3J_{H-H}$ = 7.5 Hz, 2H), 7.32 (t, $^3J_{H-H}$ = 7.5 Hz, 2H), 4.44 (dd, $^3J_{H-H}$ = 10.5, 7.0 Hz, 1H), 4.37 (dd, $^3J_{H-H}$ = 10.5, 6.7 Hz, 1H), 4.35 – 4.27 (m, 3H), 4.24 (t, $^3J_{H-H}$ = 6.9 Hz, 1H), 4.07 (dd, $^3J_{H-H}$ = 8.3, 6.0 Hz, 1H), 3.99 (dd, $^2J_{H-H}$ = 17.4, $^4J_{H-H}$ = 2.6 Hz, 1H), 3.93 (dd, $^2J_{H-H}$ = 17.4, $^4J_{H-H}$ = 2.6 Hz, 1H), 2.56 (t, $^4J_{H-H}$ = 2.6 Hz, 1H), 2.42 – 2.24 (m, 8H), 2.18 – 2.00 (m, 4H), 2.00 – 1.84 (m, 4H), 1.45 (s, 9H), 1.42 (s, 18H), 1.41 (s, 9H); ^{13}C NMR (101 MHz, CD_3OD): 175.0 (C), 174.1 (C), 174.1 (C), 173.9 (C), 173.8 (C), 173.8 (C), 173.6 (C), 173.2 (C), 158.9 (C), 145.2 (C), 145.2 (C), 142.6 (2xC), 128.8 (2xCH), 128.2 (2xCH), 126.3 (CH), 126.2 (CH), 121.0 (2xCH), 81.9 (C), 81.9 (C), 81.9 (C), 81.8 (C), 80.4 (C), 72.3 (CH), 68.2 (CH_2), 56.5 (CH), 54.9 (CH), 54.5 (CH), 54.0 (CH), 48.4 (CH), 32.7 (CH_2), 32.6 (3xCH_2), 29.6 (CH_2), 28.4 (3xCH_3), 28.4 (9xCH_3), 28.2 (CH_2), 28.0 (CH_2), 27.9 (CH_2), 27.6 (CH_2).

Compound 126 was prepared following the general procedure of Fmoc deprotection, starting from **125** (1.27 g, 1.25 mmol) in 10 mL of 2 M dimethylamine in THF, yielding **126** as a colorless solid (993 mg, 100%).

Compound 127. To a solution of **126** (920 mg, 1.16 mmol) in dry DMF (3 mL), was added a 30 seconds-premixed solution of HATU (529 mg, 1.39 mmol), Fmoc-Glu(O*t*Bu)-OH (590 mg, 1.39 mmol) and DIPEA (242 µL, 1.39 mmol) in dry DMF (3 mL). Reaction mixture was stirred at rt for 30 min. Reaction mixture was diluted with EtOAc and washed with $NaHCO_3$ sat. aq. solution (x3), 10% citric acid solution (x3), water (x1), 5% LiCl solution (x2) and brine. Organic phase was then dried with Na_2SO_4, filtered and concentrated. Crude was dissolved in a minimal amount of CH_2Cl_2 and precipitated in pentane (x3). Crude was then

purified by flash column chromatography (BGB® Scorpius 25 g SiO_2 col., 40 mL/min, linear gradient 0 – 8% MeOH in CH_2Cl_2) to yield **127** as a colorless solid (1.09 g, 79%). R_f (CH_2Cl_2 + 10% MeOH): 0.7; Mp: 210.2 – 211.0 °C; $[\alpha]_D^{20}$ – 21.2 (*c* 0.50, MeOH); IR (neat): 3286 (m), 2977 (m), 1728 (s), 1626 (s), 1520 (s), 1452 (m), 1366 (m), 1254 (m), 1149 (s), 1054 (s), 1014 (s), 847 (m), 740 (m), 692 (s); ^{1}H NMR (400 MHz, CD_3OD): 7.80 (d, $^3J_{H-H}$ = 7.4 Hz, 2H), 7.69 (d, $^3J_{H-H}$ = 7.4 Hz, 2H), 7.40 (t, $^3J_{H-H}$ = 7.4 Hz, 2H), 7.32 (t, $^3J_{H-H}$ = 7.4 Hz, 2H), 4.47 (t, $^3J_{H-H}$ = 8.9 Hz, 1H), 4.38 (t, $^3J_{H-H}$ = 8.9 Hz, 1H), 4.35 – 4.20 (m, 5H), 4.09 – 4.04 (m, 1H), 4.02 – 3.89 (m, 2H), 2.55 (t, $^4J_{H-H}$ = 2.4 Hz, 1H), 2.42 – 2.25 (m, 10H), 2.20 – 1.87 (m, 10H), 1.45 (s, 9H), 1.43 (s, 9H), 1.42 (s, 9H), 1.42 (s, 9H), 1.41 (s, 9H); ^{13}C NMR (101 MHz, CD_3OD): 175.5 (C), 174.6 (C), 174.3 (C), 174.0 (C), 173.9 (2xC), 173.8 (C), 173.7 (C), 173.2 (C), 159.0 (C), 145.2 (C), 145.1 (C), 142.7 (2xC), 128.9 (2xCH), 128.2 (2xCH), 126.3 (CH), 126.2 (CH), 121.0 (2xCH), 81.9 (C), 81.9 (2xC), 81.8 (C), 81.7 (C), 80.4 (C), 72.3 (CH), 68.3 (CH_2), 56.9 (CH), 55.7 (CH), 55.3 (CH), 54.7 (CH), 54.1 (CH), 48.4 (CH), 32.9 (CH_2), 32.8 (CH_2), 32.7 (CH_2), 32.7 (CH_2), 32.6 (CH_2), 29.6 (CH_2), 28.4 (6xCH_3), 28.4 (9xCH_3), 28.2 (CH_2), 27.8 (2xCH_2), 27.5 (CH_2), 27.3 (CH_2).

Compound 128 was prepared following the general procedure of Fmoc deprotection, starting from **127** (1.04 g, 0.864 mmol) in 10 mL of 2 M dimethylamine in THF, yielding **128** as a colorless solid (860 mg, *quant.*).

Compound 129. To a solution of **128** (800 mg, 0.815 mmol) in dry DMF (2 mL), was added a 30 seconds-premixed solution of HATU (372 mg, 0.978 mmol), Fmoc-Glu(O*t*Bu)-OH (416 mg, 0.978 mmol) and DIPEA (170 µL, 0.978 mmol) in dry DMF (3 mL). Reaction mixture was stirred at rt for 30 min. Reaction mixture was diluted with EtOAc and washed with $NaHCO_3$ sat. aq. solution (x3), 10% citric acid solution (x3), water (x1), 5% LiCl solution (x2) and brine. Organic phase was then dried with Na_2SO_4, filtered and concentrated. Crude was dissolved in a minimal amount of CH_2Cl_2 and precipitated in pentane (x3). Crude was then

purified by flash column chromatography (BGB® Scorpius 25 g SiO_2 col., 40 mL/min, linear gradient 0 – 8% MeOH in CH_2Cl_2) to yield **129** as a colorless solid (1.02 g, 90%). R_f (CH_2Cl_2 + 10% MeOH): 0.8; Mp: 236.1 – 236.9 °C; $[\alpha]_D^{20}$ – 8.4 (*c* 0.50, DMSO); IR (neat): 3292 (m), 2977 (m), 1728 (s), 1626 (s), 1520 (s), 1452 (m), 1366 (m), 1253 (m), 1253 (m), 1148 (s), 1053 (m), 847 (m), 740 (m), 691 (m); ^{1}H NMR (400 MHz, CD_3OD): 7.81 (d, $^3J_{H-H}$ = 7.5 Hz, 2H), 7.69 (d, $^3J_{H-H}$ = 7.5 Hz, 2H), 7.40 (t, $^3J_{H-H}$ = 7.5 Hz, 2H), 7.32 (t, $^3J_{H-H}$ = 7.5 Hz, 2H), 4.52 (dd, $^2J_{H-H}$ = 10.5, $^3J_{H-H}$ = 6.9 Hz, 1H), 4.40 (dd, $^2J_{H-H}$ = 10.5, $^3J_{H-H}$ = 6.9 Hz, 1H), 4.32 (dd, $^3J_{H-H}$ = 9.8, 4.6 Hz, 1H), 4.28 – 4.22 (m, 2H), 4.21 – 4.16 (m, 2H), 4.14 (dd, $^3J_{H-H}$ = 8.7, 6.2 Hz, 1H), 4.05 (t, $^3J_{H-H}$ = 7.3 Hz, 1H), 3.97 (d, $^4J_{H-H}$ = 2.5 Hz, 2H), 2.54 (t, $^4J_{H-H}$ = 2.5 Hz, 1H), 2.49 – 2.27 (m, 12H), 2.21 – 1.91 (m, 12H), 1.45 (s, 9H), 1.44 (s, 9H), 1.43 (s, 18H), 1.40 (s, 9H), 1.39 (s, 9H); ^{13}C NMR (126 MHz, DMSO-*d*$_6$): 171.7 (C), 171.7 (C), 171.6 (C), 171.6 (2xC), 171.5 (C), 171.4 (C), 171.0 (C), 170.9 (C), 170.9 (C), 170.8 (C), 170.6 (C), 156.0 (C), 143.8 (C), 143.7 (C), 140.7 (2xC), 127.6 (2xCH), 127.0 (2xCH), 125.3 (2xCH), 120.1 (CH), 120.1 (CH), 80.8 (C), 79.7 (2xC), 79.6 (C), 79.6 (2xC), 79.6 (C), 73.1 (CH), 65.7 (CH_2), 53.9 (CH), 51.8 (2xCH), 51.8 (CH), 51.8 (CH), 51.6 (CH), 46.6 (CH), 31.4 (CH_2) 31.3 (3xCH_2), 31.2 (CH_2), 31.1 (CH_2), 28.0 (CH_2), 27.7 (6xCH_3), 27.7 (12xCH_3), 27.3 (CH_2), 27.3 (4xCH_2), 27.2 (CH_2).

Compound 130 was prepared following the general procedure of Fmoc deprotection, starting from **129** (504 mg, 0.363 mmol) in 9 mL of 2 M dimethylamine in THF, yielding **130** as a colorless solid (450 mg, *quant.*).

Compound 131. To a solution of **130** (349 mg, 0.299 mmol) in dry DMF (1 mL), was added a 30 seconds-premixed solution of HATU (137 mg, 0.359 mmol), **35** (116 mg, 0.359 mmol) and DIPEA (63 µL, 0.359 mmol) in dry DMF (1 mL). Reaction mixture was stirred at rt for 30 min. Reaction mixture was diluted with EtOAc and washed with $NaHCO_3$ sat. aq. solution (x3), 10% citric acid solution (x3), water (x1), 5% LiCl solution (x2) and brine.

Organic phase was then dried with Na_2SO_4, filtered and concentrated. Crude was dissolved in a minimal amount of CH_2Cl_2 and precipitated in pentane (x3). Crude was then purified by flash column chromatography (BGB® Scorpius 25 g SiO_2 col., 40 mL/min, linear gradient 0 – 8% MeOH in CH_2Cl_2) to yield **131** as a colorless solid (340 mg, 77%). R_f (CH_2Cl_2 + 10% MeOH): 0.5; Mp: 226.6 – 227.2 °C; $[\alpha]_D^{20}$ + 9.7 (*c* 0.50, MeOH); IR (neat): 3283 (m), 2977 (m), 1727 (s), 1624 (s), 1522 (s), 1450 (m), 1366 (m), 1254 (m), 1148 (s), 847 (m), 698 (m); ^{1}H NMR (400 MHz, CD_3OD): 4.33 (dd, $^3J_{H-H}$ = 10.3, 4.3 Hz, 1H), 4.22 (dd, $^3J_{H-H}$ = 7.8, 6.8 Hz, 1H), 4.14 – 4.01 (m, 4H), 3.98 (t, $^4J_{H-H}$ = 2.5 Hz, 2H), 3.63 – 3.52 (m, 6H), 3.56 (t, $^3J_{H-H}$ = 6.6 Hz, 2H), 3.49 (t, $^3J_{H-H}$ = 6.6 Hz, 2H), 3.46 – 3.34 (m, 2H), 2.87 – 2.77 (m, 1H), 2.59 – 2.48 (m, 3H), 2.51 (t, $^4J_{H-H}$ = 2.5 Hz, 1H), 2.47 – 2.30 (m, 12H), 2.23 – 2.10 (m, 6H), 2.08 – 1.92 (m, 6H), 1.83 – 1.73 (m, 2H), 1.65 – 1.56 (m, 2H), 1.52 – 1.34 (m, 4H), 1.45 (s, 9H), 1.44 (s, 36H), 1.44 (s, 9H); ^{13}C NMR (126 MHz, DMSO-d_6): 172.8 (C), 172.1 (C), 171.9 (C), 171.7 (C), 171.6 (C), 171.5 (2xC), 171.5 (3xC), 171.2 (C), 171.0 (C), 170.9 (C), 170.6 (C), 80.8 (C), 79.6 (3xC), 79.6 (C), 79.6 (C), 79.6 (C), 73.0 (CH), 70.2 (CH_2), 69.6 (CH_2), 69.4 (CH_2), 69.0 (CH_2), 52.9 (CH), 52.6 (CH), 52.1 (CH), 52.0 (CH), 51.9 (CH), 51.7 (CH), 45.3 (CH_2), 38.6 (CH_2), 32.0 (CH_2), 31.4 (CH_2), 31.3 (CH_2), 31.3 (CH_2), 31.2 (CH_2), 31.2 (CH_2), 31.1 (CH_2), 30.8 (CH_2), 30.5 (CH_2), 29.1 (CH_2), 28.0 (CH_2), 27.7 (18xCH_3), 27.2 (CH_2), 27.1 (CH_2), 27.0 (CH_2), 26.8 (CH_2), 26.7 (CH_2), 26.4 (CH_2), 26.1 (CH_2), 24.9 (CH_2).

Compound 132. To a solution of **131** (232 mg, 0.158 mmol) in THF/H_2O 2/1 (5 mL), **36** (59 mg, 0.19 mmol), $CuSO_4{\cdot}5H_2O$ (39 mg, 0.16 mmol), sodium ascorbate (62 mg, 0.31 mmol) and TBTA (8.4 mg, 16 µmol) were added and the reaction mixture was stirred at rt for 1 h. Solvent was removed *in vacuo*, H_2O and CH_2Cl_2 were added, phases were separated, and aqueous phase was extracted by CH_2Cl_2 (x3). Organic phases were collected together, washed with brine (x1), dried over Na_2SO_4, filtered and concentrated. Crude was purified by flash column chromatography (BGB® Scorpius 25g, 25 mL/min, linear gradient 0 – 10% MeOH in

CH_2Cl_2) to yield **132** as a colorless solid (235 mg, 83%). R_f (CH_2Cl_2 + 10% MeOH): 0.2; Mp: 191.6 – 192.1 °C; $[\alpha]_D^{20}$ +20.7 (*c* 0.50, MeOH); IR (neat): 3296 (m), 2934 (m), 1726 (s), 1645 (s), 1535 (s), 1451 (m), 1366 (s), 1150 (s), 845 (m), 563 (m); ^1H NMR (400 MHz, CD_3OD): 7.95 (s, 1H), 4.64 (d, $^2J_{H-H}$ = 15.2 Hz, 1H), 4.55 – 4.47 (m, 3H), 4.43 – 4.37 (m, 1H), 4.35 (dd, $^3J_{H-H}$ = 7.9, 4.4 Hz, 1H), 4.28 (dd, $^3J_{H-H}$ = 10.2, 4.4 Hz, 1H), 4.16 (dd, $^3J_{H-H}$ = 9.6, 5.2 Hz, 1H), 4.13 – 4.08 (m, 1H), 4.05 – 3.95 (m, 2H), 3.74 – 3.61 (m, 2H), 3.62 – 3.53 (m, 6H), 3.56 (t, $^3J_{H-H}$ = 6.6 Hz, 2H), 3.49 (t, $^3J_{H-H}$ = 6.6 Hz, 2H), 3.46 – 3.34 (m, 2H), 3.24 (ddd, $^3J_{H-H}$ = 8.6, 6.2, 4.3 Hz, 1H), 2.96 (dd, $^2J_{H-H}$ = 12.7, $^4J_{H-H}$ = 5.0 Hz, 1H), 2.88 – 2.77 (m, 1H), 2.72 (d, $^2J_{H-H}$ = 12.7 Hz, 1H), 2.61 – 2.31 (m, 15H), 2.25 – 1.96 (m, 14H), 1.82 – 1.68 (m, 3H), 1.67 – 1.54 (m, 5H), 1.52 – 1.35 (m, 9H), 1.45 (s, 18H), 1.44 (s, 9H), 1.44 (s, 18H), 1.42 (s, 9H); ^{13}C NMR (126 MHz, DMSO-d_6): 172.9 (C), 172.5 (C), 172.2 (C), 171.9 (C), 171.7 (2xC), 171.6 (C), 171.6 (C), 171.5 (C), 171.5 (C), 171.5 (C), 171.4 (C), 171.2 (C), 171.0 (C), 170.8 (C), 162.7 (C), 144.6 (C), 122.9 (CH), 79.7 (2xC), 79.6 (C), 79.6 (3xC), 70.2 (CH_2), 69.6 (CH_2), 69.4 (CH_2), 69.0 (CH_2), 61.0 (CH), 59.2 (CH), 55.4 (CH), 53.0 (CH), 52.8 (CH), 52.3 (2xCH), 52.1 (CH), 51.9 (CH), 48.7 (CH_2), 45.4 (CH_2), 39.6 (CH_2), 38.9 (CH_2), 38.6 (CH_2), 35.0 (CH_2), 34.4 (CH_2), 32.0 (CH_2), 31.4 (CH_2), 31.3 (2xCH_2), 31.2 (CH_2), 31.2 (2xCH_2), 30.8 (CH_2), 30.5 (CH_2), 29.1 (CH_2), 28.1 (CH_2), 28.0 (CH_2), 27.7 (18xCH_3), 27.2 (CH_2), 27.0 (CH_2), 26.9 (CH_2), 26.7 (CH_2), 26.6 (CH_2), 26.3 (CH_2), 26.1 (CH_2), 25.1 (CH_2), 24.9 (CH_2).

Compound 13. A solution of **132** (166 mg, 93.0 µmol) in TFA/H_2O/TIPS 95/2.5/2.5 (2 mL) was stirred at rt for 30 min. Solvent was removed *in vacuo*, crude was dissolved in small amount of MeOH and was precipitated in Et_2O (x3) to yield **13** as a colorless solid (104 mg, 76%). ^1H NMR (500 MHz, DMSO-d_6): 12.07 (br s, 6H), 8.34 (t, $^3J_{H-H}$ = 5.7 Hz, NH), 8.18 (d, $^3J_{H-H}$ = 7.1 Hz, NH), 8.05 (d, $^3J_{H-H}$ = 7.5 Hz, NH), 8.00 – 7.93 (m, 4xNH), 7.88 (d, $^3J_{H-H}$ = 7.4 Hz, NH), 7.85 (s, 1H), 7.83 (d, $^3J_{H-H}$ = 7.4 Hz, NH), 6.42 (s, NH), 6.36 (s, NH), 4.37 (t, $^3J_{H-H}$ = 6.2 Hz, 2H), 4.34 – 4.29 (m, 2H), 4.28 – 4.11 (m, 8H), 3.62 (t, $^3J_{H-H}$ = 6.6 Hz, 2H), 3.51 –

3.43 (m, 6H), 3.40 – 3.27 (m, 4H), 3.21 – 3.15 (m, 2H), 3.10 (ddd, $^3J_{H\text{-}H}$ = 8.5, 6.3, 4.4 Hz, 1H), 2.82 (dd, $^2J_{H\text{-}H}$ = 12.4, $^3J_{H\text{-}H}$ = 5.1 Hz, 1H), 2.57 (d, $^2J_{H\text{-}H}$ = 12.4 Hz, 1H), 2.40 – 2.31 (m, 4H), 2.29 – 2.15 (m, 12H), 2.04 (t, $^3J_{H\text{-}H}$ = 7.5 Hz, 2H), 1.97 – 1.84 (m, 6H), 1.83 – 1.66 (m, 8H), 1.64 – 1.56 (m, 1H), 1.52 – 1.43 (m, 5H), 1.42 – 1.33 (m, 2H), 1.32 – 1.24 (m, 4H); ^{13}C NMR (126 MHz, DMSO-d_6): 174.0 (C), 174.0 (C), 173.9 (2xC), 173.9 (C), 172.6 (C), 172.5 (C), 171.9 (C), 171.8 (C), 171.4 (C), 171.2 (C), 171.2 (C), 171.1 (C), 171.0 (C), 162.8 (C), 144.6 (C), 122.9 (CH), 70.2 (CH_2), 69.6 (CH_2), 69.4 (CH_2), 69.1 (CH_2), 61.0 (CH), 59.2 (CH), 55.4 (CH), 52.5 (CH), 52.4 (CH), 52.1 (4xCH), 48.7 (CH_2), 45.4 (CH_2), 39.6 (CH_2), 38.9 (CH_2), 38.6 (CH_2), 35.0 (CH_2), 34.4 (CH_2), 32.0 (CH_2), 30.7 (CH_2), 30.5 (CH_2), 30.3 (CH_2), 30.2 (CH_2), 30.2 (CH_2), 30.1 (3xCH_2), 29.1 (CH_2), 28.1 (CH_2), 28.0 (CH_2), 27.2 (CH_2), 27.2 (2xCH_2), 27.0 (CH_2), 26.9 (CH_2), 26.7 (CH_2), 26.1 (CH_2), 25.1 (CH_2), 24.9 (CH_2). HRMS (ESI, +ve) calcd for $C_{59}H_{91}ClN_{14}O_{24}S$ ($[M + 2H]^{2+}$): 724.2943, found: 724.2935.

2.2. General procedure for streptavidin adducts formation

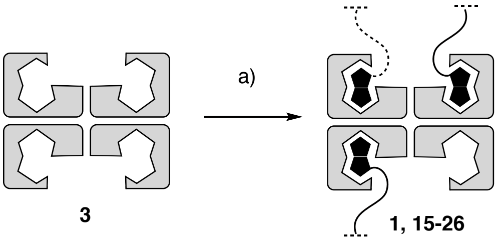

Scheme S10 a) **4-14**, PBS, rt, 10 min.

Complexes 1, 15-21. To a freshly prepared solution of WT streptavidin **3** (20 µM in PBS, 1 mL), the different peptides **4-11** (6 µL, 10 mM in DMSO, 3 equivalents) were added and the mixture was shaken for 10 min at rt. Then, the complexes **1, 15-26** were concentrated and washed with PBS (3 x 0.5 mL) using Amicon® Ultra 0.5 mL centrifugal filters (cut off: 30 kDa, 10 min, 4.0 krpm). The material was recovered from the centrifugal filter and diluted again with PBS to the required concentration (i.e., 200 µM, 100 µL).

Complex 22 was similarly prepared using 6 μL of peptide **12** (10 mM in DMSO, 3 equivalents).

Complex 23 was similarly prepared using 4 μL of peptide **13** (10 mM in DMSO, 2 equivalents).

Complex 24 was similarly prepared using 2 μL each of peptides **12** and **13** (10 mM in DMSO, 1 equivalent each).

Complex 25 was similarly prepared using 4 μL of peptide **14** (10 mM in DMSO, 2 equivalents).

Complex 26 was similarly prepared using 2 μL each of peptides **13** and **14** (10 mM in DMSO, 1 equivalent each).

3. HC-CAPA

3.1. HGM cell line

HeLa cells stably expressing the HaloTag-GFP-Mito fusion protein (HGM): The cells were originally designed by the Chenoweth lab as described in ref. S10. They were cultured using the described procedure in ref. S4.

3.2. 2D HC-CAPA

3.2.1 General HC-CAPA protocol

As in ref. S11, HGM cells were seeded at 8 ×10^4 cells/mL in FluoroBrite DMEM + 10% FBS on μ-Plates 96-well ibiTreat sterile and kept at 37 °C with 5% CO_2 overnight. Next day, cells were washed with PBS (3 × 3 mL/well) and the media were exchanged to Leibovitz's (4 × 150 μL/well) using a plate washer, keeping a final volume of 135 μL/well. Then, serial dilutions of the corresponding complexes **1**, **15-26** in PBS were prepared in a 96-well V-bottom plate and added to the μ-Plate containing the cells (15 μL/well, 10x final concentration in PBS) to reach a final volume of 150 μL/well (0 to maximum 20 μM). Cells were incubated for 4 h at

37 °C with 5% CO_2. After this, cells were washed again and HRO **2** was added (15 µL/well, 50 µM in PBS) to reach a final volume of 150 µL/well (5 µM), except for the control wells, where only PBS was added (15 µL/well). After 15 min of incubation at 37 °C with 5% CO_2, the plate was washed again. Then, Hoechst 33342 was added (15 µL/well, 170 µM in PBS) to reach a final volume of 150 µL/well (17 µM). After 15 min of incubation at 37 °C with 5% CO_2, the plate was washed one last time and the cells were kept in clean Leibovitz's media. During imaging, samples were kept at 37 °C with 5% CO_2. A total of 16 images/well at 40x were recorded, using three channels: blue (excitation filter: 377/50 nm, emission filter: 477/60 nm, exposure time: 10 ms), green (excitation filter: 475/34 nm, emission filter: 536/40 nm, exposure time: 20 ms) and red (excitation filter: 531/40 nm, emission filter: 593/40 nm, exposure time: 30 ms), as shown in Figure S2. Duplicates were performed for each condition.

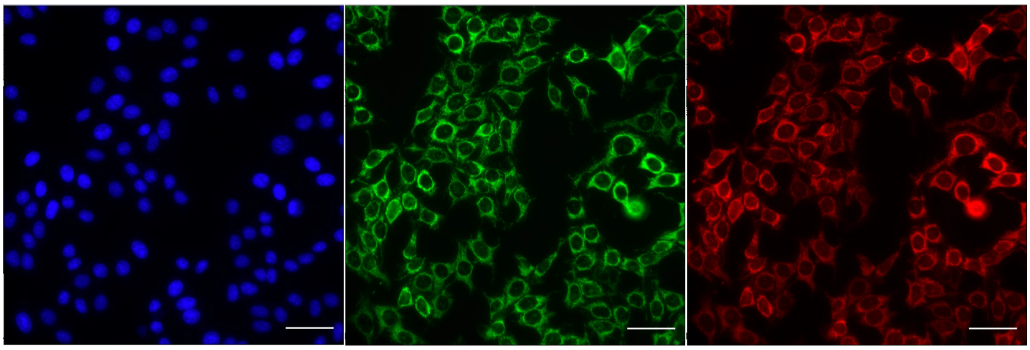

Fig. S2 Blue channel recording the Hoechst 33342 localization in the nuclei (left), green channel recording the GFP signal in the mitochondria (middle) and red channel recording the signal from HRO **2** (right) covalently attached to HaloTag protein in the mitochondria. Scale bar: 50 µm.

3.2.2. HC-CAPA analysis protocol

As in ref. S11, for each cell, the DAPI and GFP channel images were used for the segmentation of the nuclei and whole cell body, respectively. Three successive filters have then been used for the cell selection process:

1) Cells positively expressing GFP (GFP channel with minimum intensity threshold).

2) The shape of the nucleus, to eliminate dead or dividing cell (maximum roundness of 0.7, with roundness = height / length). Cells partially outside of the picture were also removed.

3) The size of the nucleus, an additional filter for dividing and dead cells (minimum area of 130 μm^2).

The mitochondrial mask was then applied to extract the integrated intensity values (sum of the intensities of the pixels included in the mask) in the red channel image, from the labeling with compound HRO **2** (Figure S3).

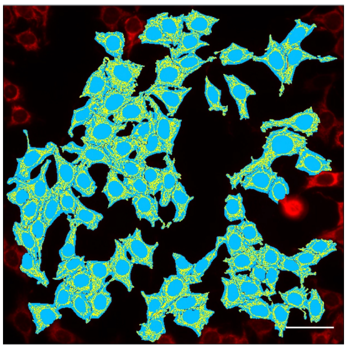

Fig. S3 Masks applied for the quantification of the fluorescence intensity of HRO **2**, in the red channel image. Cell body (light blue) and mitochondria (yellow). Scale bar: 50 µm.

3.2.3. HC-CAPA results for uptake quantification

HC-CAPA was performed as in section 3.2. As in ref. S11, the integrated intensity values in the mitochondria for each condition using optimized HC-CAPA were then normalized using the value of integrated intensity with addition of HRO **2** (0 µM of complexes **1, 15-26**) as maximum signal (I_{rel} = 1) and the value of integrated intensity without addition of

HRO **2** (0 μM of complexes **1, 15-26**) as minimum (I_{rel} = 0), for each set of experiments. Duplicates were performed for each condition. The resulting dependence of the relative intensity values (I_{rel}) to the concentration of complexes **1, 15-26** (*c*) was plotted and fitted with equation (S1) to retrieve the half-maximal penetration concentration (CP_{50}) value (*n* is the *Hill* coefficient).

$$I_{rel} = 1 / (1 + (c / CP_{50})^n) \qquad (S1)$$

3.3. 3D HC-CAPA

3.3.1 3D HC-CAPA protocol

HGM cells were seeded at 5 × 10^3 cells/mL in FluoroBrite DMEM + 10% FBS on Nunclon™ Sphera™ U-bottom 96-well sterile μ-Plates and kept at 37 °C with 5% CO_2 for 3 days. After the spheroids were formed, the medium was exchanged to Leibovitz's (6 × 200 μL/well) using a Biotek MultiFlo™ FX plate washer, keeping a final volume of 135 μL/well. Then, serial dilutions of the corresponding complexes **18-20** in PBS were prepared in a 96-well V-bottom plate and added to the μ-Plate containing the spheroids (15 μL/well, 10x final concentration in PBS) to reach a final volume of 150 μL/well (0 to maximum 20 μM). Cells were incubated for 2 or 6 h at 37 °C with 5% CO_2. After this, cells were washed again and HRO **2** was added (15 μL/well, 50 μM in PBS) to reach a final volume of 150 μL/well (5 μM), except for the control wells, where only PBS was added (15 μL/well). After 45 min of incubation at 37 °C with 5% CO_2, the plate was washed again. Then, the plate was kept for 1 h at 37 °C with 5% CO_2 and was washed again. During imaging, samples were kept at 37 °C with 5% CO_2. A z-stack of 19 images/well (10 μm per step) at 10x were recorded, using two channels: green (excitation filter: 475/34 nm, emission filter: 536/40 nm, exposure time: 200 ms) and red (excitation filter: 531/40 nm, emission filter: 593/40 nm, exposure time: 200 ms), as shown in Figure S4. Duplicates were performed for each condition.

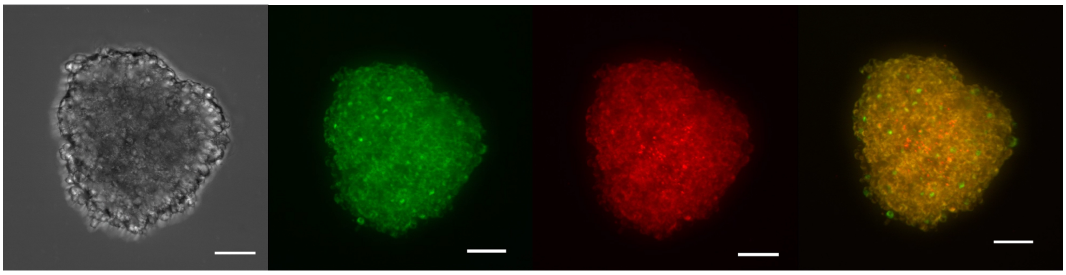

Fig. S4 TL channel (left), Green channel recording the GFP signal in the mitochondria (middle left) and red channel recording the signal from HRO **2** (middle right) covalently attached to HaloTag protein in the mitochondria. Merged images showing colocalization of GFP and HRO **2** signals (right). Images are 2D projections of all planes. Scale bar: 100 µm.

3.3.2 3D HC-CAPA analysis protocol

For each spheroid, the TL and green channel images were used for the segmentation of the object. A gaussian filter was applied to blur the image and allow the creation of the spheroid body mask (Figure S5).

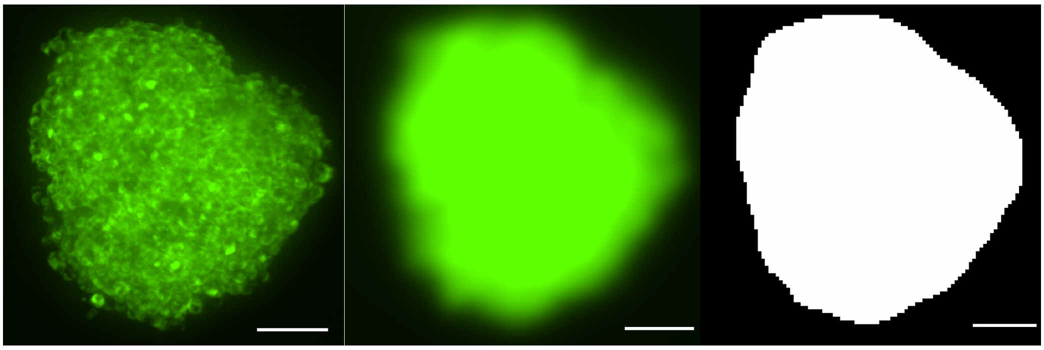

Fig. S5 Green channel recording the GFP signal in the mitochondria (left), applied Gaussian filter (middle) to generate the spheroid mask in white (right). Images are 2D projections of all planes. Scale bar: 100 µm.

Spheroid mask was applied to extract the average intensity values (average of the intensities of the pixels included in the mask) in the red channel image, from the labeling with compound HRO **2** (Figure S5).

3.3.2 3D HC-CAPA results for uptake quantification

HC-CAPA was performed as in section 3.3.2. The average intensity values, extracted from the mask analysis, for each condition using optimized 3D HC-CAPA were then normalized using the value of average intensity with addition of HRO **2** (0 μM of complexes **18-20**) as maximum signal (I_{rel} = 1) and the value of average intensity with addition of ct-COOH **35** (100 μM) as minimum (I_{rel} = 0), for each set of experiments. Duplicates were performed for each condition.

The resulting dependence of the relative intensity values (I_{rel}) to the concentration of complexes **18-20** (*c*) was plotted and fitted with equation (S2) to retrieve the half-maximal spheroid penetration concentration (SP_{50}) value (*n* is the *Hill* coefficient).

$$I_{rel} = 1 / (1 + (c / SP_{50})^n) \qquad (S2)$$

4. Delivery of QDs and SupraFlipper in spheroids

4.1. Quantum dot delivery in spheroids

4.1.1. Quantum dot adduct formation

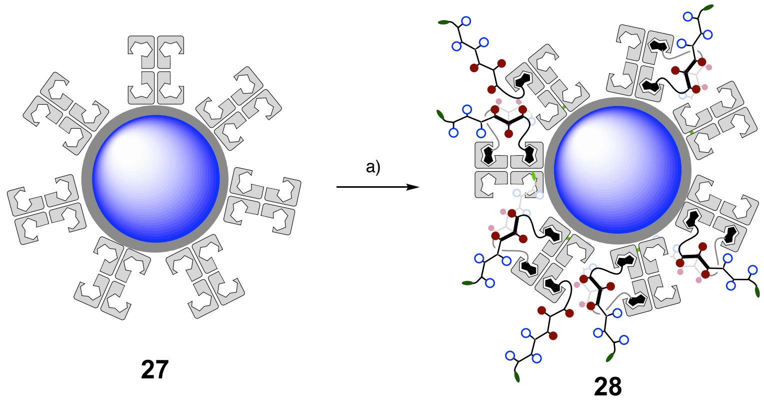

27

28

Scheme S11 a) 80 eq. of **5**, PBS, 4 °C, 1 h.

Complex 28 was prepared following the procedure reported in ref. S12. To a solution of Qdot™ 605 streptavidin conjugate **27** (1 μM in PBS, 3 μL), **5** (24 μL, 10 μM in PBS, 80 equivalents) and 3 μL PBS (final concentration = 100 nM) were added and the mixture was shaken for 1 h at 4 °C to form **28** (Scheme S11).

4.1.2. Quantum dot delivery protocol

As in section 3.3.1., HGM cells were seeded at 5 × 10^3 cells/mL in FluoroBrite DMEM + 10% FBS on Nunclon™ Sphera™ U-bottom 96-well sterile μ-Plates and kept at 37 °C with 5% CO_2 for 3 days. After the spheroids were formed, the medium was exchanged to Leibovitz's (6 × 200 μL/well) using a Biotek MultiFlo™ FX plate washer, keeping a final volume of 135 μL/well. Then, QDs complexes **27** and **28** in PBS were added to the μ-Plate containing the spheroids (15 μL/well, 10x final concentration in PBS) to reach a final volume of 150 μL/well (final concentration = 10 nM), except for the control wells, where only PBS was added (15 μL/well). Spheroids were incubated for 6 h at 37 °C with 5% CO_2. After this, spheroids were washed and imaged with a z-stack of 19 images/well (10 μm per step) at 10x, using three channels: Transmitted light, green (excitation filter: 475/34 nm, emission filter: 536/40 nm, exposure time: 200 ms) and GFP-TxRed (excitation filter: 475/34 nm, emission filter: 641/75 nm, exposure time: 200 ms), as shown in Figure S6. During imaging, samples were kept at 37 °C with 5% CO_2. Duplicates were performed for each condition.

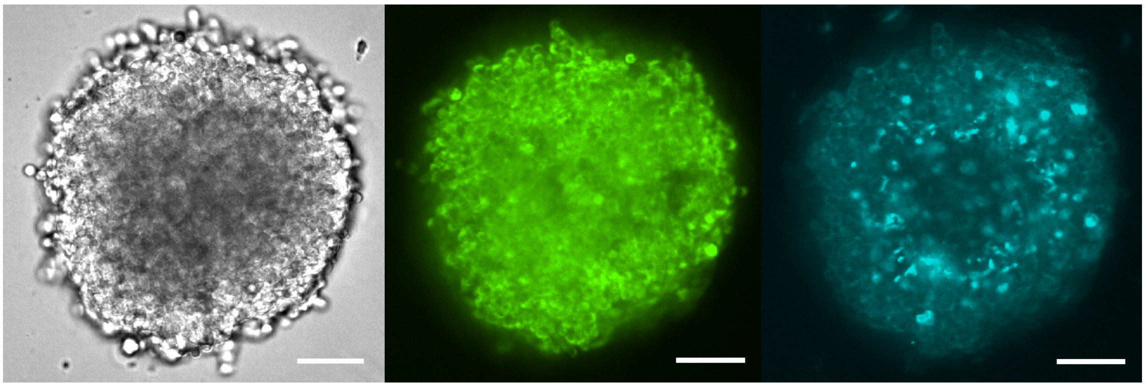

Fig. S6 TL channel (left), green channel recording the GFP signal in the mitochondria (middle) and light blue channel recording the signal from QDs **28** (right). Cross-sectional images were taken at 80 μm from the bottom. Scale bar: 100 μm.

4.1.3. Quantum dot analysis protocol

As in section 3.3.2., for each spheroid, the TL and green channel images were used for the segmentation of the object using a gaussian filter. To remove the bright fluorescent QDs aggregates, a "find blob" module was applied (Figure S7), to generate a modified spheroid body mask which was used to extract the average intensity values (average of the intensities of the pixels included in the mask) in the QDs channel image (Figure S7).

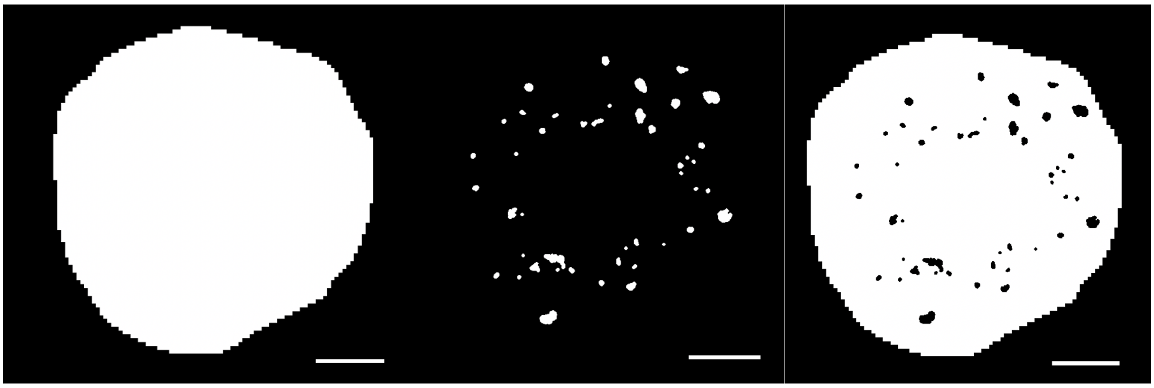

Fig. S7 Spheroid body mask (left), detection of bright aggregates with a "find blob" module (middle) and applied final spheroid body mask (right). Scale bar: 100 μm.

4.1.4 Quantum dot delivery quantification

Average fluorescent intensity values per spheroid (I) were calculated by subtracting average background intensity (control spheroid without addition of QDs) to the average intensity of the QDs-treated spheroids. Average intensities I_T for each condition were normalized against that obtained with spheroids treated with QD complex **27** (I_0) using Equation (S3):

$$I_T = I / I_0 \quad (S3)$$

Results from quantification are shown in Figure S8.

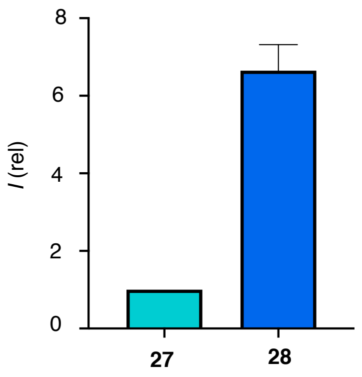

Fig. S8 Relative average intensities with standard deviation extracted from the QD channel, corresponding to the spheroids treated with **27** and **28**, respectively.

4.2. SupraFlipper delivery and release in spheroids

4.2.1. SupraFlipper streptavidin complex formation

To a freshly prepared solution of WT streptavidin **3** (200 µM in PBS, 1 mL), **4** (2 µL, 10 mM in DMSO, 2 equivalents) and SupraFlipper **29** (10 µL, 1 mM in DMSO, 1 equivalents) were added and the mixture was shaken for 10 min at rt. Aggregates were spun down for 1 min. Control complex **34** was prepared similarly, without the addition of **4** (Figure S9).

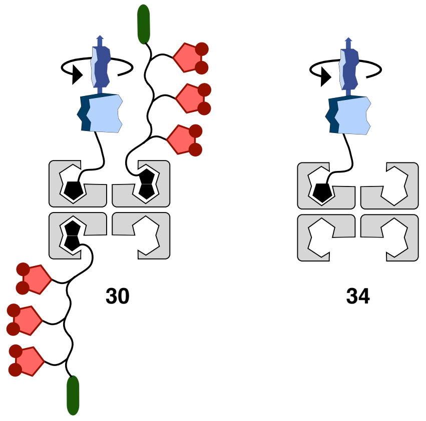

Fig. S9 Streptavidin complexes with SupraFlipper **30** and control without transporter **34**.

4.2.2. Delivery and release protocol

As in section 3.3.1., HGM cells were seeded at 5 × 10^3 cells/mL in FluoroBrite DMEM + 10% FBS on Nunclon™ Sphera™ U-bottom 96-well sterile µ-Plates and kept at 37 °C with 5% CO_2 for 3 days. After the spheroids were formed, the medium was exchanged to Leibovitz's (6 × 200 µL/well) using a Biotek MultiFlo™ FX plate washer, keeping a final volume of 135 µL/well. Then, complexes **30** and **34** in PBS were prepared and added to the µ-Plate containing the spheroids (15 µL/well, 10x final concentration in PBS) to reach a final volume of 150 µL/well (final concentration = 5 µM). Cells were incubated for 4 h at 37 °C with 5% CO_2. After this, spheroids were washed and imaged with a z-stack of 19 images/well (10 µm per step), using three channels: Transmitted light (brightfield), green (excitation filter: 475/34 nm, emission filter: 536/40 nm, exposure time: 200 ms) and GFP-TxRed (excitation filter: 475/34 nm, emission filter: 641/75 nm, exposure time: 200 ms). Then, biotin **31** was added (15 µL/well, 1 mM in PBS) to reach a final volume of 150 µL/well (100 µM of biotin), except for the control wells, where only PBS was added (15 µL/well). After 2 h of incubation at 37 °C with 5% CO_2, the plate was washed again. Then, the plate was imaged using the same previous conditions. During imaging, samples were kept at 37 °C with 5% CO_2. Duplicates were performed for each condition.

4.2.3. SupraFlipper delivery and release analysis protocol

As in section 3.3.2., for each spheroid, the TL and green channel images were used for the segmentation of the object using a gaussian filter. In addition, several filters were added to generate a layer quantification. Briefly, the spheroid body mask was shrunk to create layers starting from center to the extremities (Figure S10), to generate a modified spheroid body mask. Layer 1 in white (Figure S10) was used to extract the average intensity values (average of the

intensities of the pixels included in the layer) in the GFP-TxRed channel image to report on maximal penetration of the complex (Figure S10).

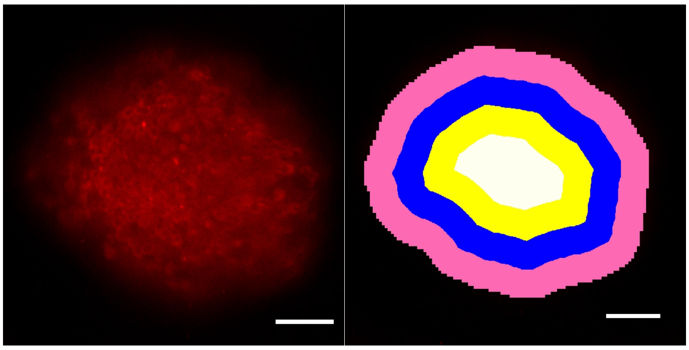

Fig. S10 Red channel recording the SupraFipper signal in the spheroid after addition of biotin for 2 h (left), Layer analysis applied to the red channel with layer 1 (white), layer 2 (yellow), layer 3 (blue) and layer 4 (pink) masks (right). Cross-sectional CSDM images were taken at 80 µm from the bottom. Scale bar: 100 µm.

4.2.4. SupraFlipper delivery and release quantification

Delivery and release were performed as in section 4.1.2. The average intensity values for each condition were obtained using analysis protocol in section 4.1.3.

The resulting average fluorescent intensity values per spheroid (I) were normalized compared to the intensity of the spheroids treated with complex **34** before addition of biotin (I_0), using Equation (S4), to give I_T:

$$I_T = I / I_0 \quad (S4)$$

Results from the experiments are reported in Figure S11.

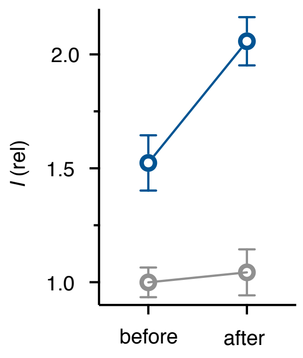

Fig. S11 Relative flipper intensity with standard deviation measured in the core of the spheroid treated with complex **30** (blue cirles) and control complex **34** (grey circles) for 4 h, before and after 2 h incubation with 100 μM of biotin **31**. Experiments were performed in duplicates.

4.2.5. FLIM in spheroids

HGM spheroids were prepared, incubated and washed as in section 4.2.2. On the day of the experiment, after the final washing, the labelled spheroid was carefully transferred by pipette aspiration to a 35 mm dish containing fresh Leibovitz's medium (2.7 mL). Images of the spheroid were acquired in two channels, recording GFP (λ_{ex} = 488 nm; λ_{em} = 497 – 515 nm) and SupraFlipper signal (λ_{ex} = 488 nm; λ_{em} = 619 – 800 nm). Afterward, biotin **31** (0.3 mL, 10 mM) was added to the dish, reaching a final concentration of 1 mM. Several pictures of the same plane of the spheroid and in the two channels were recorded over time, until no further increase of intensity of the flipper was observed (approximately 40 min after the addition). Finally, a solution of NaCl (5 M, 1.1 mL) was added, to reach a final concentration of NaCl of 1 M. After 10 min, images in both channels were again acquired.

5. References

S1 R. Martinent, D. Du, J. López-Andarias, N. Sakai and S. Matile, *ChemBioChem*, 2020, **22**, 253–259.

S2 D. Liße, V. Wilkens, C. You, K. Busch and J. Piehler, *Angew. Chem. Int. Ed.*, 2011, **50**, 9352–9355.

S3 J. López-Andarias, K. Straková, R. Martinent, N. Jiménez-Rojo, H. Riezman, N. Sakai and S. Matile, *JACS Au*, 2021, **1**, 221–232.

S4 L. Peraro, K. L. Deprey, M. K. Moser, Z. Zou, H. L. Ball, B. Levine and J. A. Kritzer, *J. Am. Chem. Soc.*, 2018, **140**, 11360–11369.

S5 A. Doerflinger, N. N. Quang, E. Gravel, G. Pinna, M. Vandamme, F. Ducongé and E. Doris, *Chem. Commun.*, 2018, **54**, 3613–3616.

S6 a) R. Singh and G. M. Whitesides, *J. Am. Chem. Soc.*, 1990, **112**, 1190-1197; b) J. P. Danehy and V. Elia, *J. Org. Chem.*, 1972, **37**, 369-373; c) L. Schotte and H. Ström, *Acta Chem. Scand.*, 1956, **10**, 687-688; d) F. M. Unger and E. Liehl, *GB Patent*, 1985, GB2,148,296A; e) N. Sakai, M. Lista, O. Kel, S. Sakurai, D. Emery, J. Mareda, E. Vauthey and S. Matile, *J. Am. Chem. Soc.*, 2011, **133**, 15224-15227.

S7 V. Haridas, S. Sadanandan, P.-Y. Collart-Dutilleul, S. Gronthos and N. H. Voelcker, *Biomacromolecules*, 2014, **15**, 582–590.

S8 D. Abegg, G. Gasparini, D. G. Hoch, A. Shuster, E. Bartolami, S. Matile and A. Adibekian, *J. Am. Chem. Soc.*, 2017, **139**, 231–238.

S9 B. Peng, A.-G. Thorsell, T. Karlberg, H. Schüler and S. Q. Yao, *Angew. Chem. Int. Ed.*, 2017, **56**, 248–253.

S10 E. R. Ballister, C. Aonbangkhen, A. M. Mayo, M. A. Lampson and D. M. Chenoweth, *Nat. Commun.,* 2014, **5**, 5475.

S11 R. Martinent, J. López-Andarias, D. Moreau, Y. Cheng, N. Sakai and S. Matile, *Beilsein J. Org. Chem.*, 2020, **16**, 2007–2016.

S12 E. Bartolami, D. Basagiannis, L. Zong, R. Martinent, Y. Okamoto, Q. Laurent, T. R. Ward, M. Gonzalez-Gaitan, N. Sakai and S. Matile, *Chem. Eur. J.*, 2019, **25**, 4047–4051.

The original data can be found at: https://doi.org/10.5281/zenodo.5515808

6. NMR and HPLC-MS spectra

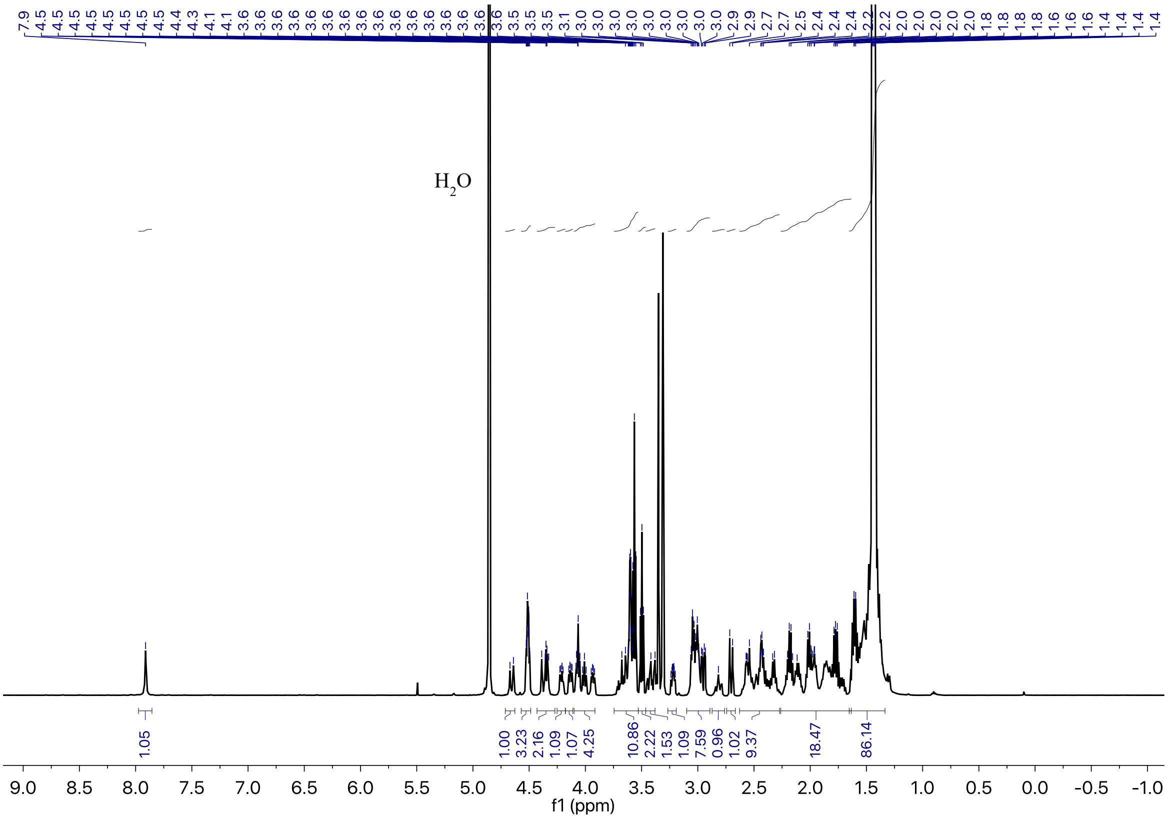

Fig. S12 ^{1}H NMR spectrum (500 MHz) of **51** in CD_3OD.

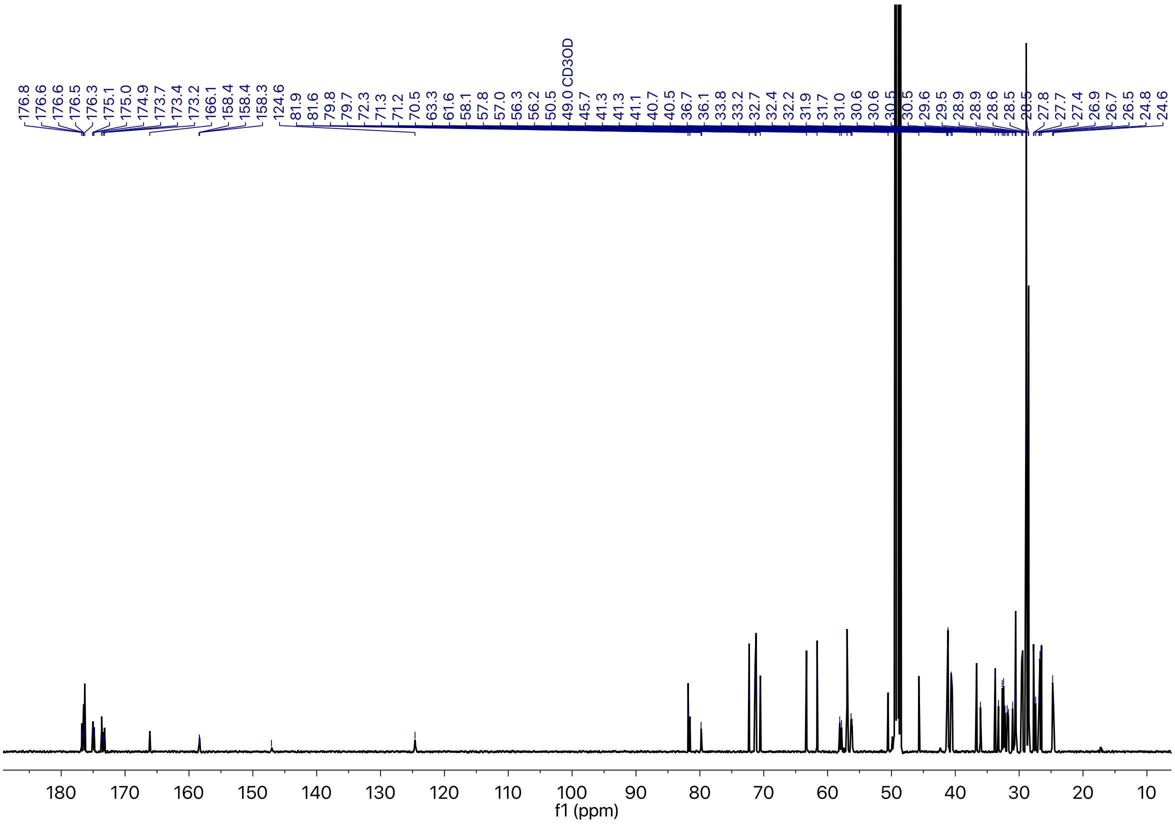

Fig. S13 ^{13}C NMR spectrum (126 MHz) of **51** in CD_3OD.

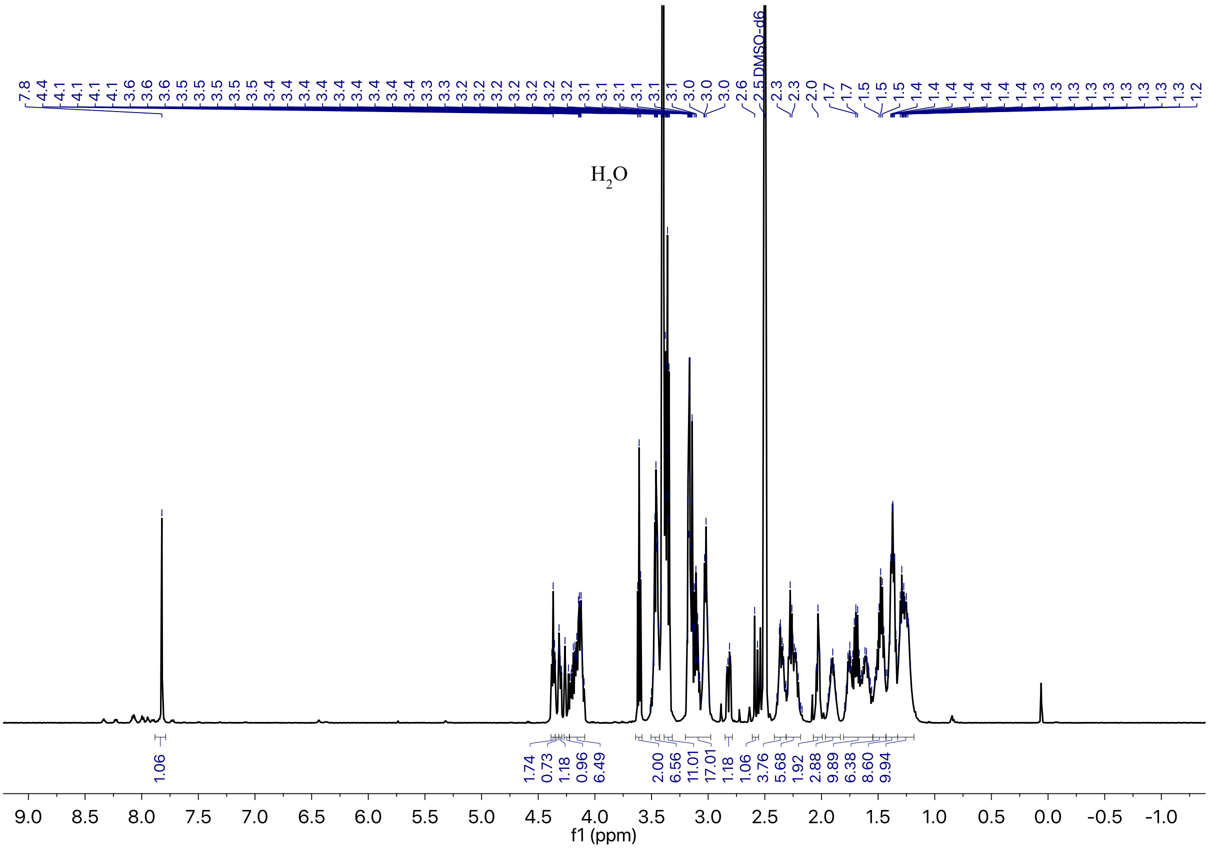

Fig. S14 ^{1}H NMR spectrum (500 MHz) of **5** in DMSO-d_6/D_2O 60/1.

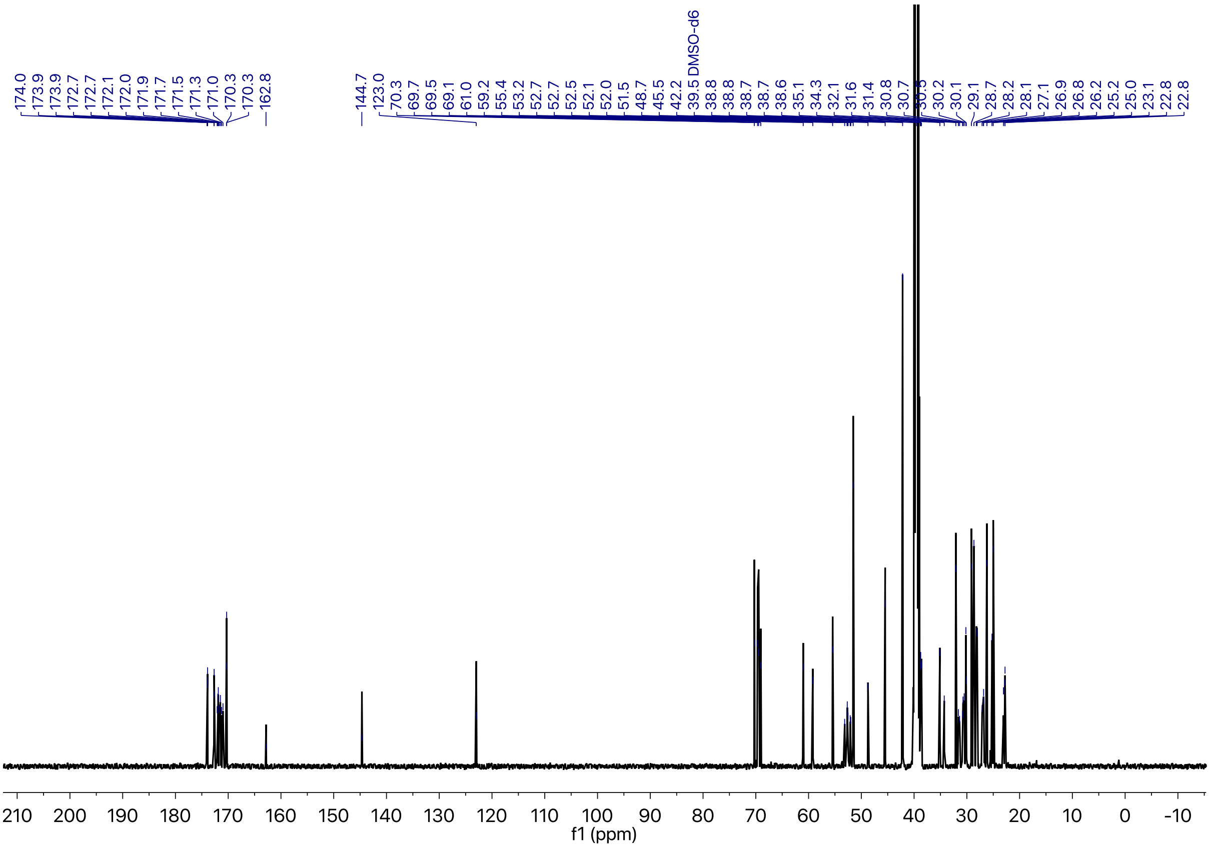

Fig. S15 ^{13}C NMR spectrum (126 MHz) of **5** in DMSO-d_6.

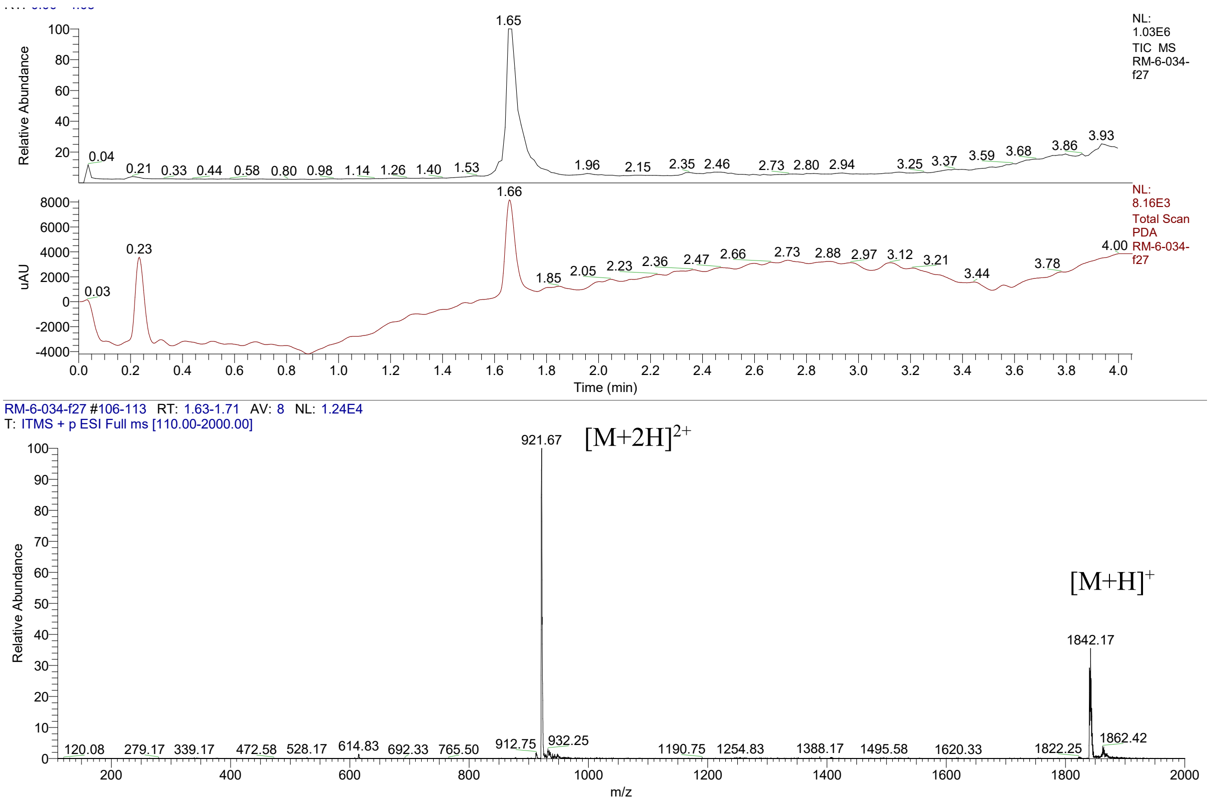

Fig. S16 HPLC-MS profile of **5**.

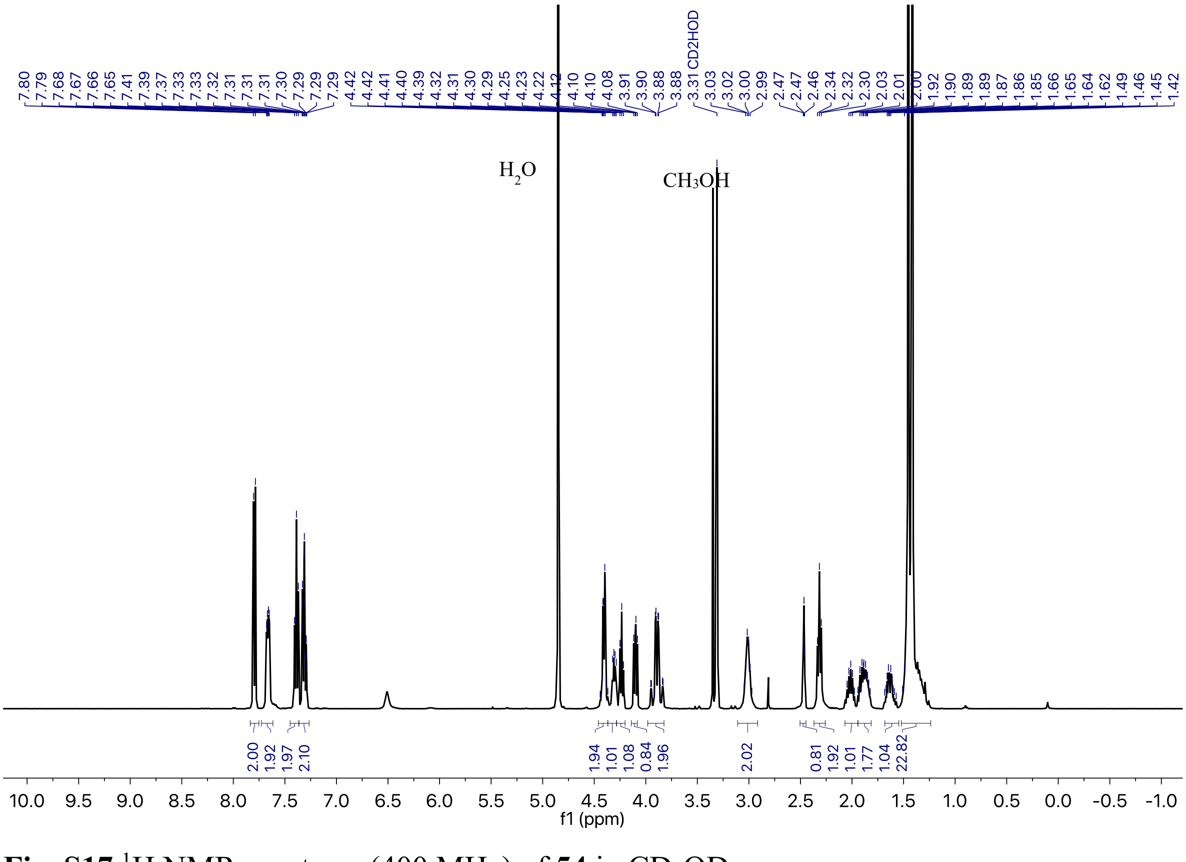

Fig. S17 ^{1}H NMR spectrum (400 MHz) of **54** in CD_3OD.

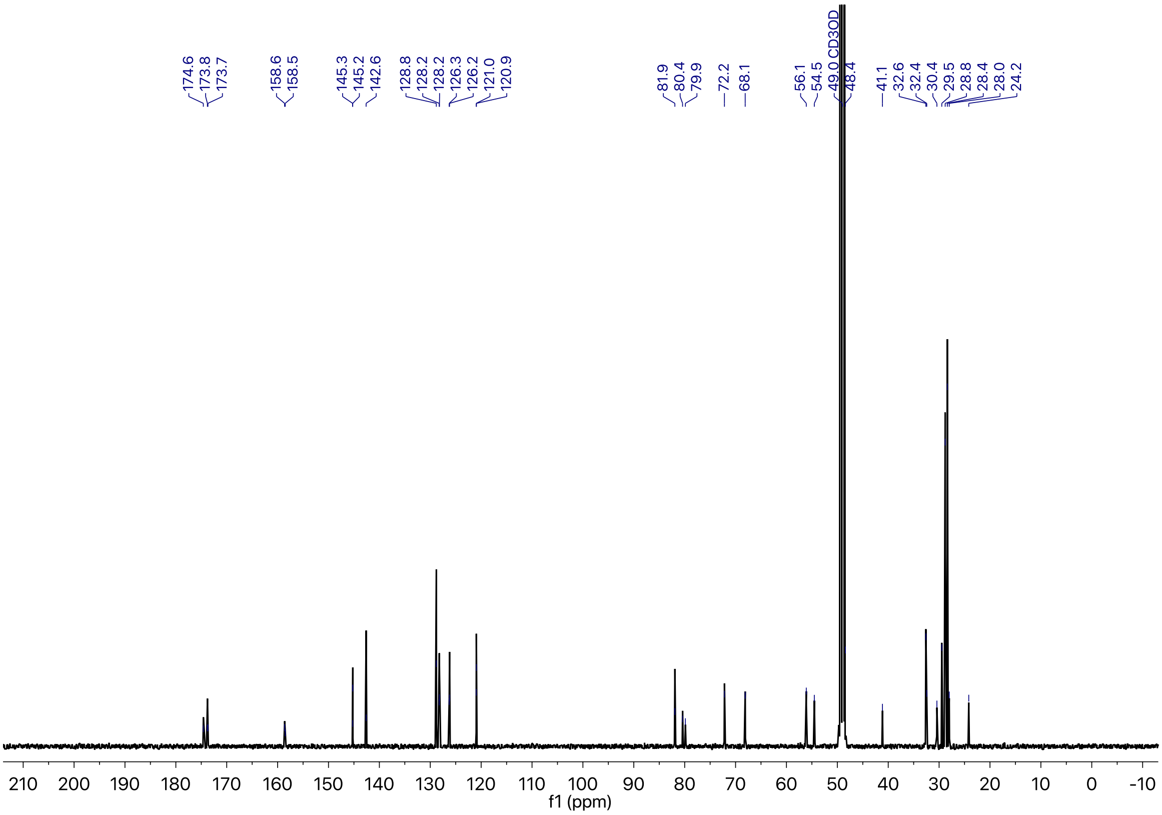

Fig. S18 ^{13}C NMR spectrum (101 MHz) of **54** in CD_3OD.

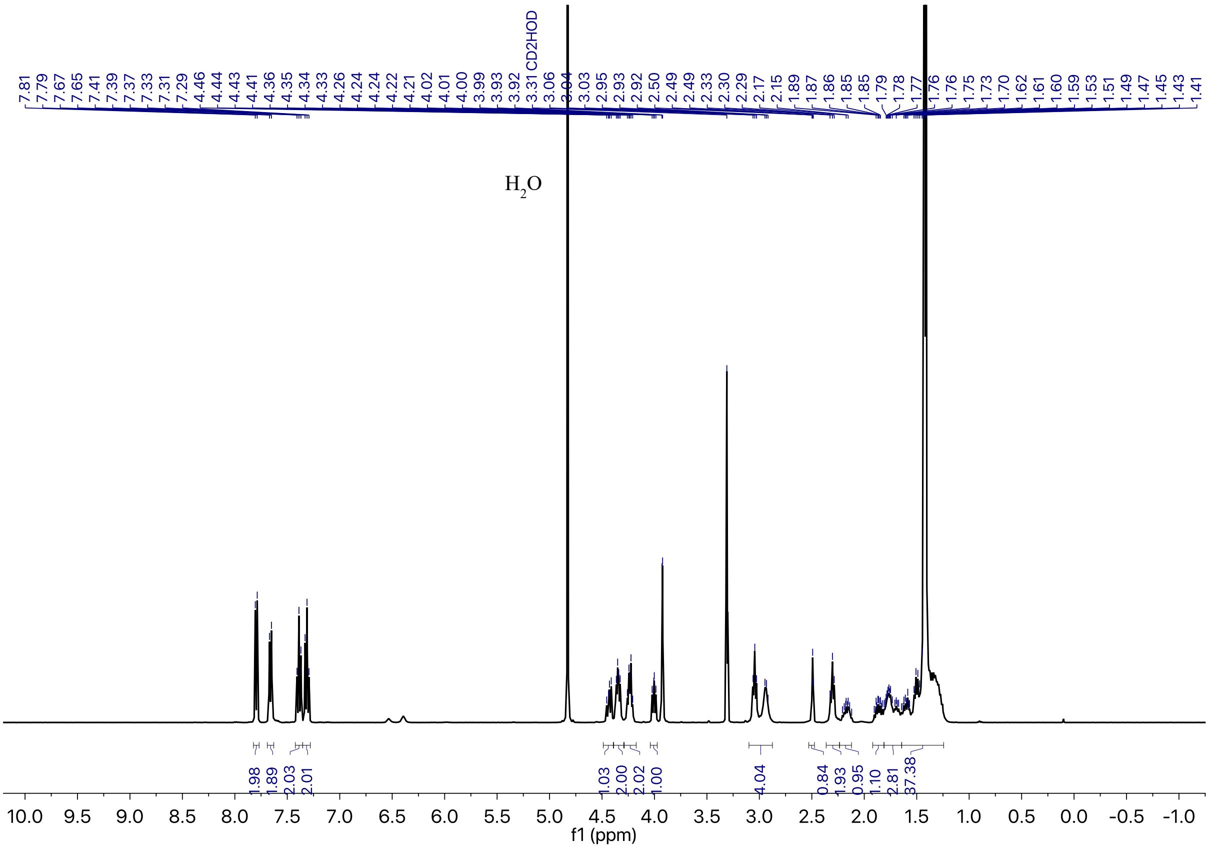

Fig. S19 ^{1}H NMR spectrum (400 MHz) of **56** in CD_3OD.

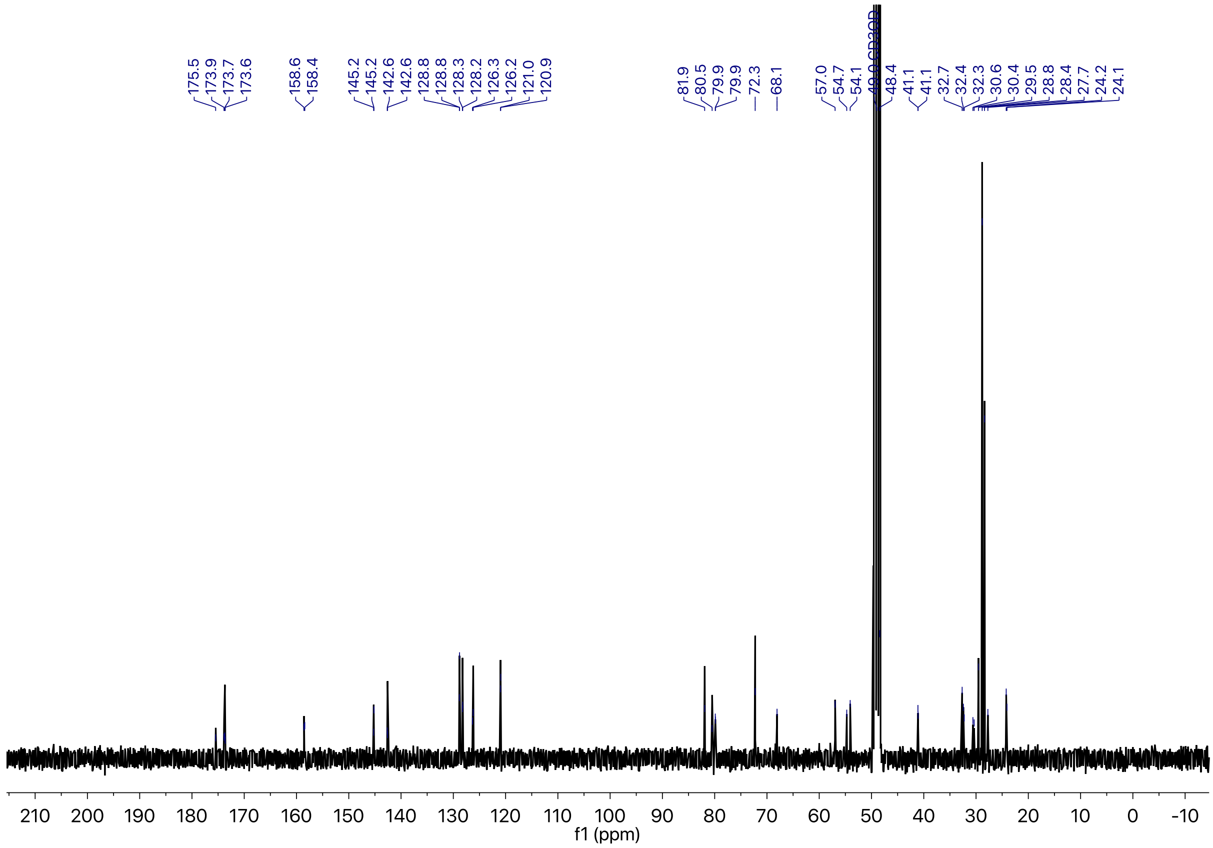

Fig. S20 ^{13}C NMR spectrum (101 MHz) of **56** in CD_3OD.

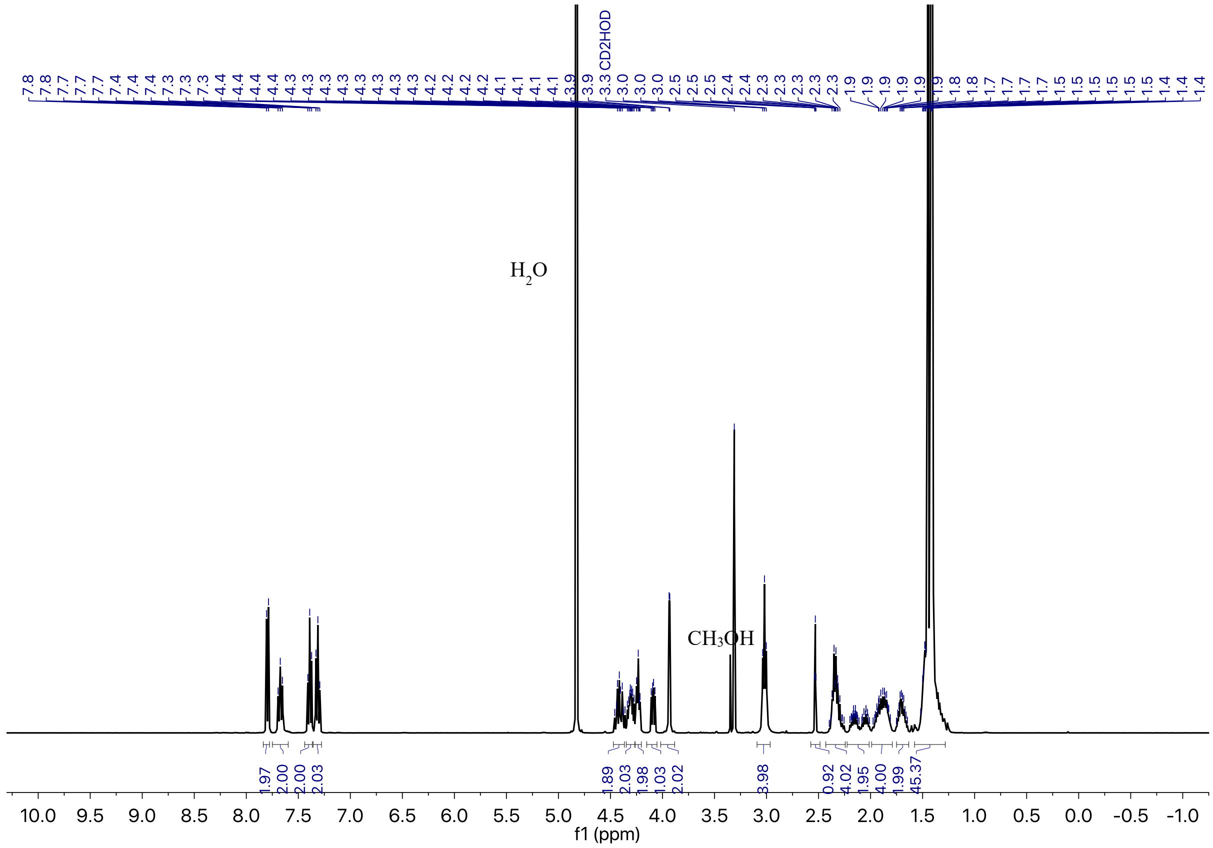

Fig. S21 ^{1}H NMR spectrum (400 MHz) of **58** in CD_3OD.

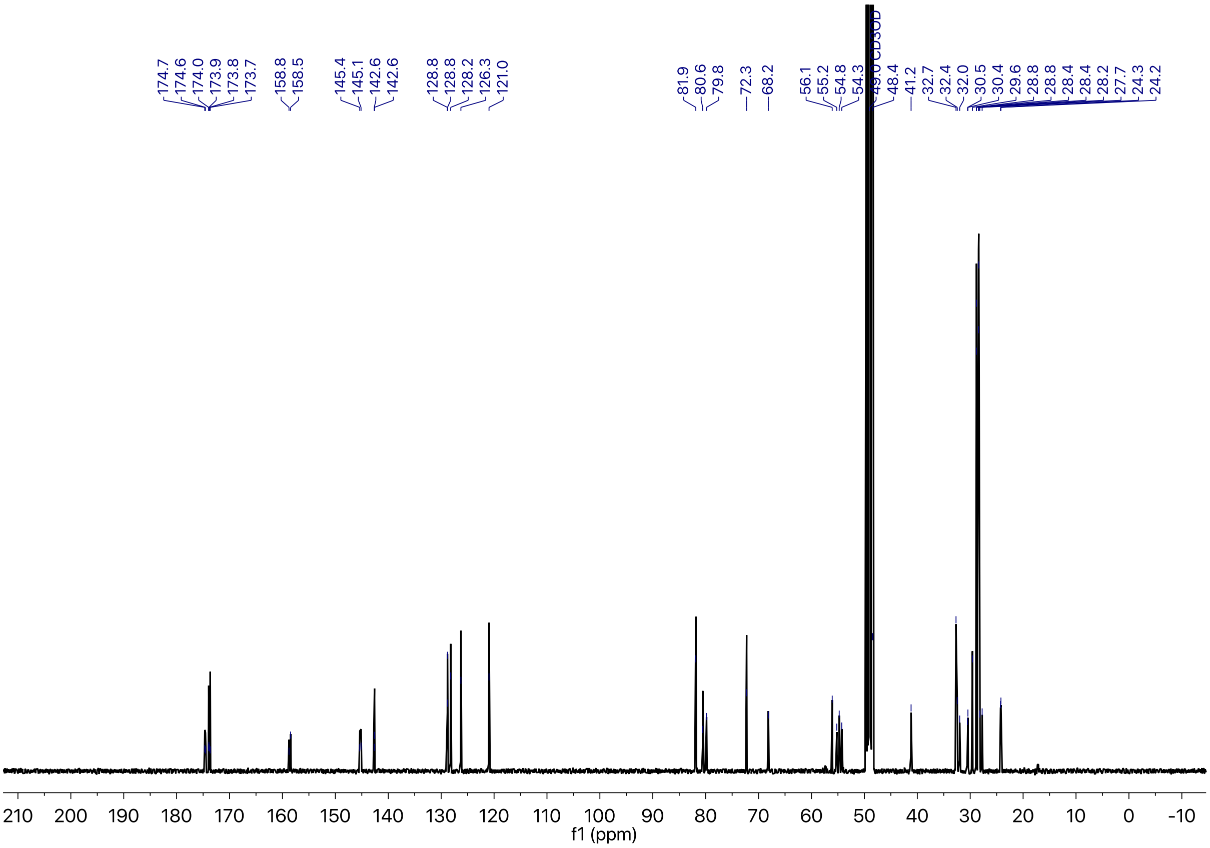

Fig. S22 ^{13}C NMR spectrum (101 MHz) of **58** in CD_3OD.

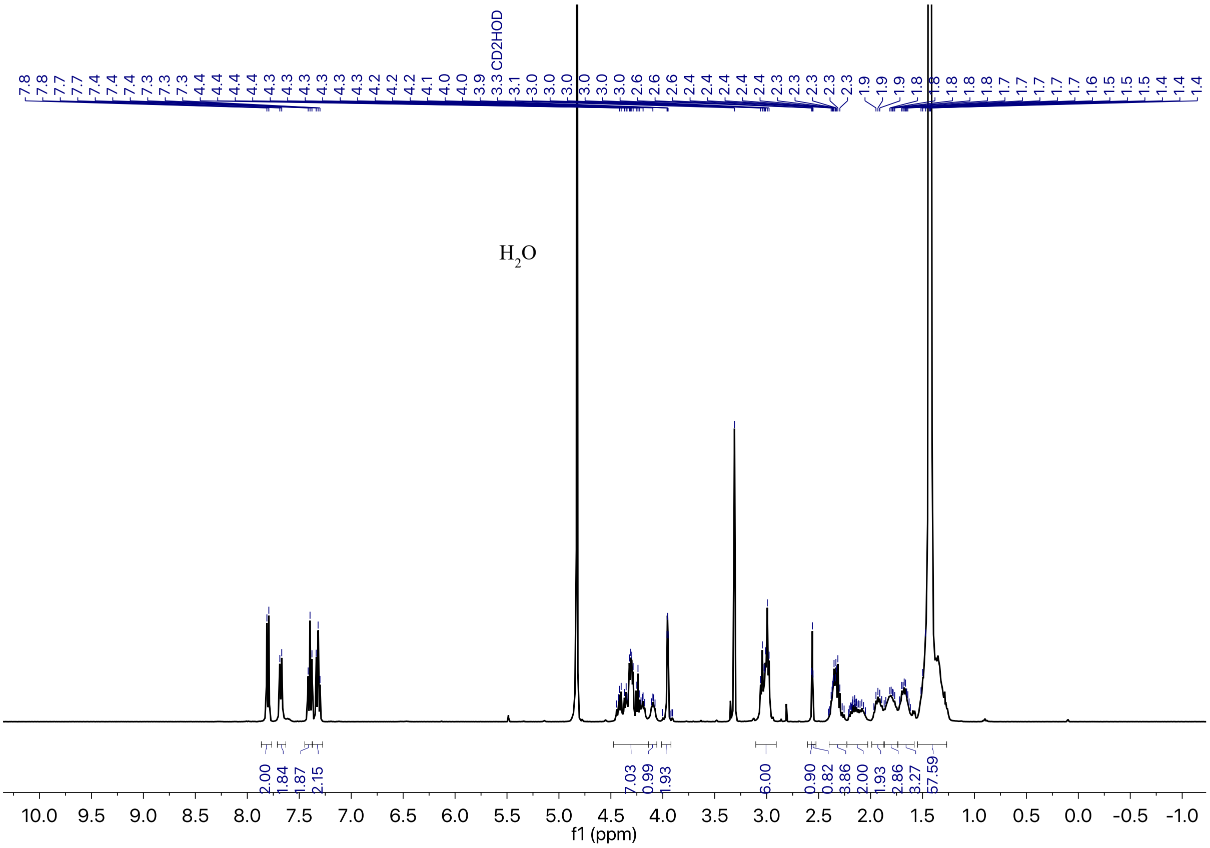

Fig. S23 ^{1}H NMR spectrum (400 MHz) of **60** in CD_3OD.

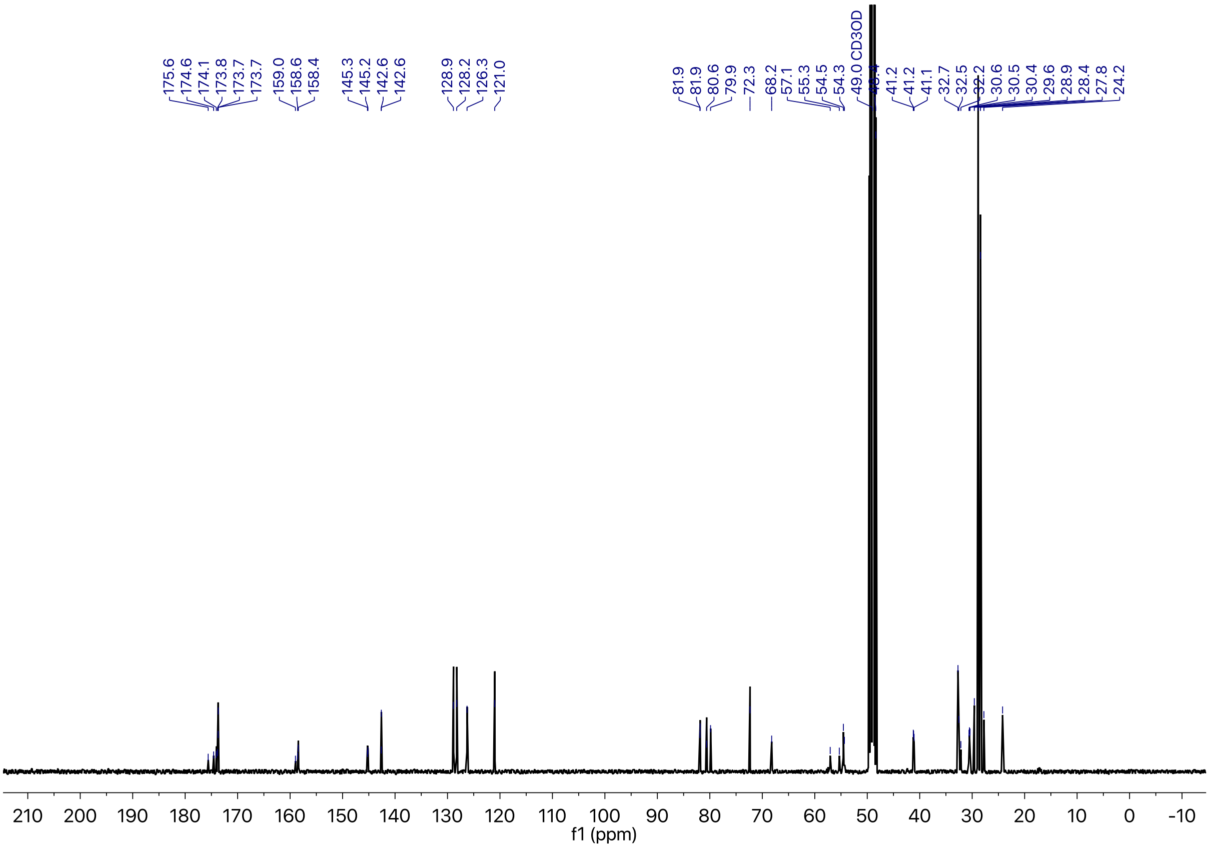

Fig. S24 ^{13}C NMR spectrum (101 MHz) of **60** in CD_3OD.

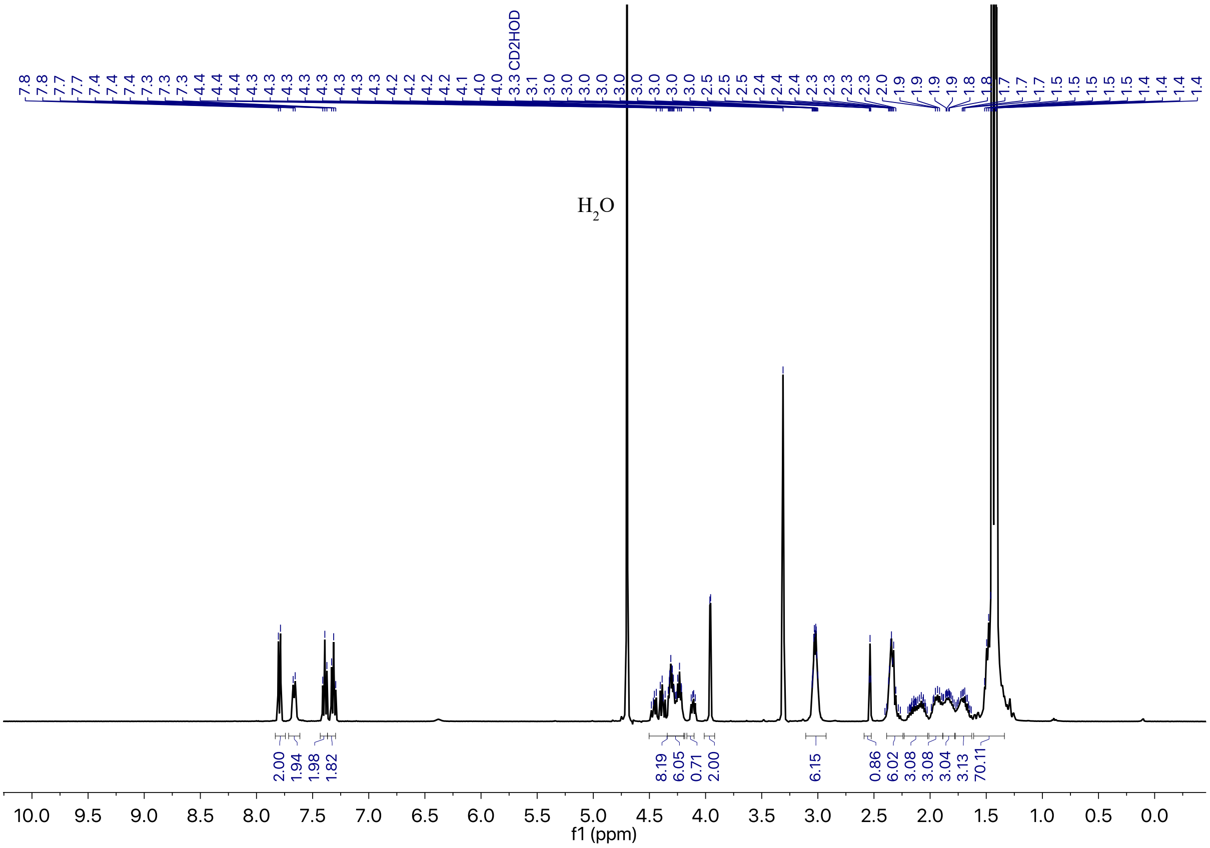

Fig. S25 ^{1}H NMR spectrum (400 MHz) of **62** in CD_3OD.

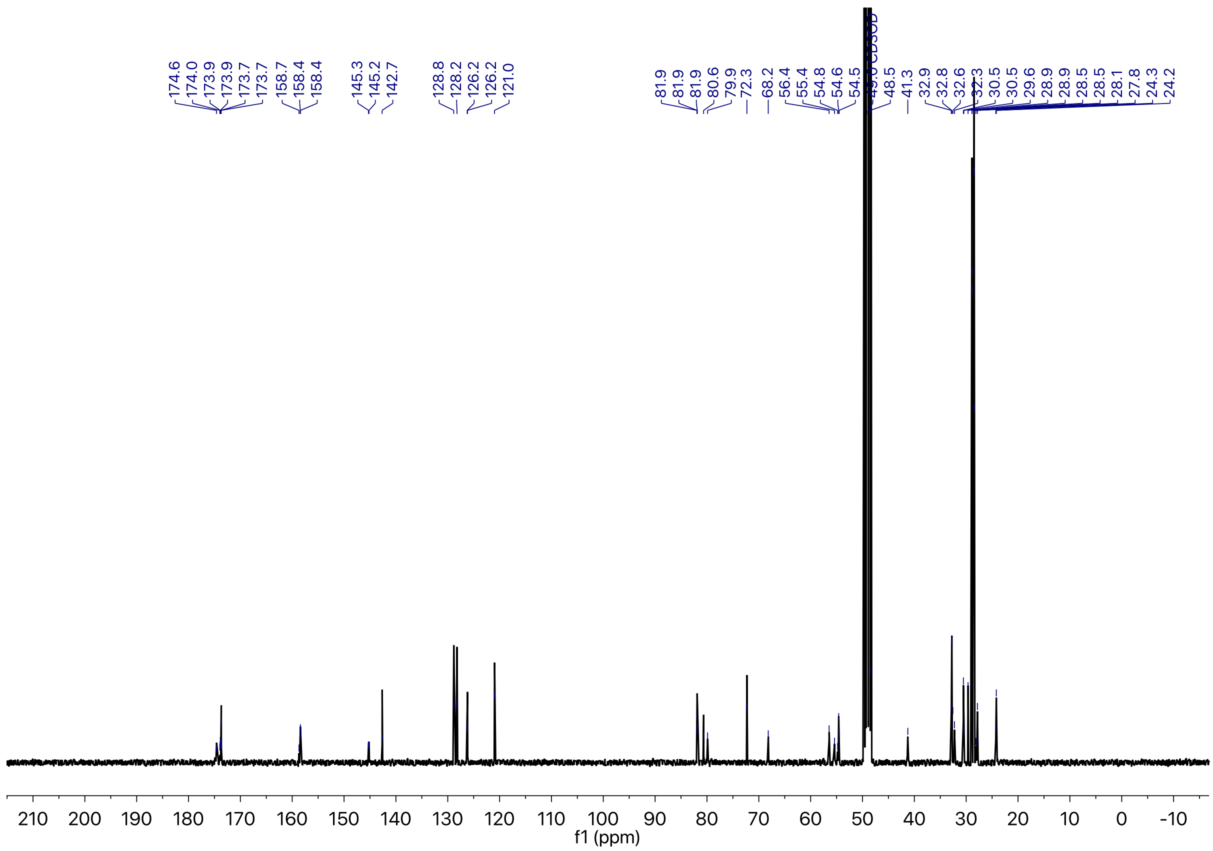

Fig. S26 ^{13}C NMR spectrum (101 MHz) of **62** in CD_3OD.

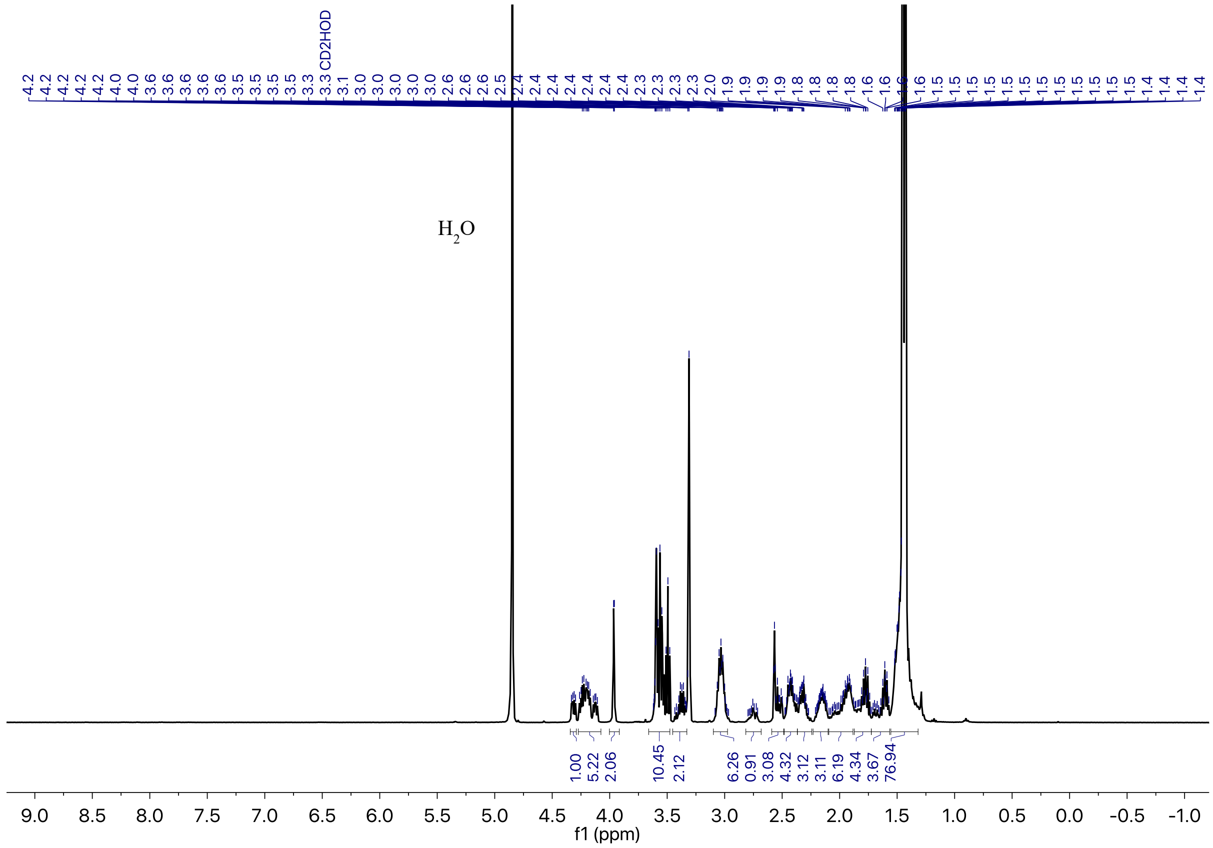

Fig. S27 ^{1}H NMR spectrum (400 MHz) of **64** in CD_3OD.

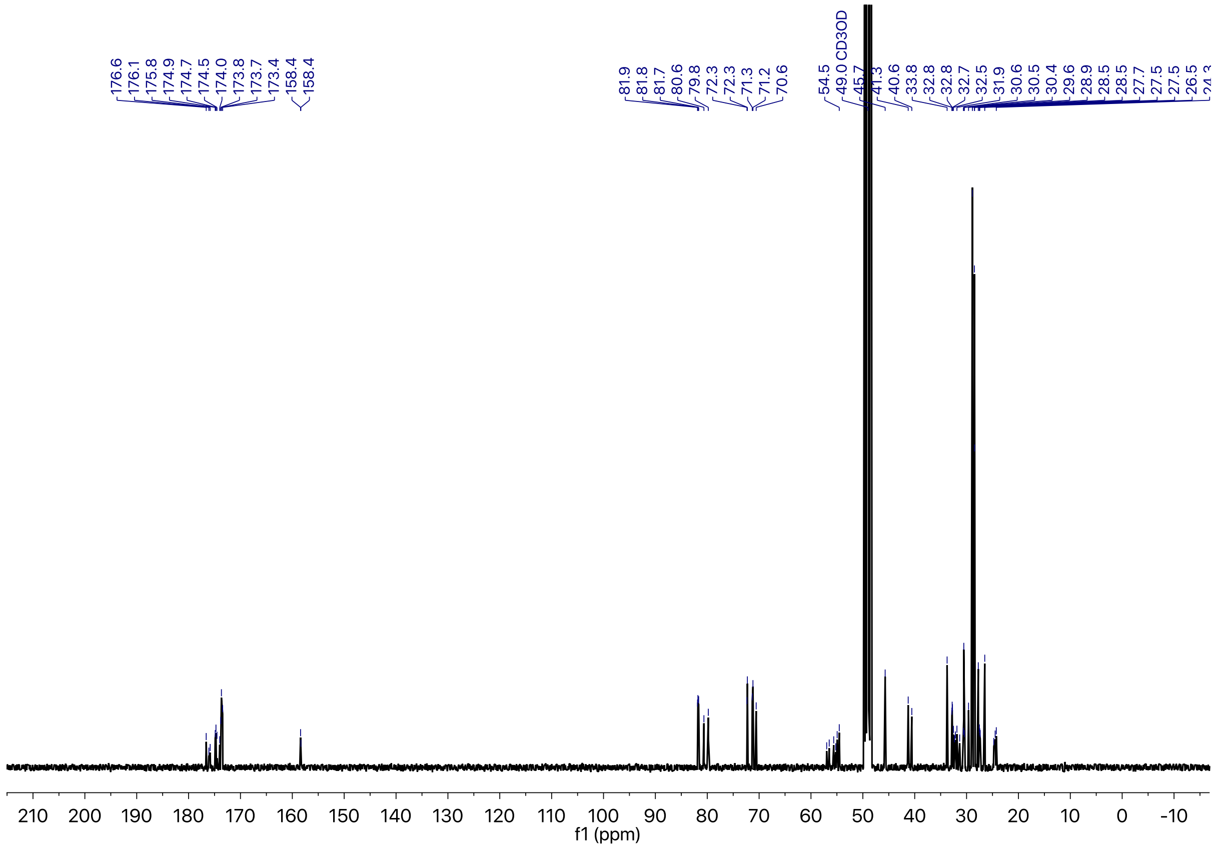

Fig. S28 ^{13}C NMR spectrum (101 MHz) of **64** in CD_3OD.

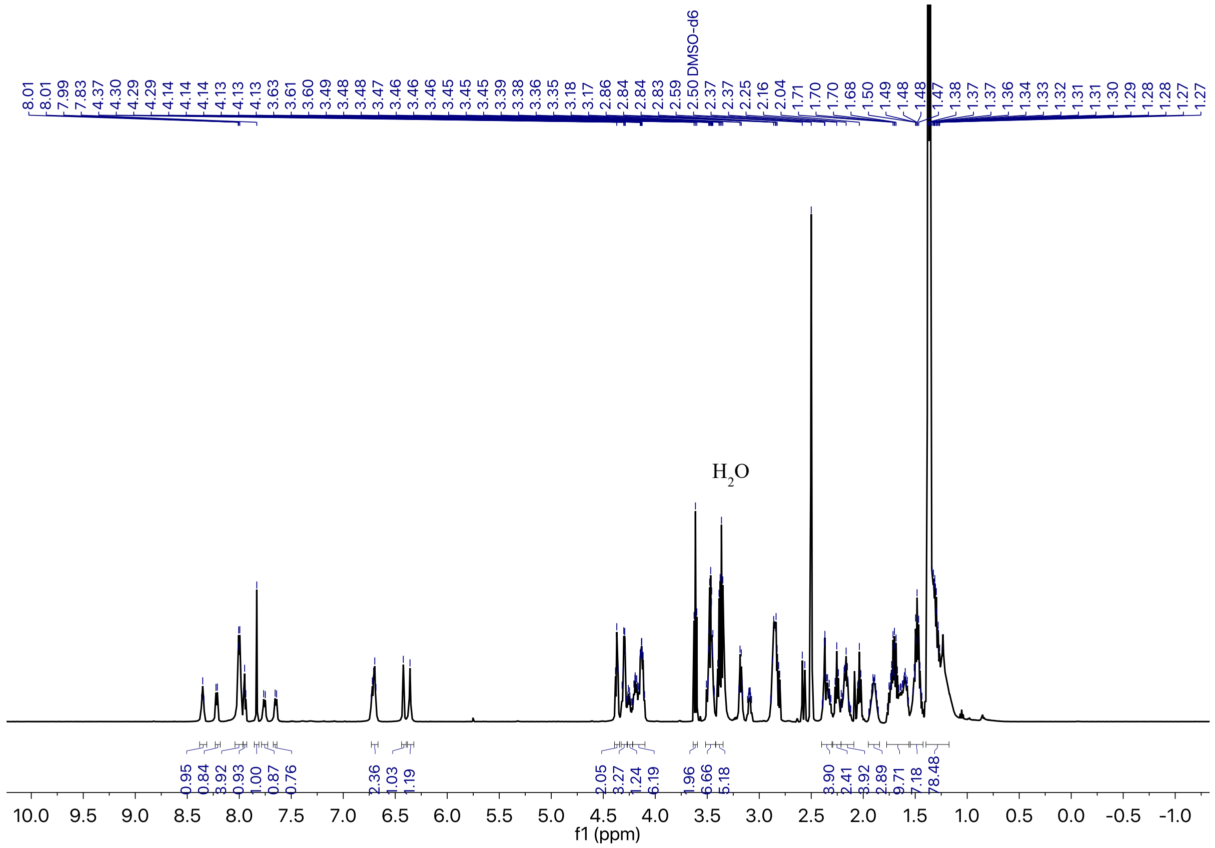

Fig. S29 ^{1}H NMR spectrum (500 MHz) of **65** in DMSO-d_6.

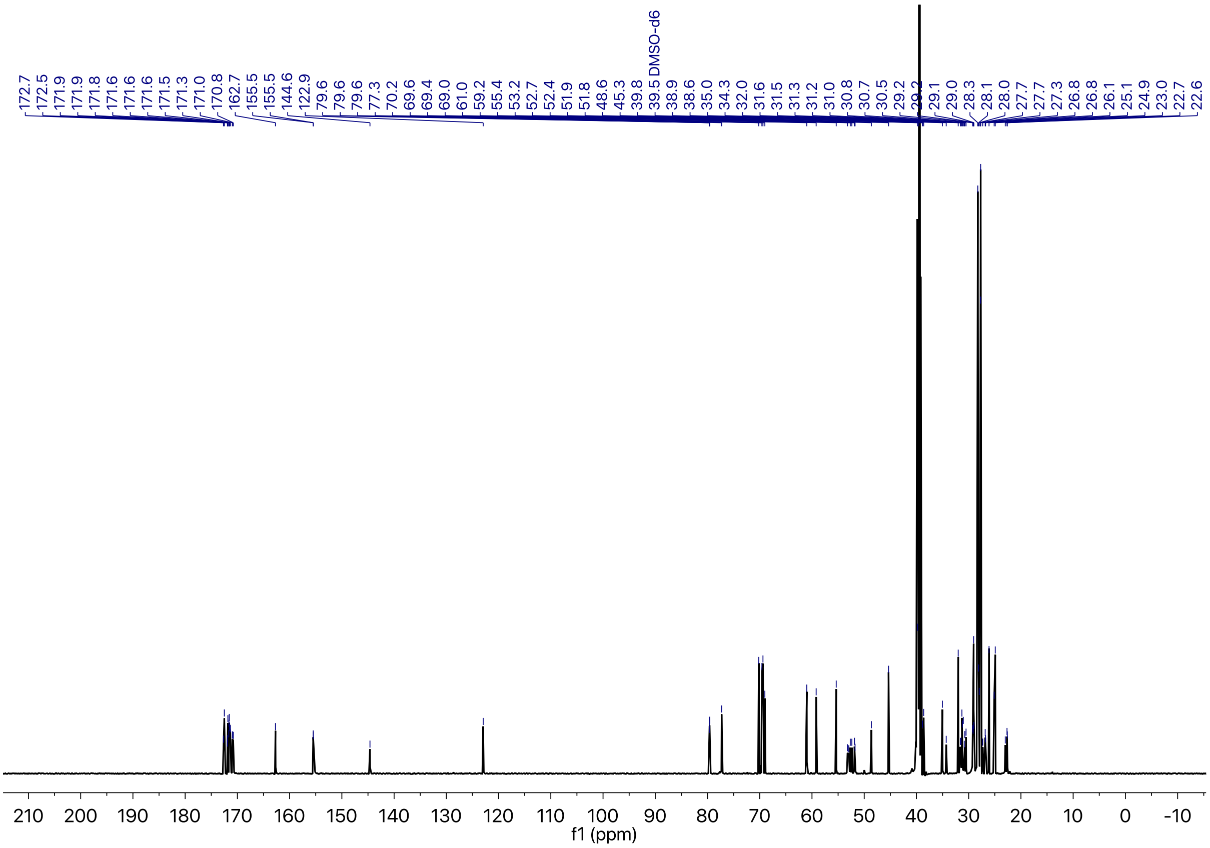

Fig. S30 ^{13}C NMR spectrum (126 MHz) of **65** in DMSO-d_6.

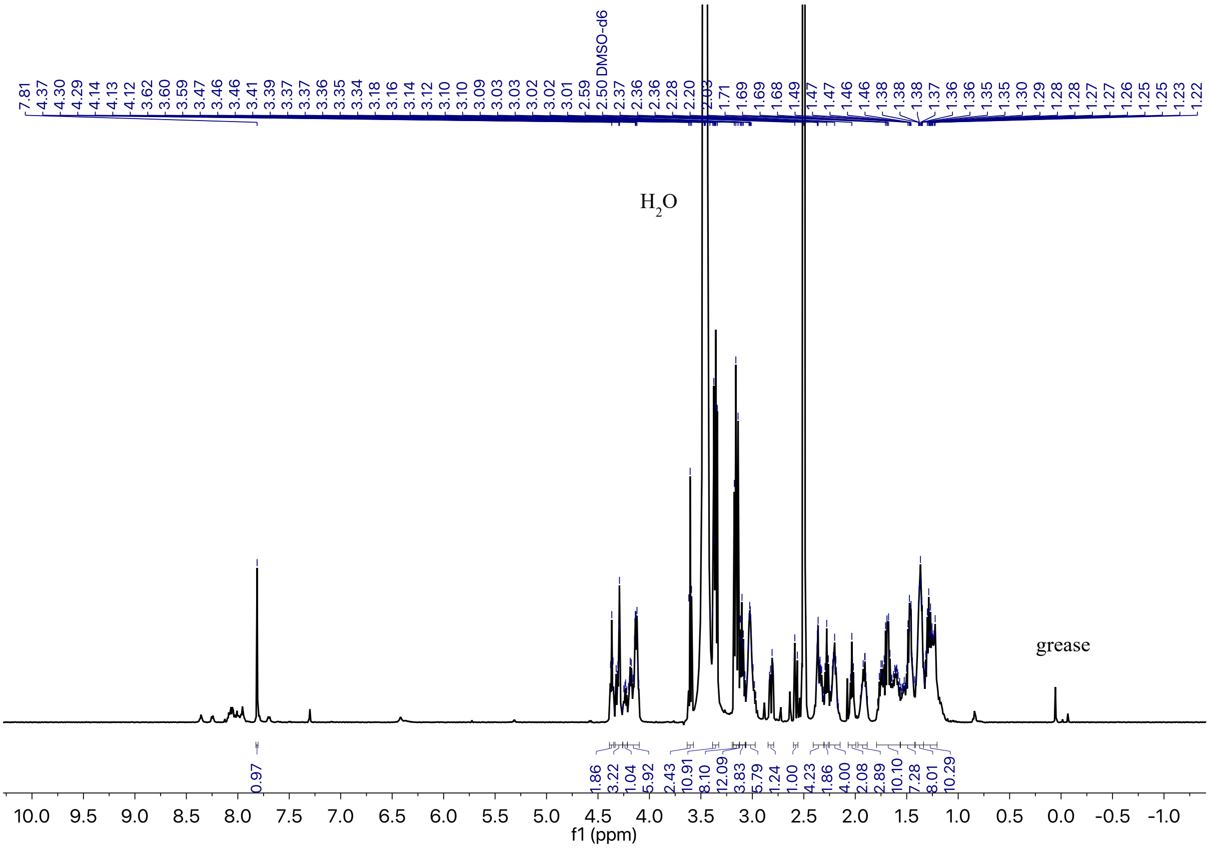

Fig. S31 ^{1}H NMR spectrum (500 MHz) of **6** in DMSO-d_6/D_2O 60/1.

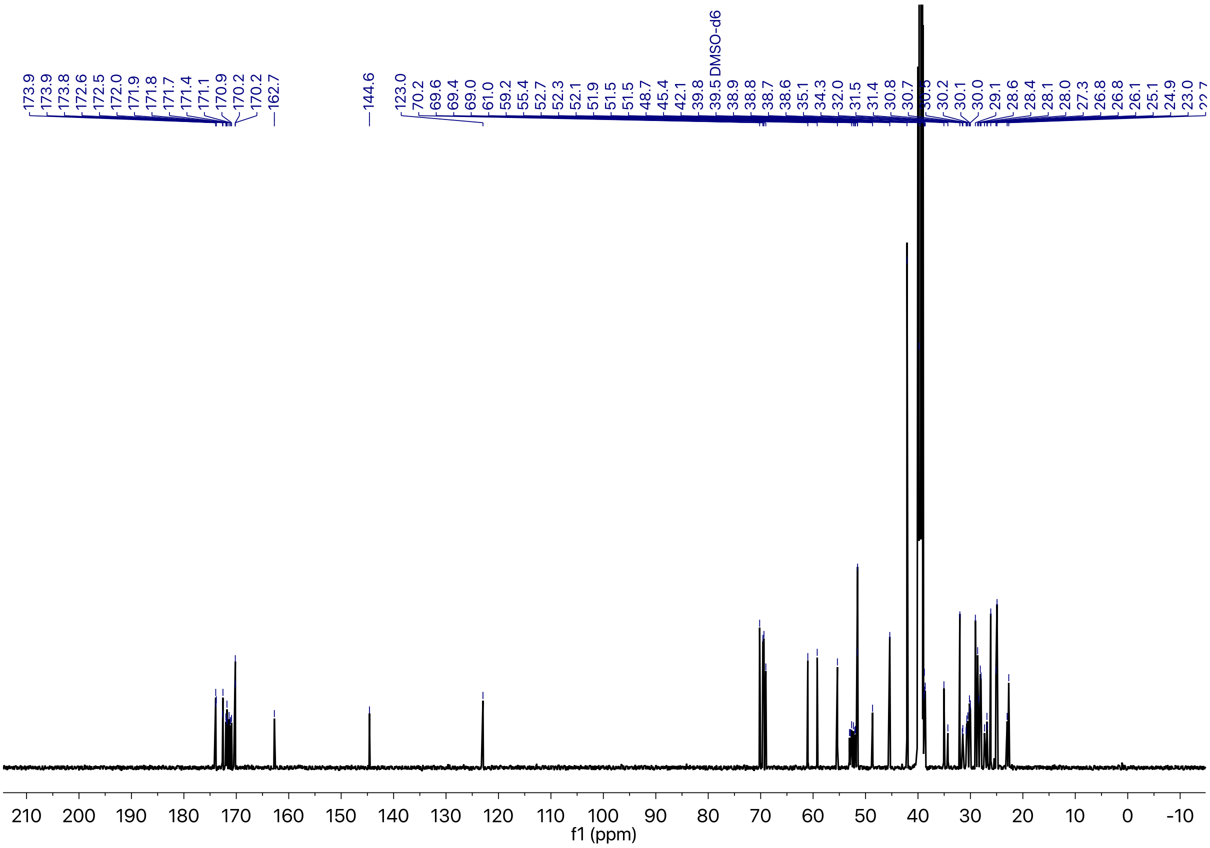

Fig. S32 ^{13}C NMR spectrum (126 MHz) of **6** in DMSO-d_6.

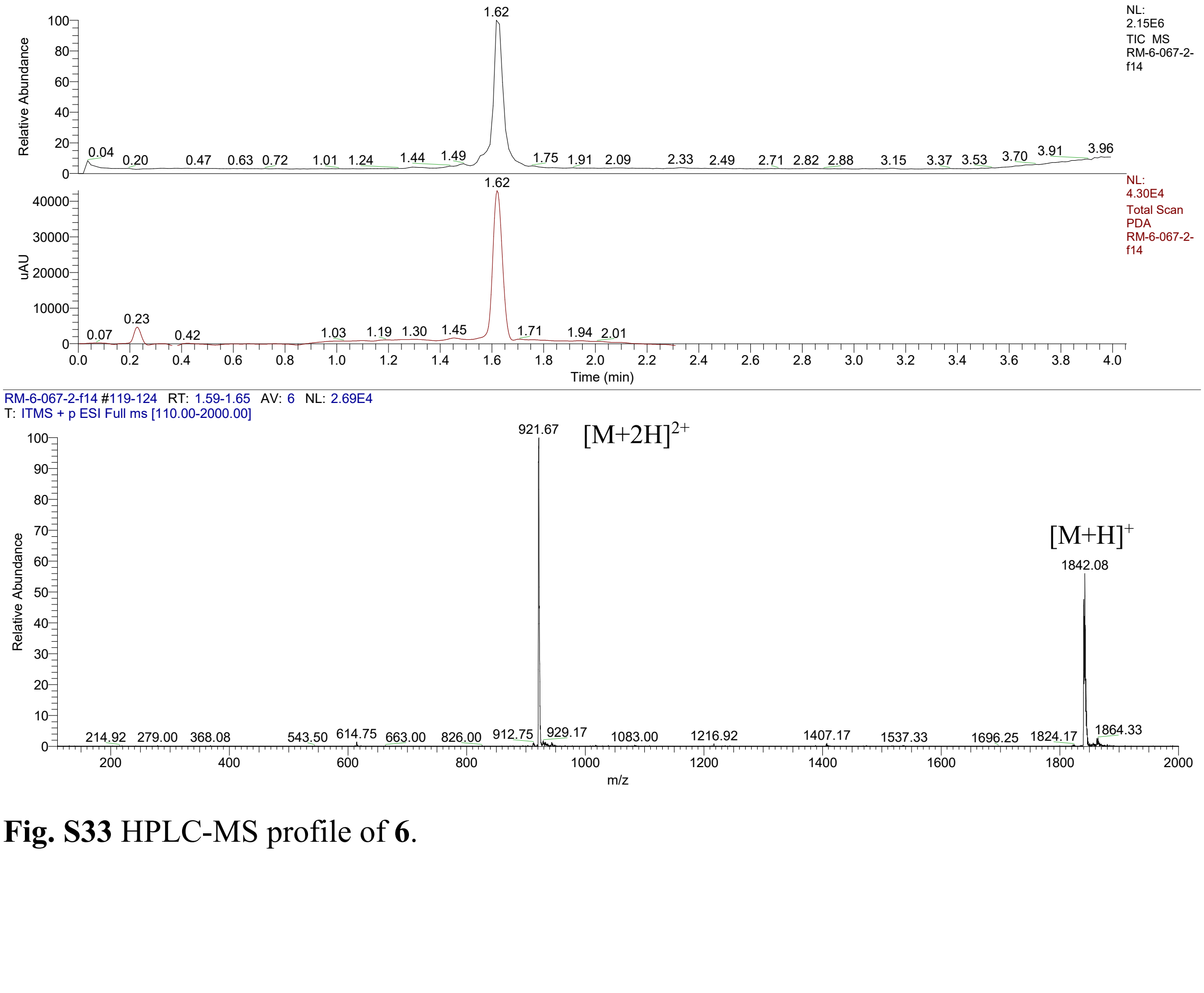

Fig. S33 HPLC-MS profile of **6**.

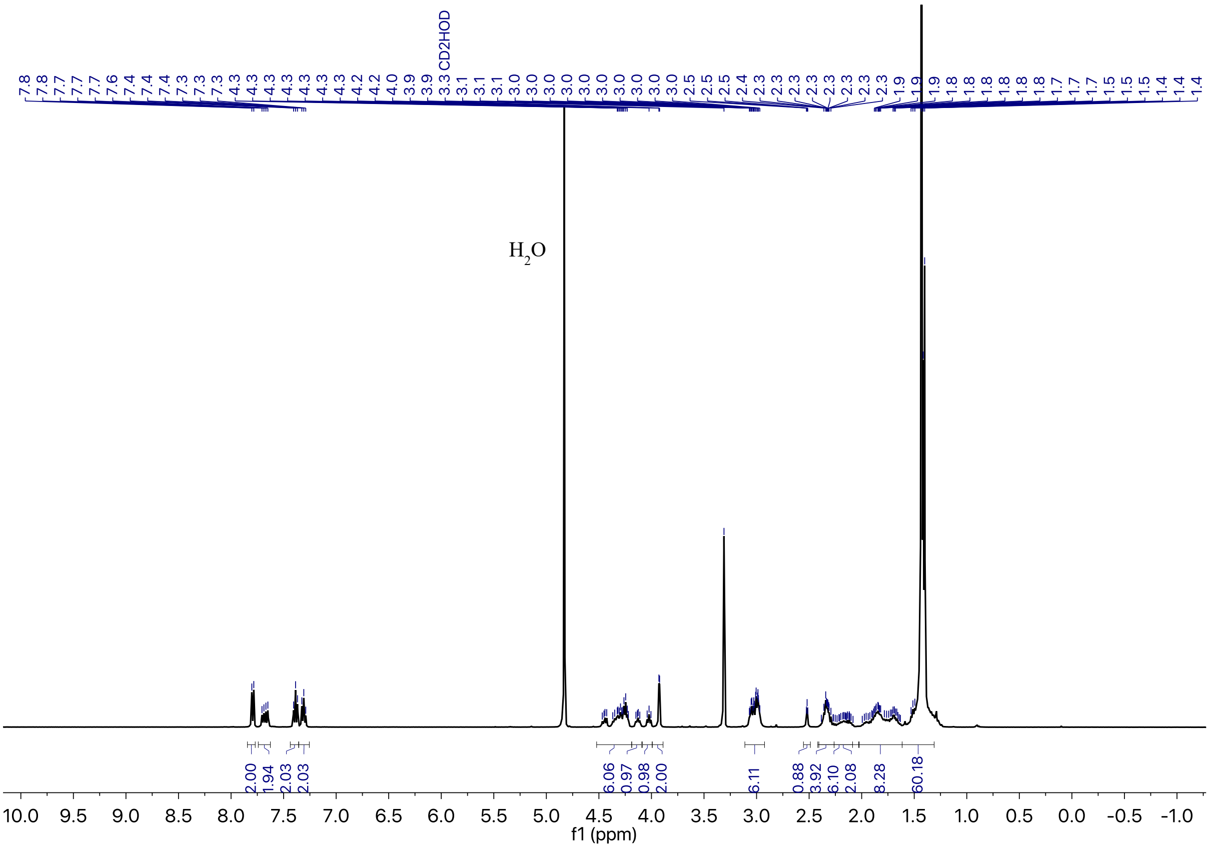

Fig. S34 ^{1}H NMR spectrum (400 MHz) of **67** in CD_3OD.

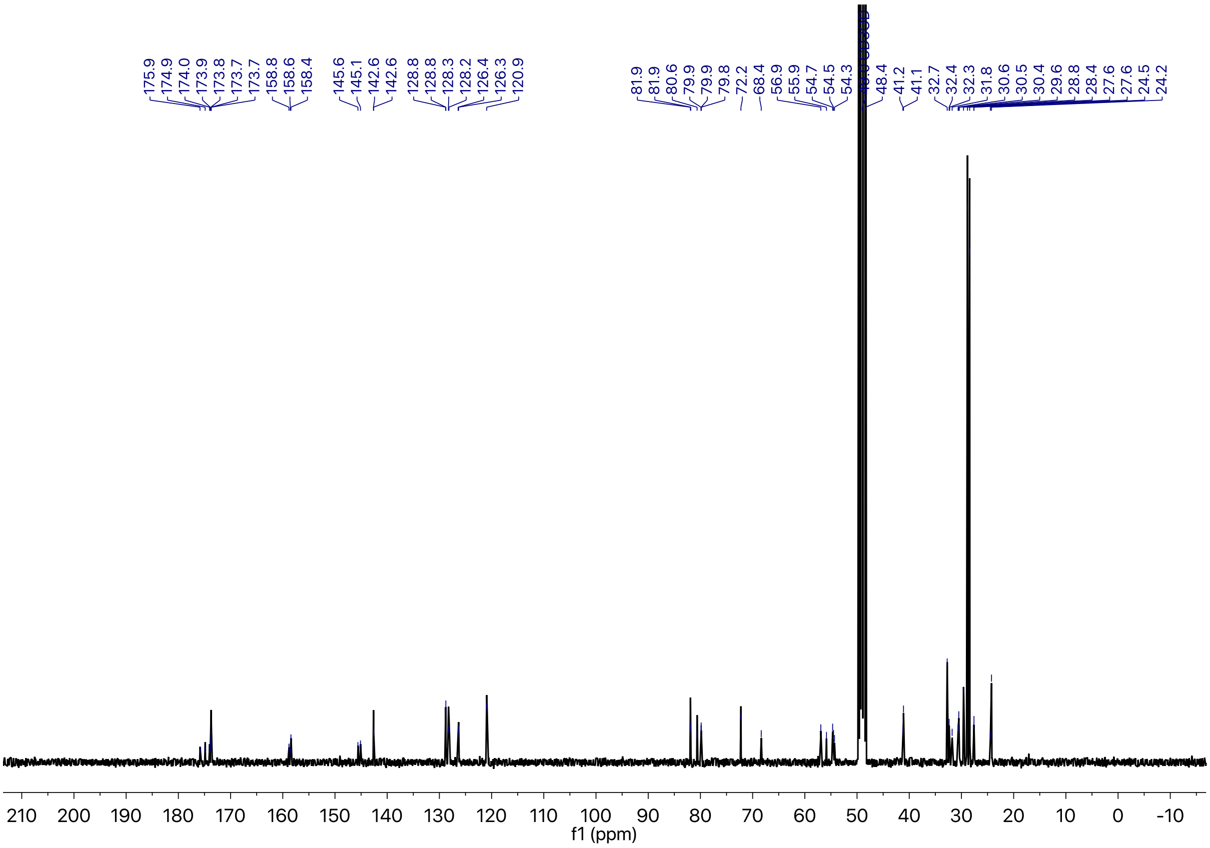

Fig. S35 ^{13}C NMR spectrum (101 MHz) of **67** in CD_3OD.

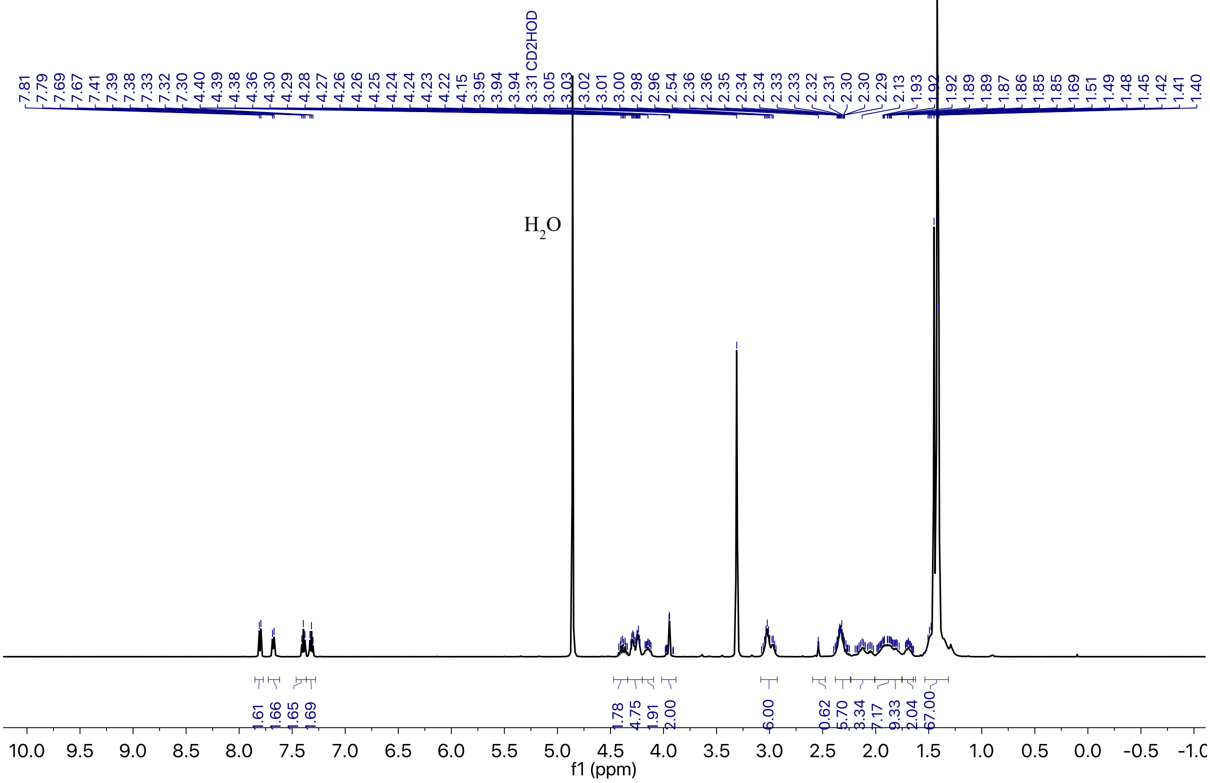

Fig. S36 ^{1}H NMR spectrum (500 MHz) of **69** in CD_3OD.

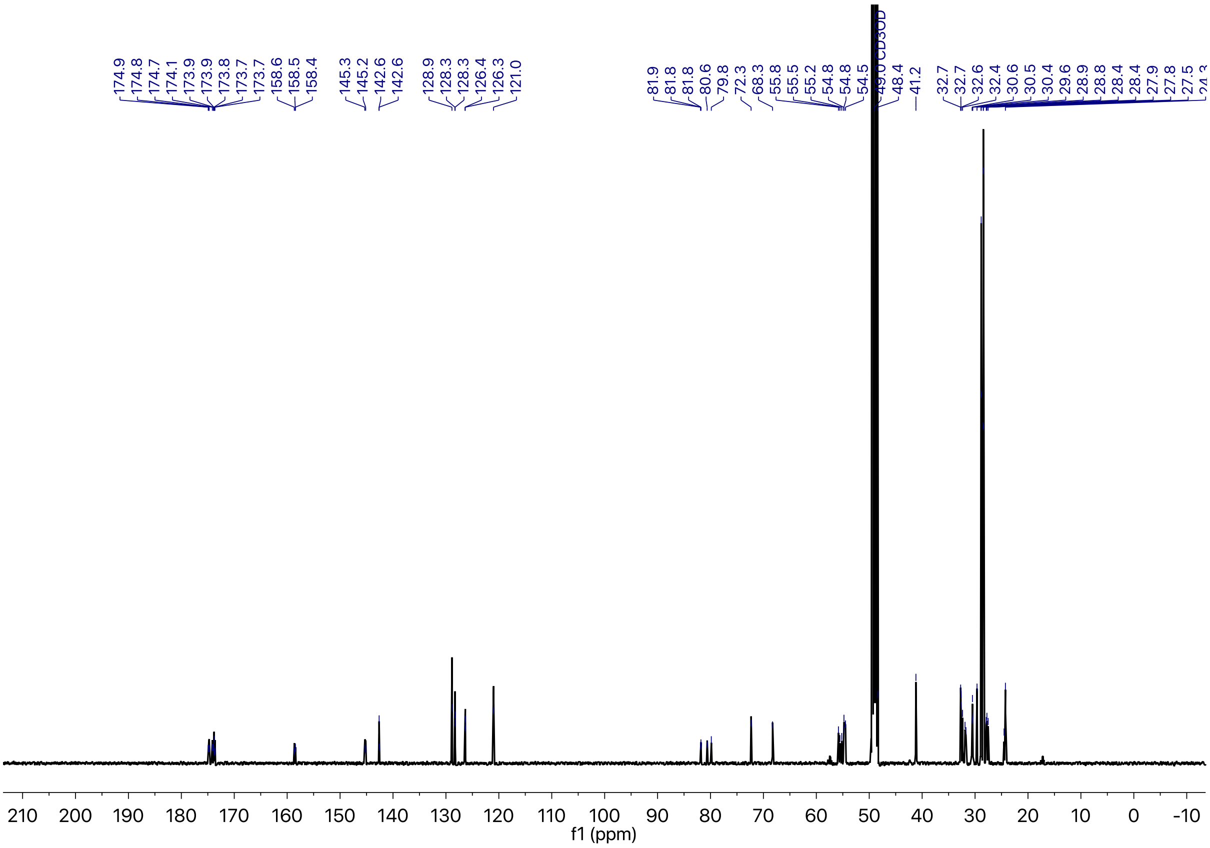

Fig. S37 ^{13}C NMR spectrum (126 MHz) of **69** in CD_3OD.

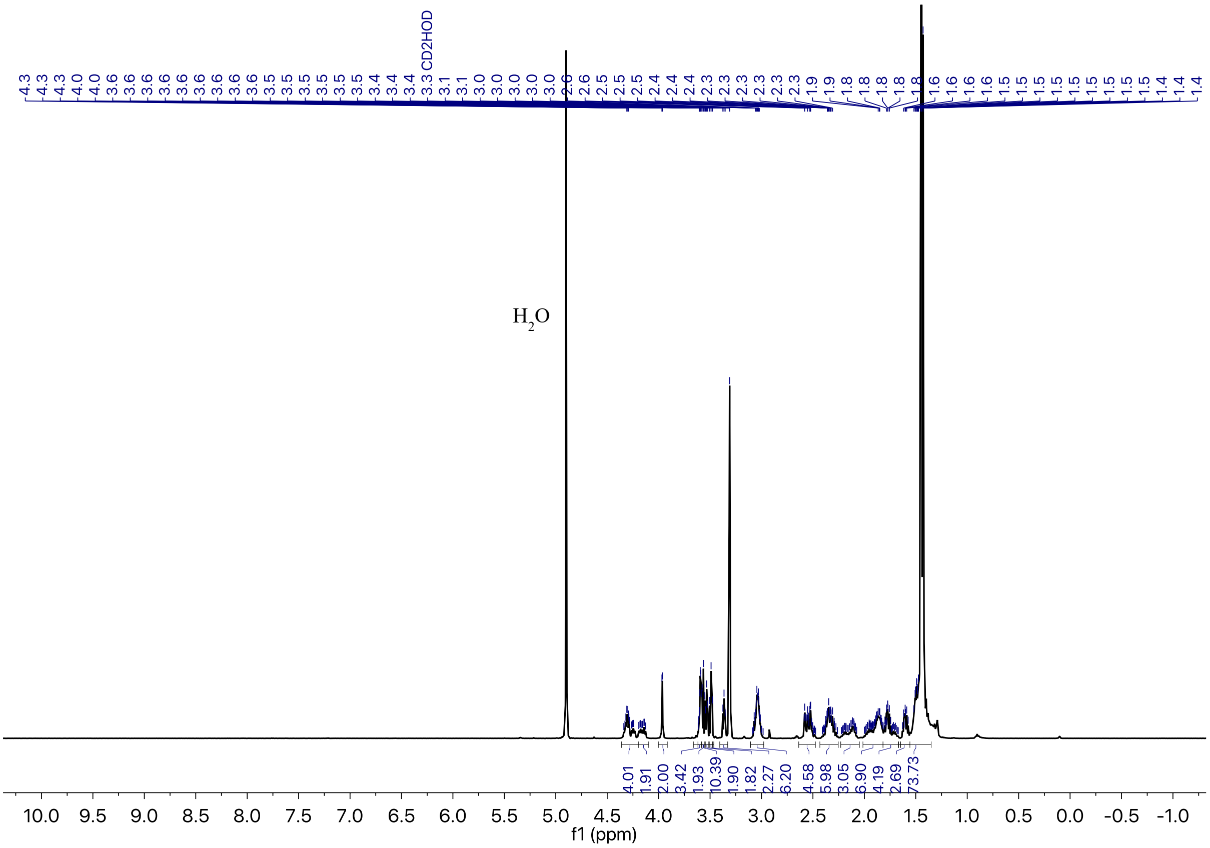

Fig. S38 ^{1}H NMR spectrum (500 MHz) of **71** in CD_3OD.

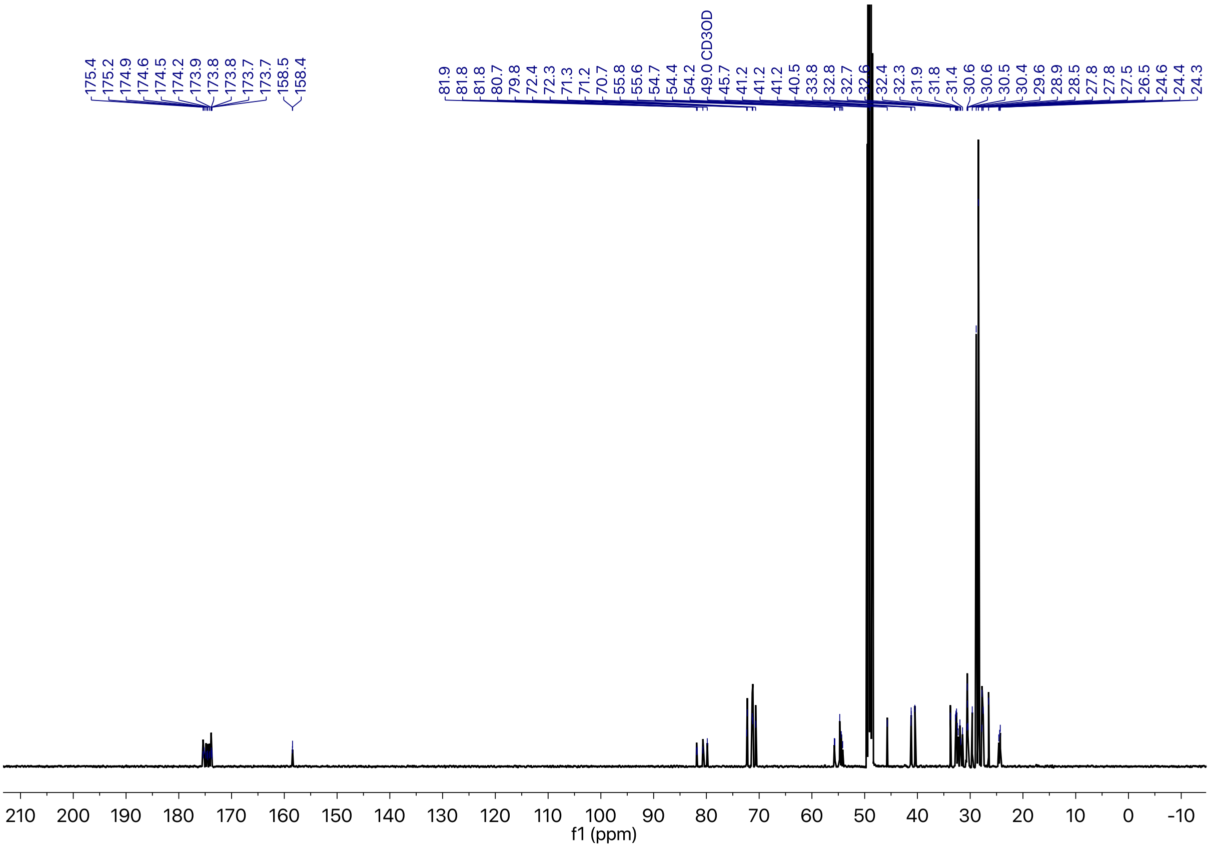

Fig. S39 ^{13}C NMR spectrum (126 MHz) of **71** in CD_3OD.

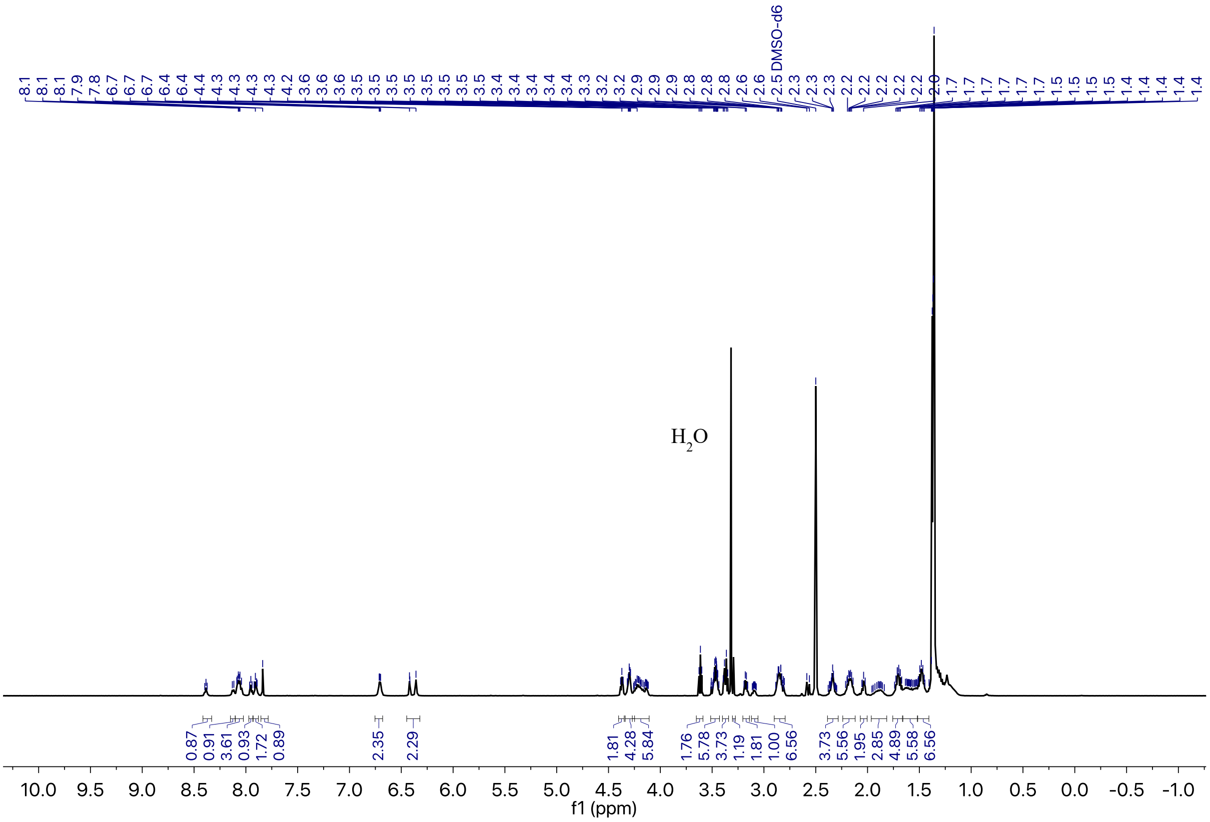

Fig. S40 ^{1}H NMR spectrum (500 MHz) of **72** in CD_3OD.

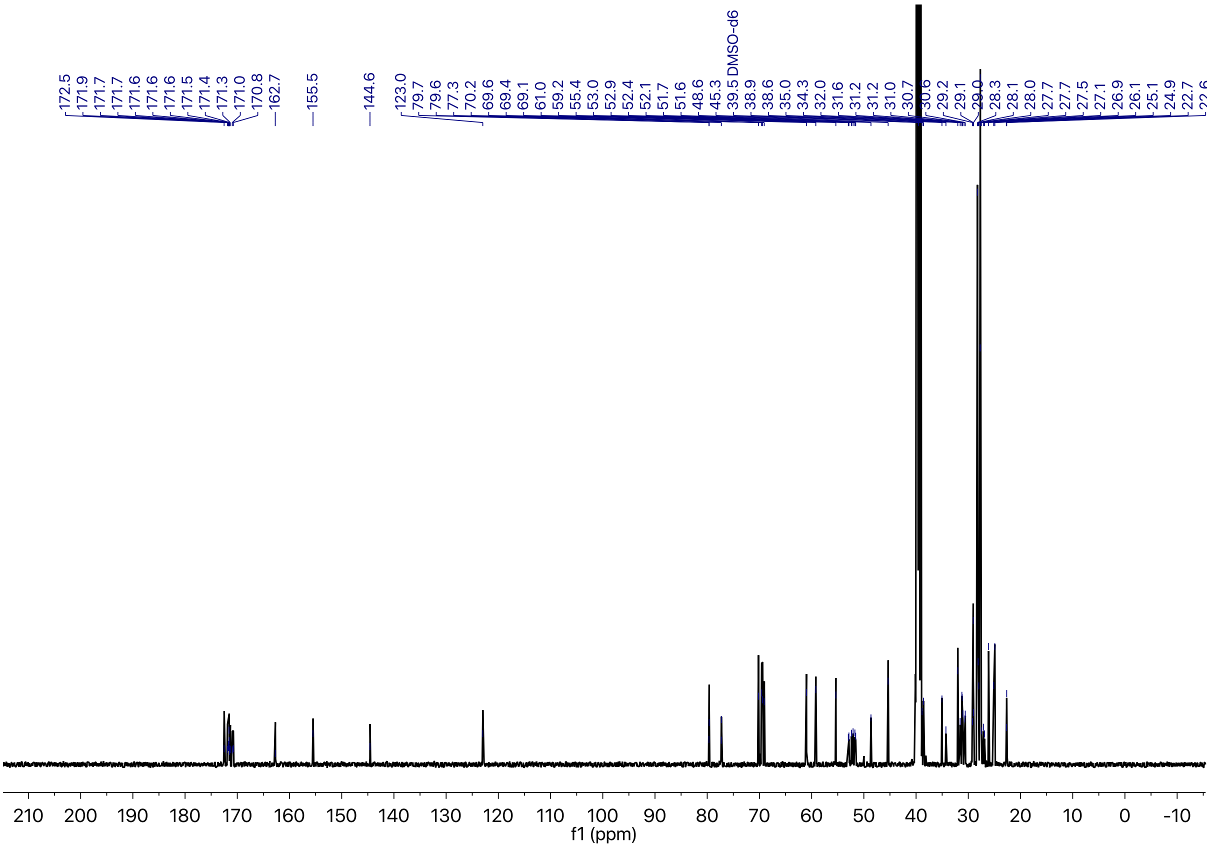

Fig. S41 ^{13}C NMR spectrum (126 MHz) of **72** in CD_3OD.

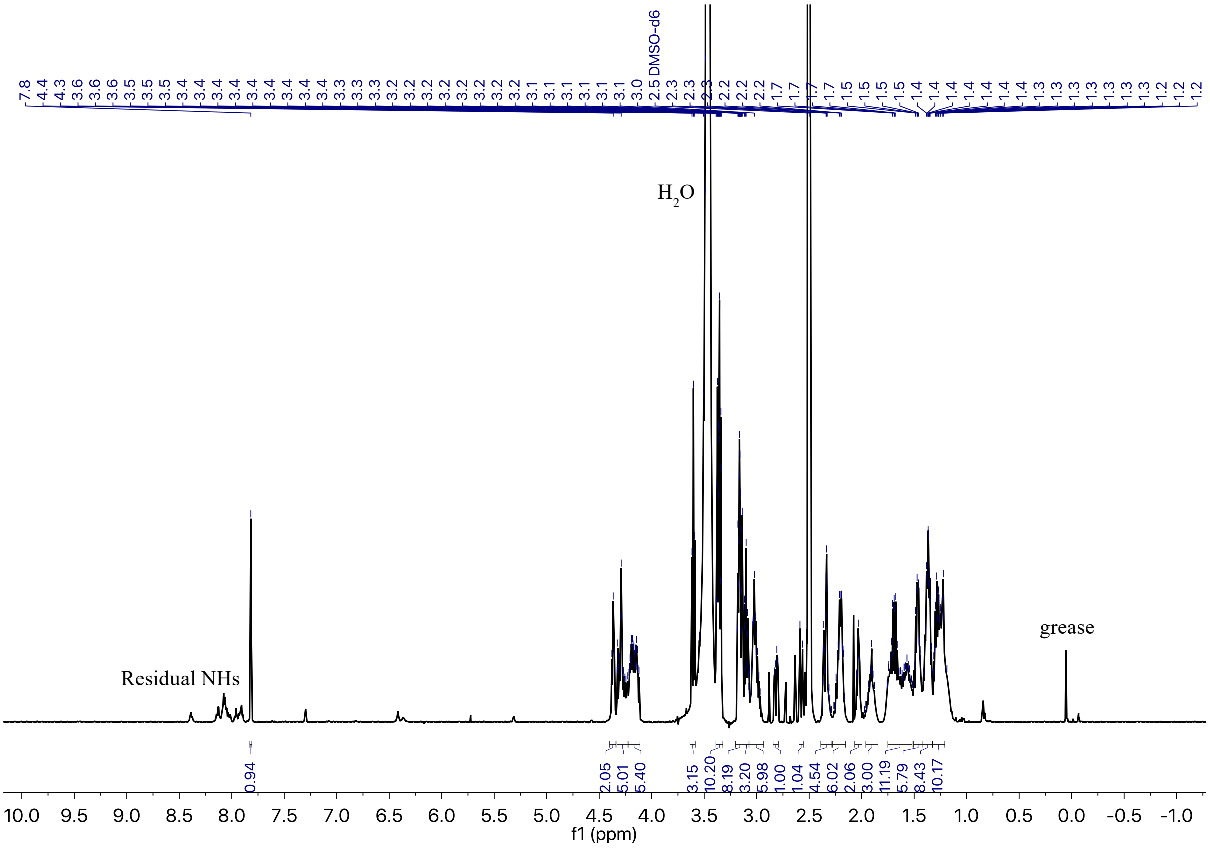

Fig. S42 ^{1}H NMR spectrum (500 MHz) of **7** in DMSO-d_6/D_2O 60:1.

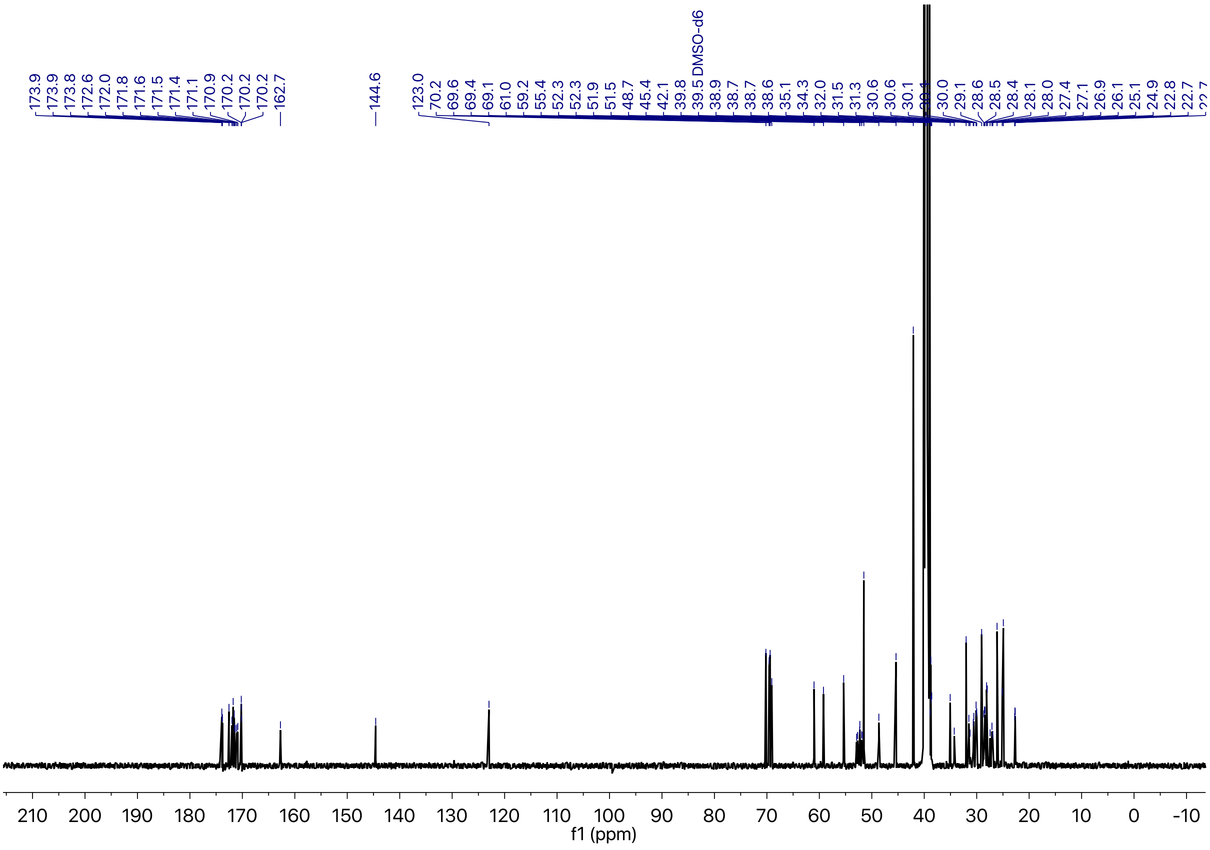

Fig. S43 ^{13}C NMR spectrum (126 MHz) of **7** in DMSO-d_6.

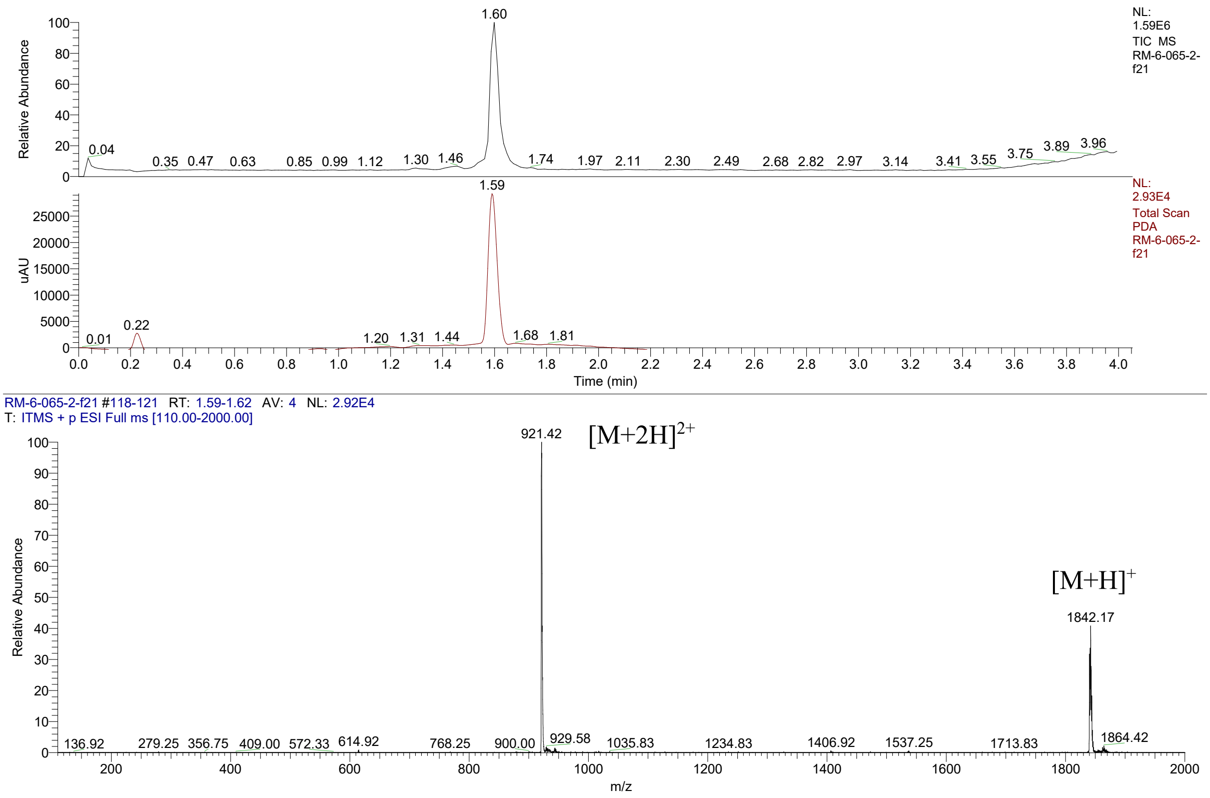

Fig. S44 HPLC-MS profile of **7**.

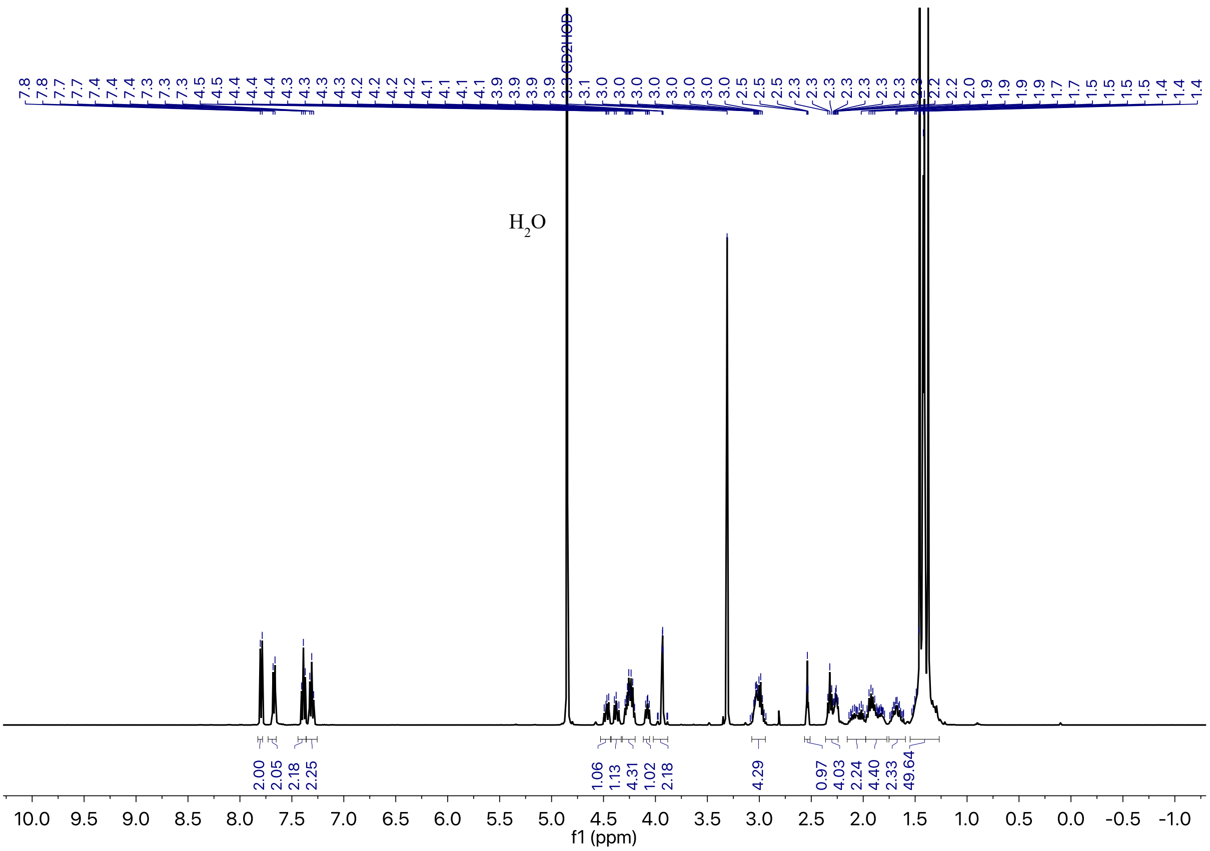

Fig. S45 ^{1}H NMR spectrum (400 MHz) of **74** in CD_3OD.

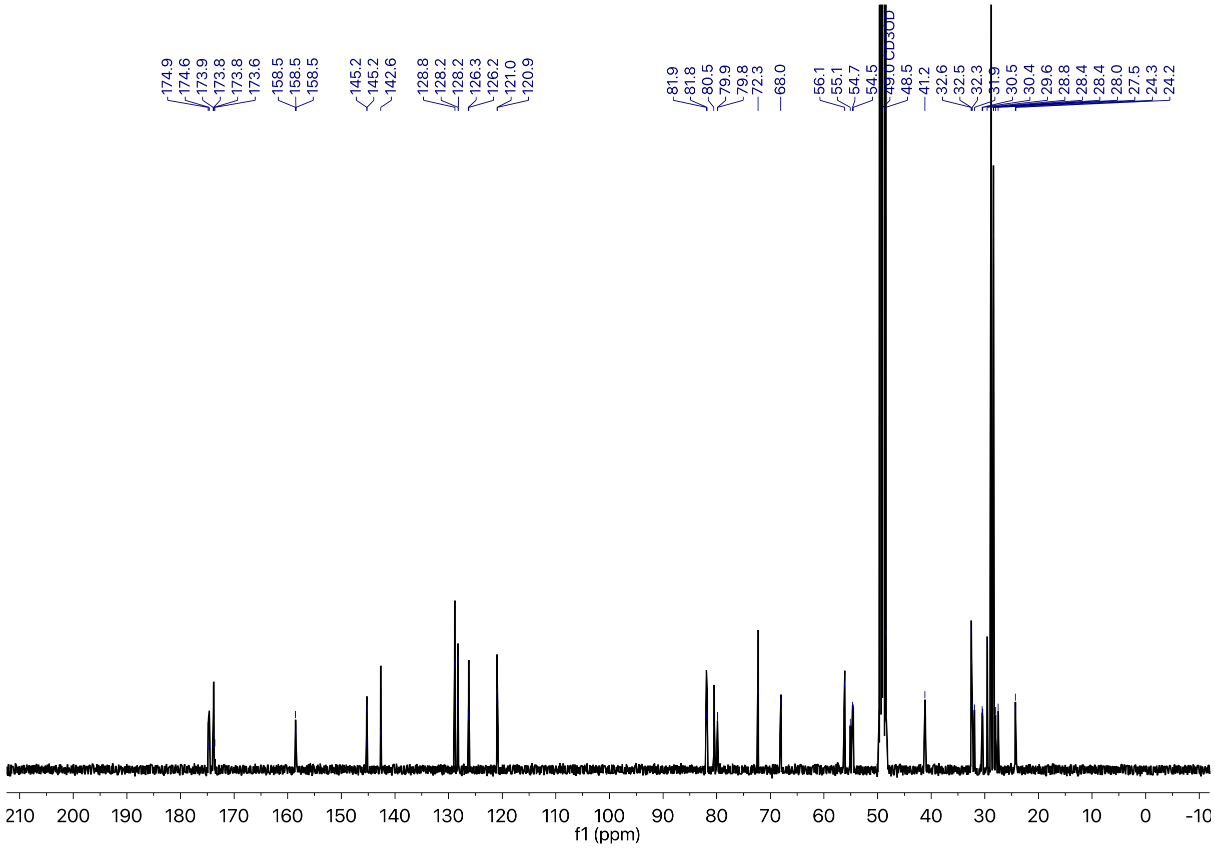

Fig. S46 ^{13}C NMR spectrum (101 MHz) of **74** in CD_3OD.

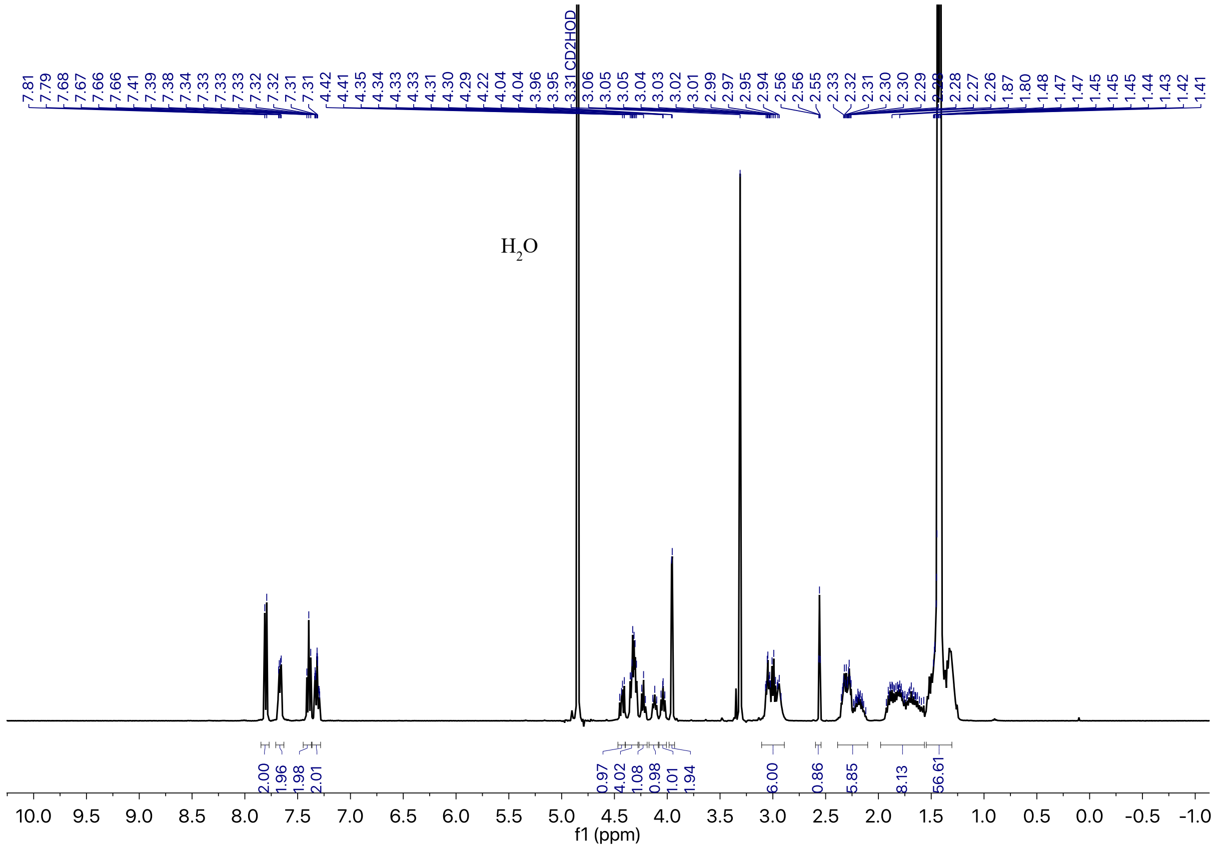

Fig. S47 ^{1}H NMR spectrum (400 MHz) of **76** in CD_3OD.

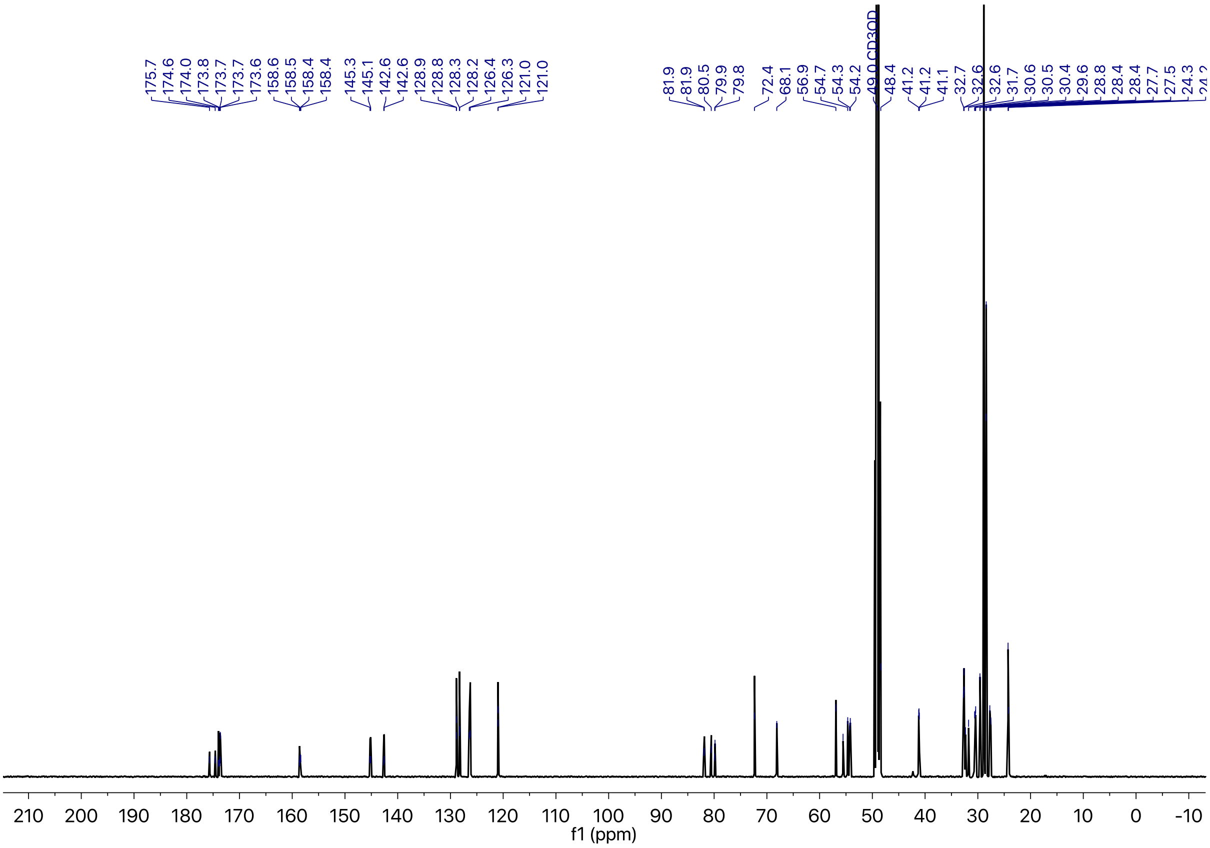

Fig. S48 ^{13}C NMR spectrum (126 MHz) of **76** in CD_3OD.

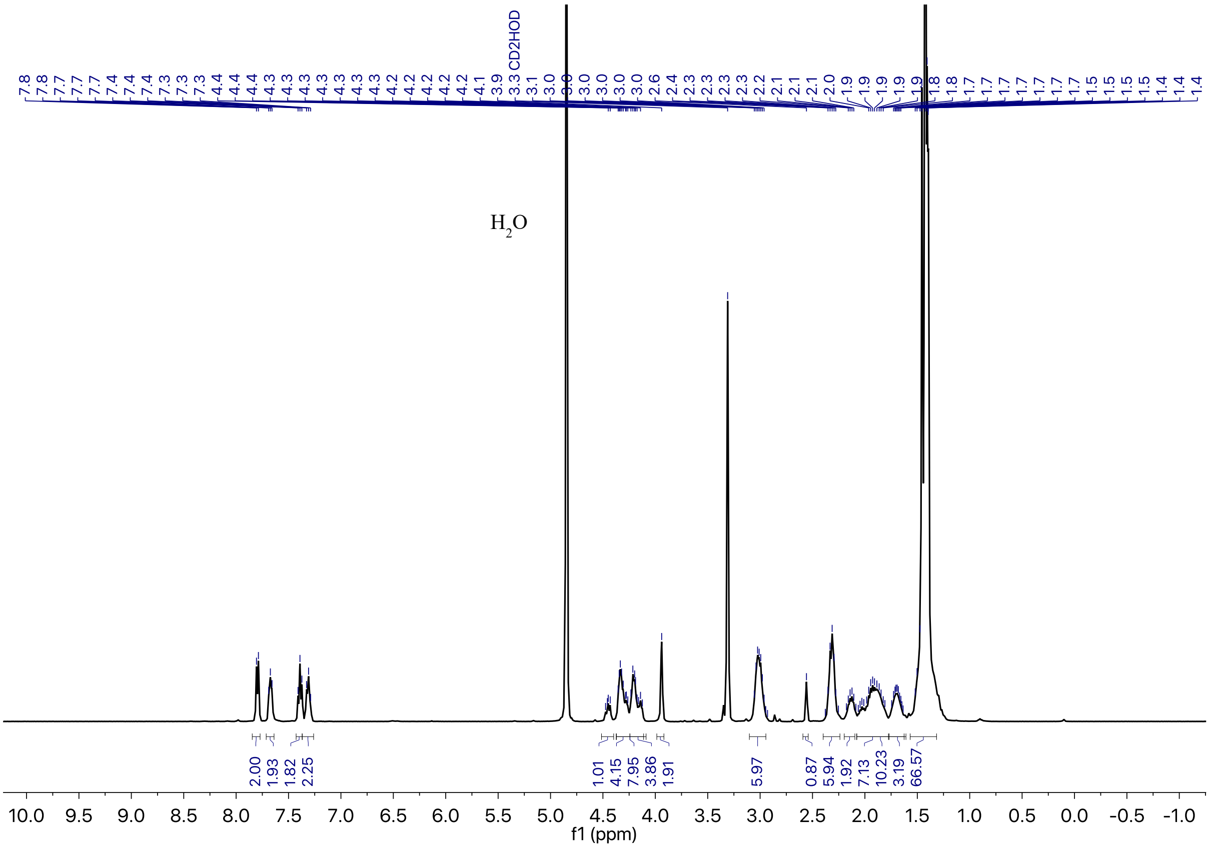

Fig. S49 ^{1}H NMR spectrum (400 MHz) of **78** in CD_3OD.

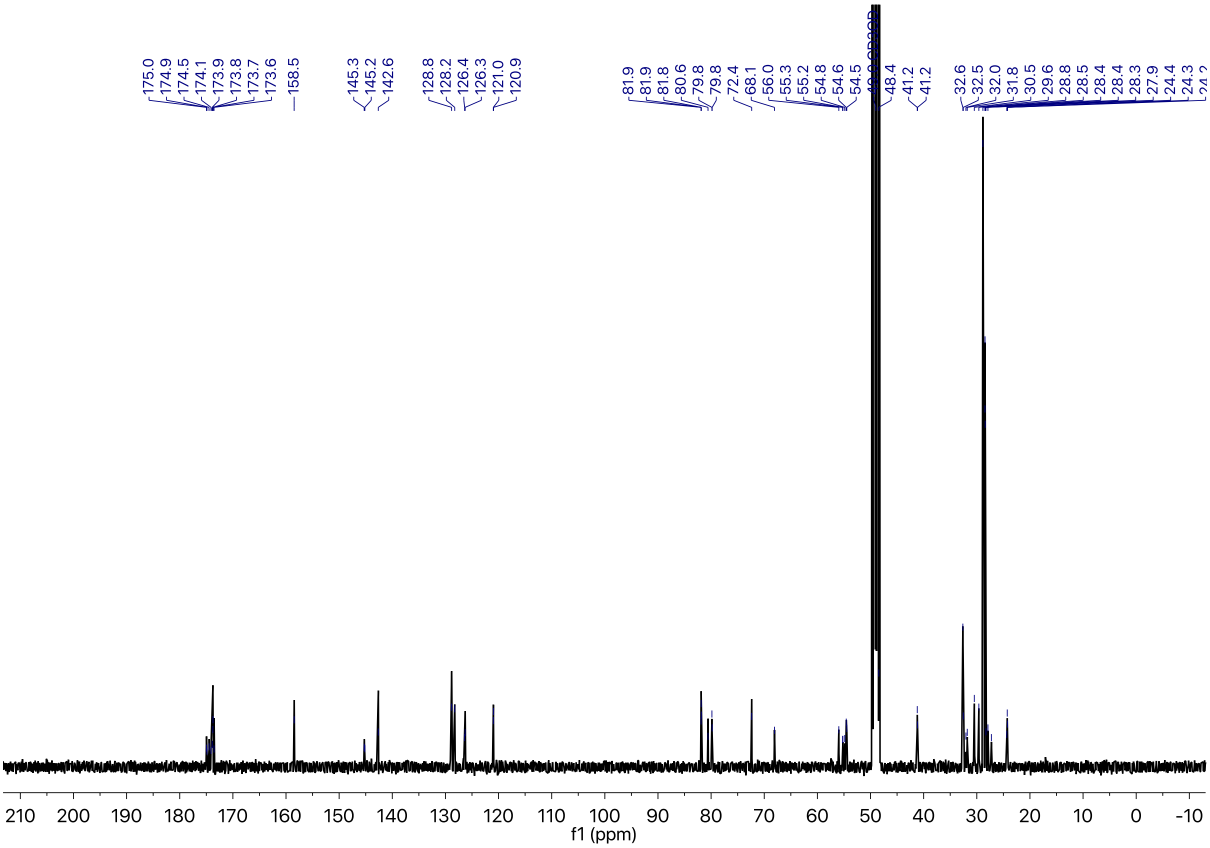

Fig. S50 ^{13}C NMR spectrum (101 MHz) of **78** in CD_3OD.

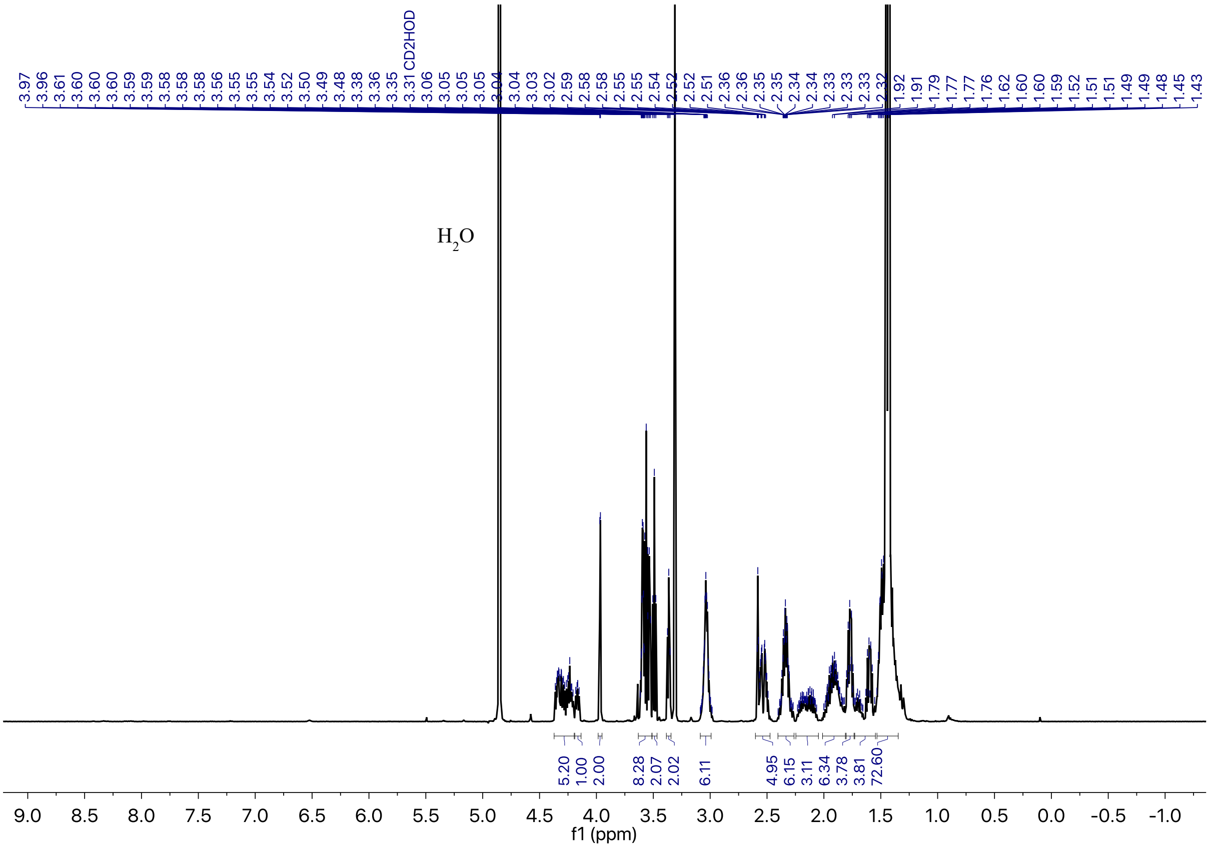

Fig. S51 ^{1}H NMR spectrum (500 MHz) of **80** in CD_3OD.

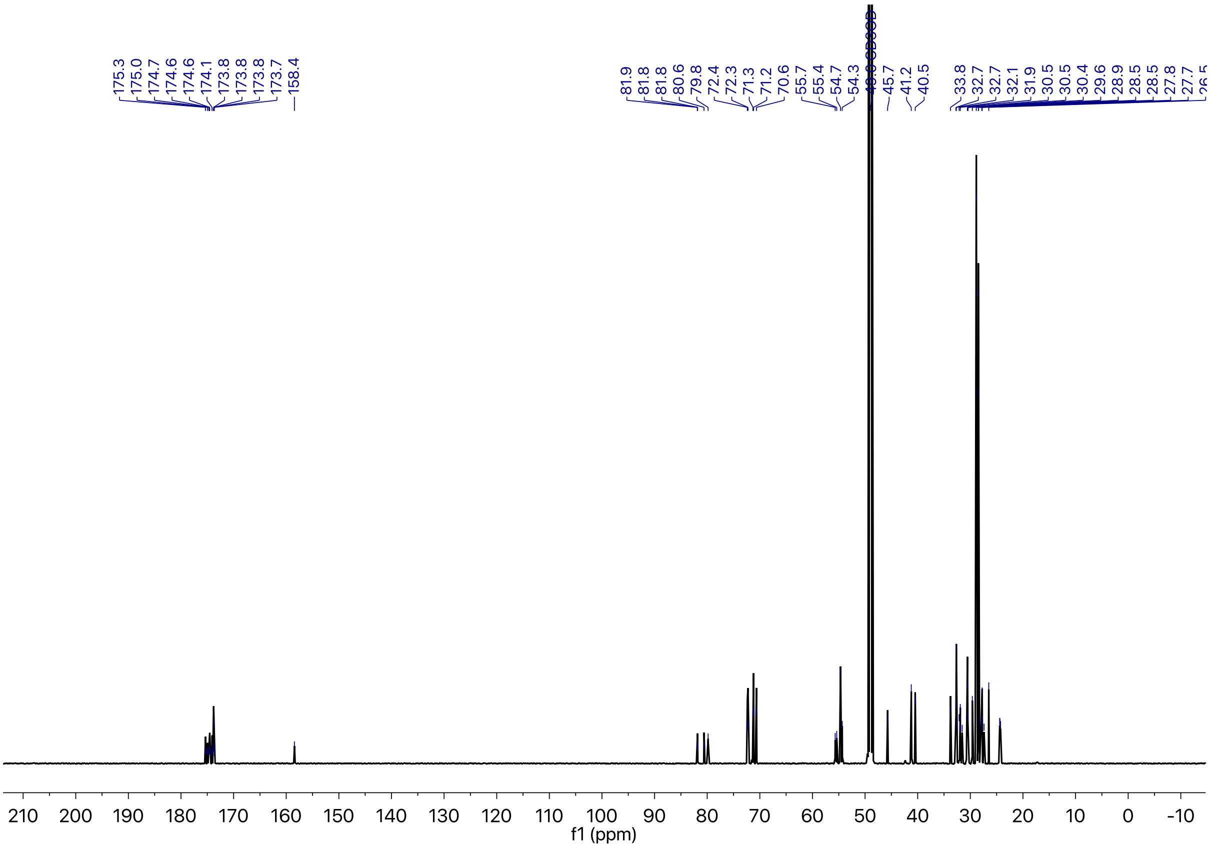

Fig. S52 ^{13}C NMR spectrum (126 MHz) of **80** in CD_3OD.

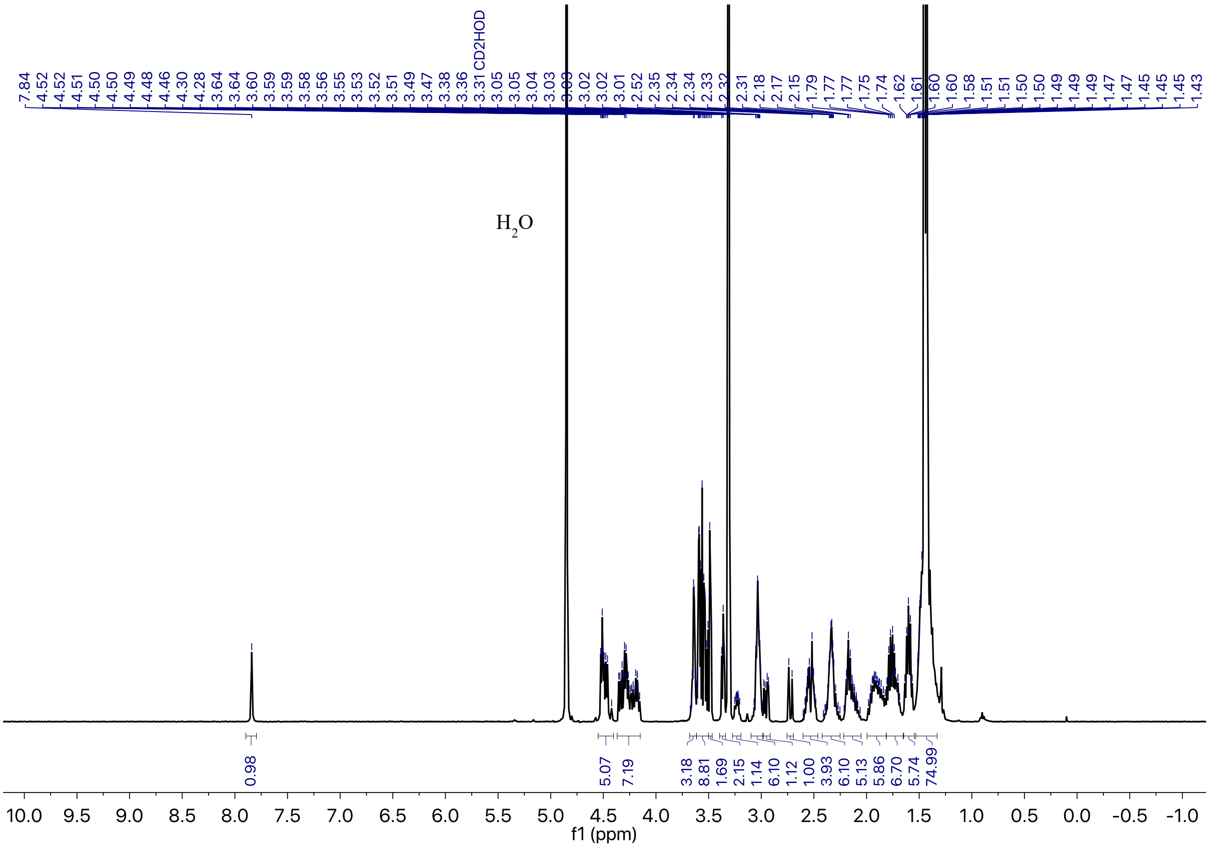

Fig. S53 ^{1}H NMR spectrum (400 MHz) of **81** in CD_3OD.

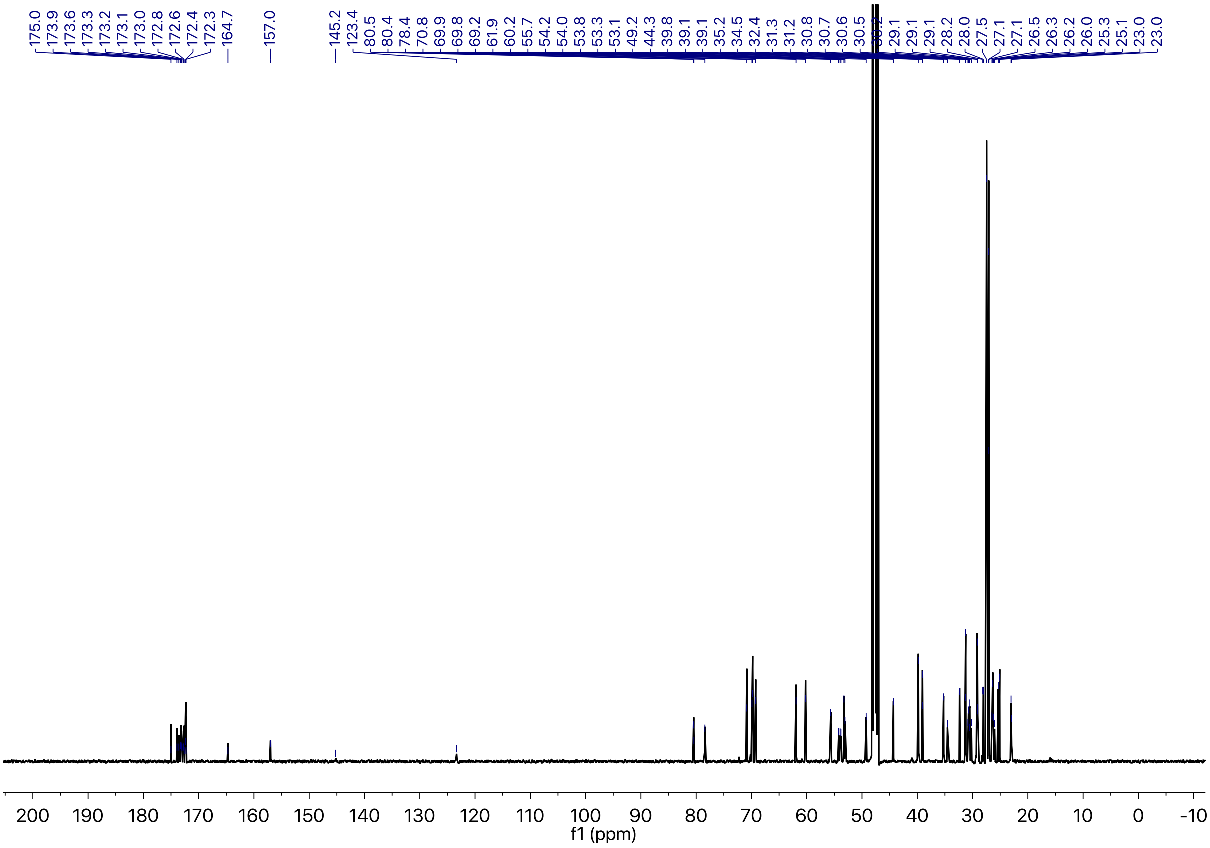

Fig. S54 ^{13}C NMR spectrum (126 MHz) of **81** in CD_3OD.

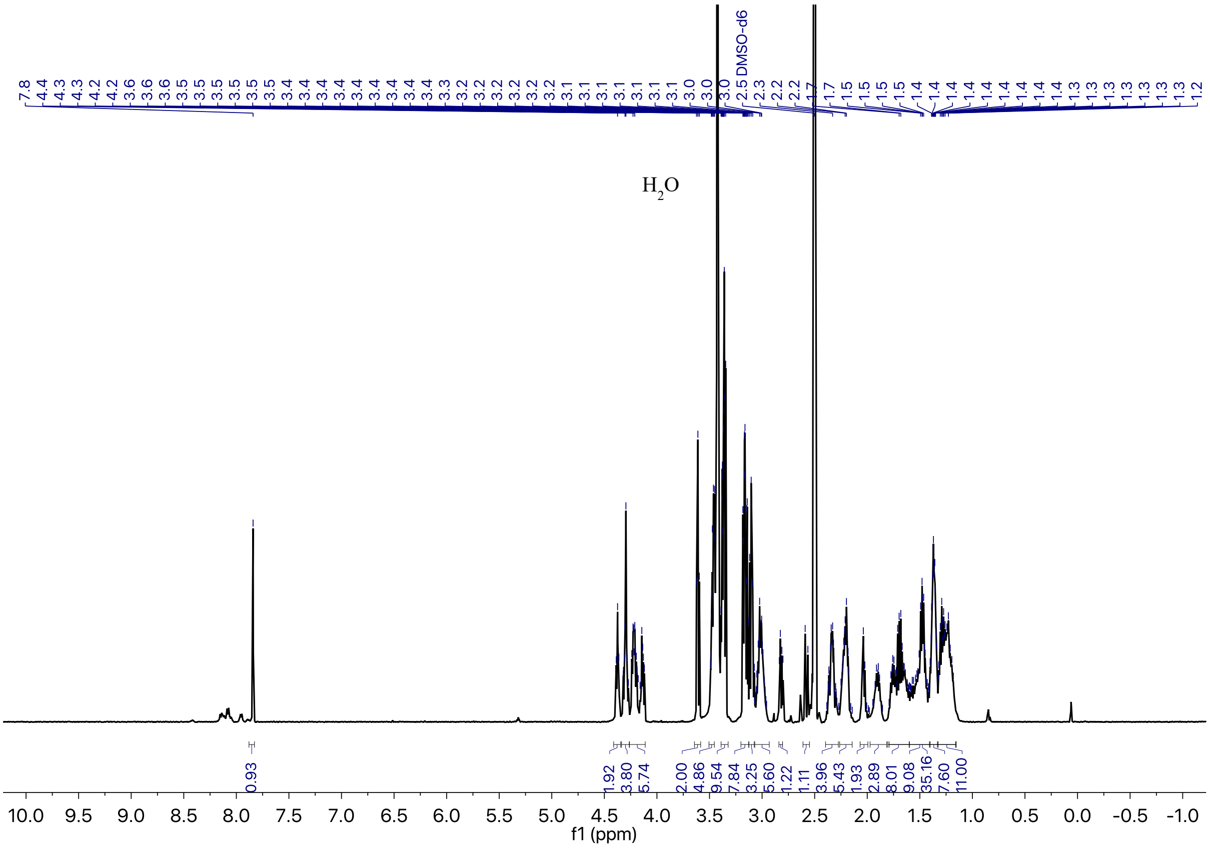

Fig. S55 ^{1}H NMR spectrum (500 MHz) of **8** in DMSO-d_6/D_2O 60:1.

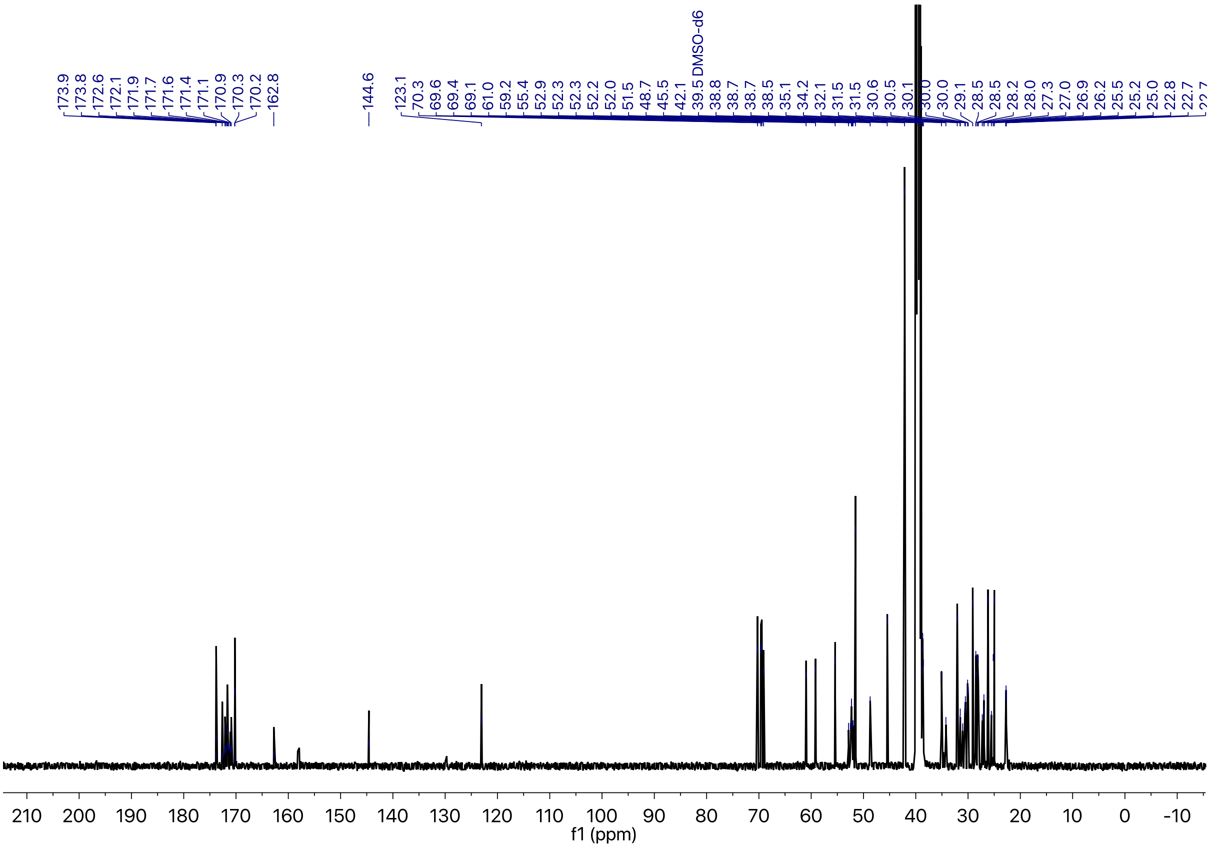

Fig. S56 ^{13}C NMR spectrum (126 MHz) of **8** in DMSO-d_6.

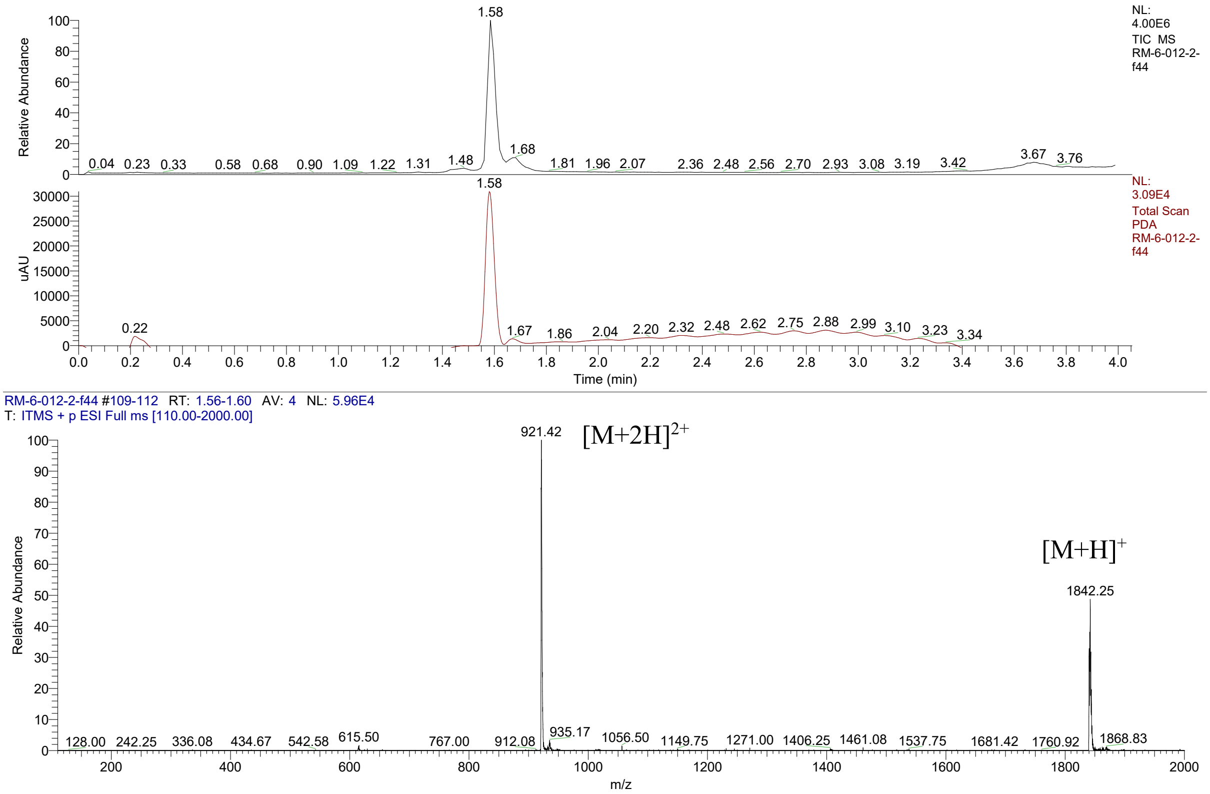

Fig. S57 HPLC-MS profile of **8**.

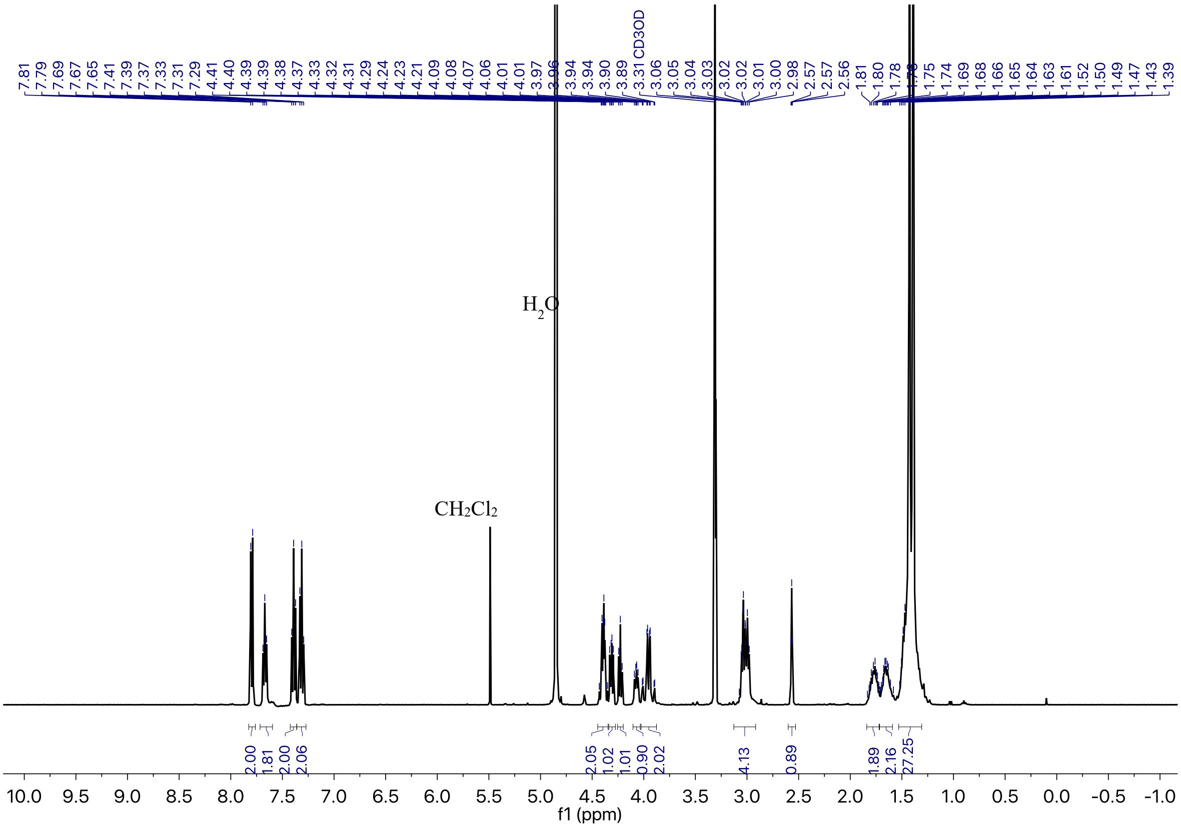

Fig. S58 ^{1}H NMR spectrum (400 MHz) of **83** in CD_3OD.

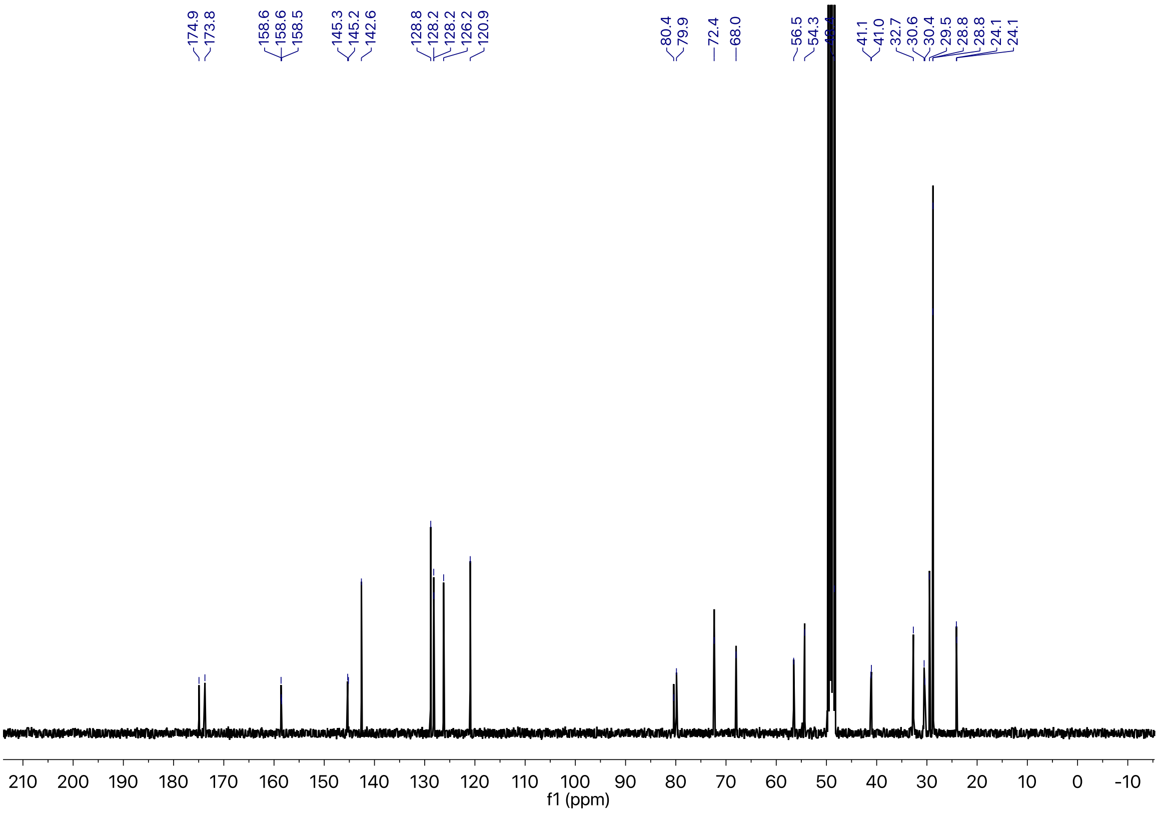

Fig. S59 ^{13}C NMR spectrum (101 MHz) of **83** in CD_3OD.

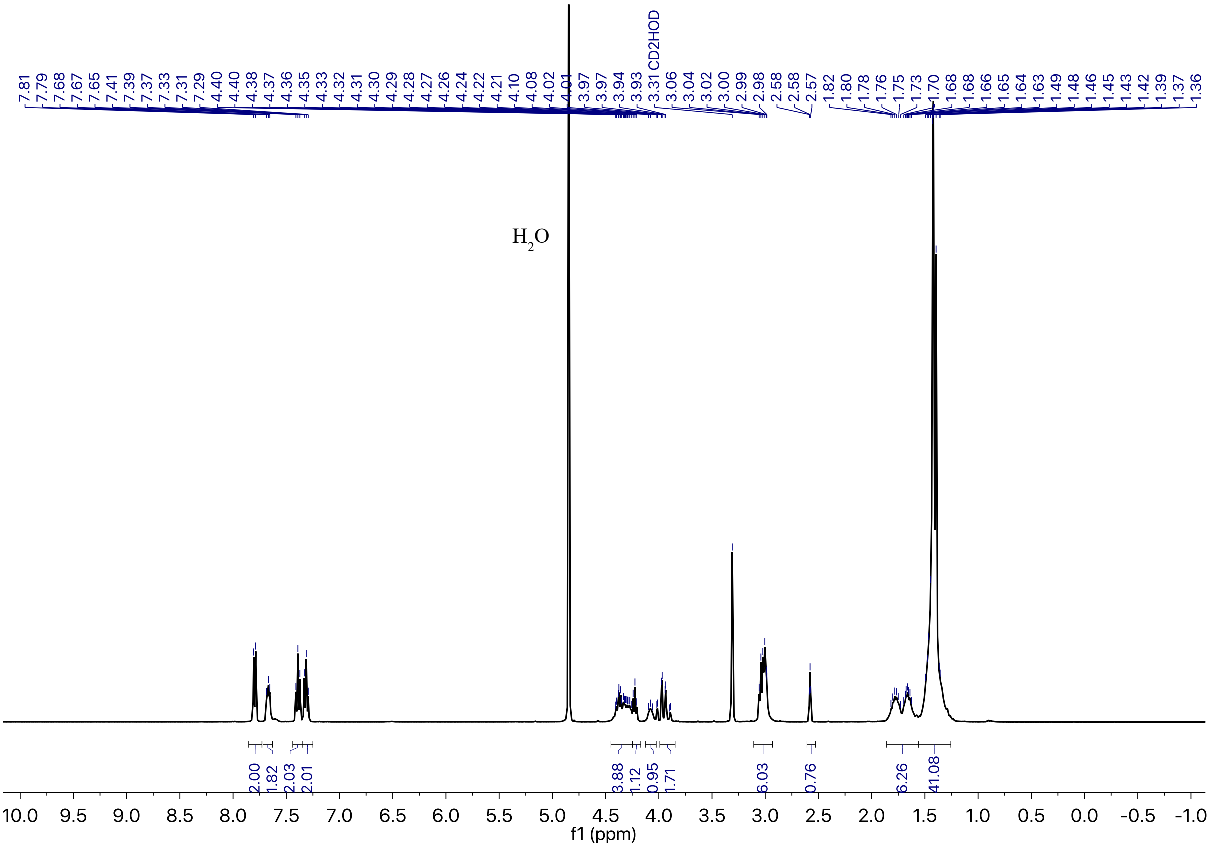

Fig. S60 ^{1}H NMR spectrum (400 MHz) of **85** in CD_3OD.

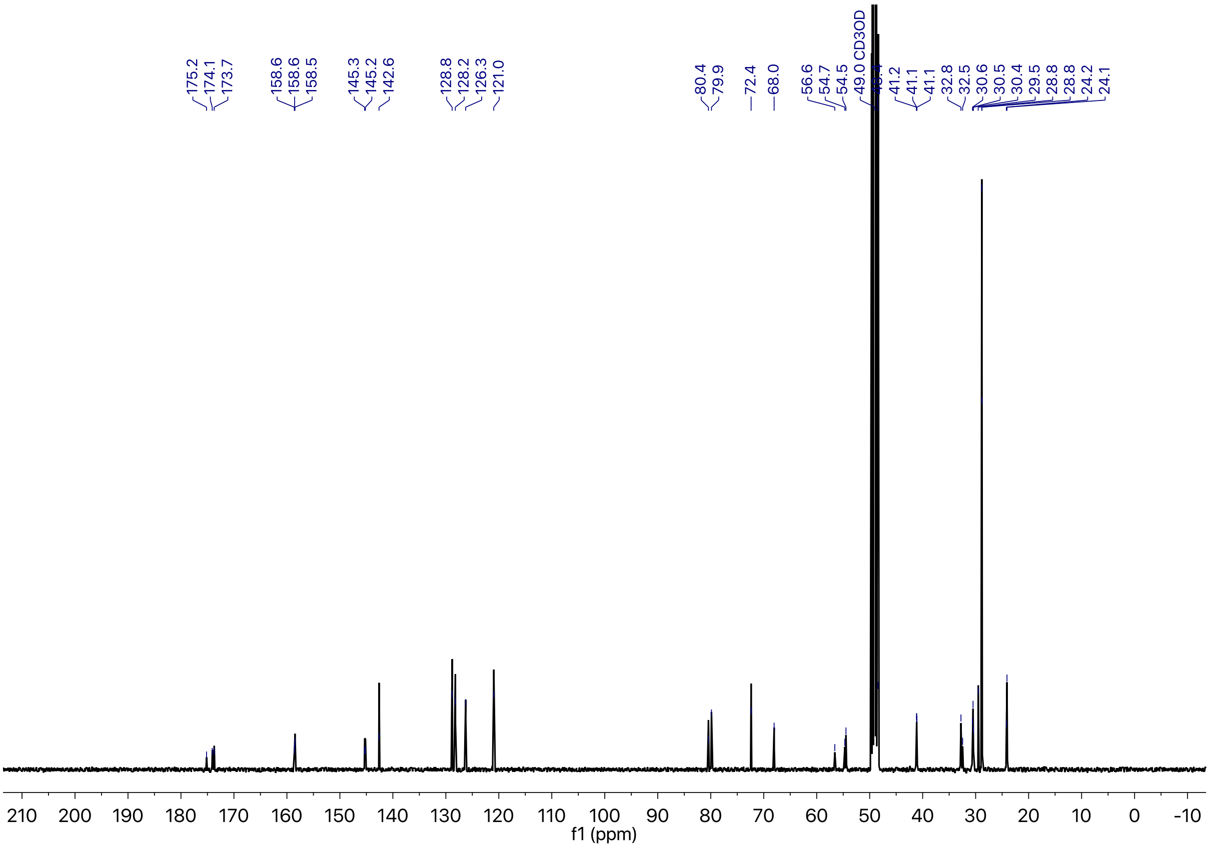

Fig. S61 ^{13}C NMR spectrum (101 MHz) of **85** in CD_3OD.

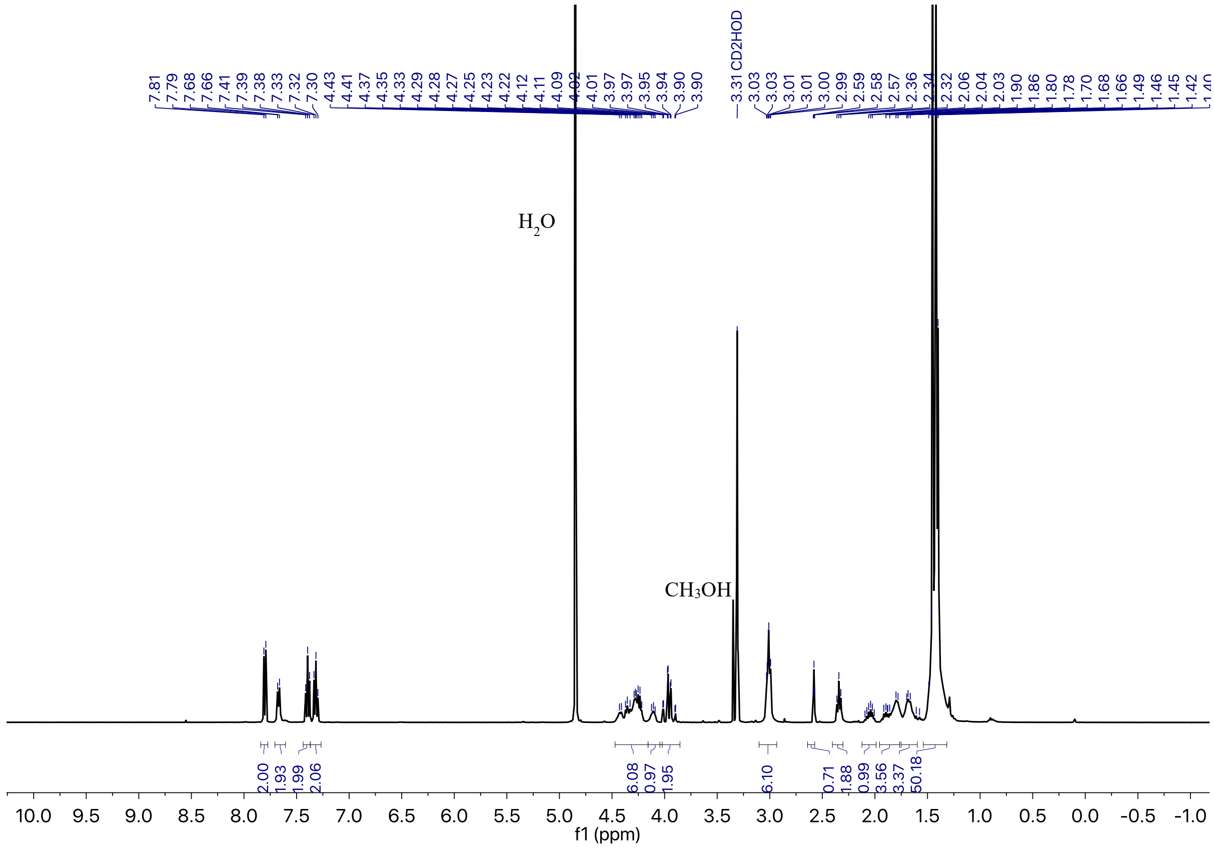

Fig. S62 ^{1}H NMR spectrum (400 MHz) of **87** in CD_3OD.

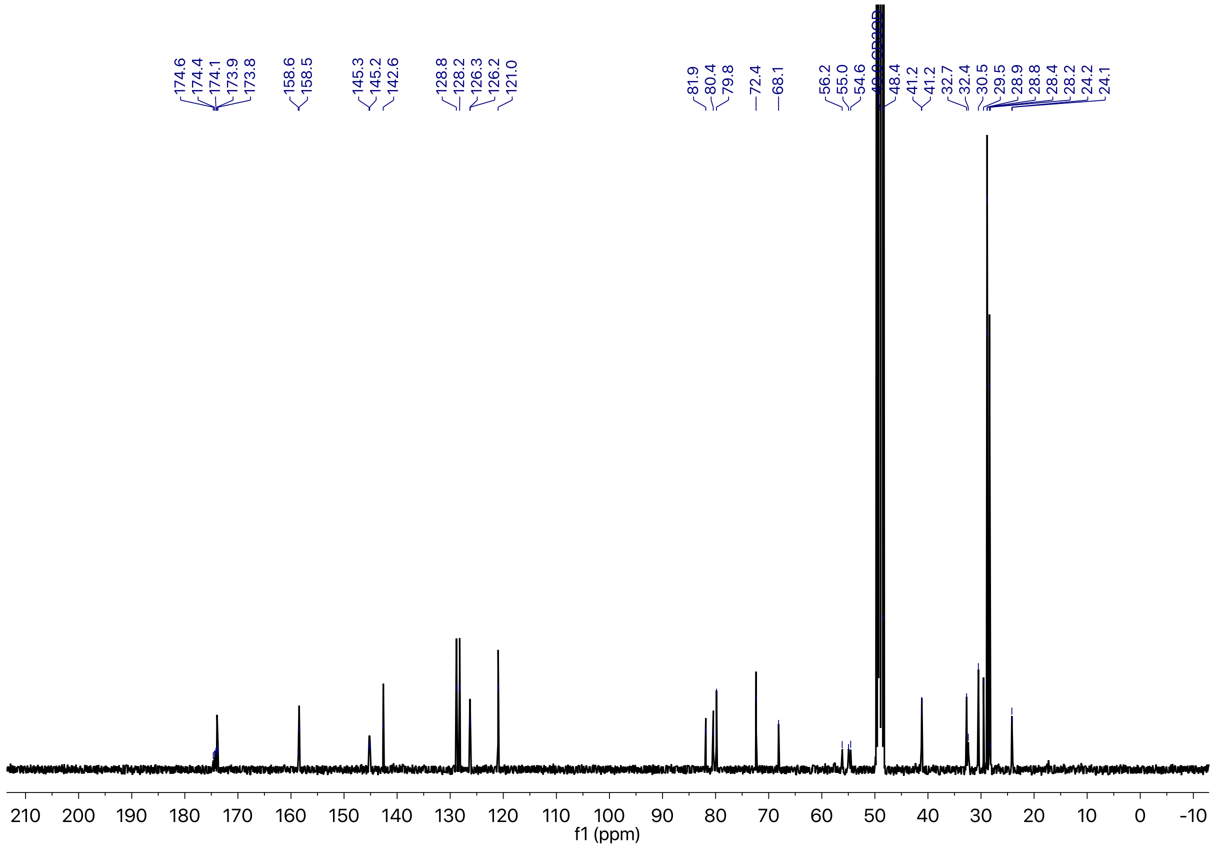

Fig. S63 ^{13}C NMR spectrum (101 MHz) of **87** in CD_3OD.

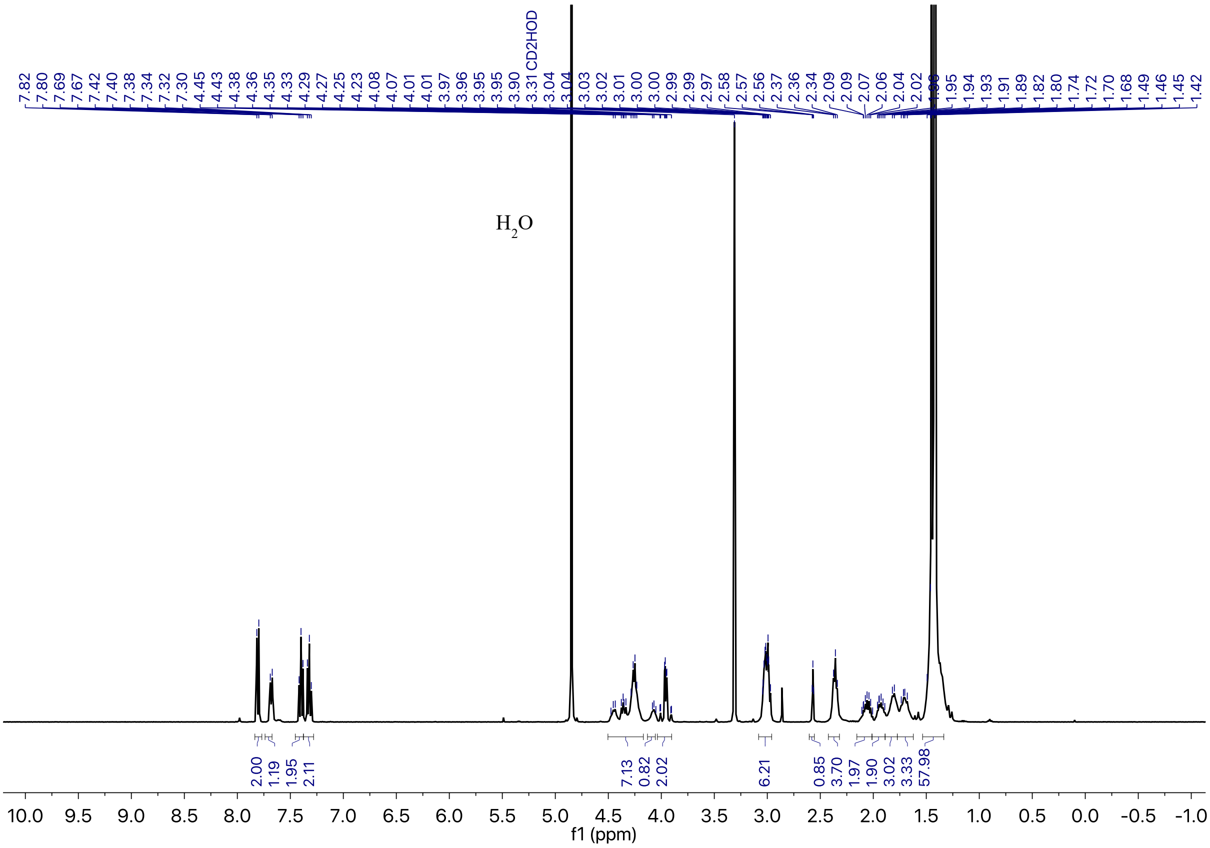

Fig. S64 ^{1}H NMR spectrum (400 MHz) of **89** in CD_3OD.

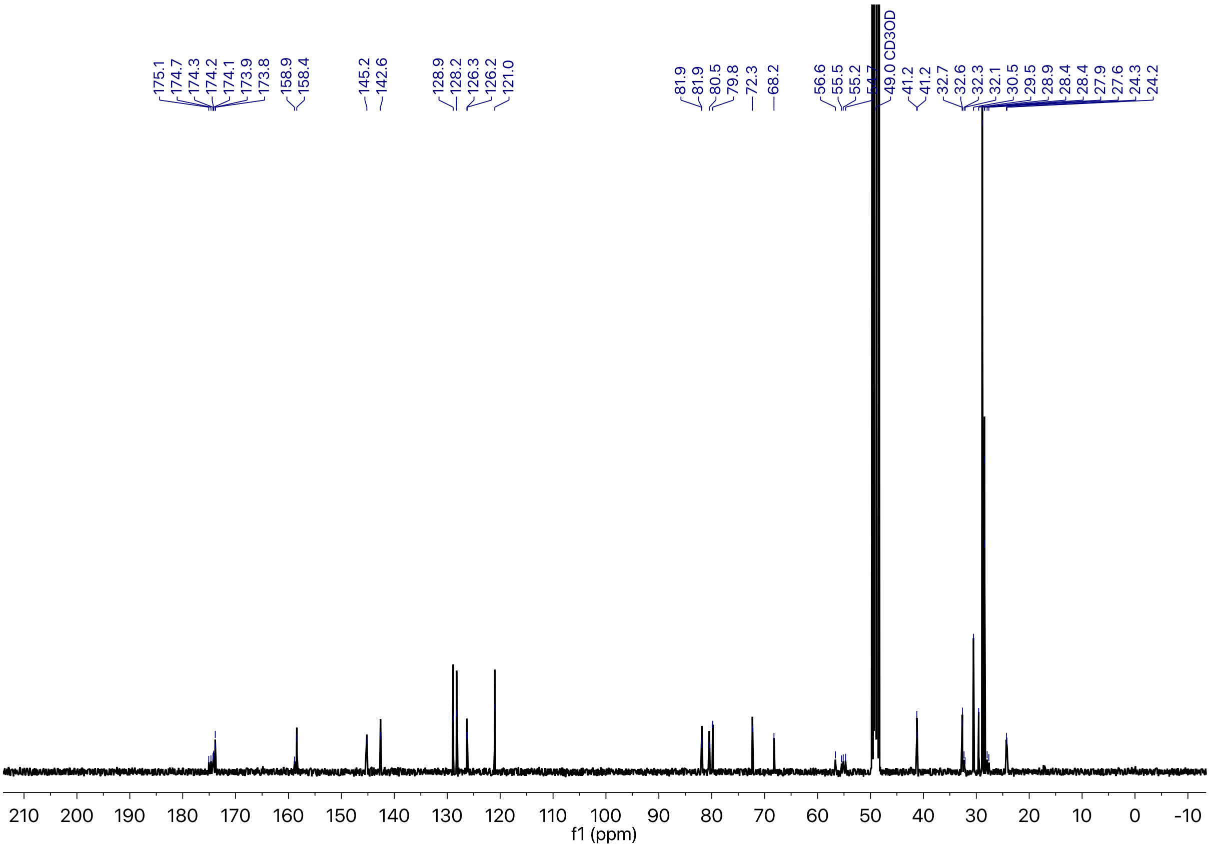

Fig. S65 ^{13}C NMR spectrum (101 MHz) of **89** in CD_3OD.

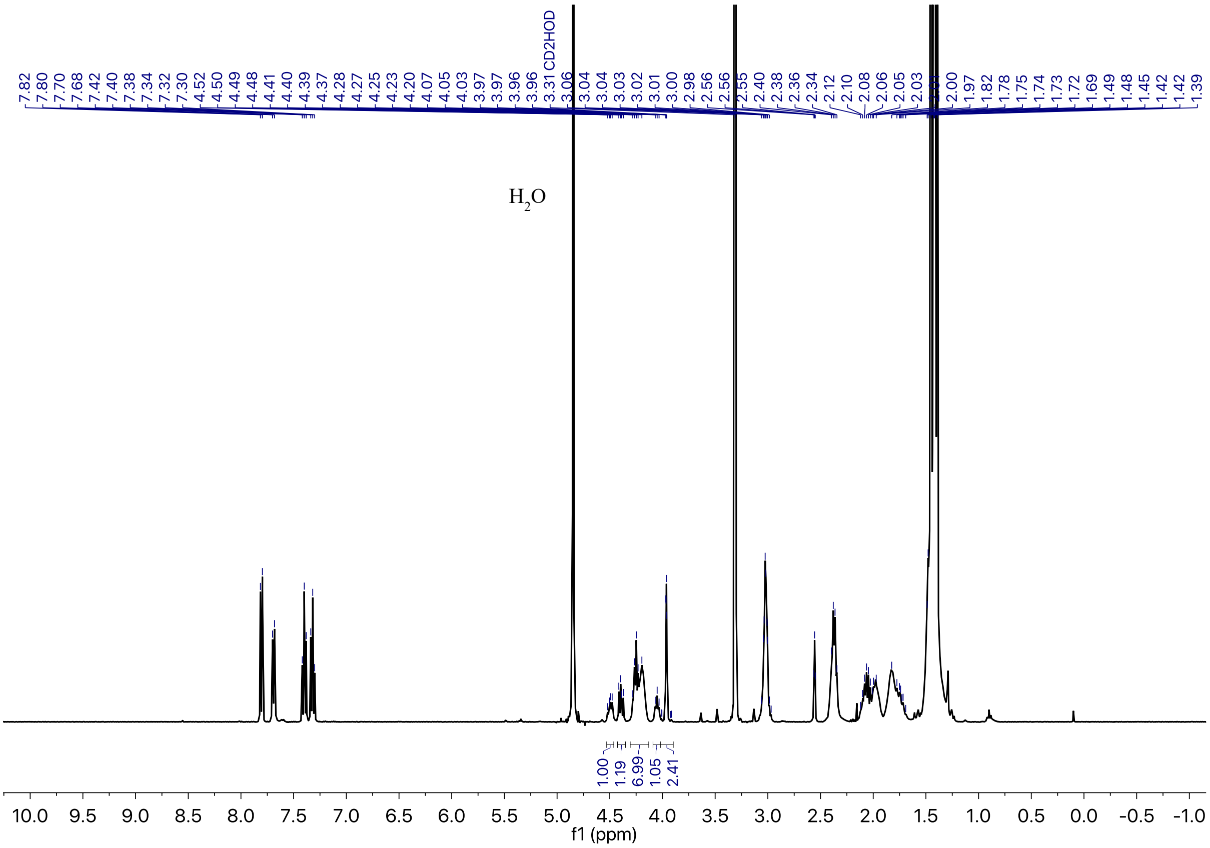

Fig. S66 ^{1}H NMR spectrum (400 MHz) of **91** in CD_3OD.

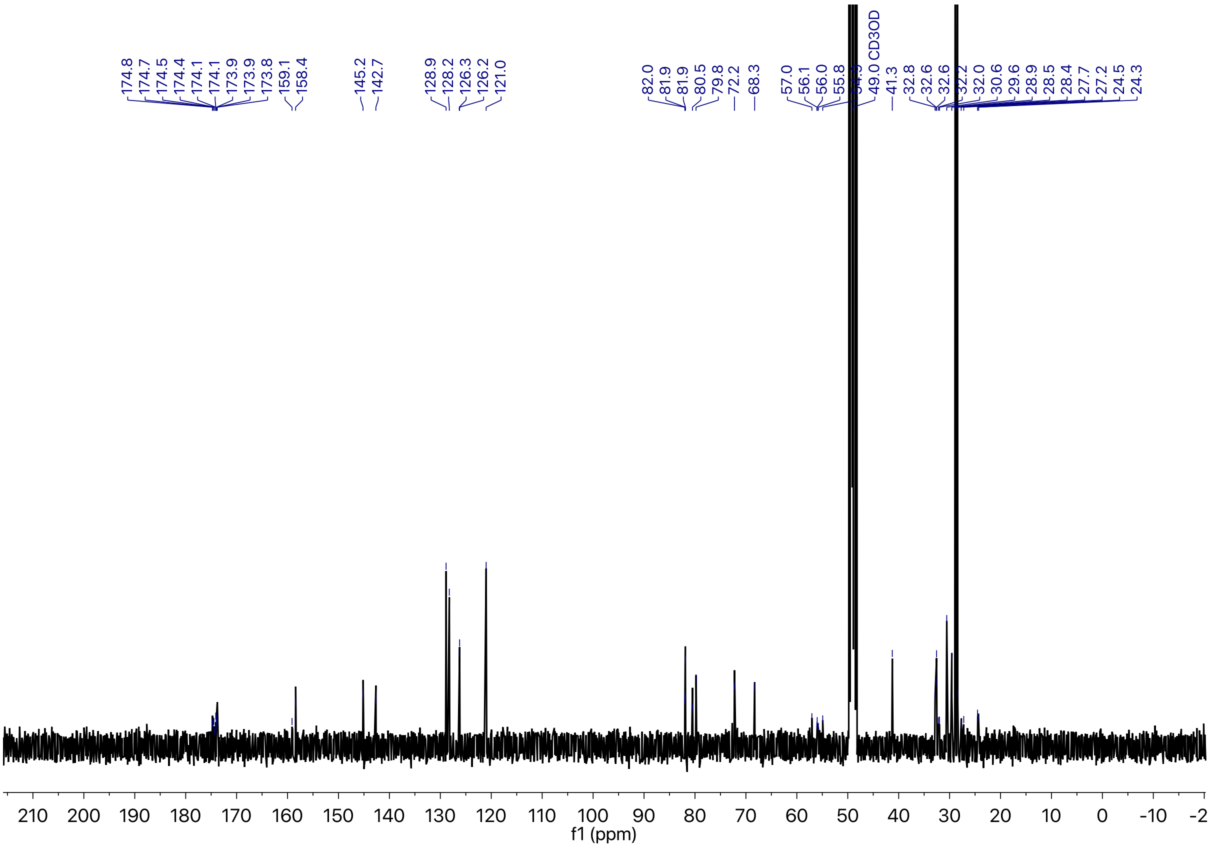

Fig. S67 ^{13}C NMR spectrum (101 MHz) of **91** in CD_3OD.

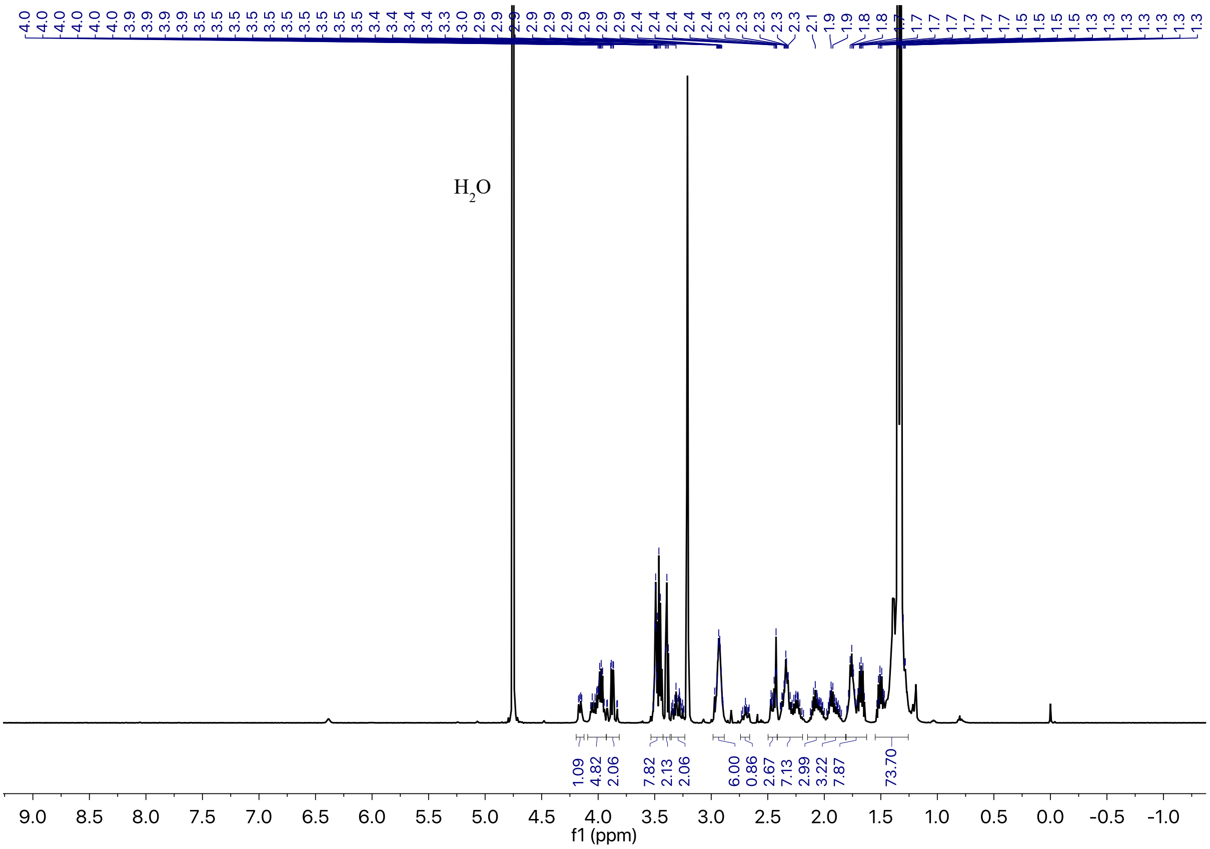

Fig. S68 ^{1}H NMR spectrum (500 MHz) of **93** in CD_3OD.

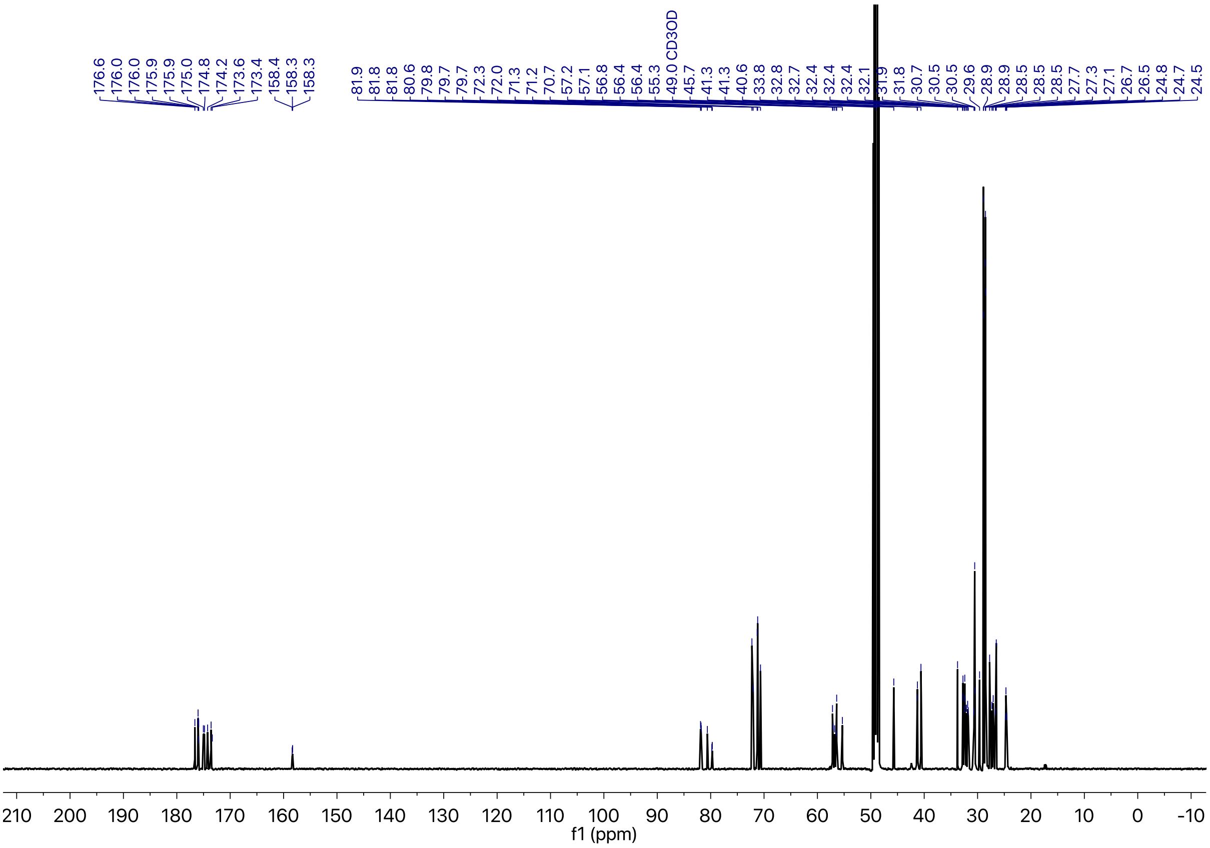

Fig. S69 ^{13}C NMR spectrum (126 MHz) of **93** in CD_3OD.

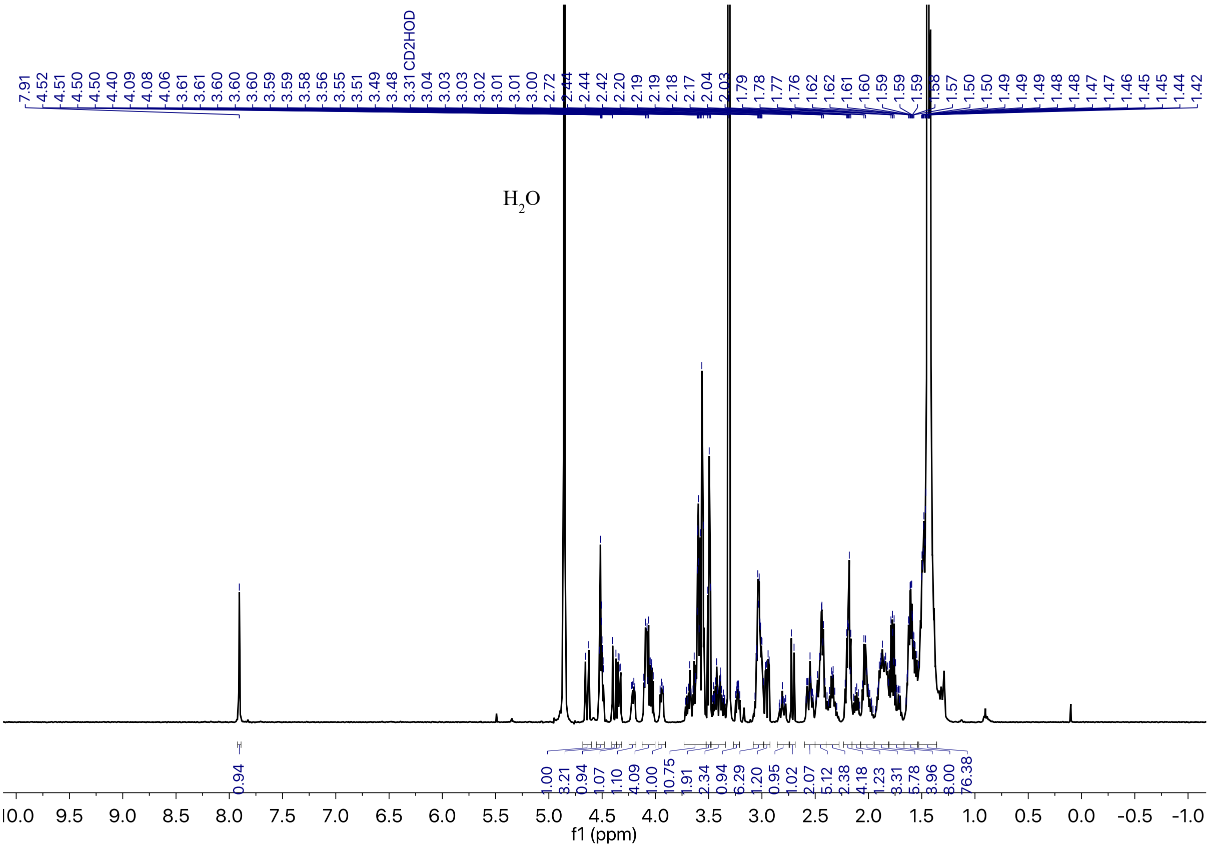

Fig. S70 ^{1}H NMR spectrum (500 MHz) of **94** in CD_3OD.

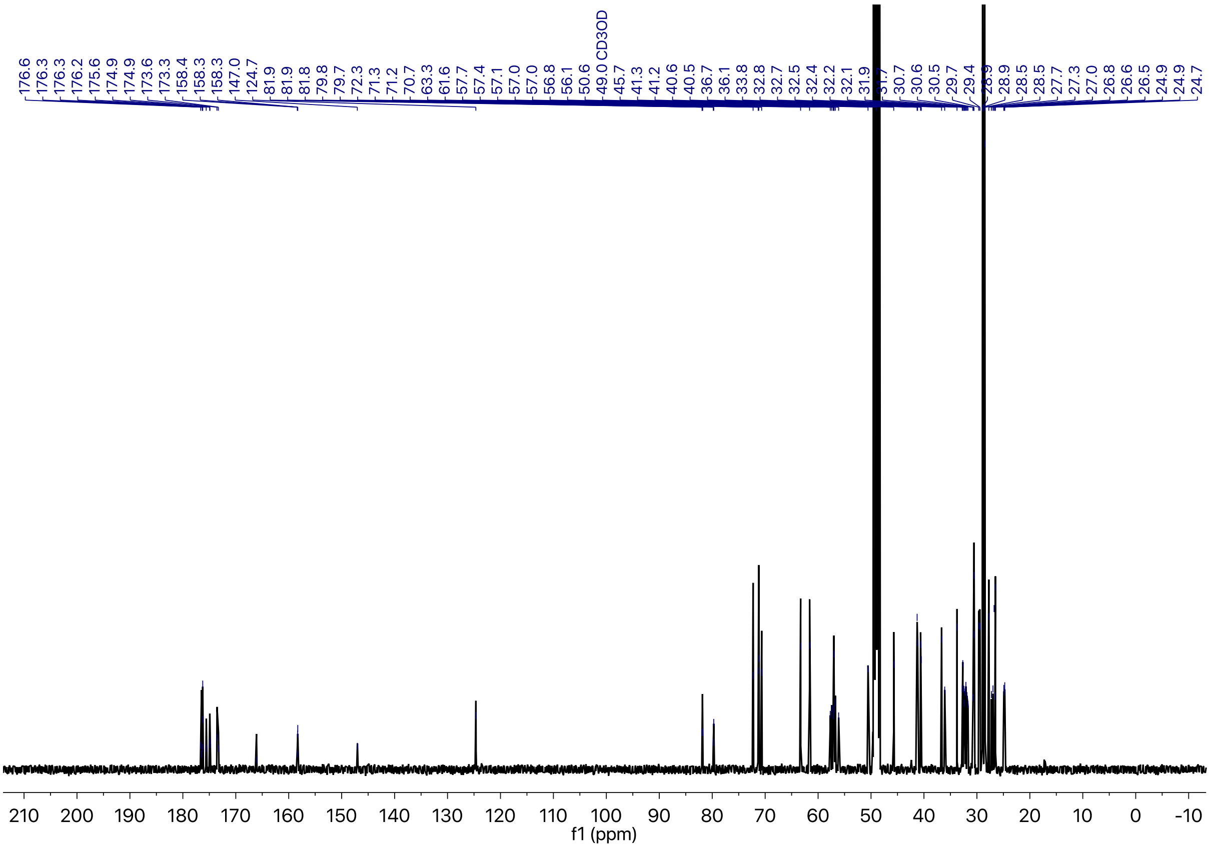

Fig. S71 ^{13}C NMR spectrum (126 MHz) of **94** in CD_3OD.

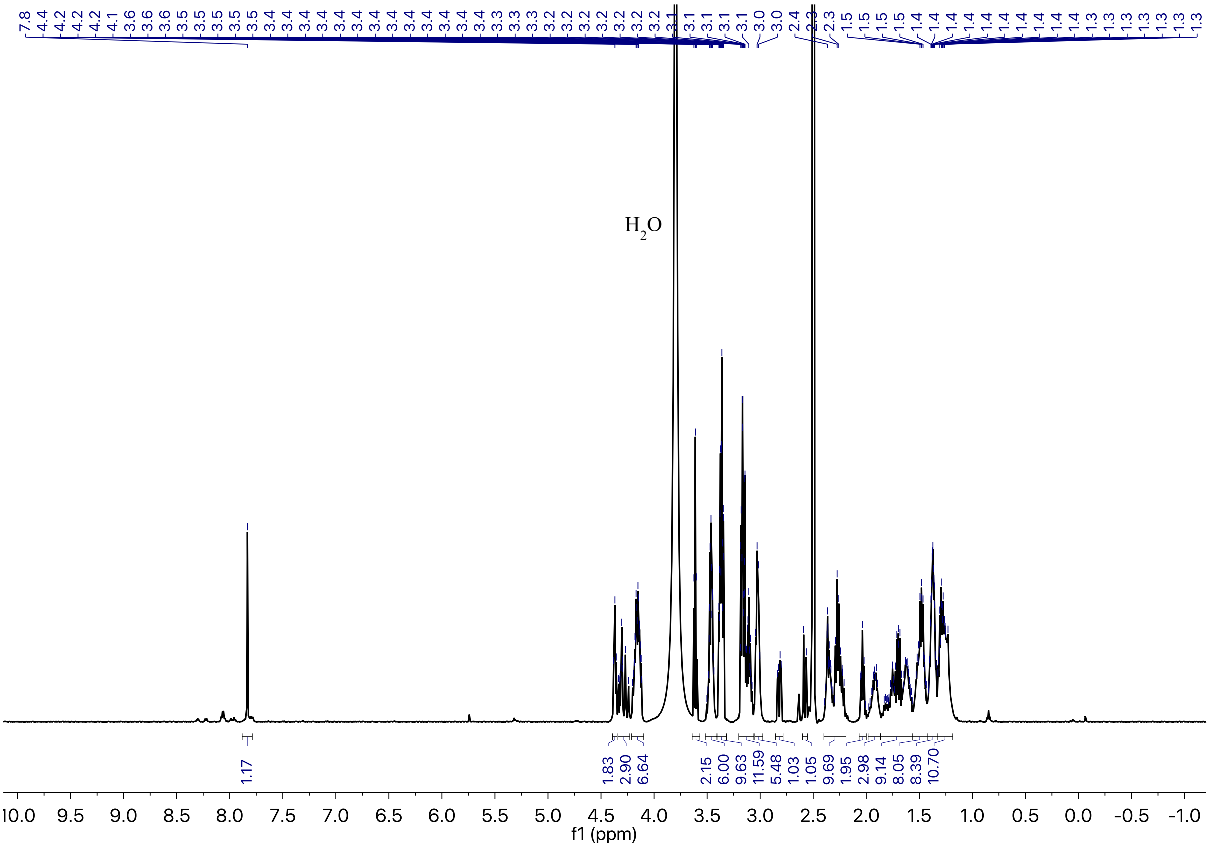

Fig. S72 ^{1}H NMR spectrum (500 MHz) of **9** in DMSO-d_6/D_2O/TFA 300:5:1.

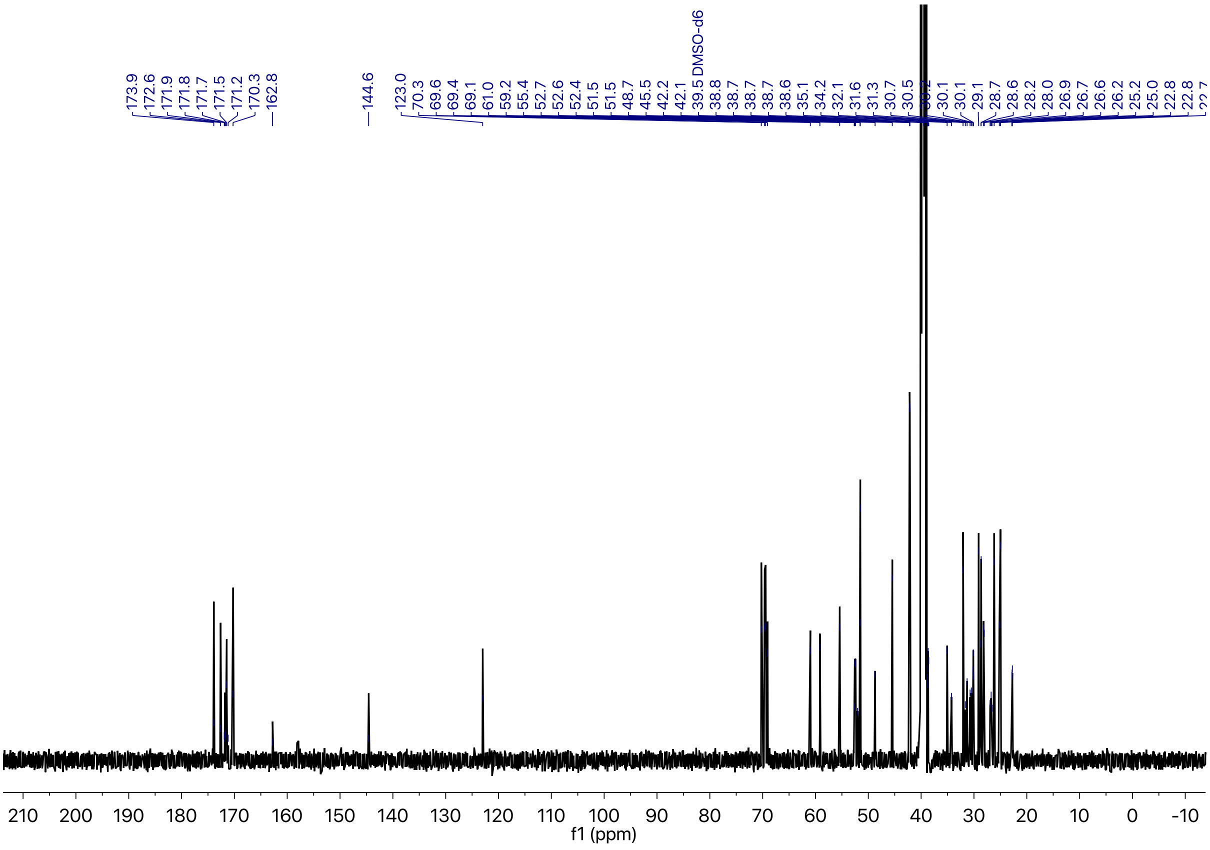

Fig. S73 ^{13}C NMR spectrum (126 MHz) of **9** in DMSO-d_6.

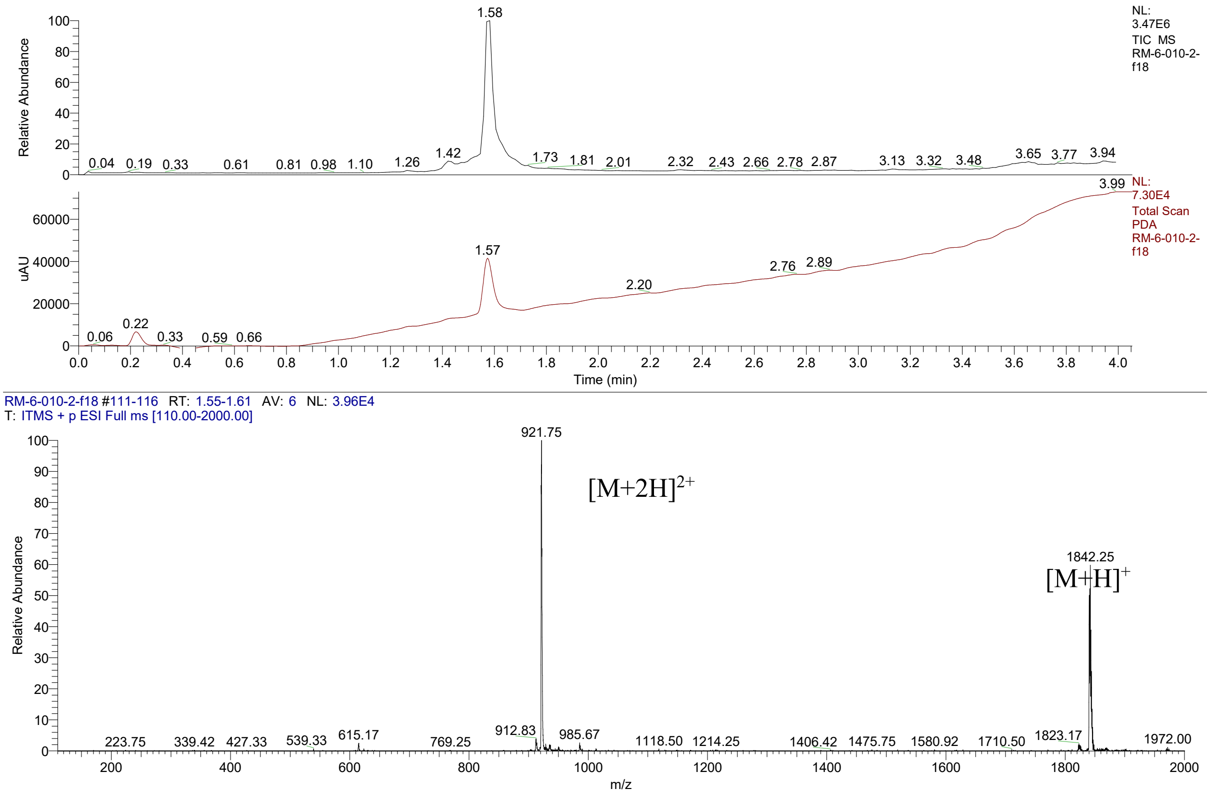

Fig. S74 HPLC-MS profile of **9**.

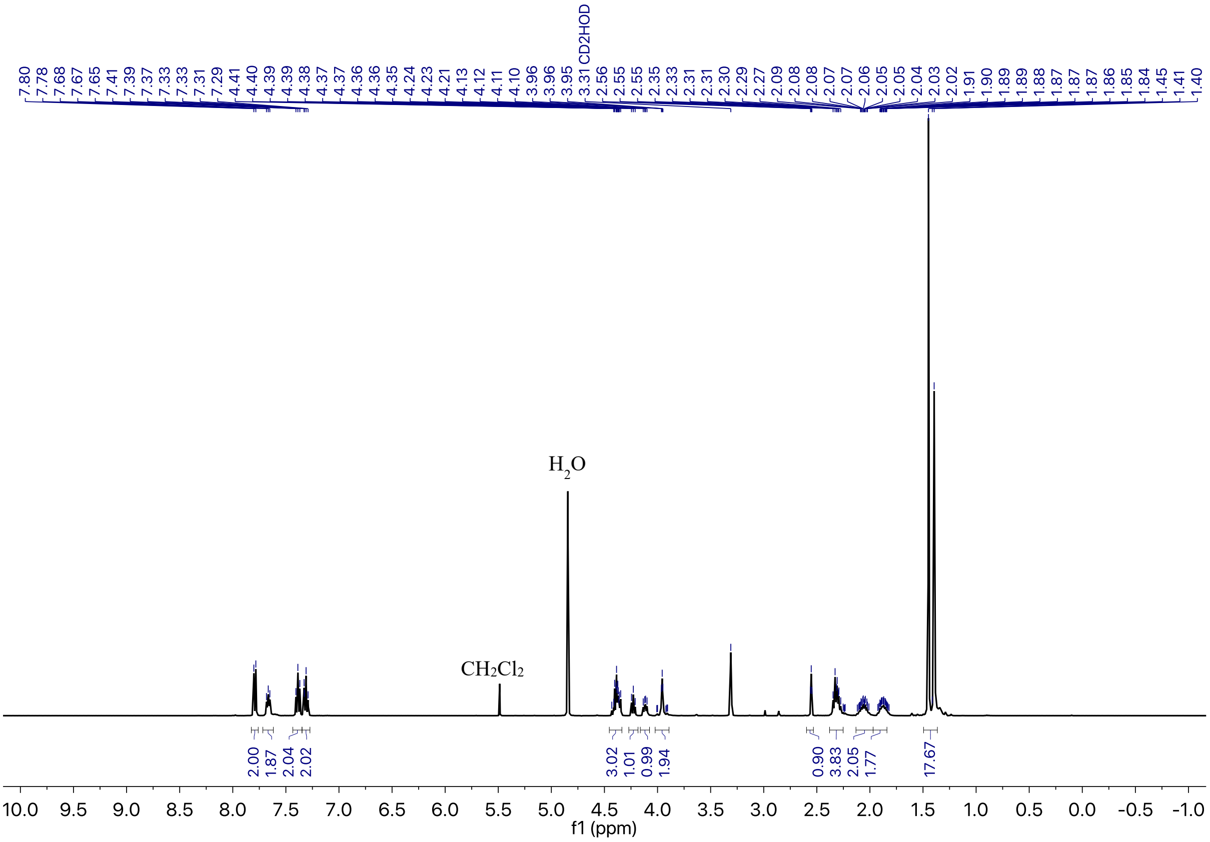

Fig. S75 ^{1}H NMR spectrum (400 MHz) of **97** in CD_3OD.

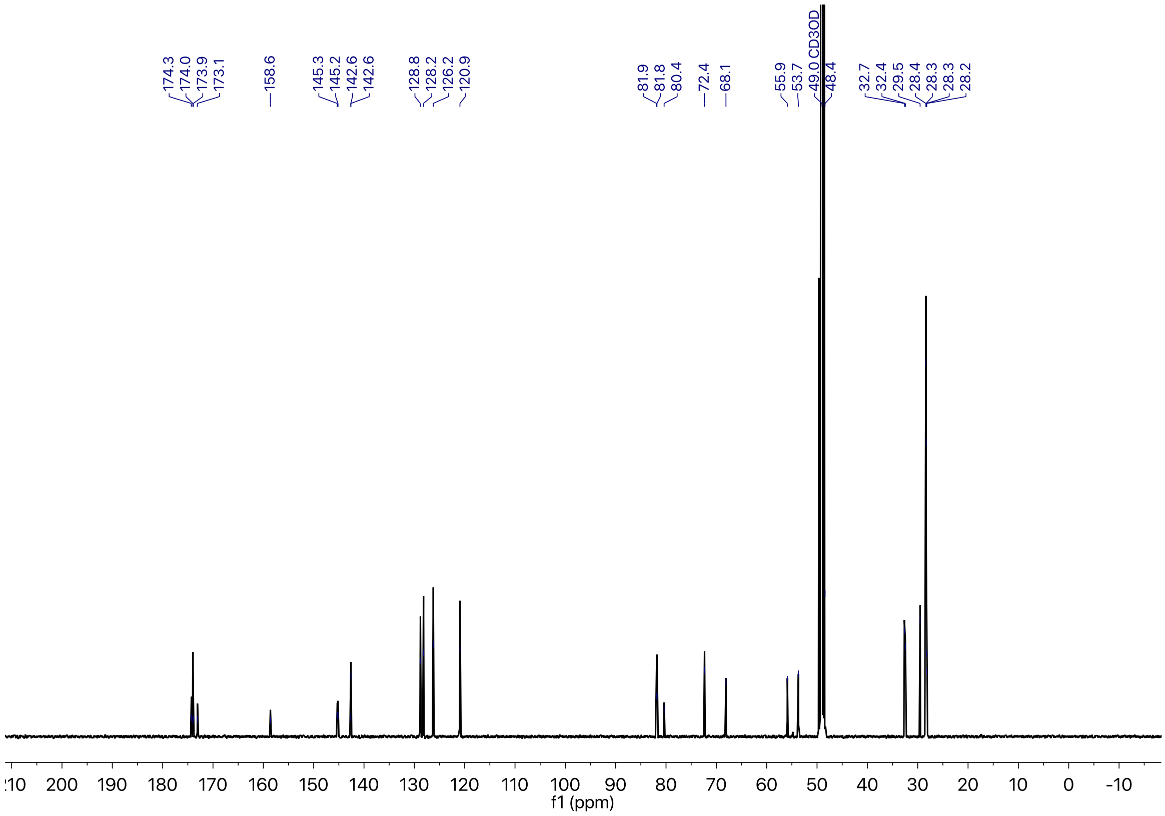

Fig. S76 ^{13}C NMR spectrum (101 MHz) of **97** in CD3OD.

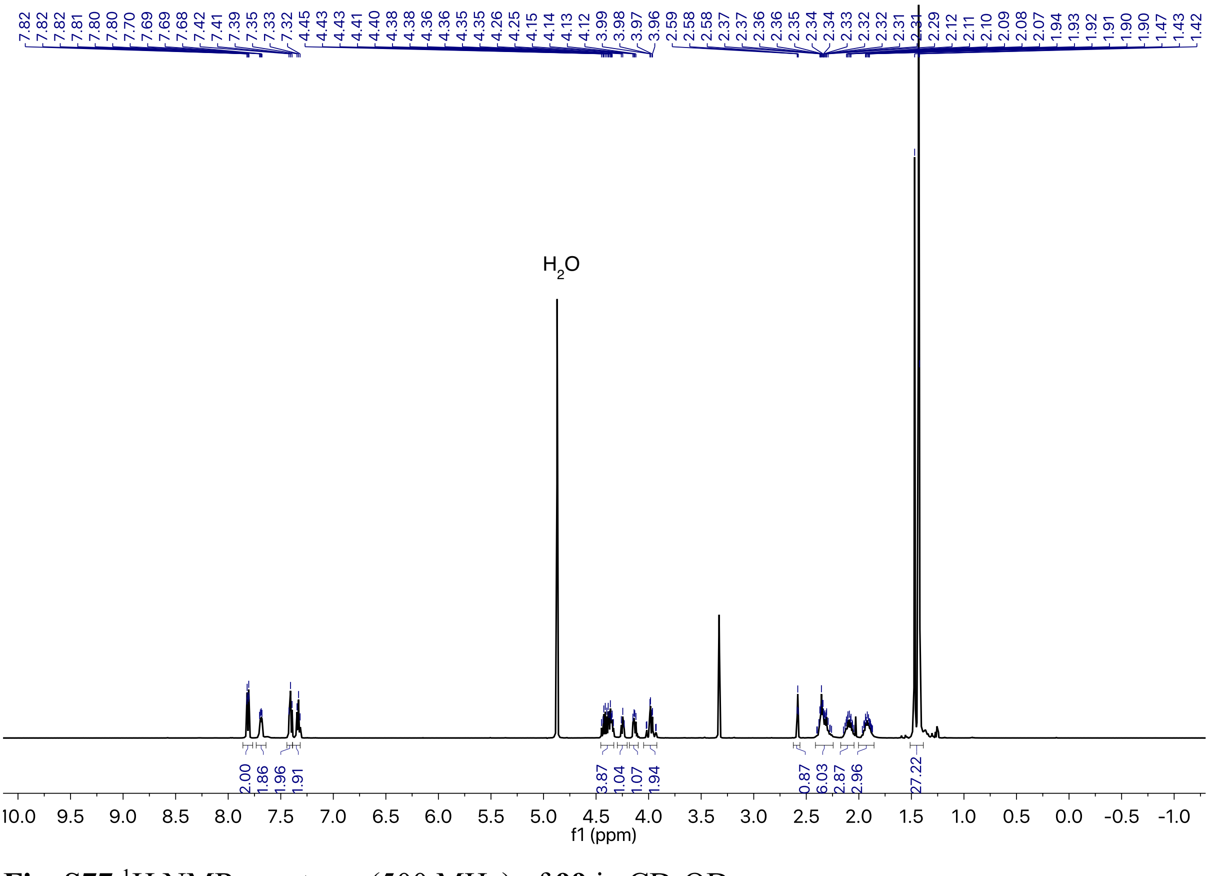

Fig. S77 ^{1}H NMR spectrum (500 MHz) of **99** in CD3OD.

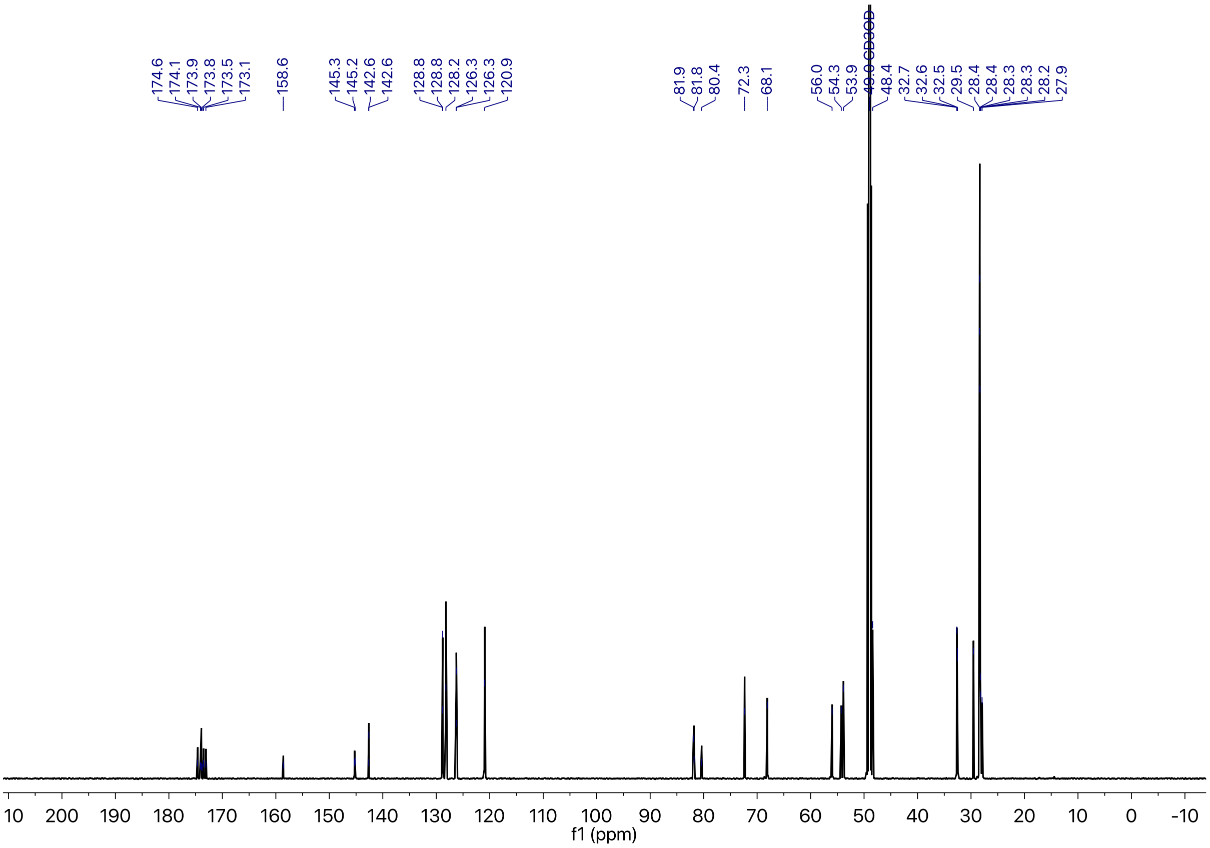

Fig. S78 ^{13}C NMR spectrum (126 MHz) of **99** in CD_3OD.

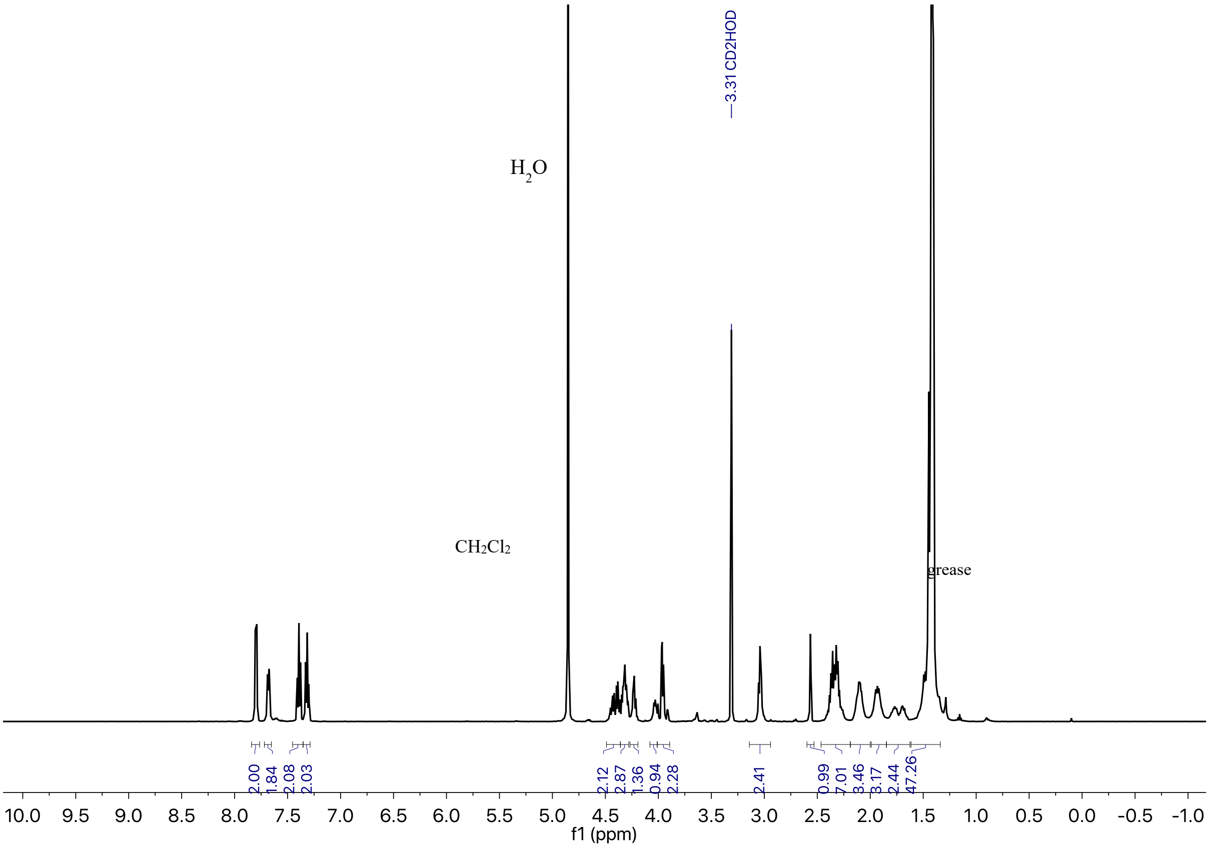

Fig. S79 ^{1}H NMR spectrum (400 MHz) of **101** in CD_3OD.

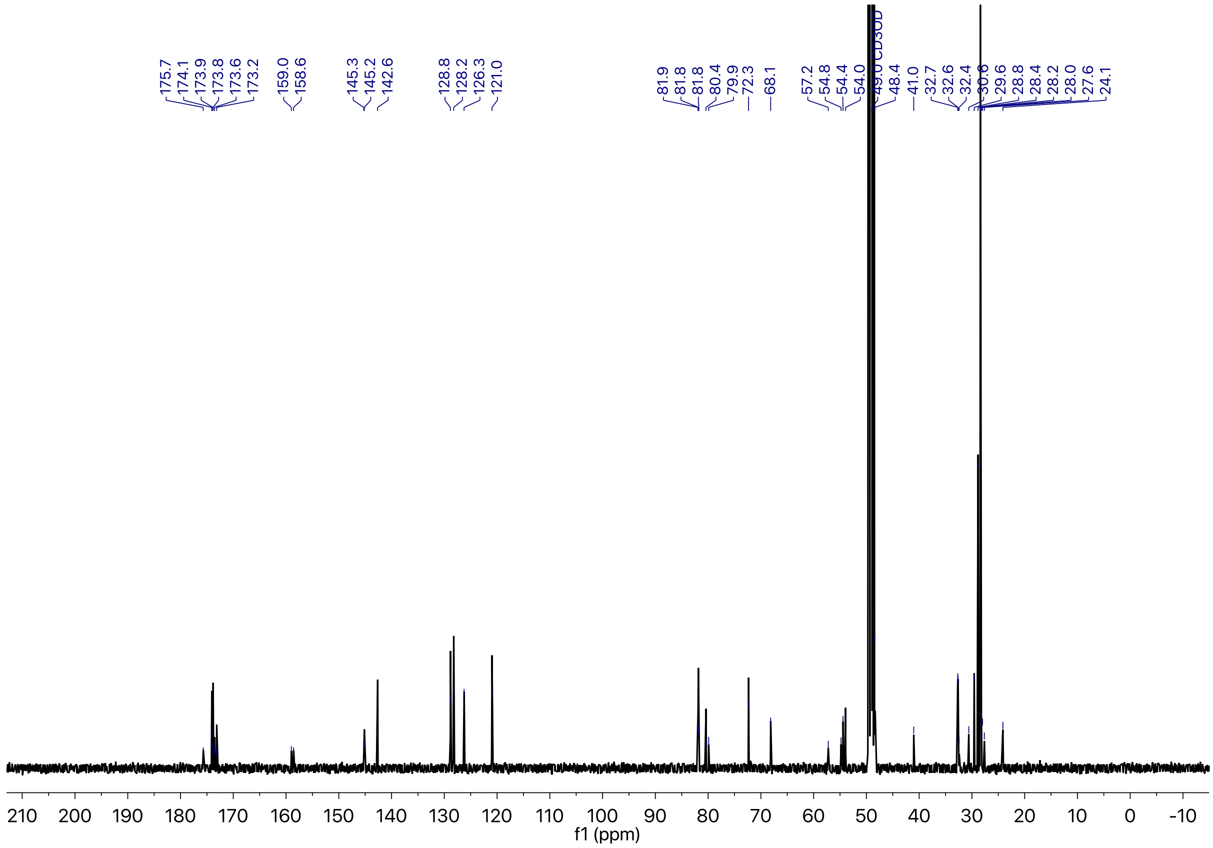

Fig. S80 ^{13}C NMR spectrum (101 MHz) of **101** in CD_3OD.

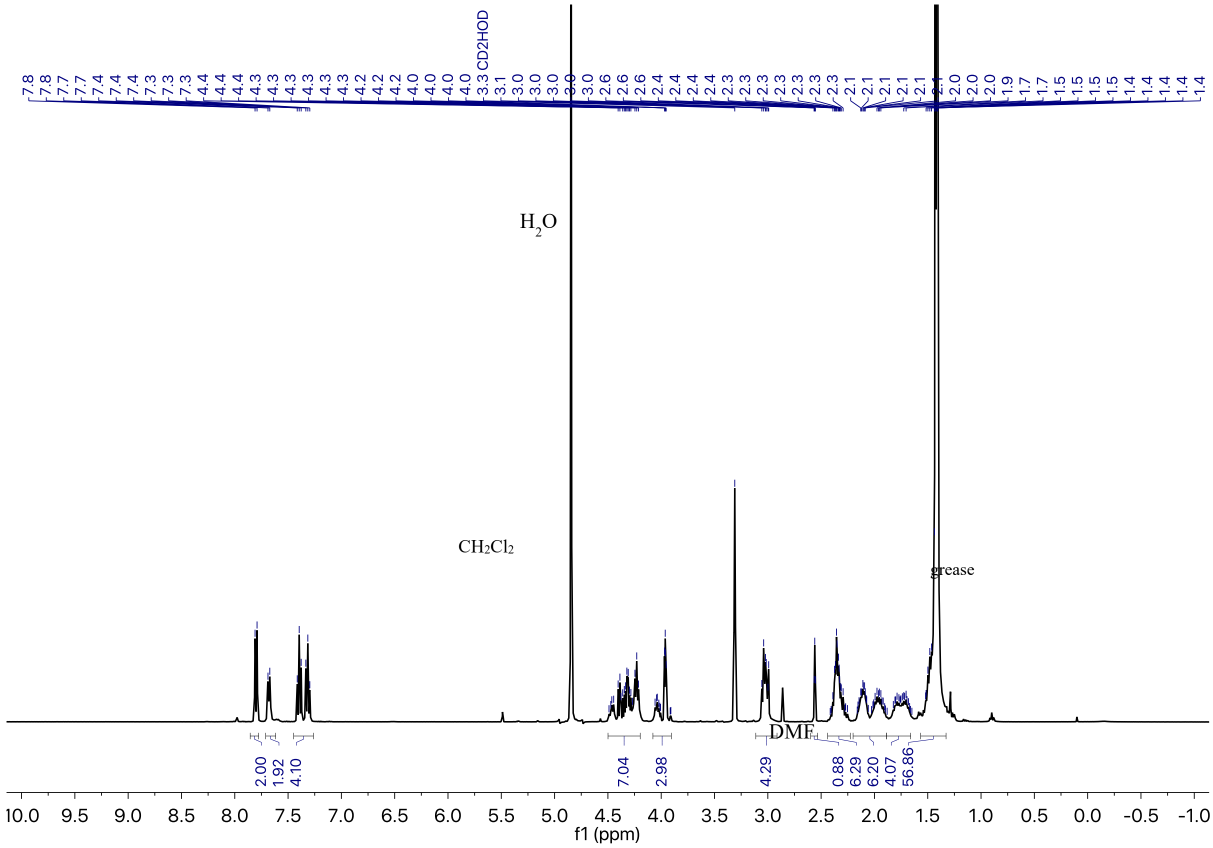

Fig. S81 ^{1}H NMR spectrum (400 MHz) of **103** in CD_3OD.

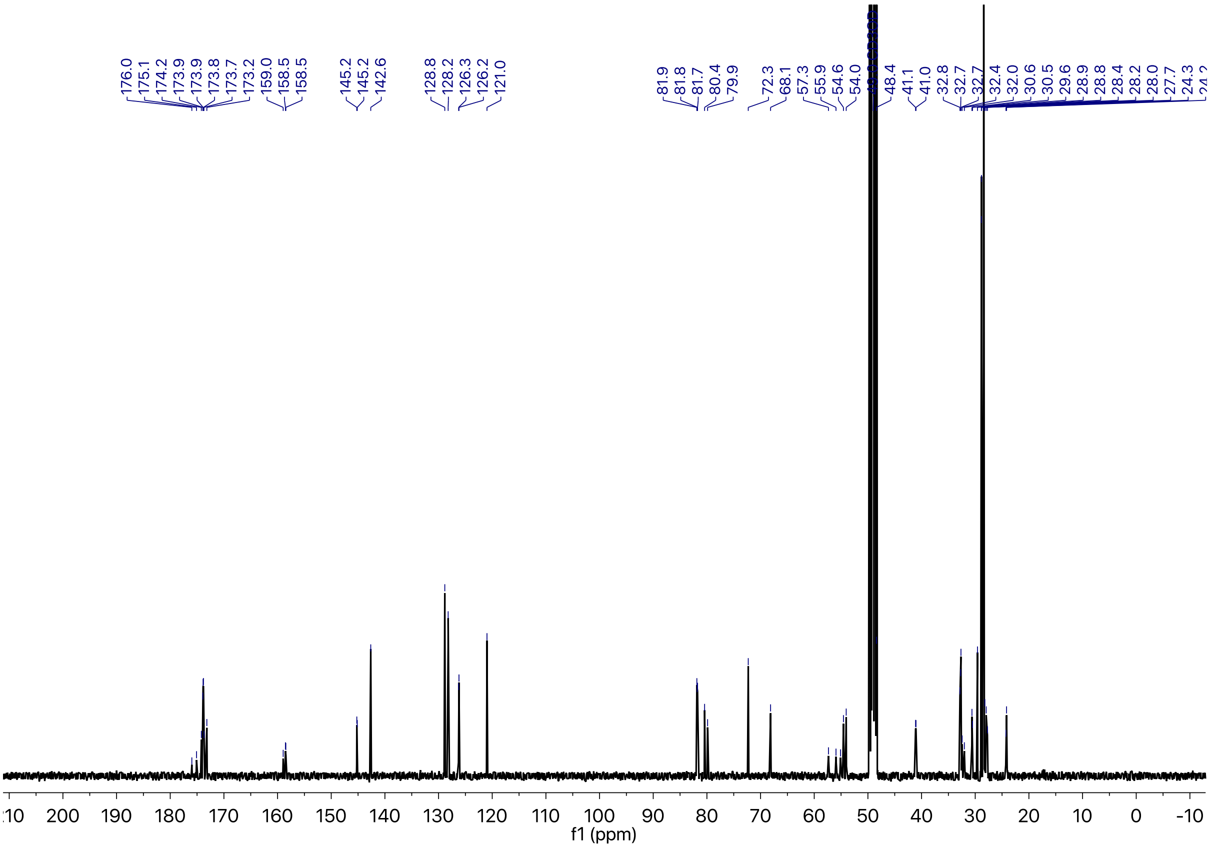

Fig. S82 ^{13}C NMR spectrum (101 MHz) of **103** in CD_3OD.

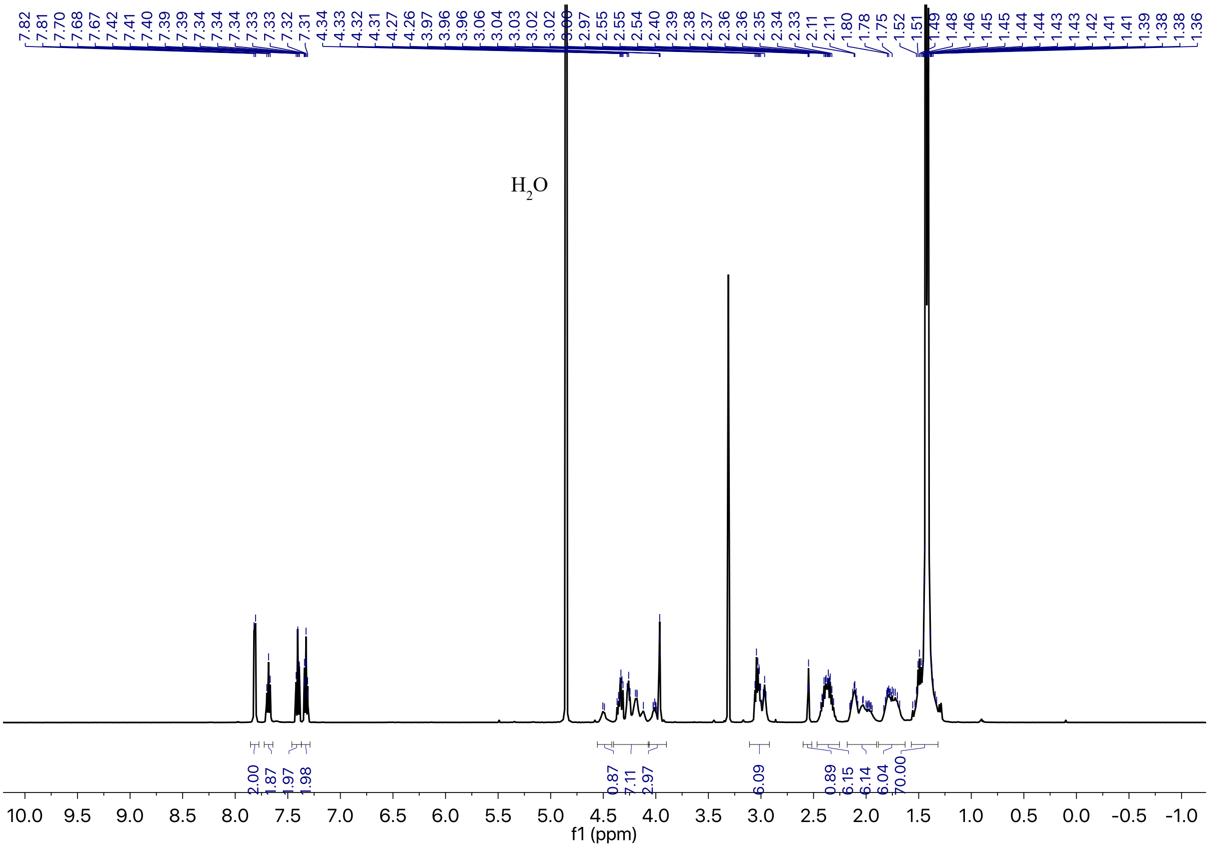

Fig. S83 ^{1}H NMR spectrum (500 MHz) of **105** in CD_3OD.

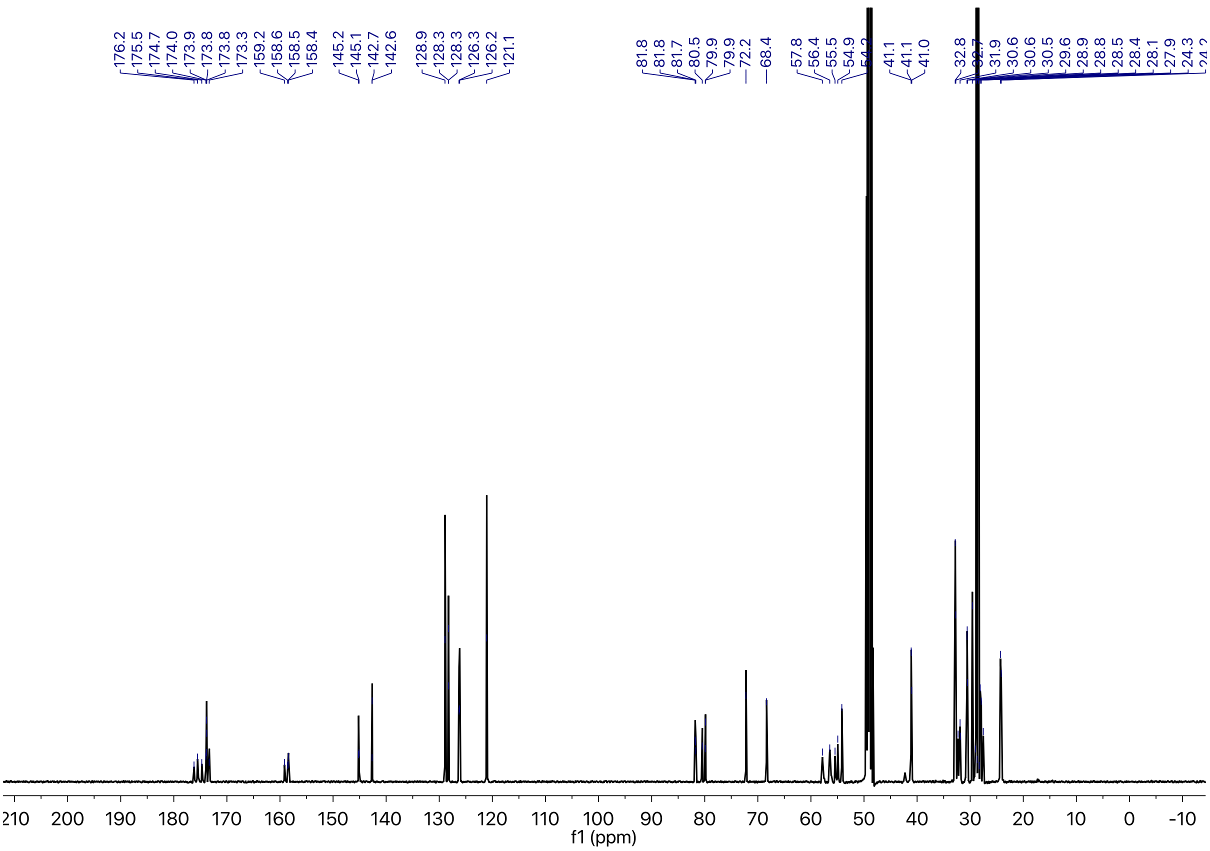

Fig. S84 ^{13}C NMR spectrum (126 MHz) of **105** in CD_3OD.

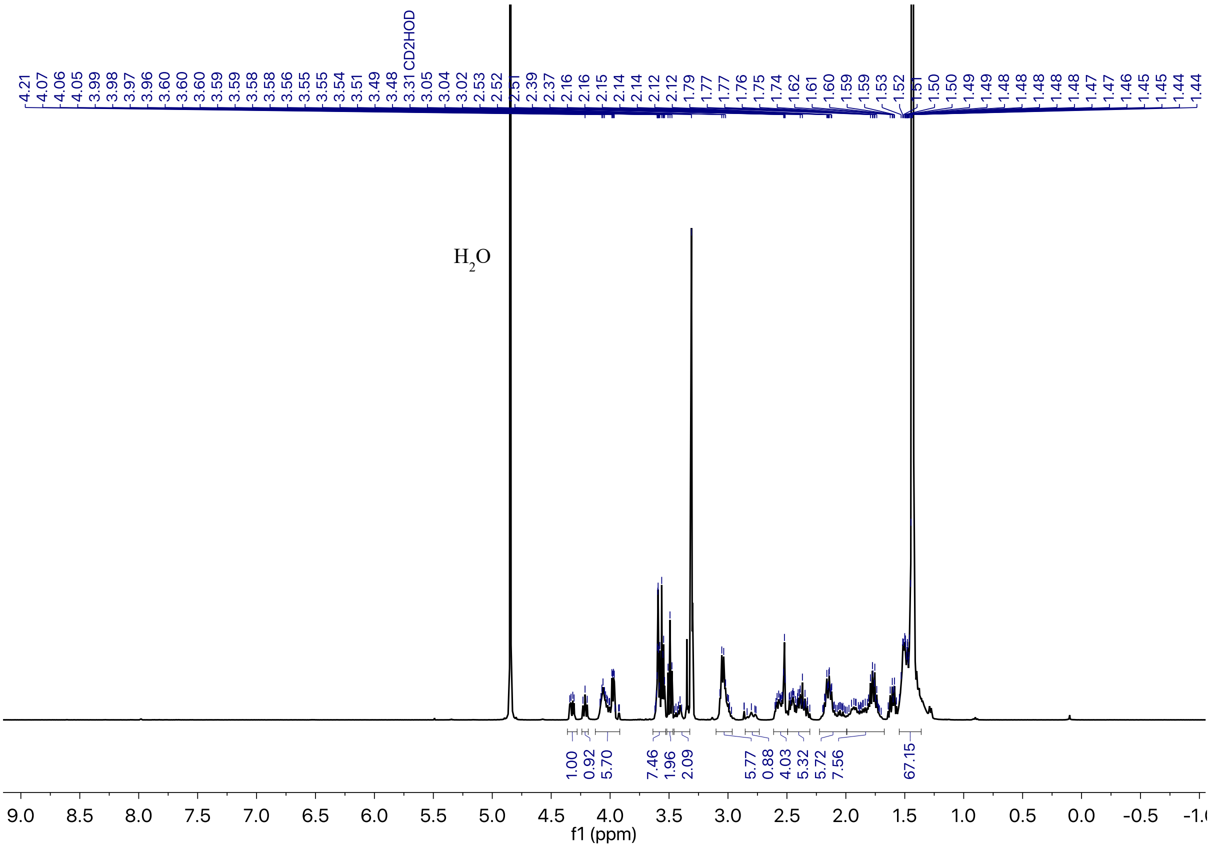

Fig. S85 ^{1}H NMR spectrum (400 MHz) of **107** in CD_3OD.

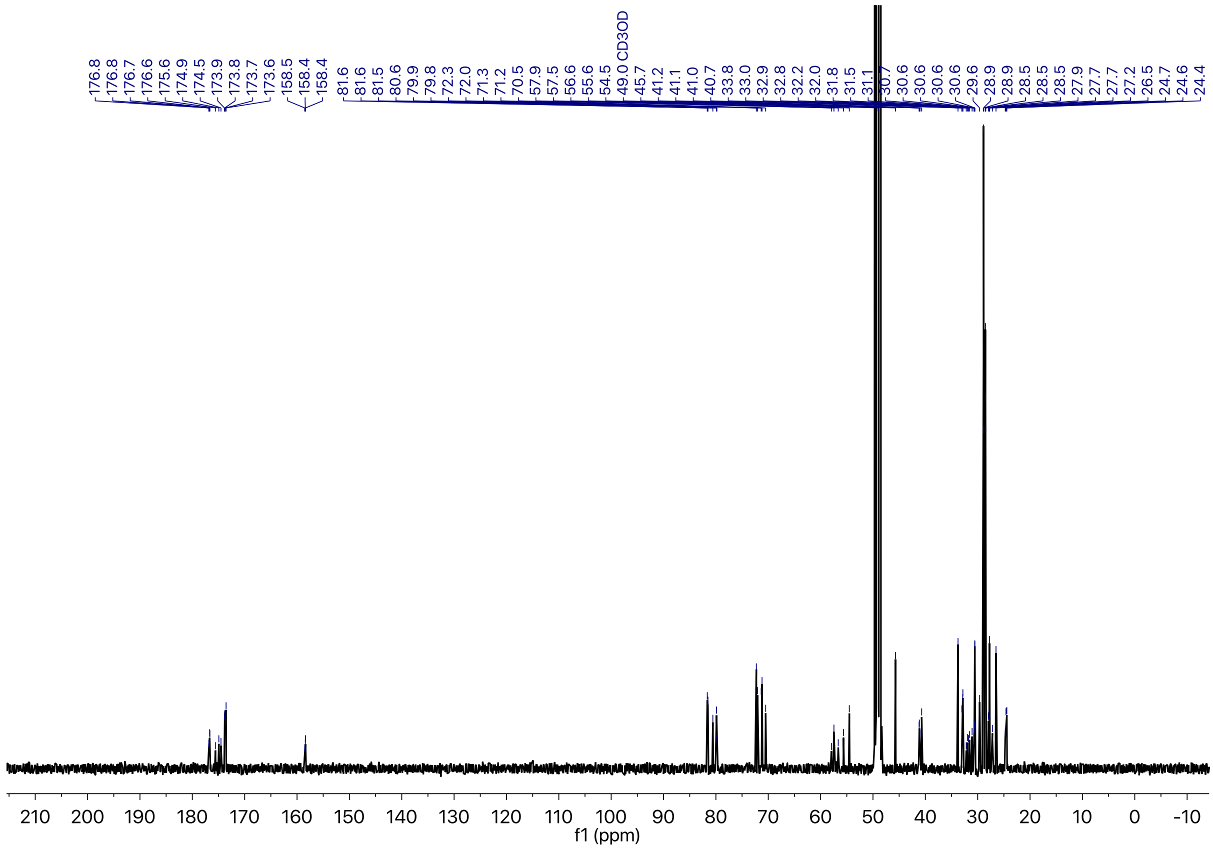

Fig. S86 ^{13}C NMR spectrum (400 MHz) of **107** in CD_3OD.

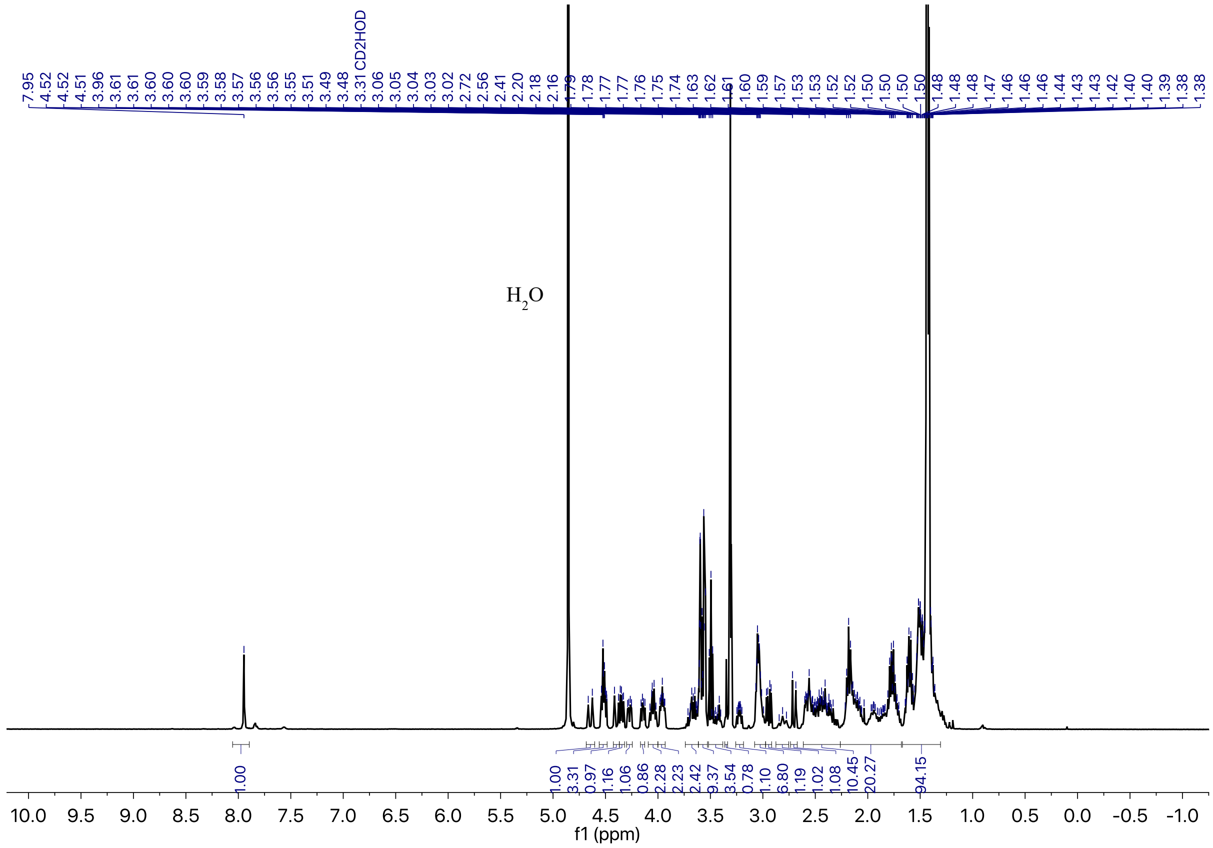

Fig. S87 ^{1}H NMR spectrum (400 MHz) of **108** in CD_3OD.

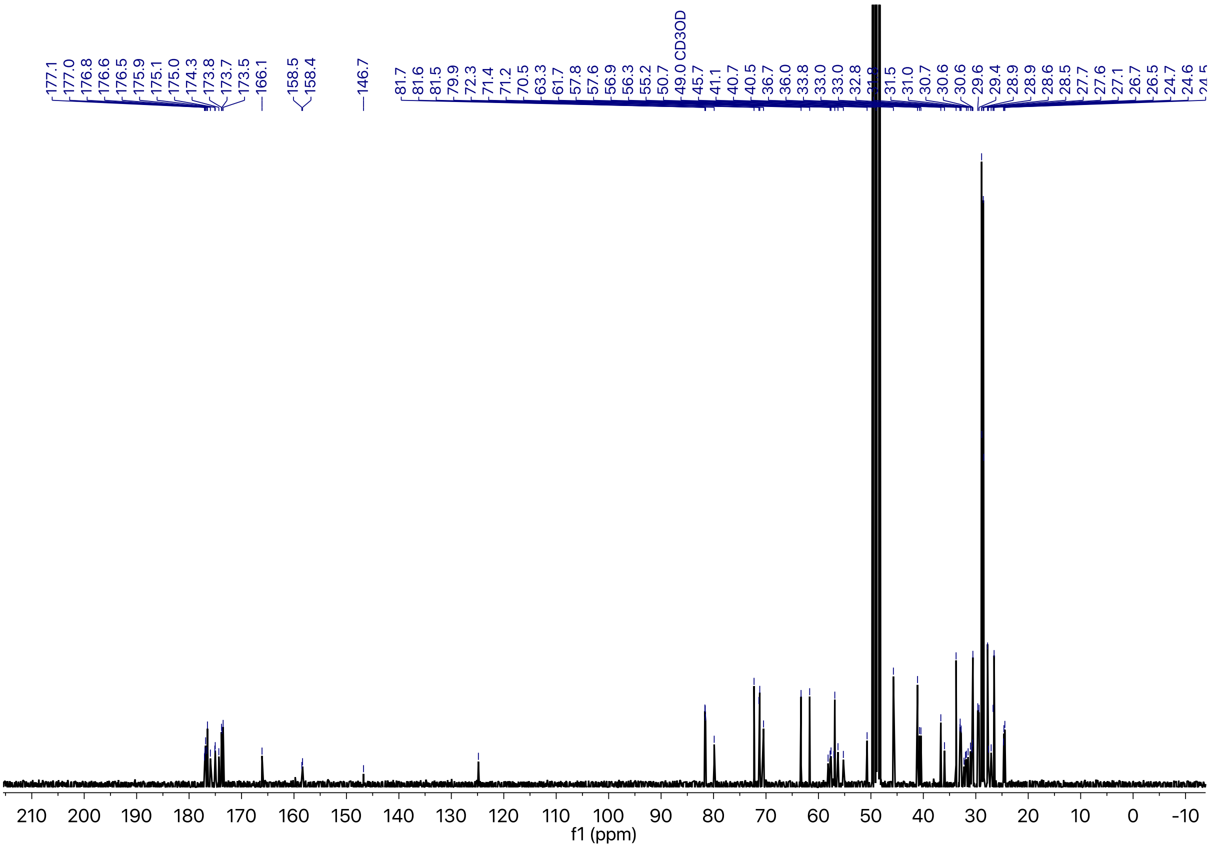

Fig. S88 ^{13}C NMR spectrum (101 MHz) of **108** in CD_3OD.

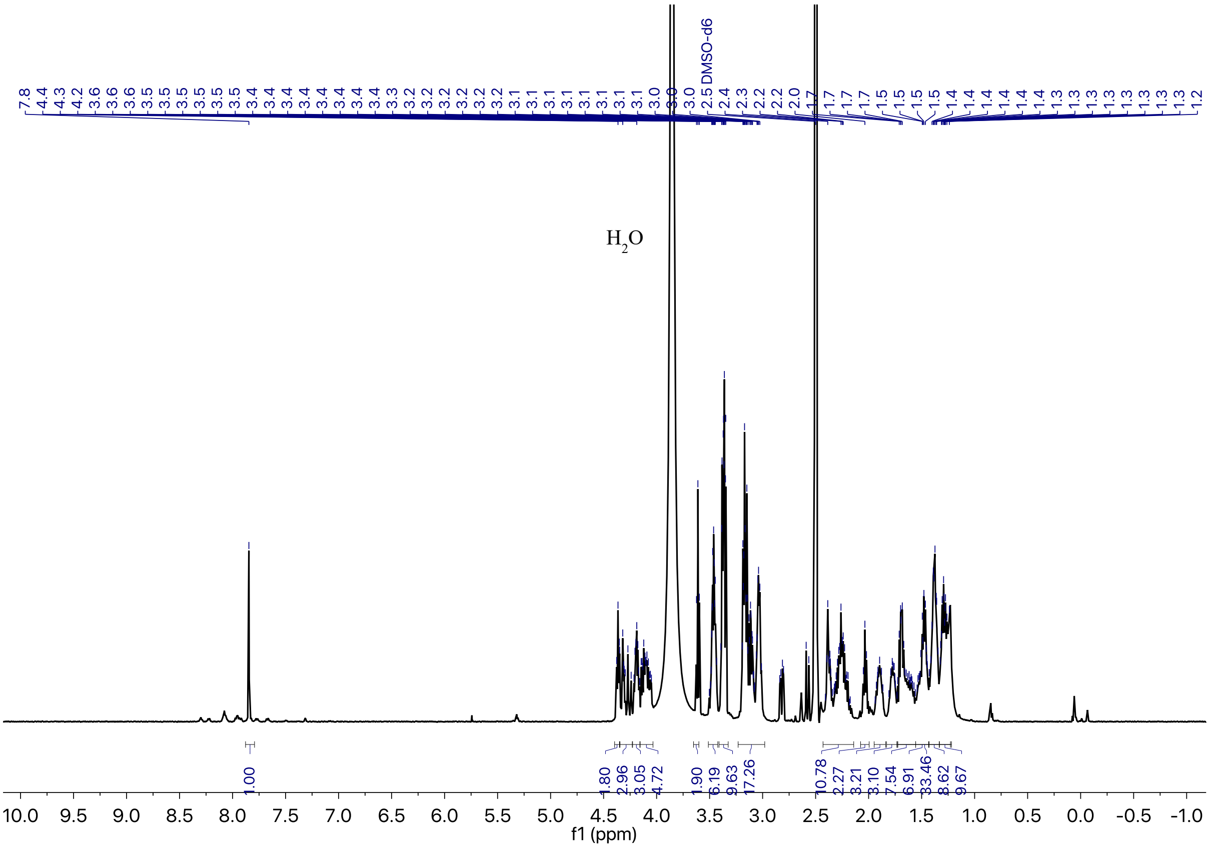

Fig. S89 ^{1}H NMR spectrum (500 MHz) of **10** in DMSO-d_6/D_2O 60:1.

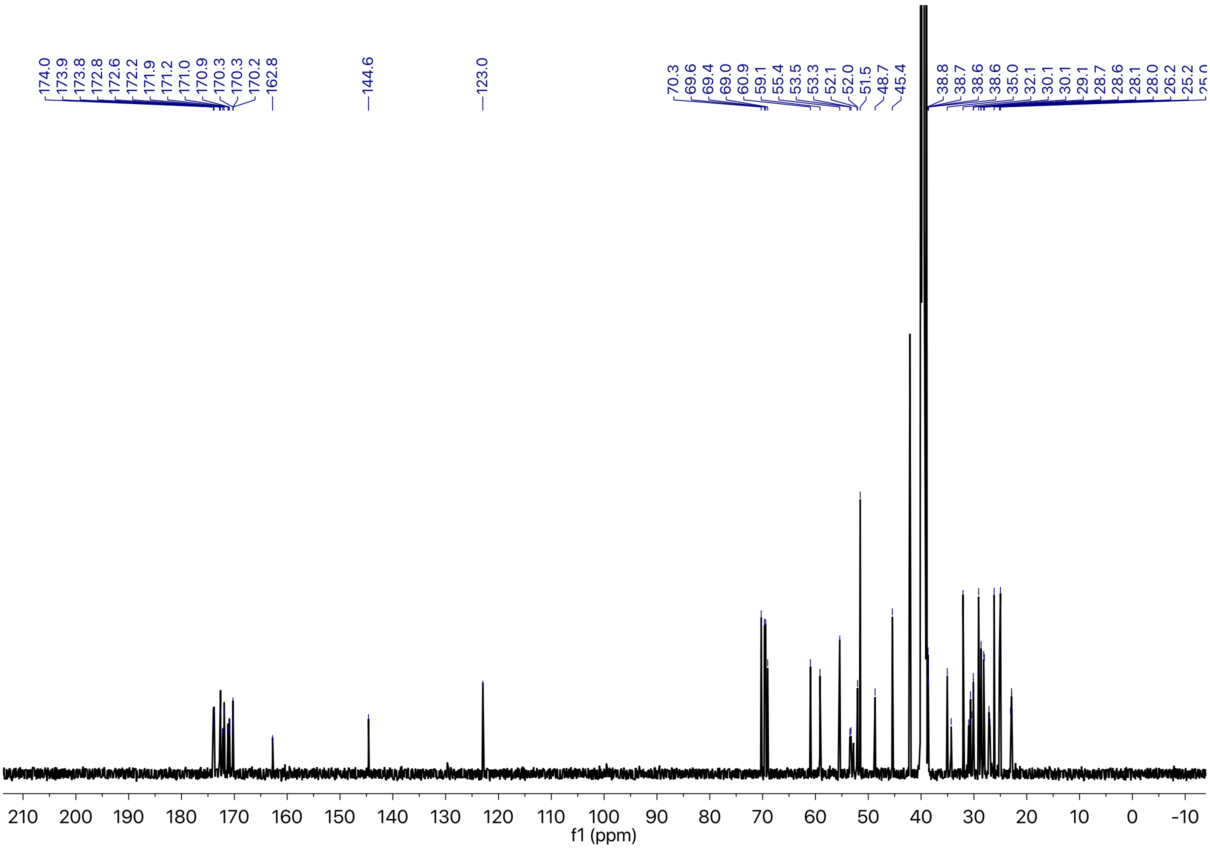

Fig. S90 ^{13}C NMR spectrum (126 MHz) of **10** in DMSO-d_6.

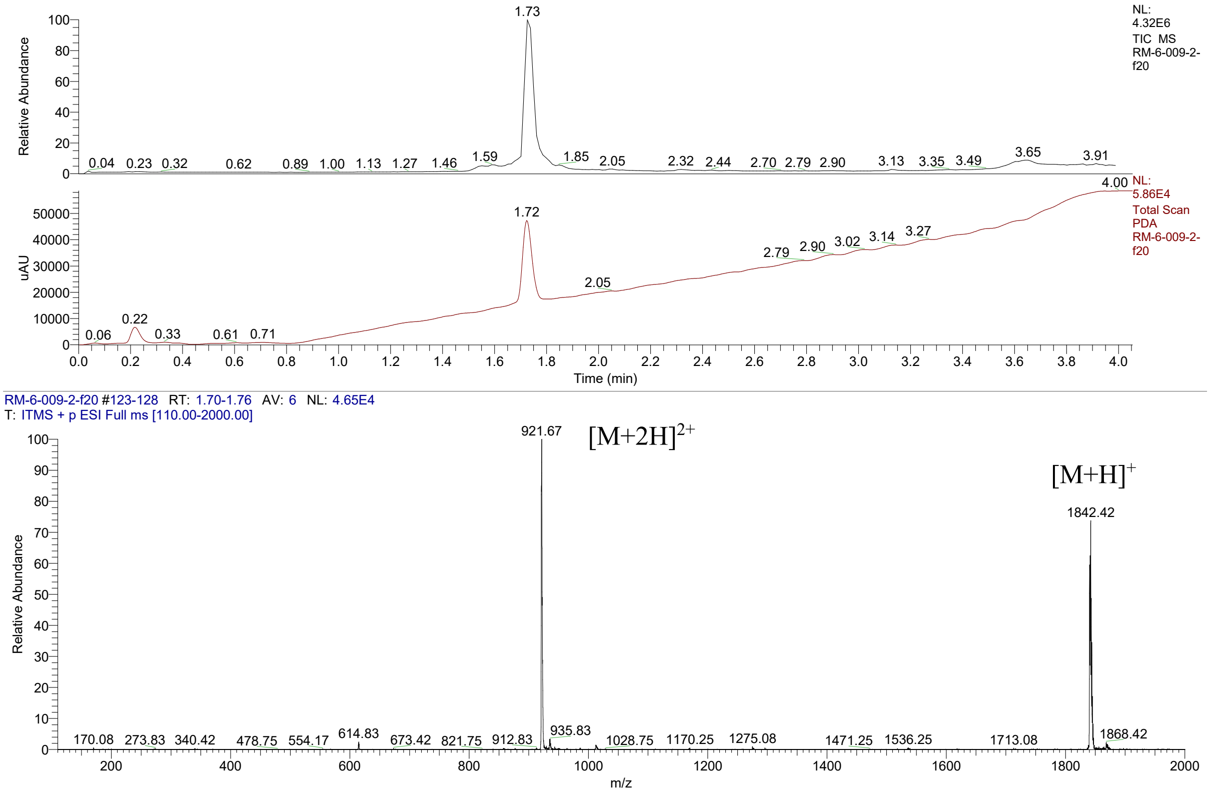

Fig. S91 HPLC-MS profile of **10**.

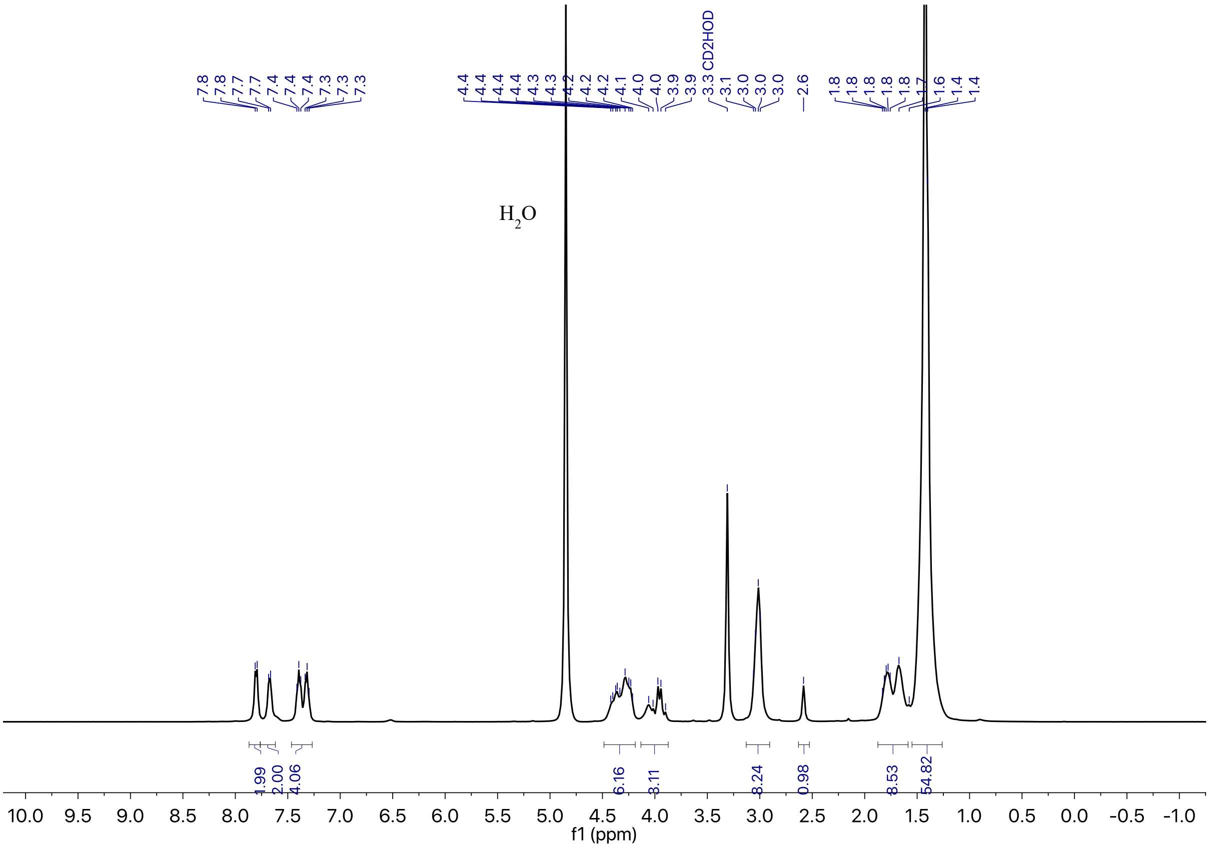

Fig. S92 ^{1}H NMR spectrum (400 MHz) of **110** in CD_3OD.

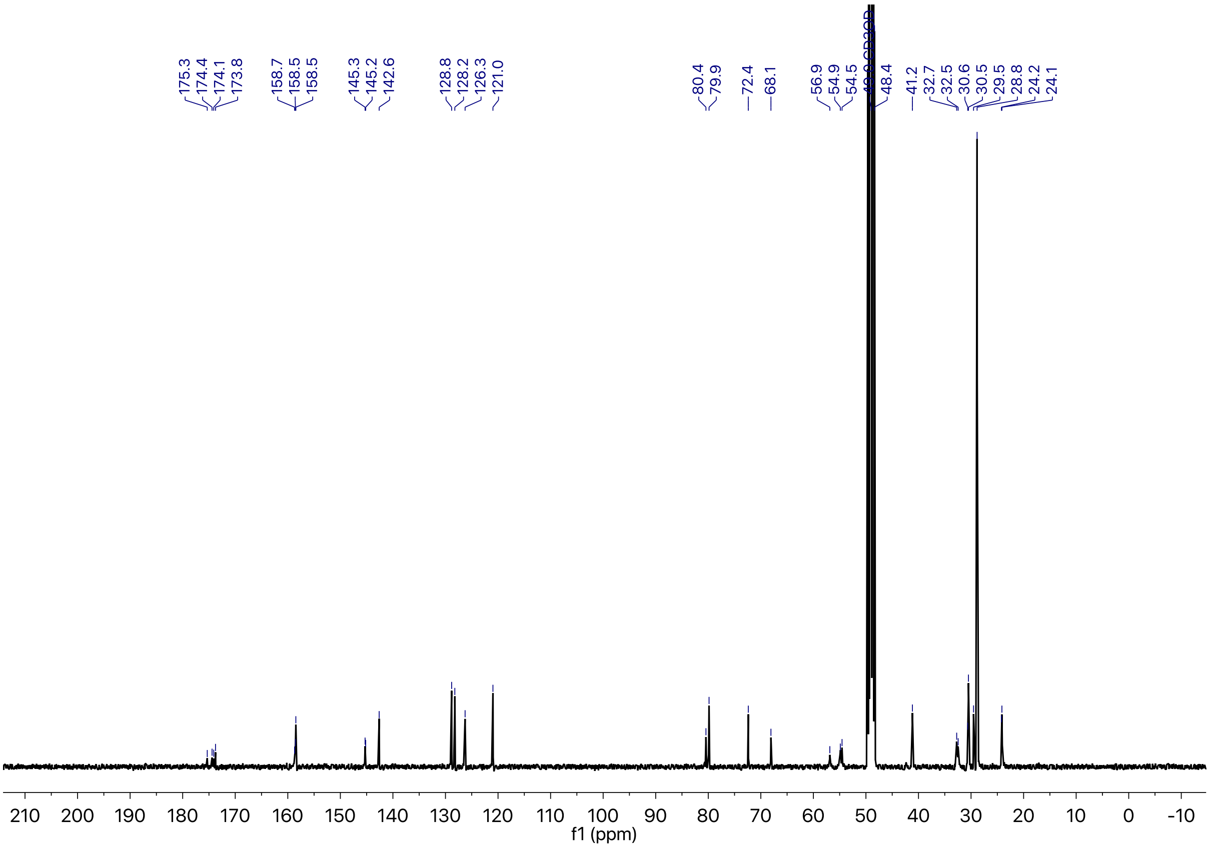

Fig. S93 ^{13}C NMR spectrum (400 MHz) of **110** in CD_3OD.

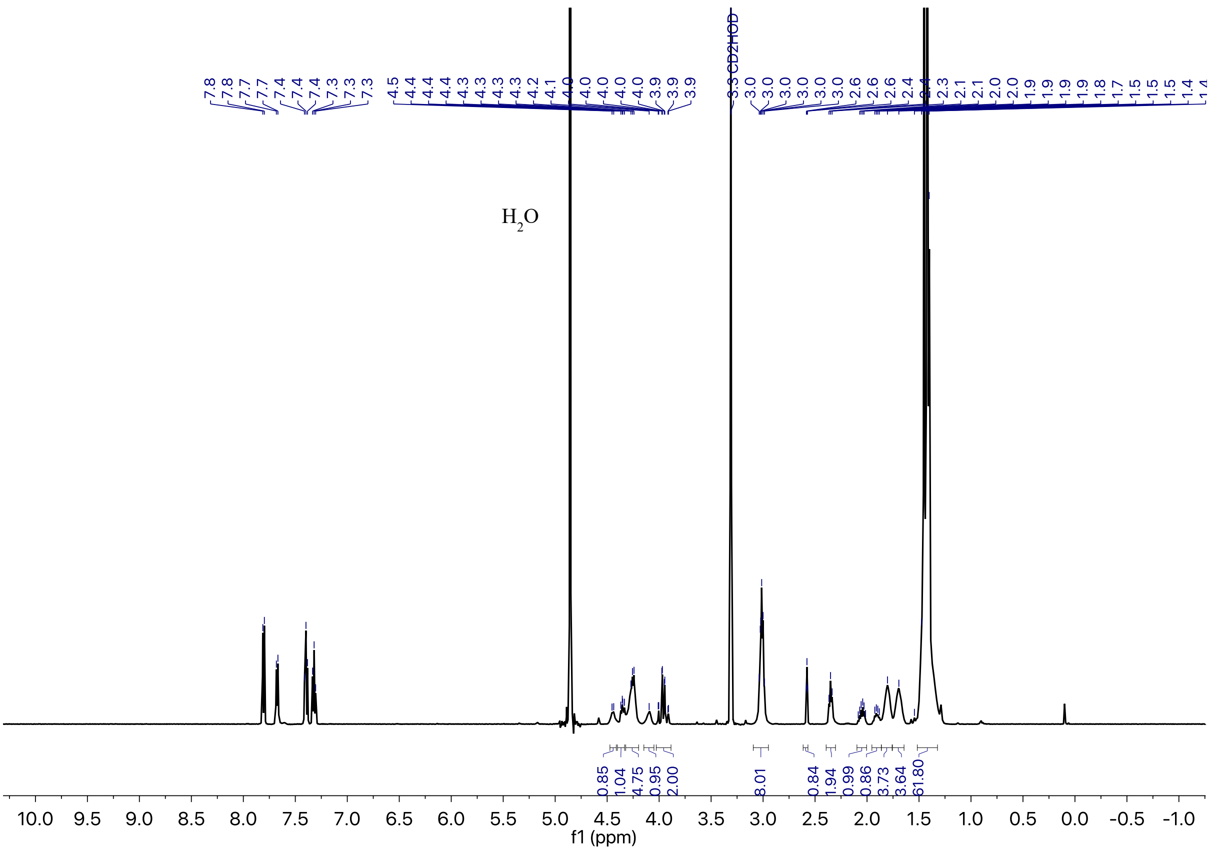

Fig. S94 ^{1}H NMR spectrum (500 MHz) of **112** in CD_3OD.

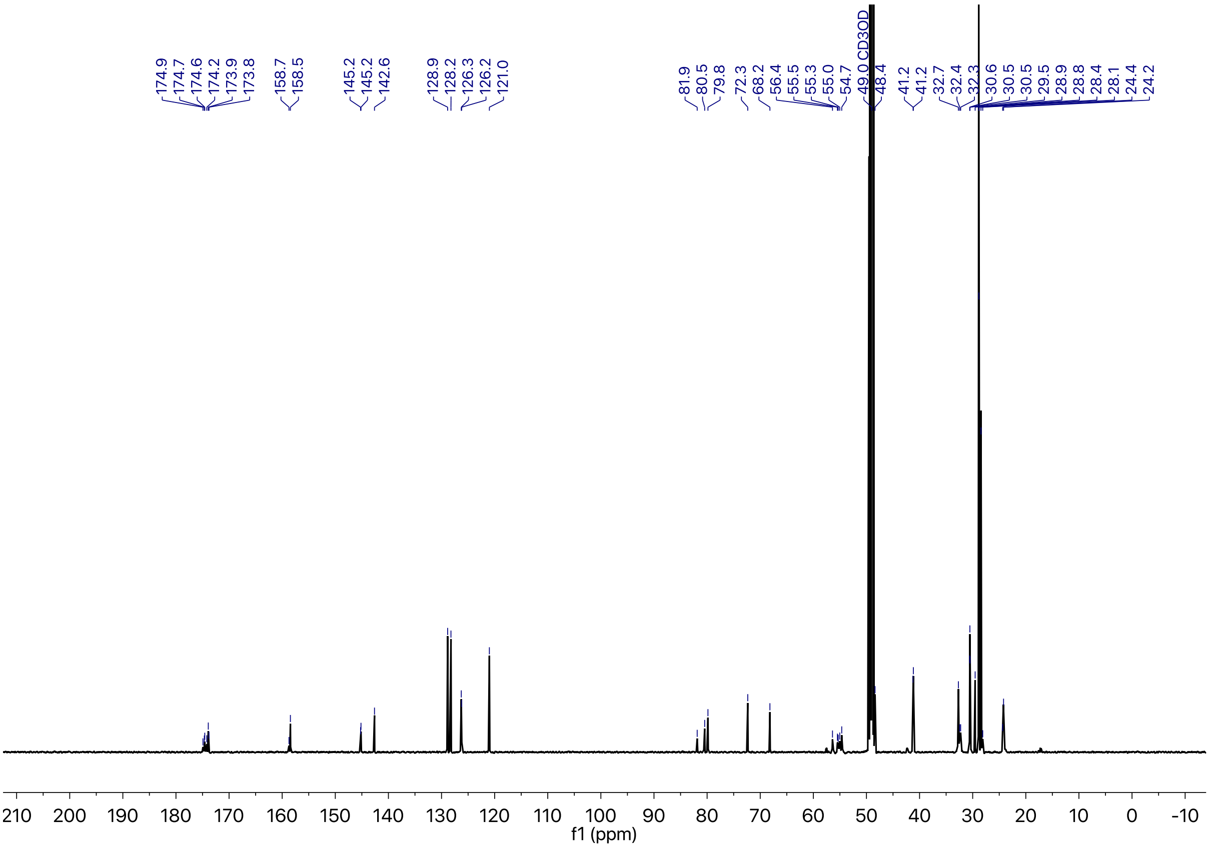

Fig. S95 ^{13}C NMR spectrum (126 MHz) of **112** in CD_3OD.

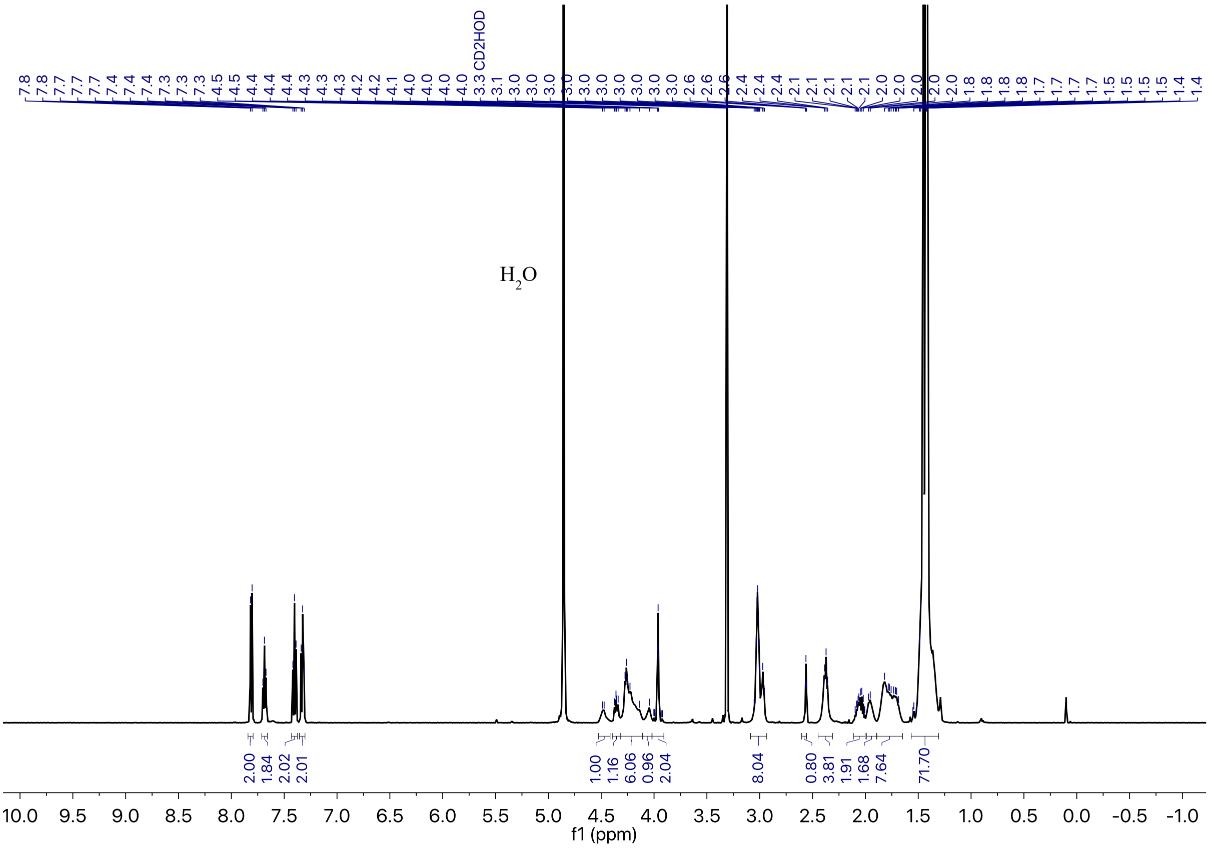

Fig. S96 ^{1}H NMR spectrum (500 MHz) of **114** in CD_3OD.

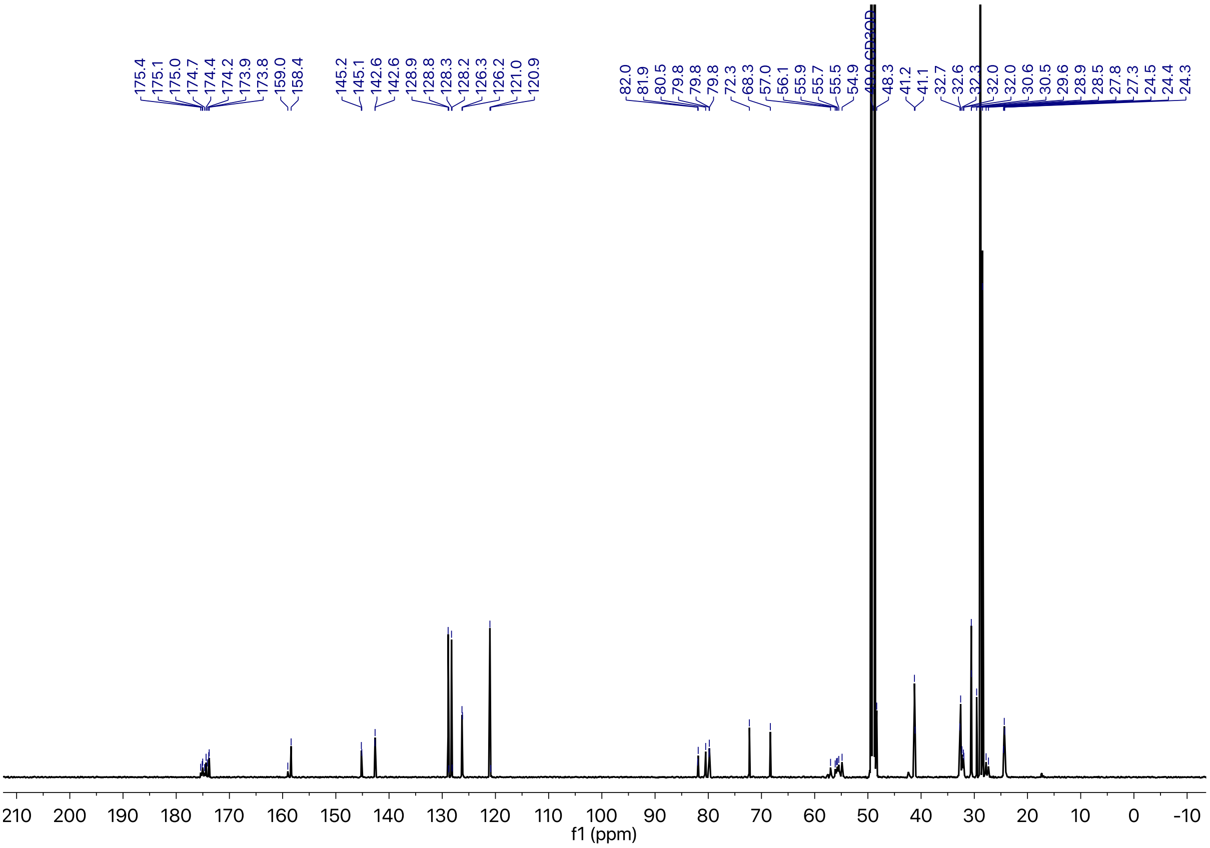

Fig. S97 ^{13}C NMR spectrum (126 MHz) of **114** in CD_3OD.

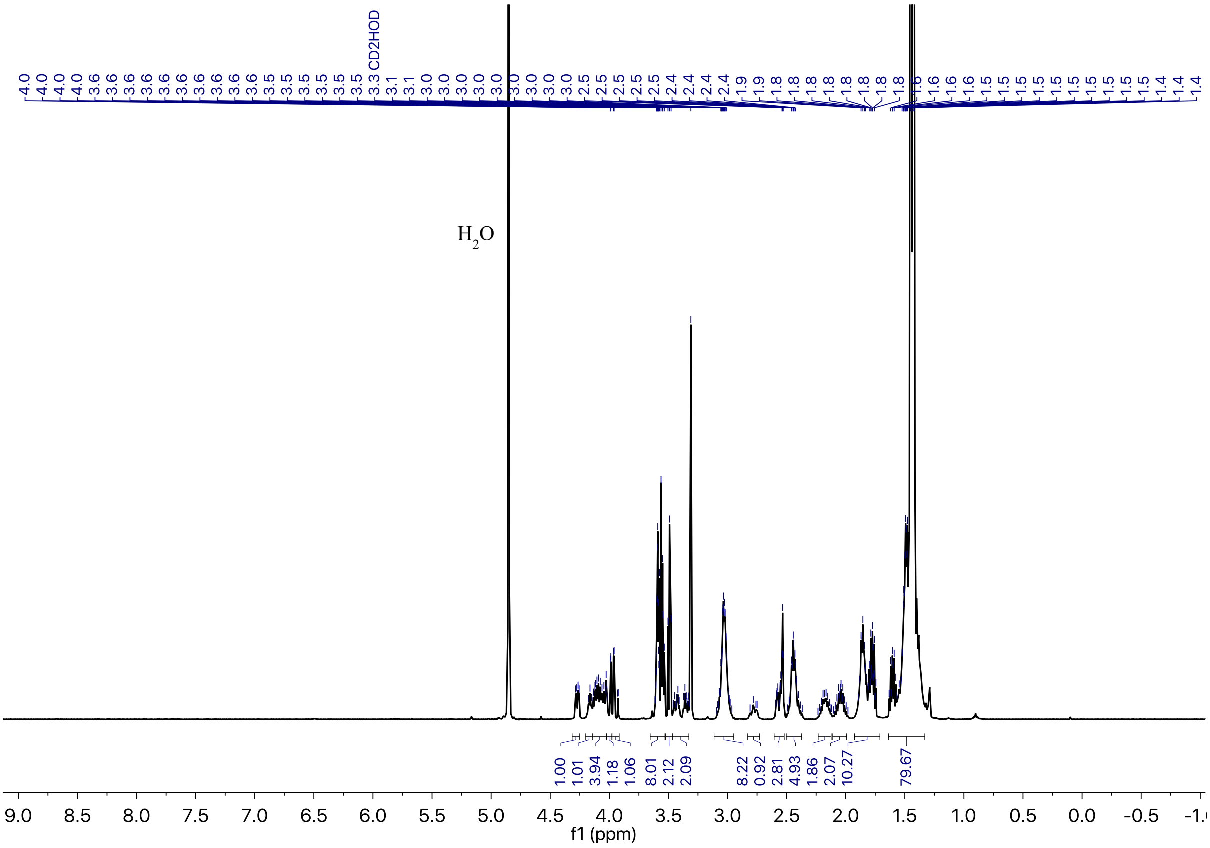

Fig. S98 ^{1}H NMR spectrum (500 MHz) of **116** in CD_3OD.

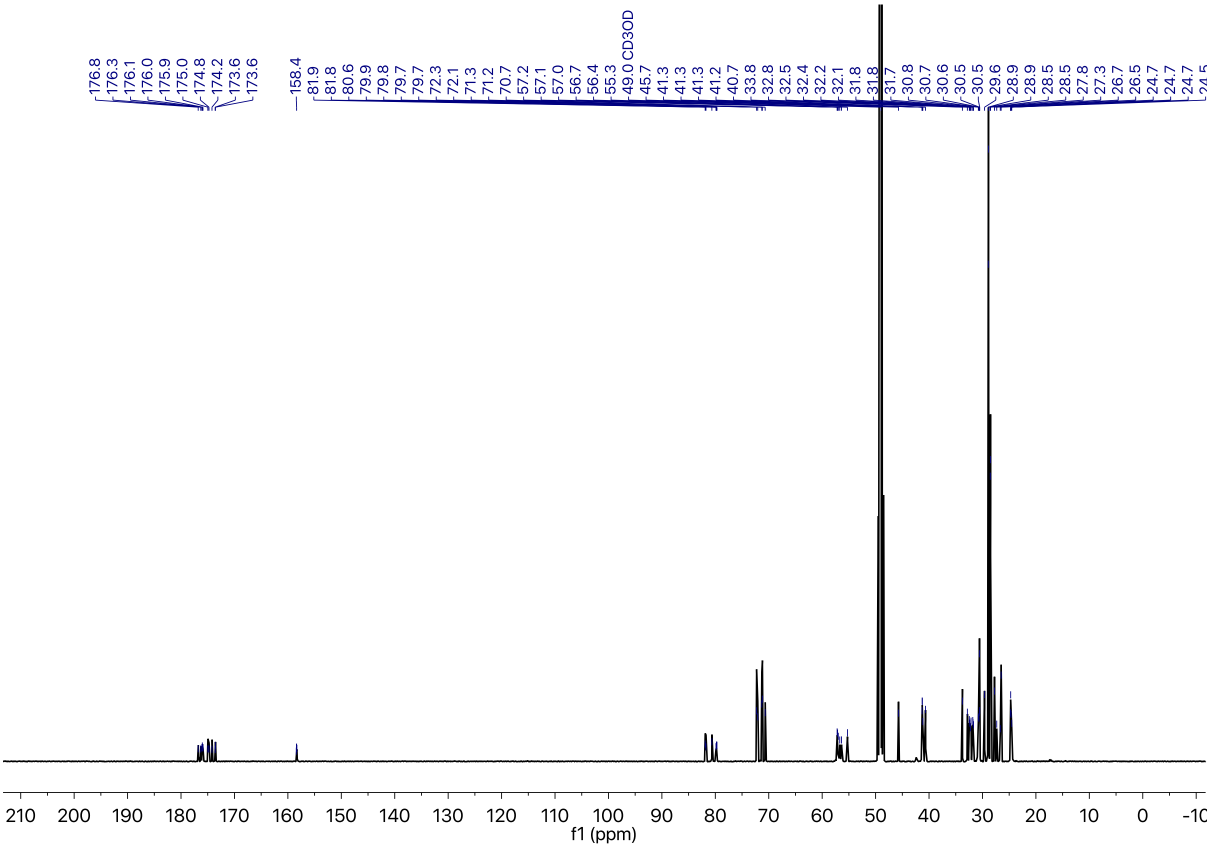

Fig. S99 ^{13}C NMR spectrum (126 MHz) of **116** in CD_3OD.

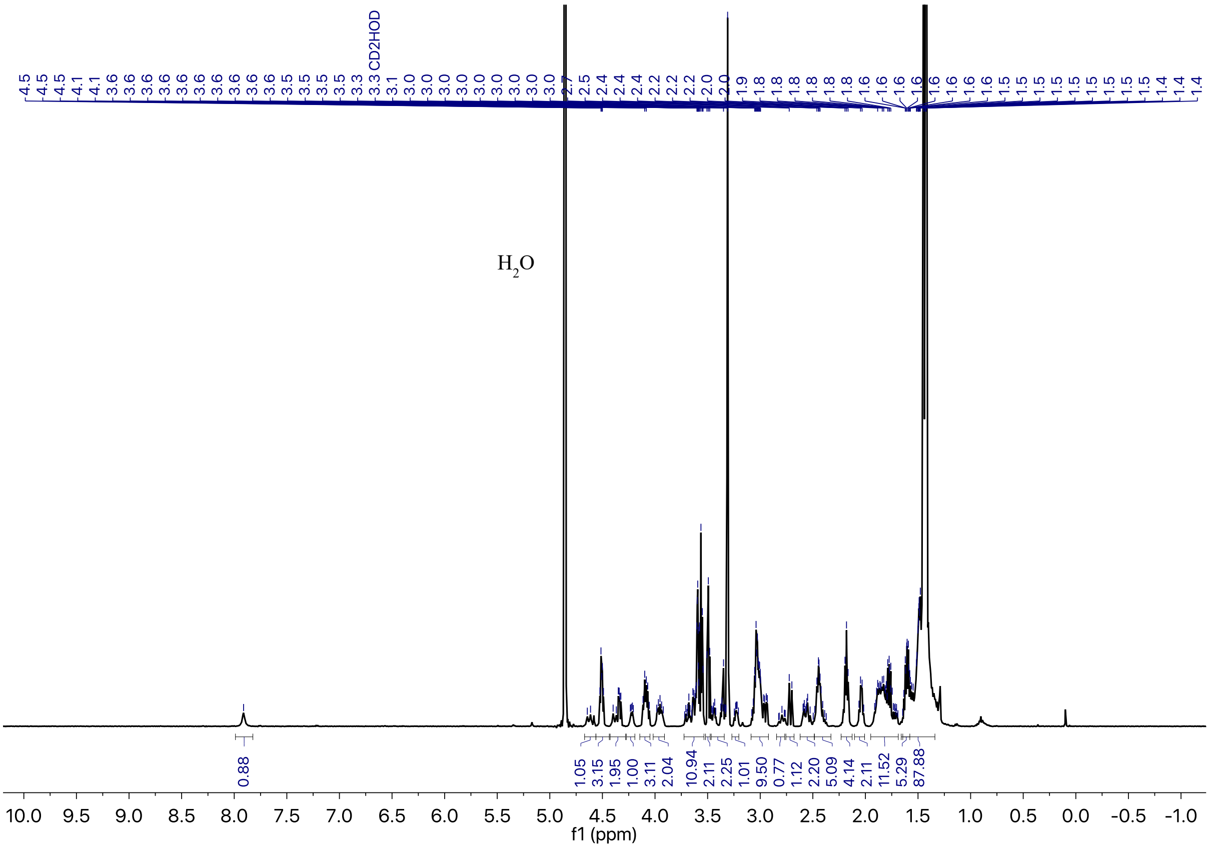

Fig. S100 ^{1}H NMR spectrum (500 MHz) of **117** in CD_3OD.

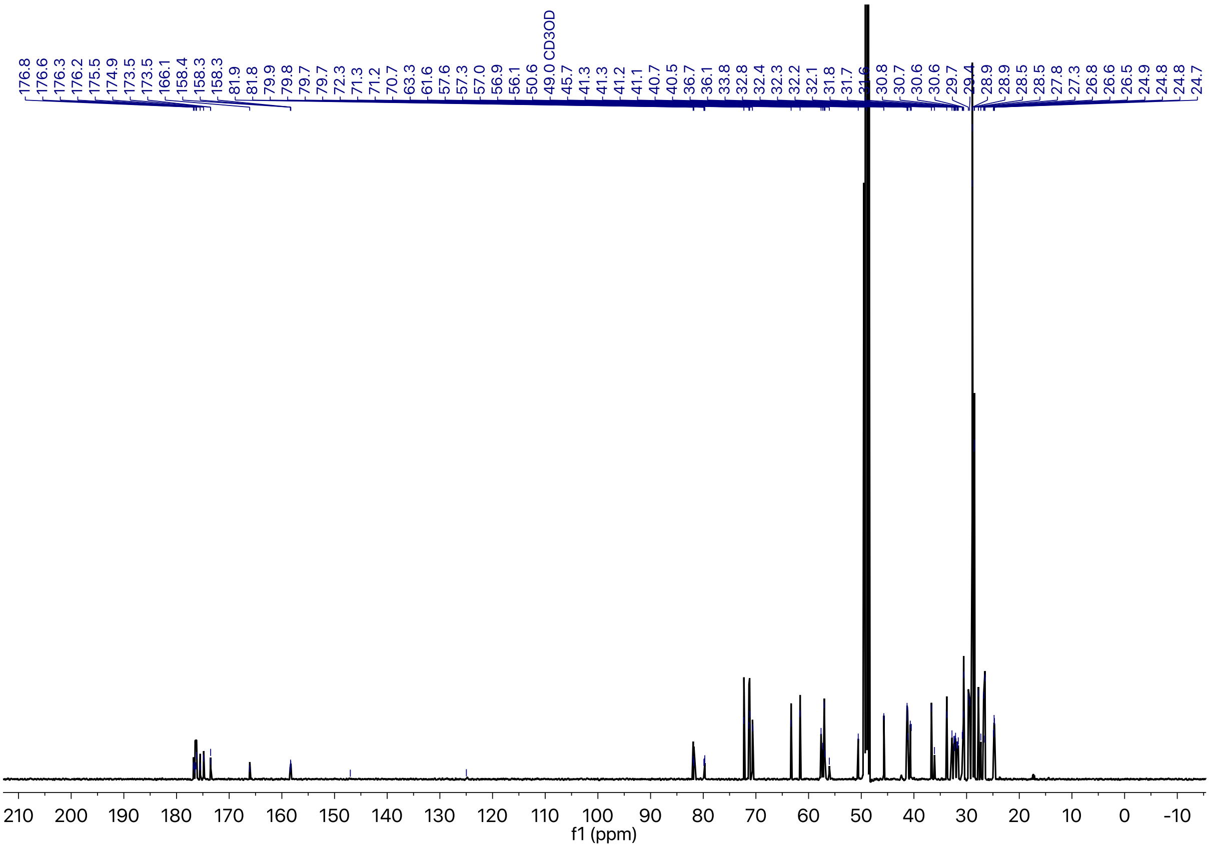

Fig. S101 ^{13}C NMR spectrum (126 MHz) of **117** in CD_3OD.

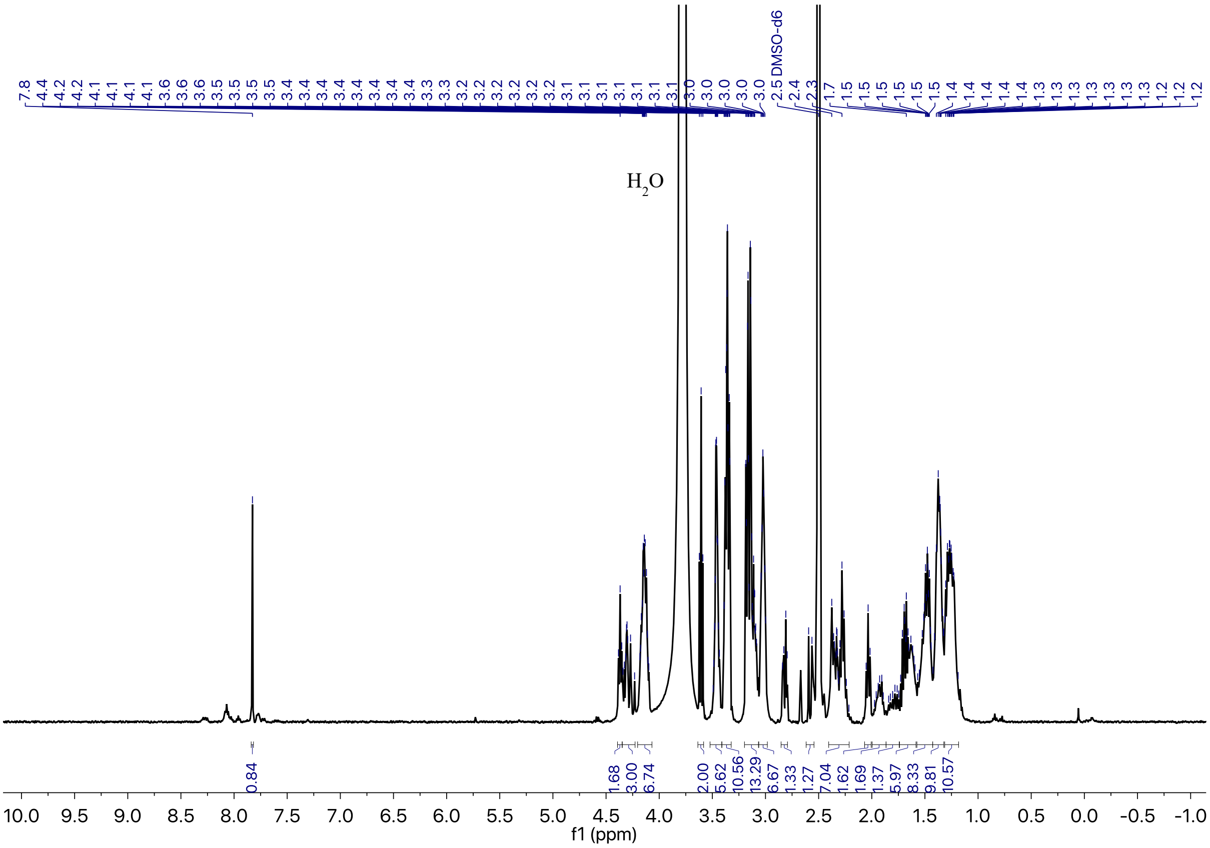

Fig. S102 ^{1}H NMR spectrum (500 MHz) of **11** in DMSO-d_6/D_2O 60:1.

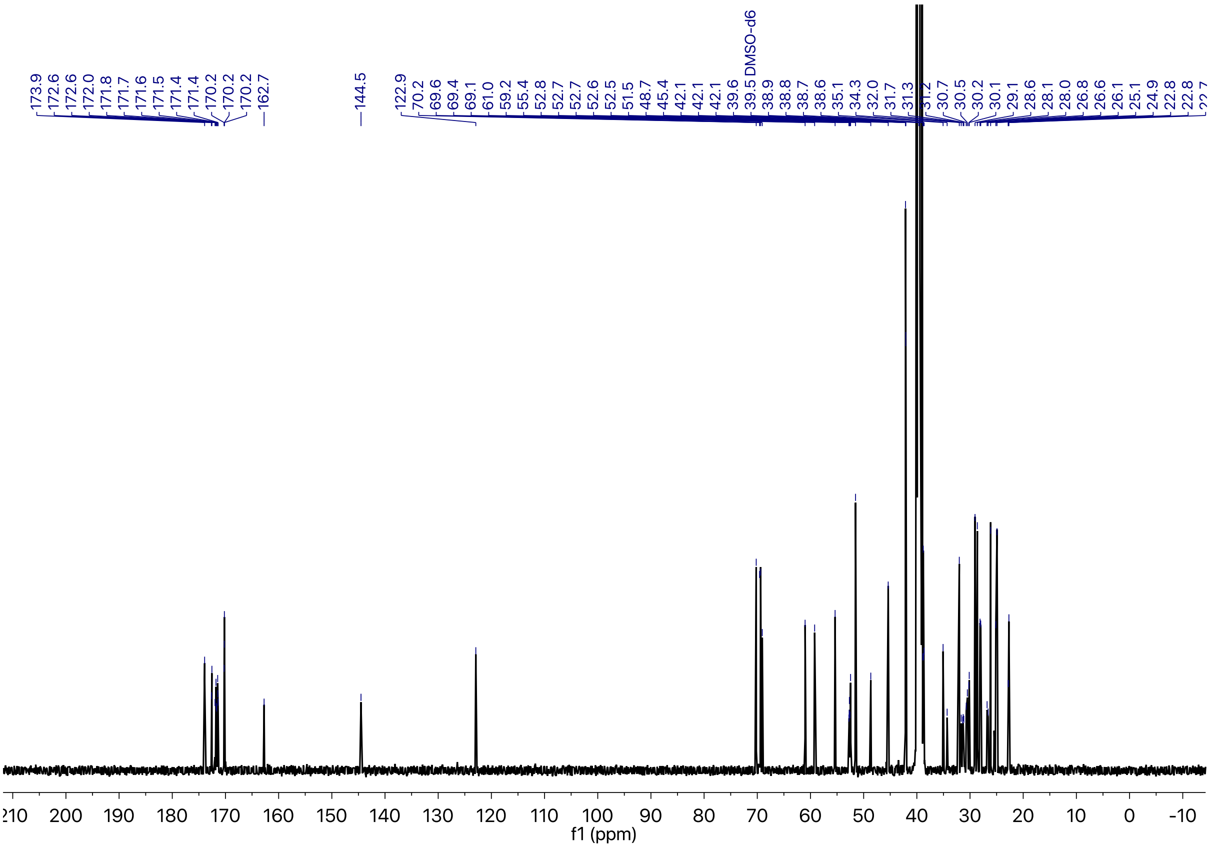

Fig. S103 ^{13}C NMR spectrum (126 MHz) of **11** in DMSO-d_6.

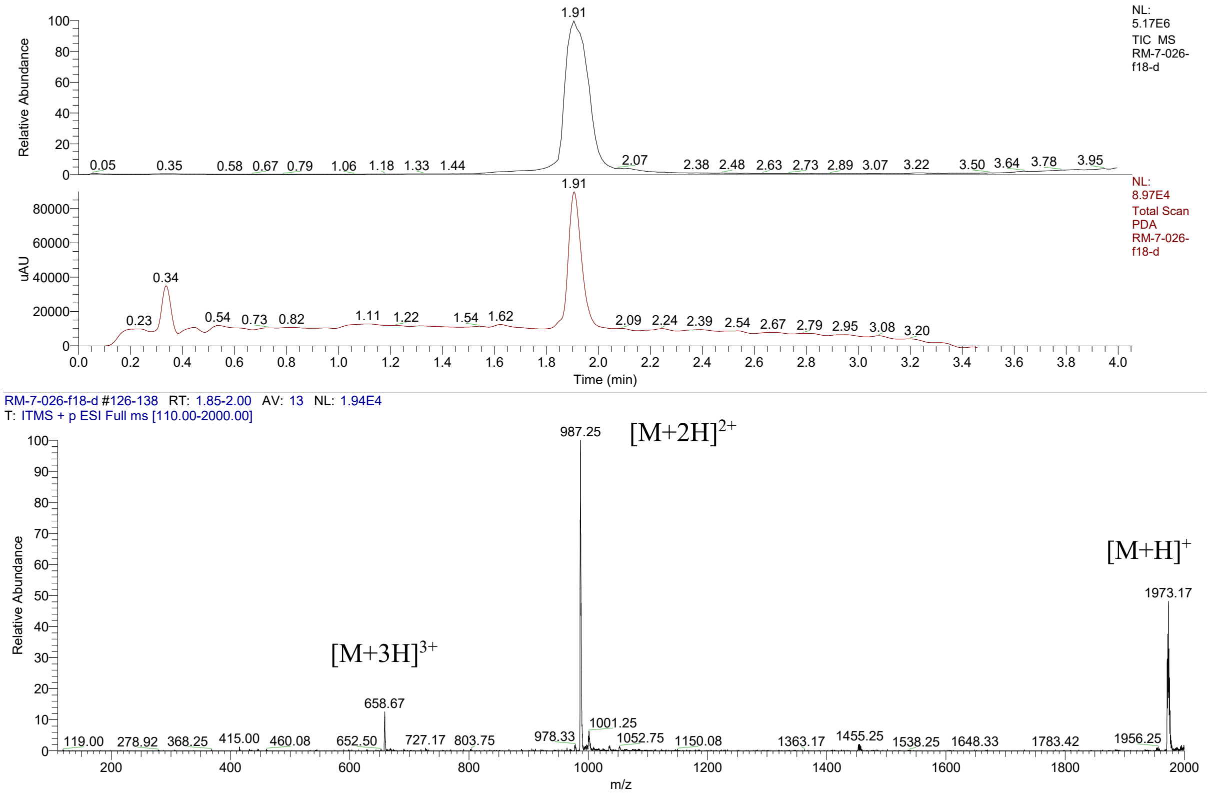

Fig. S104 HPLC-MS profile of **11**.

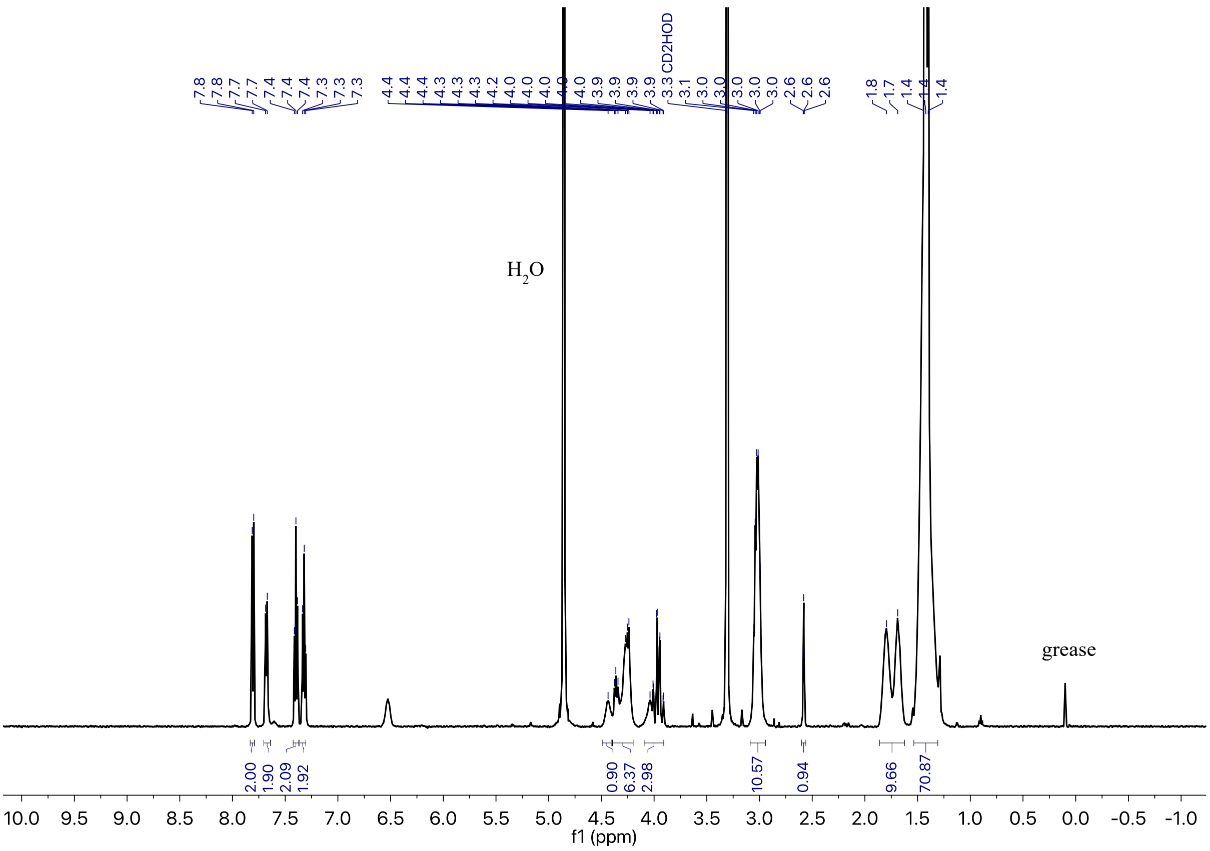

Fig. S105 ^{1}H NMR spectrum (500 MHz) of **119** in CD_3OD.

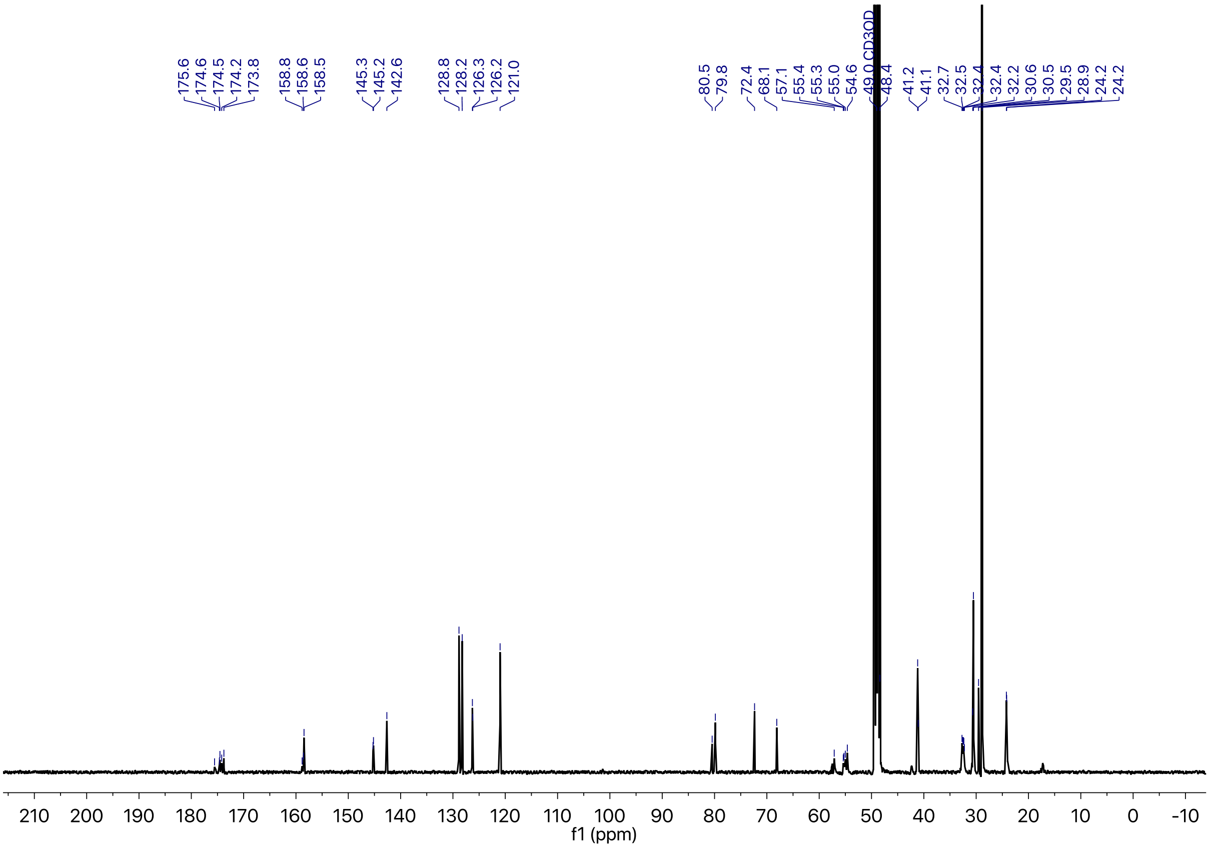

Fig. S106 ^{13}C NMR spectrum (126 MHz) of **119** in CD_3OD.

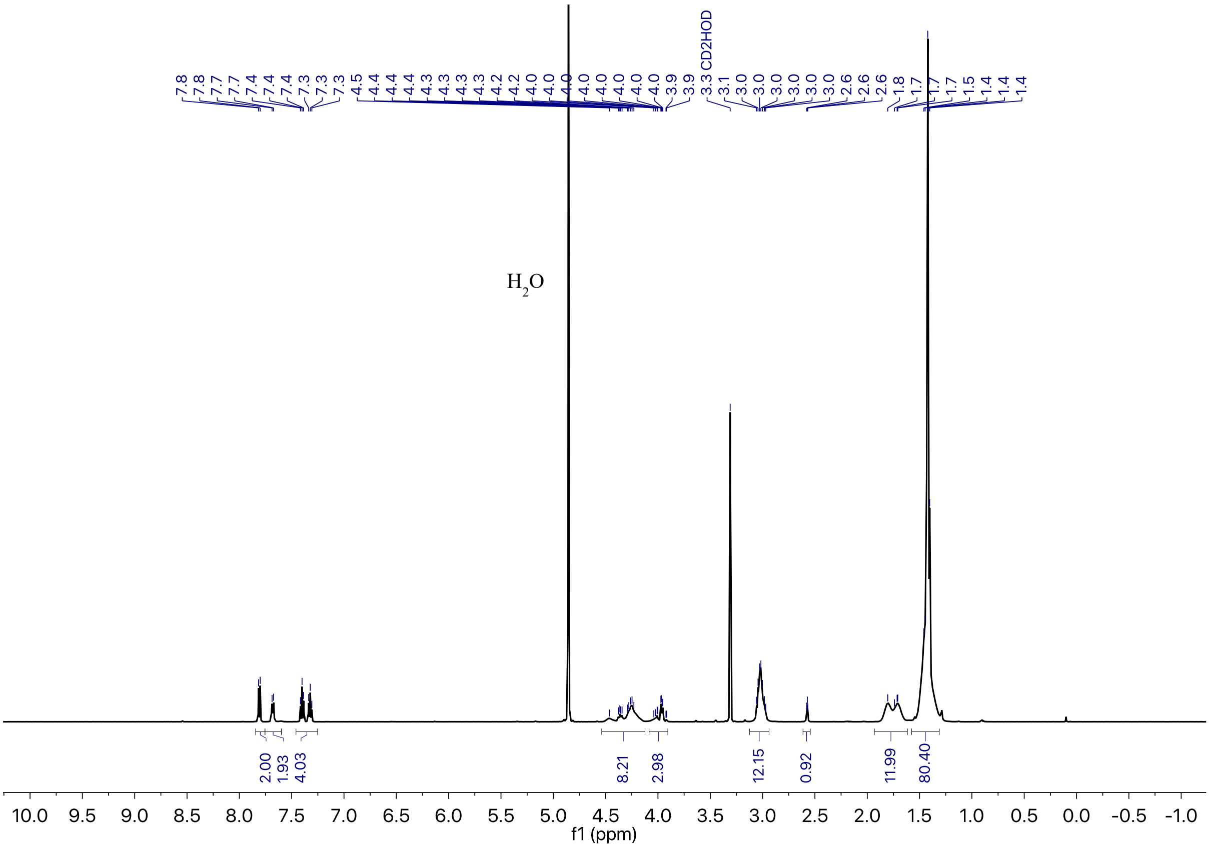

Fig. S107 ^{1}H NMR spectrum (500 MHz) of **121** in CD_3OD.

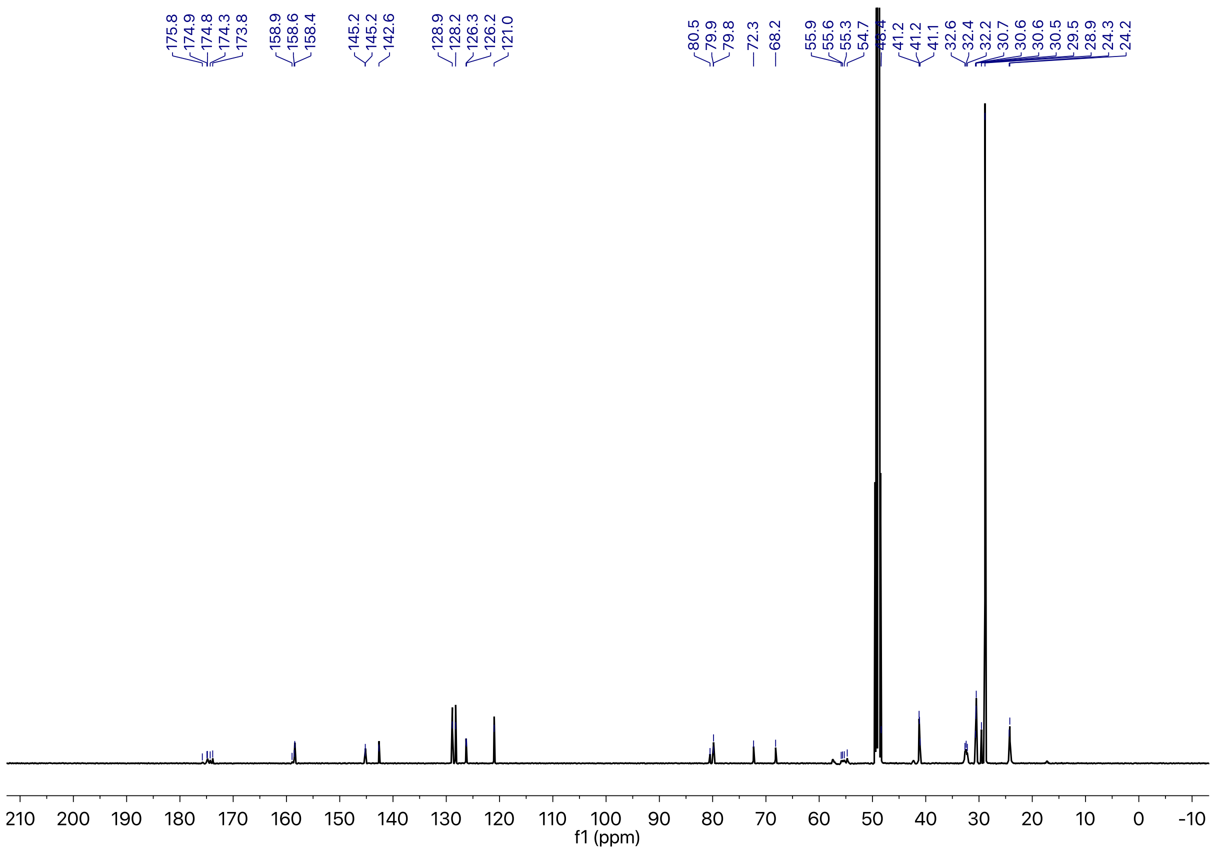

Fig. S108 ^{13}C NMR spectrum (126 MHz) of **121** in CD_3OD.

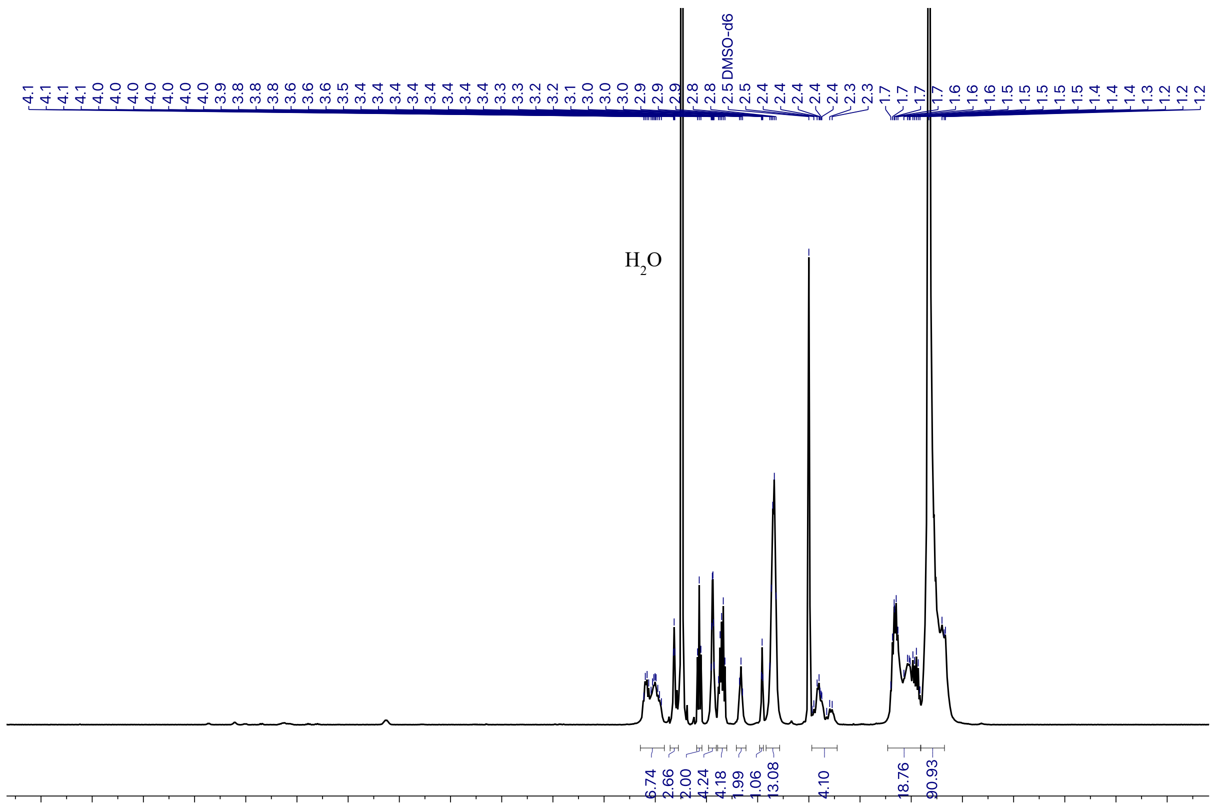

Fig. S109 ^{1}H NMR spectrum (400 MHz) of **123** in DMSO-d_6/D_2O 60:1.

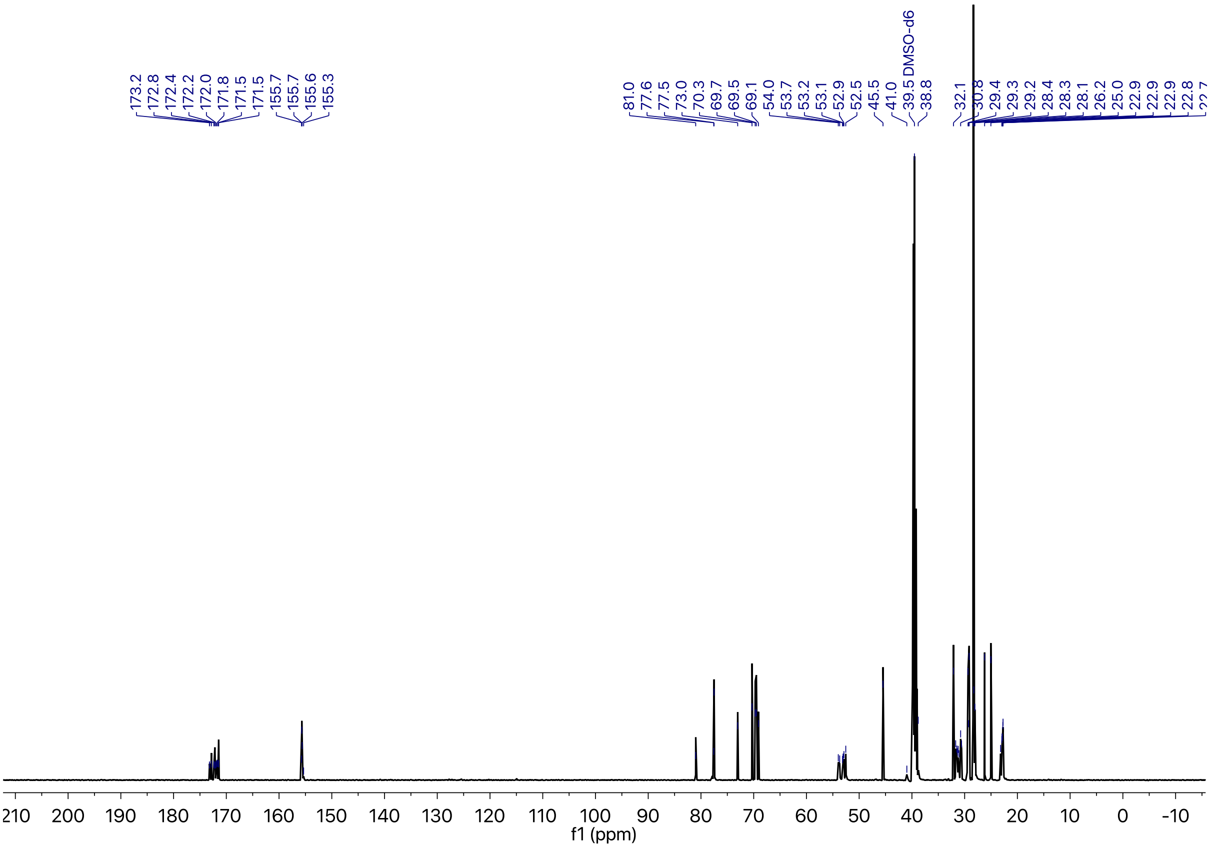

Fig. S110 ^{13}C NMR spectrum (126 MHz) of **123** in DMSO-d_6.

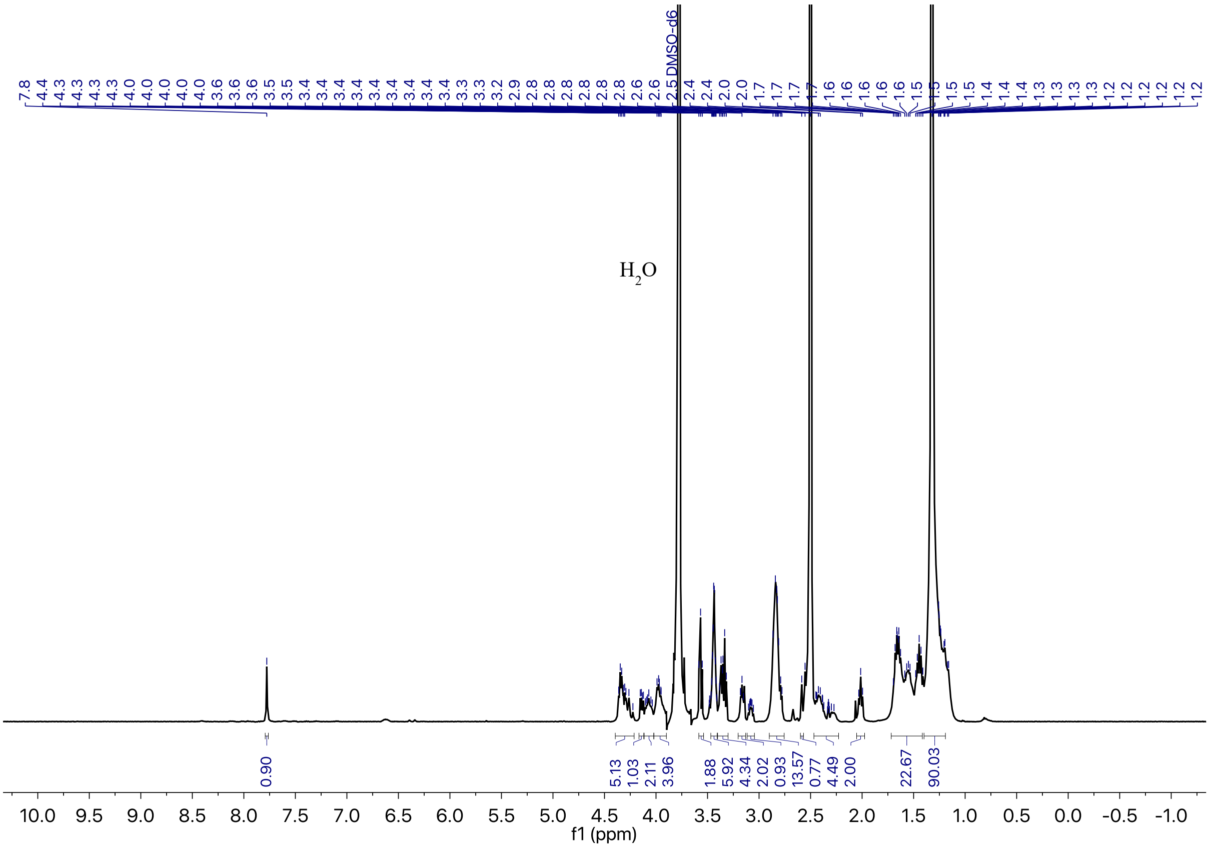

Fig. S111 ^{1}H NMR spectrum (400 MHz) of **124** in DMSO-d_6/D_2O 60:1.

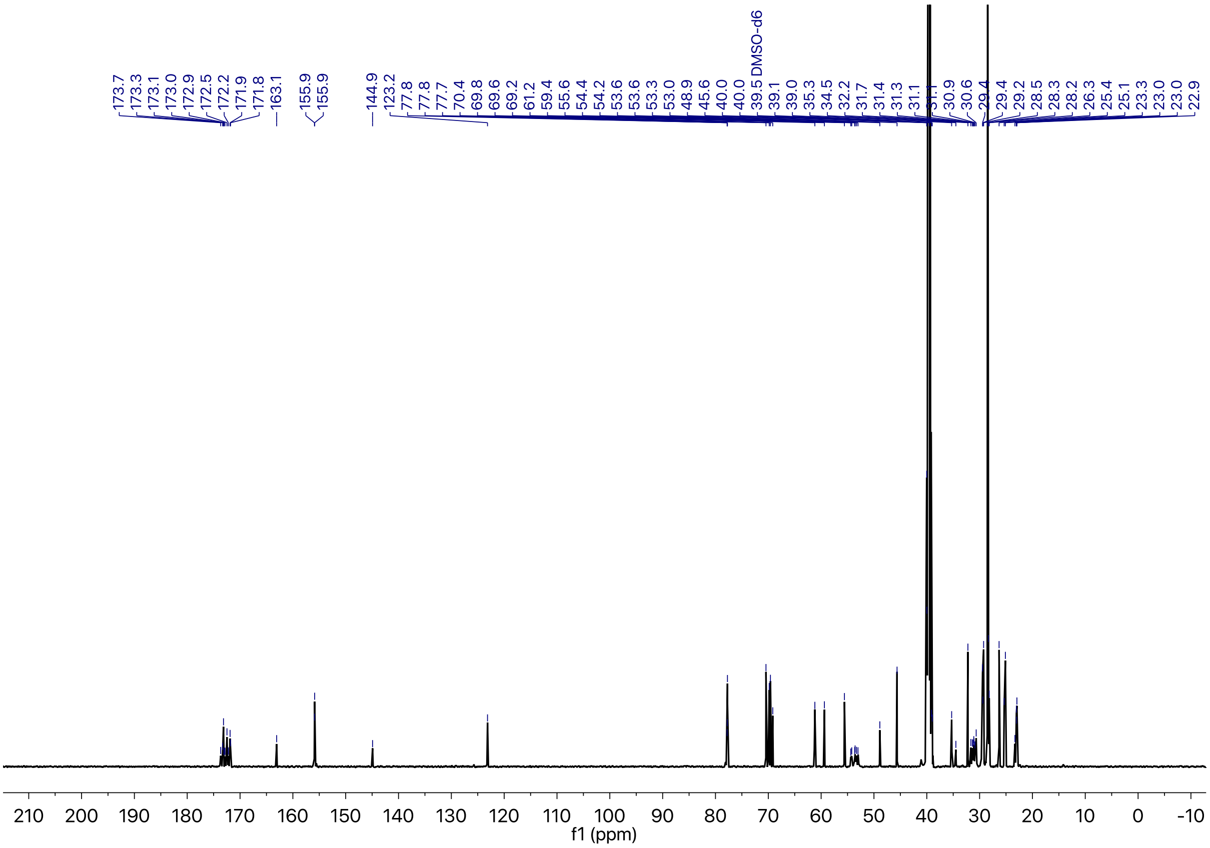

Fig. S112 ^{13}C NMR spectrum (126 MHz) of **124** in DMSO-d_6.

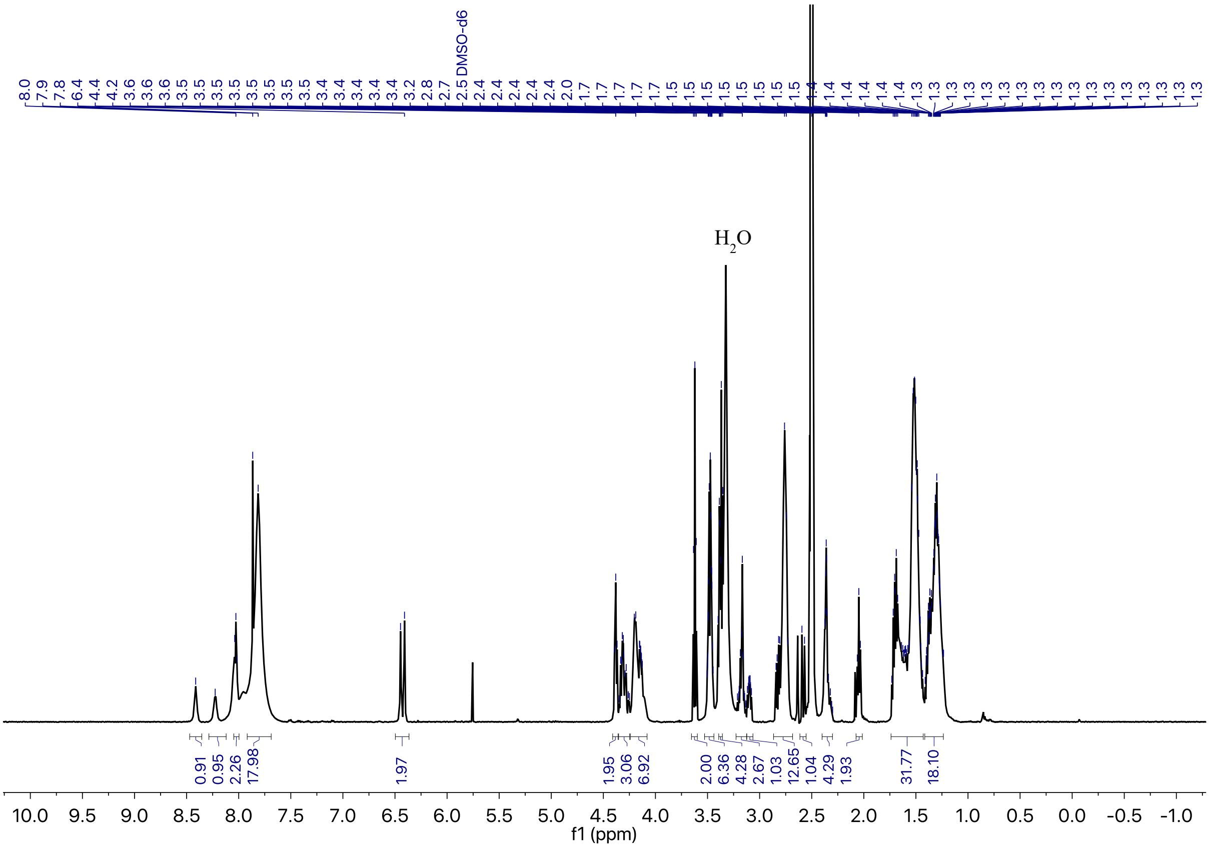

Fig. S113 ^{1}H NMR spectrum (500 MHz) of **14** in DMSO-d_6.

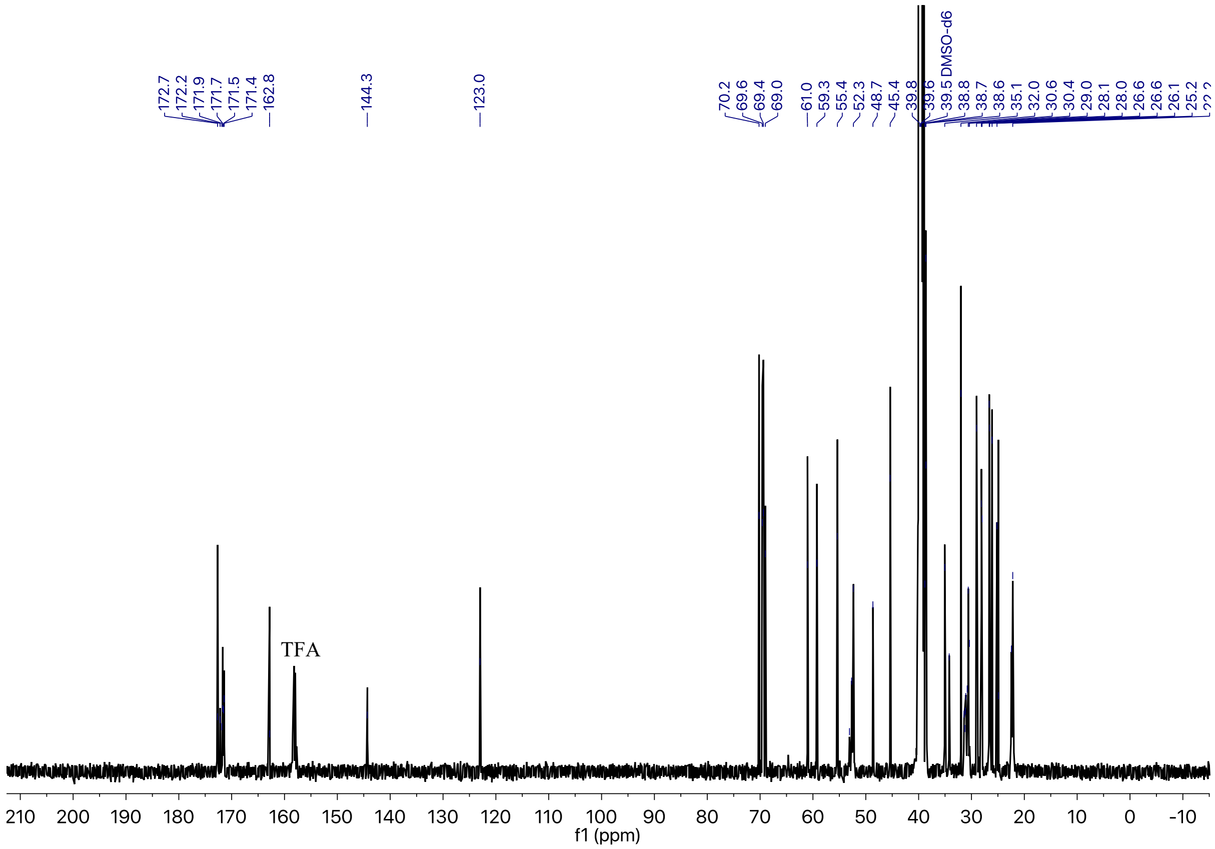

Fig. S114 ^{13}C NMR spectrum (126 MHz) of **14** in DMSO-d_6.

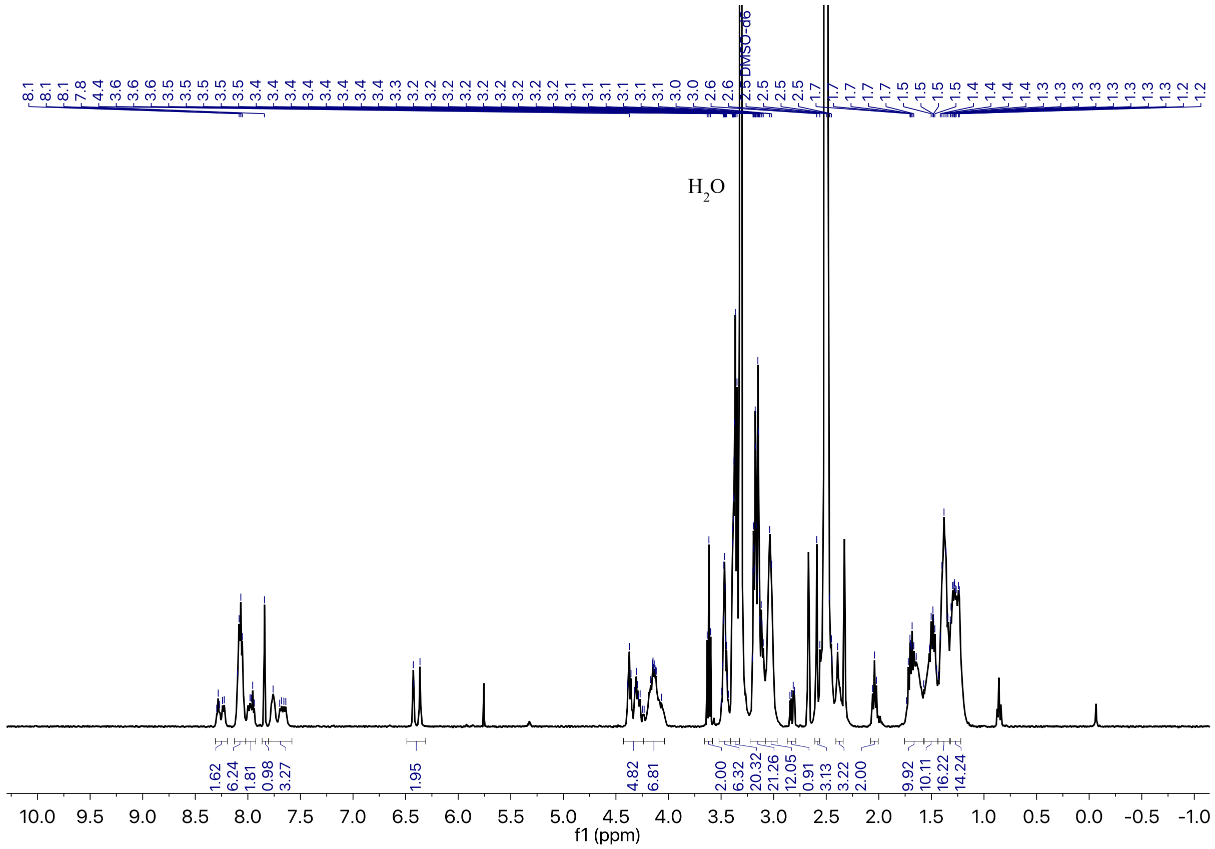

Fig. S115 ^{1}H NMR spectrum (500 MHz) of **12** in DMSO-d_6.

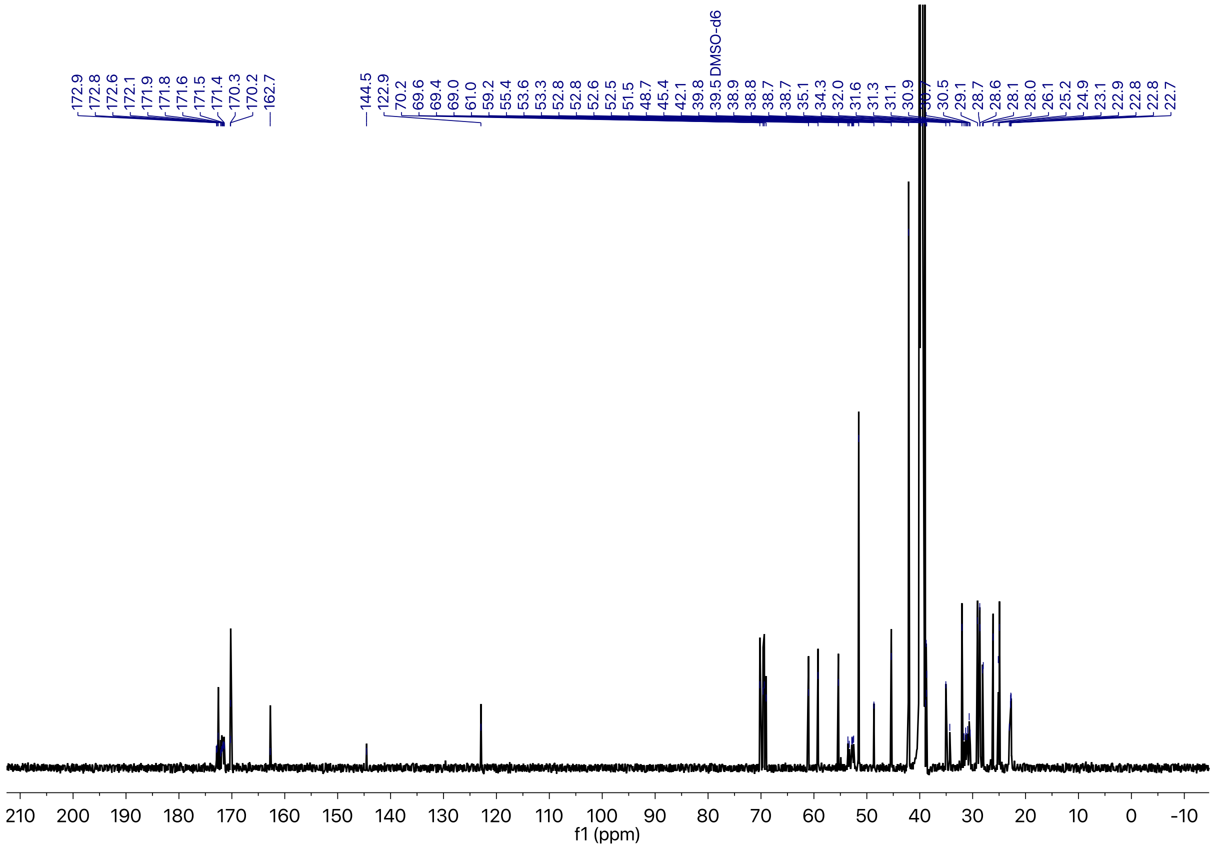

Fig. S116 ^{13}C NMR spectrum (126 MHz) of **12** in DMSO-d_6.

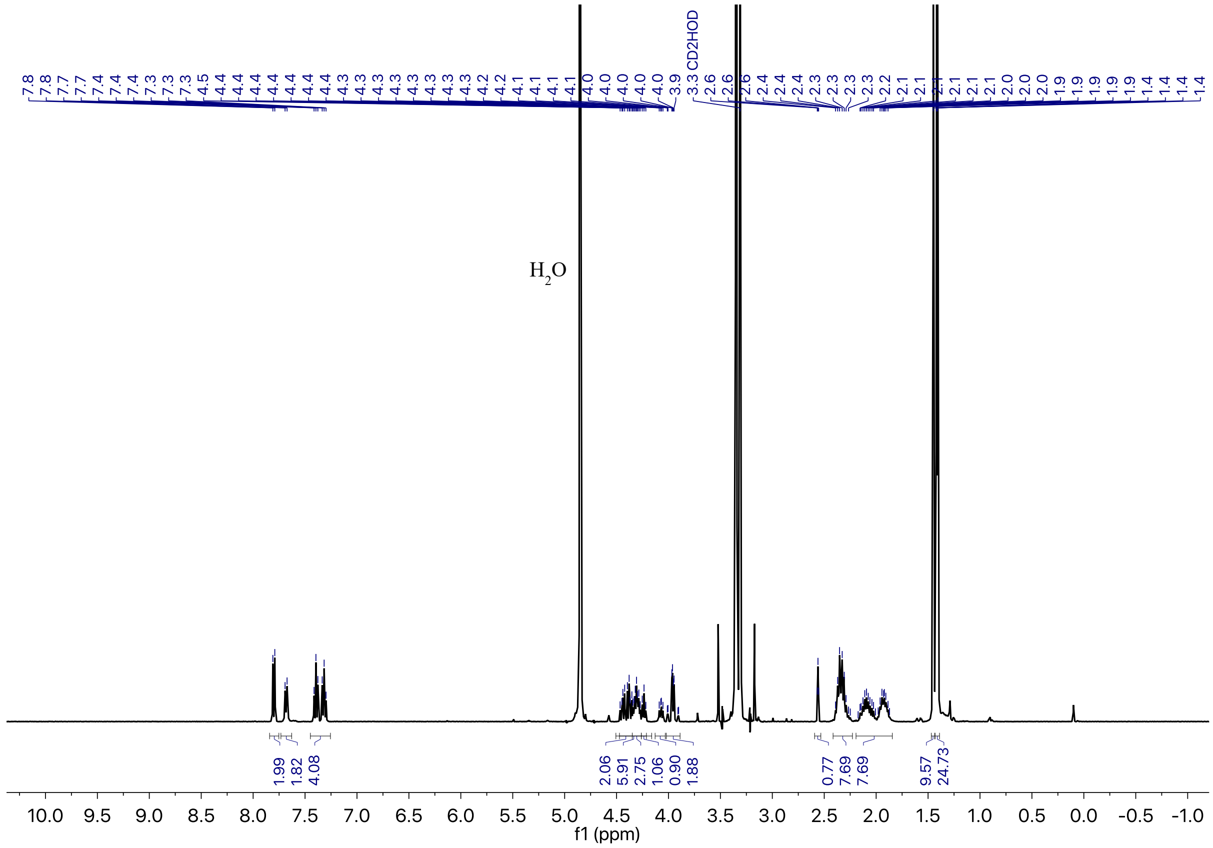

Fig. S117 ^{1}H NMR spectrum (400 MHz) of **125** in CD_3OD.

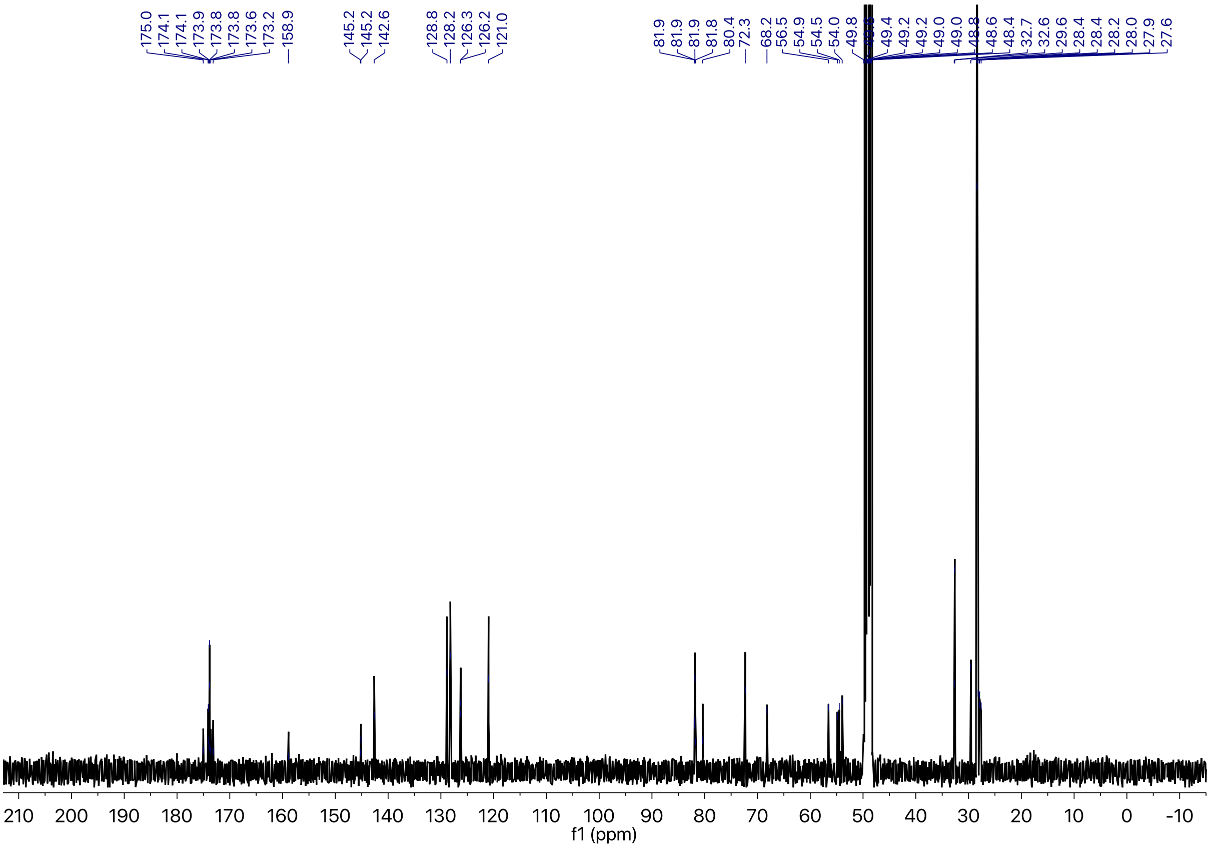

Fig. S118 ^{13}C NMR spectrum (101 MHz) of **125** in CD_3OD.

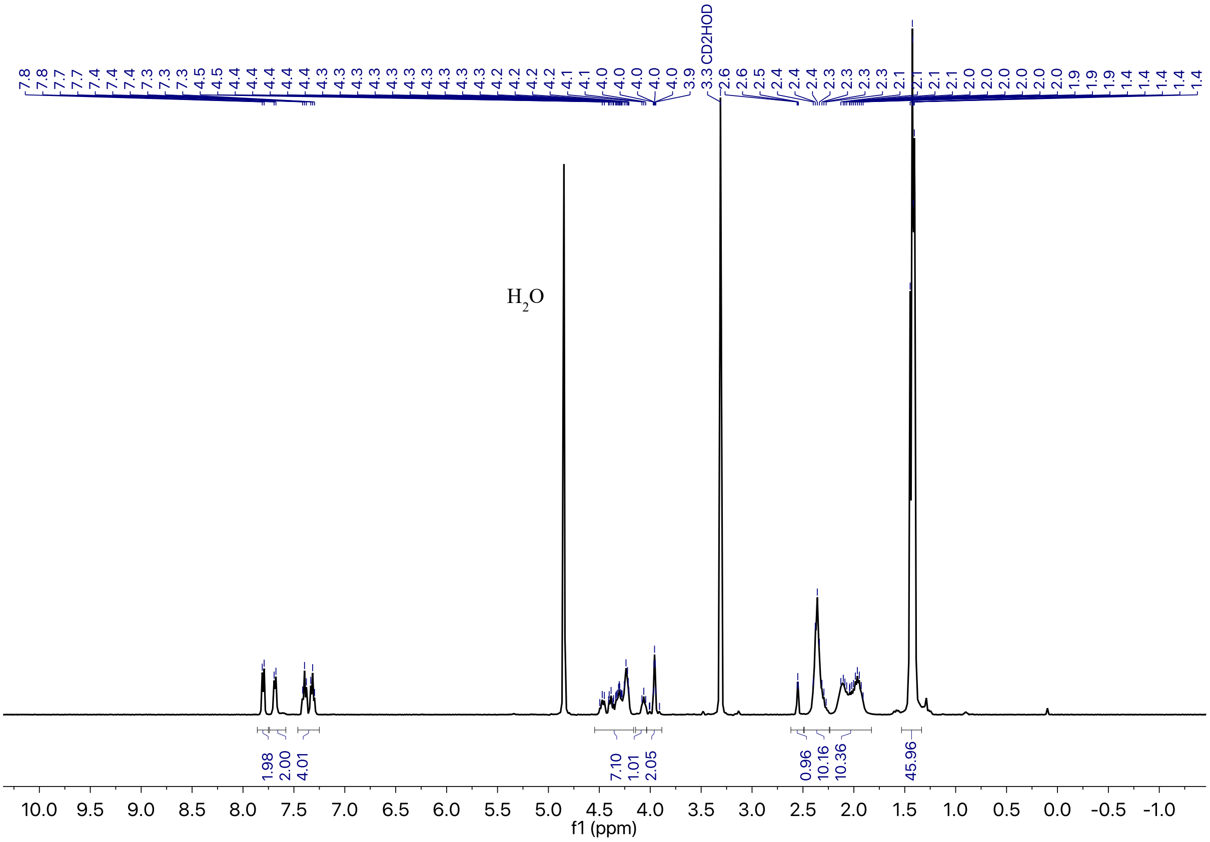

Fig. S119 ^{1}H NMR spectrum (400 MHz) of **127** in CD_3OD.

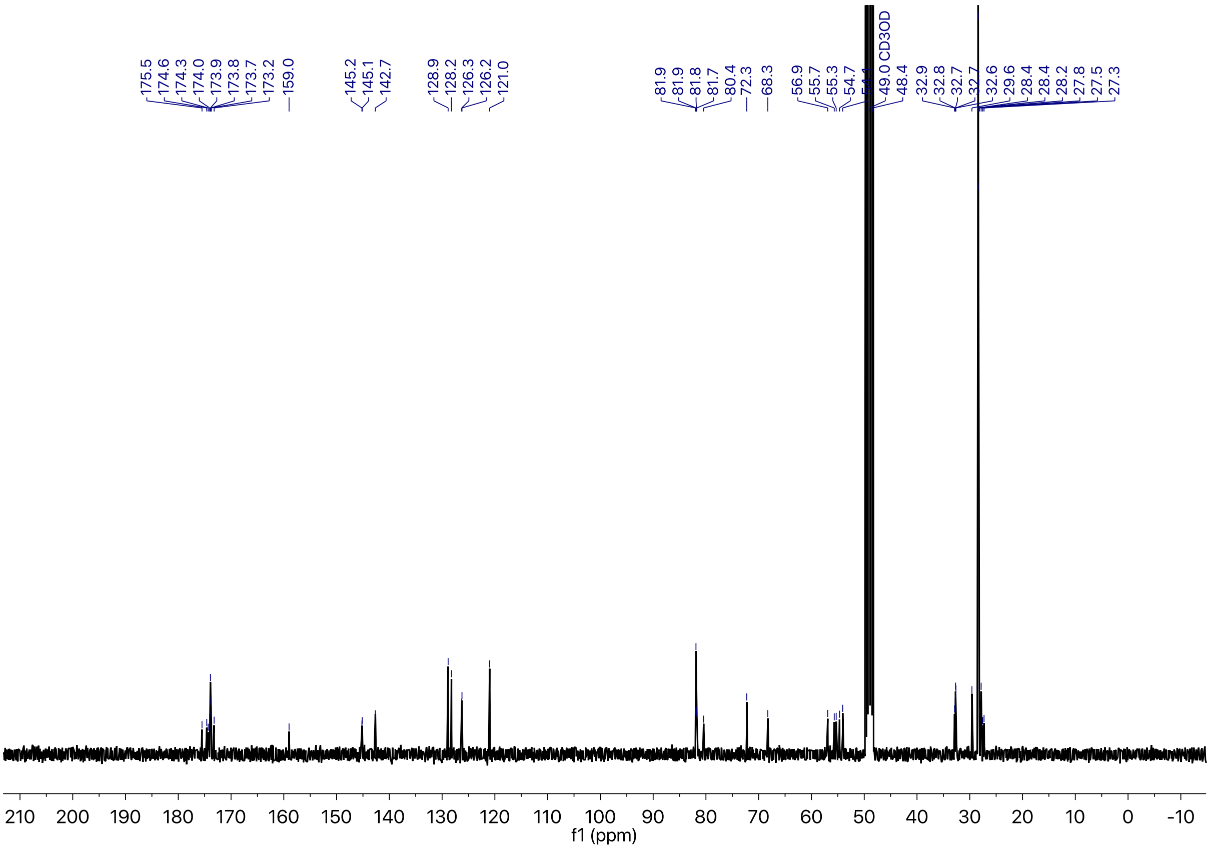

Fig. S120 ^{13}C NMR spectrum (101 MHz) of **127** in CD_3OD.

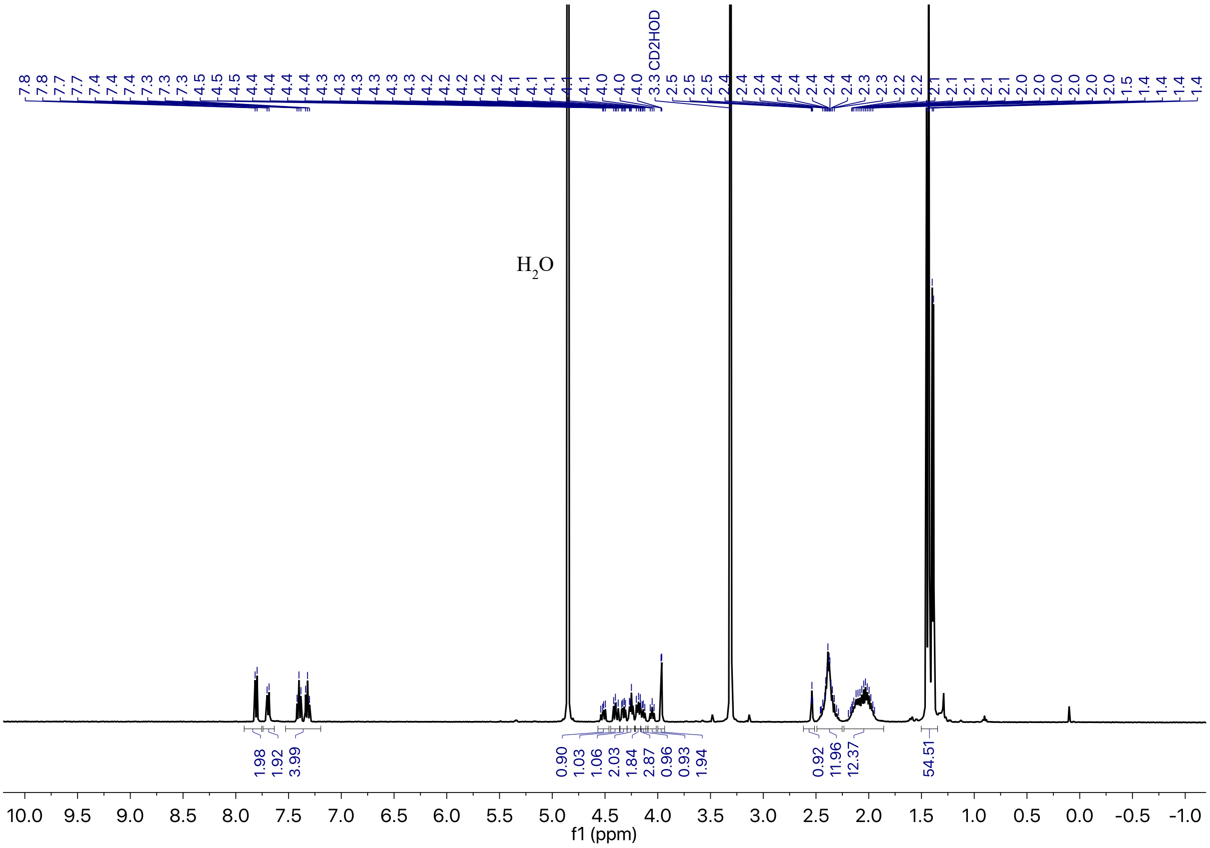

Fig. S121 ^{1}H NMR spectrum (400 MHz) of **129** in CD_3OD.

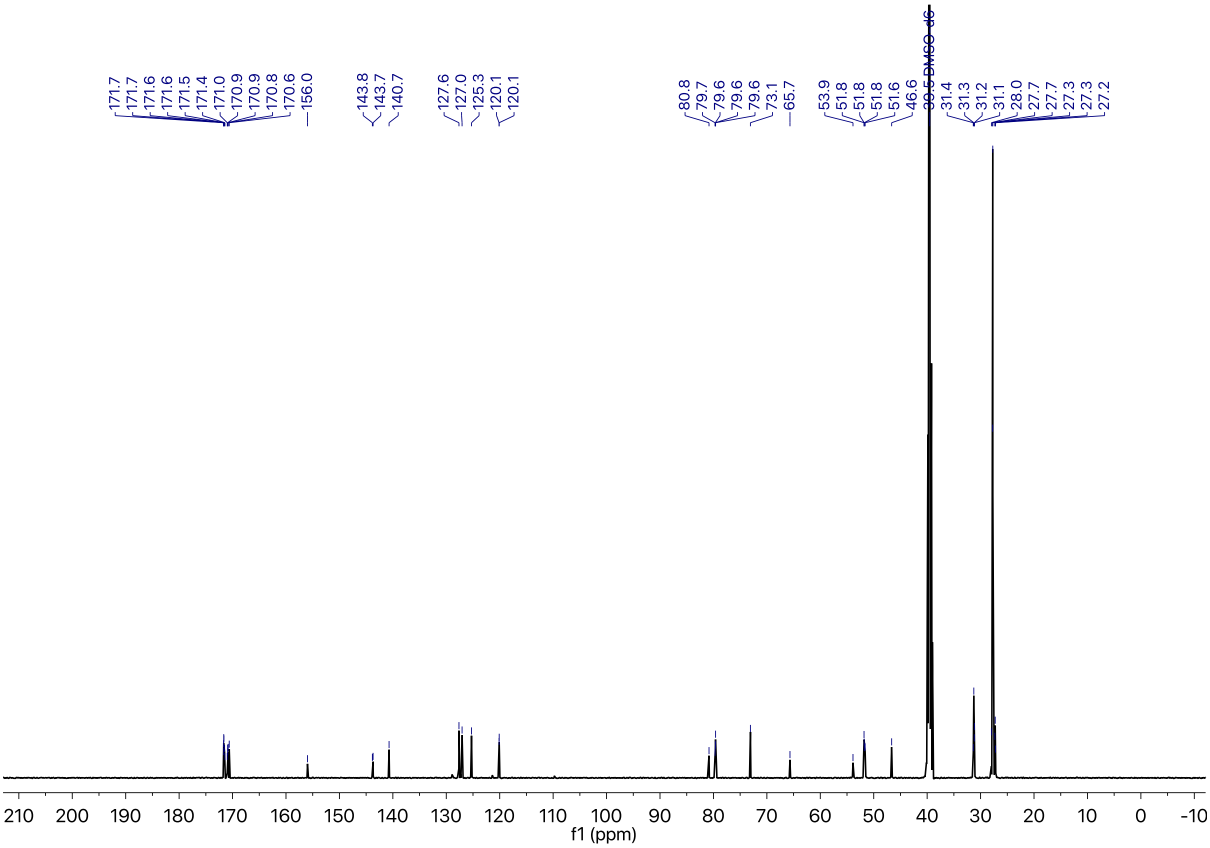

Fig. S122 ^{13}C NMR spectrum (126 MHz) of **129** in DMSO-d_6.

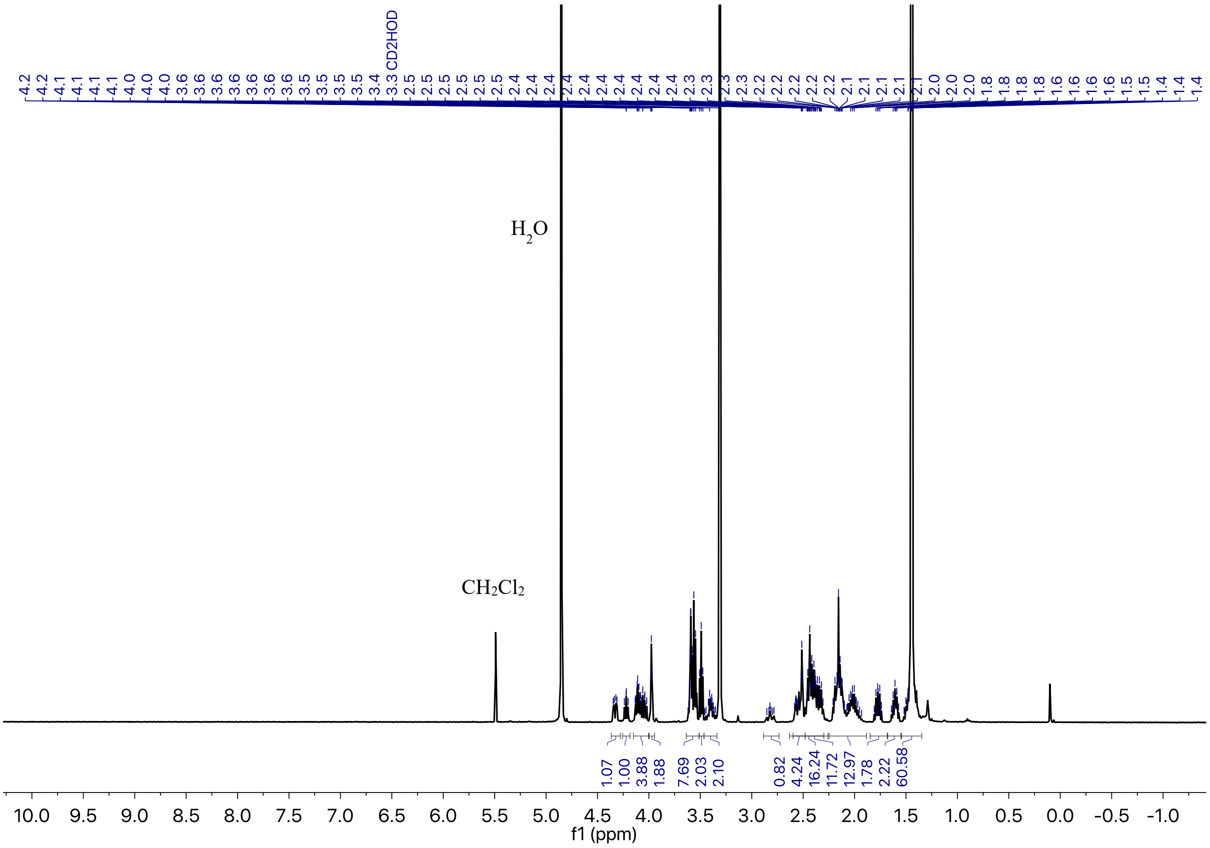

Fig. S123 ^{1}H NMR spectrum (400 MHz) of **131** in CD_3OD.

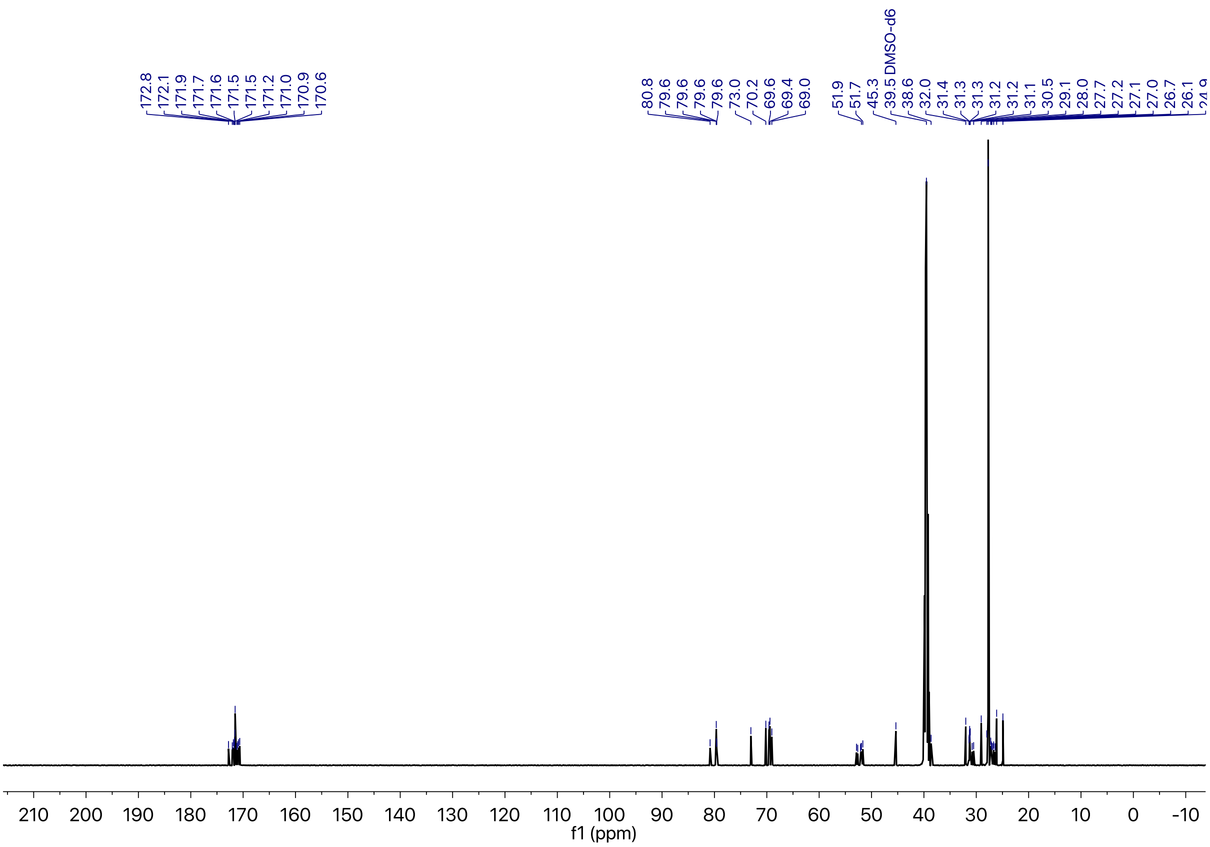

Fig. S124 ^{13}C NMR spectrum (126 MHz) of **131** in DMSO-d_6.

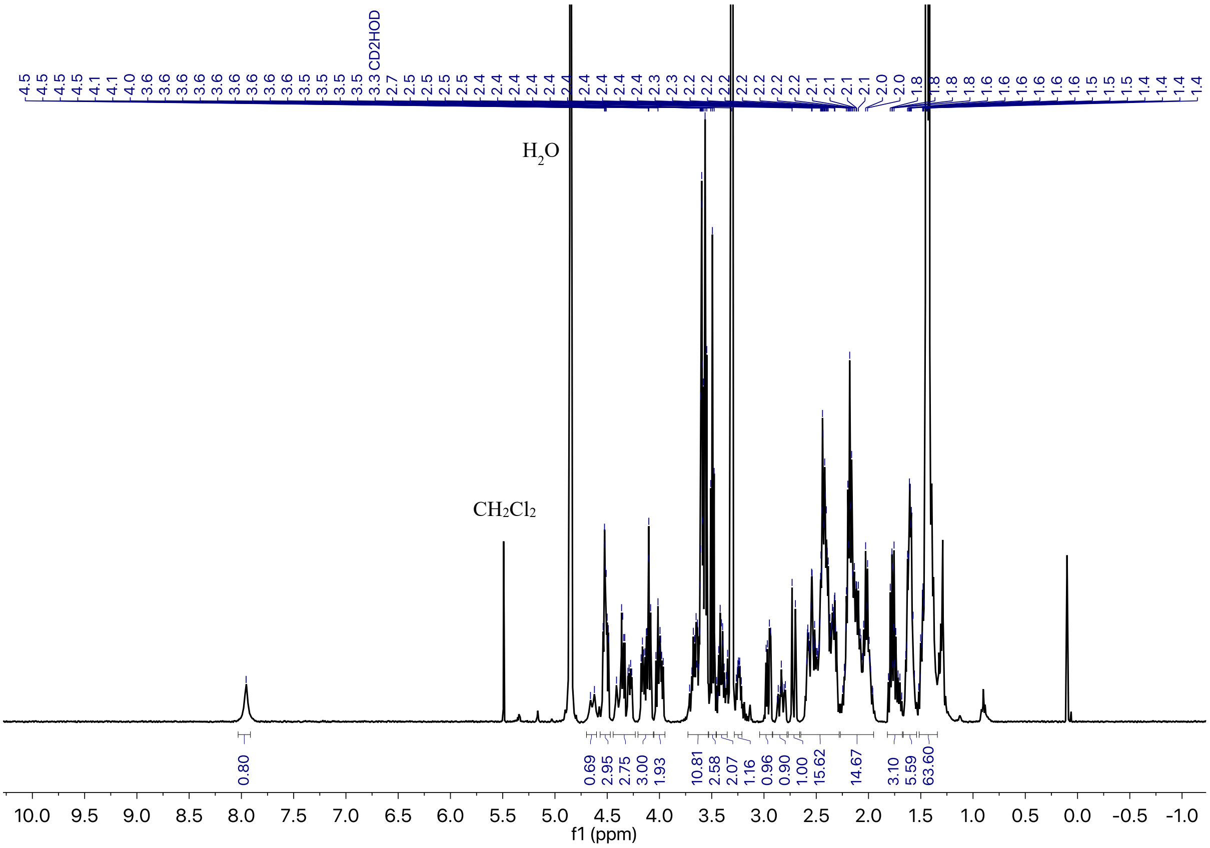

Fig. S125 ^{1}H NMR spectrum (400 MHz) of **132** in CD_3OD.

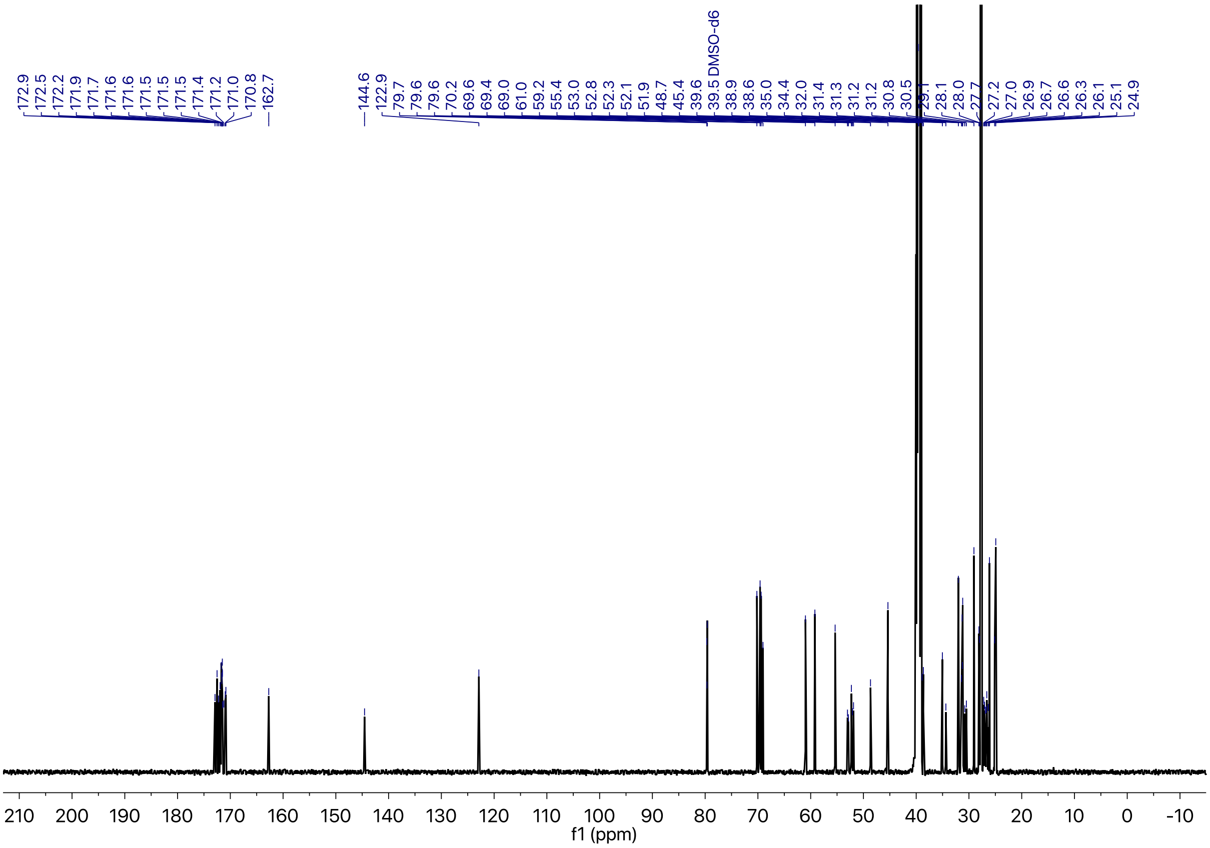

Fig. S126 ^{13}C NMR spectrum (126 MHz) of **132** in DMSO-d_6.

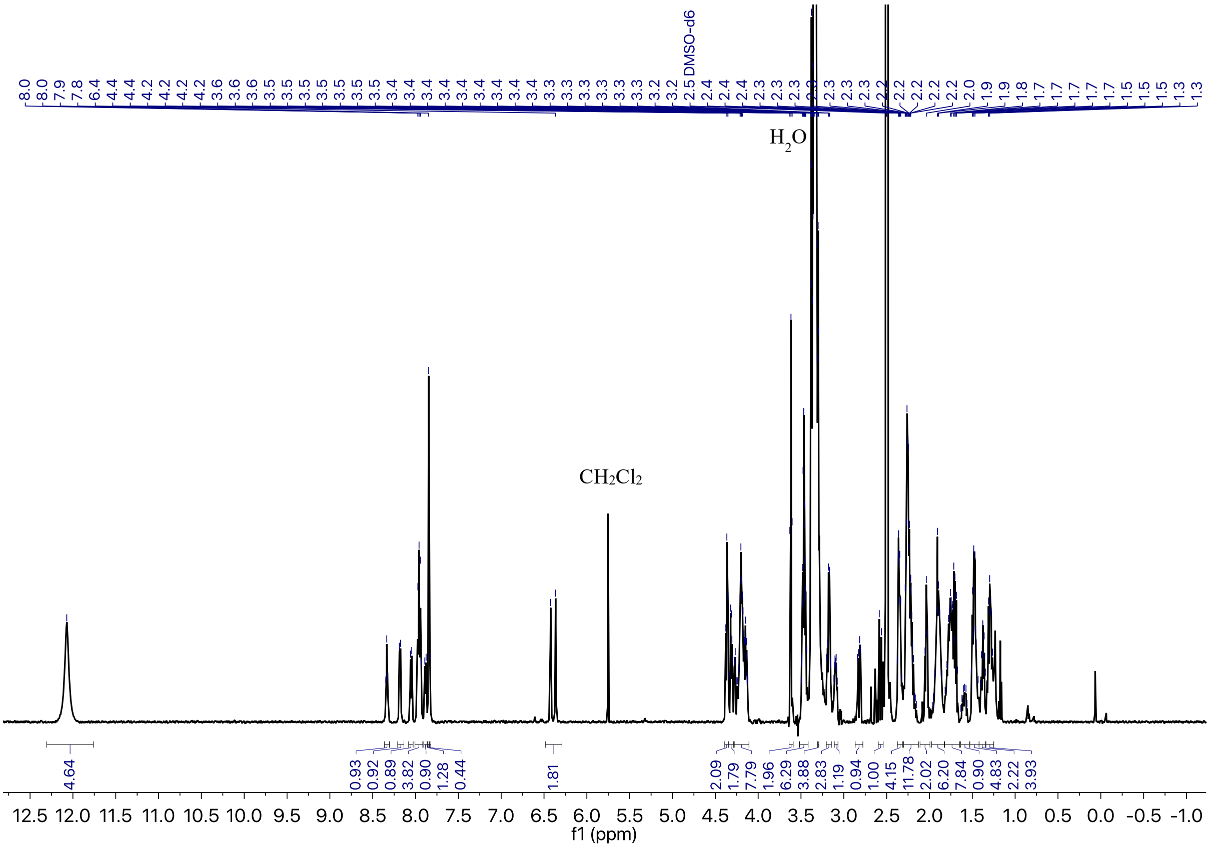

Fig. S127 ^{1}H NMR spectrum (500 MHz) of **13** in DMSO-d_6.

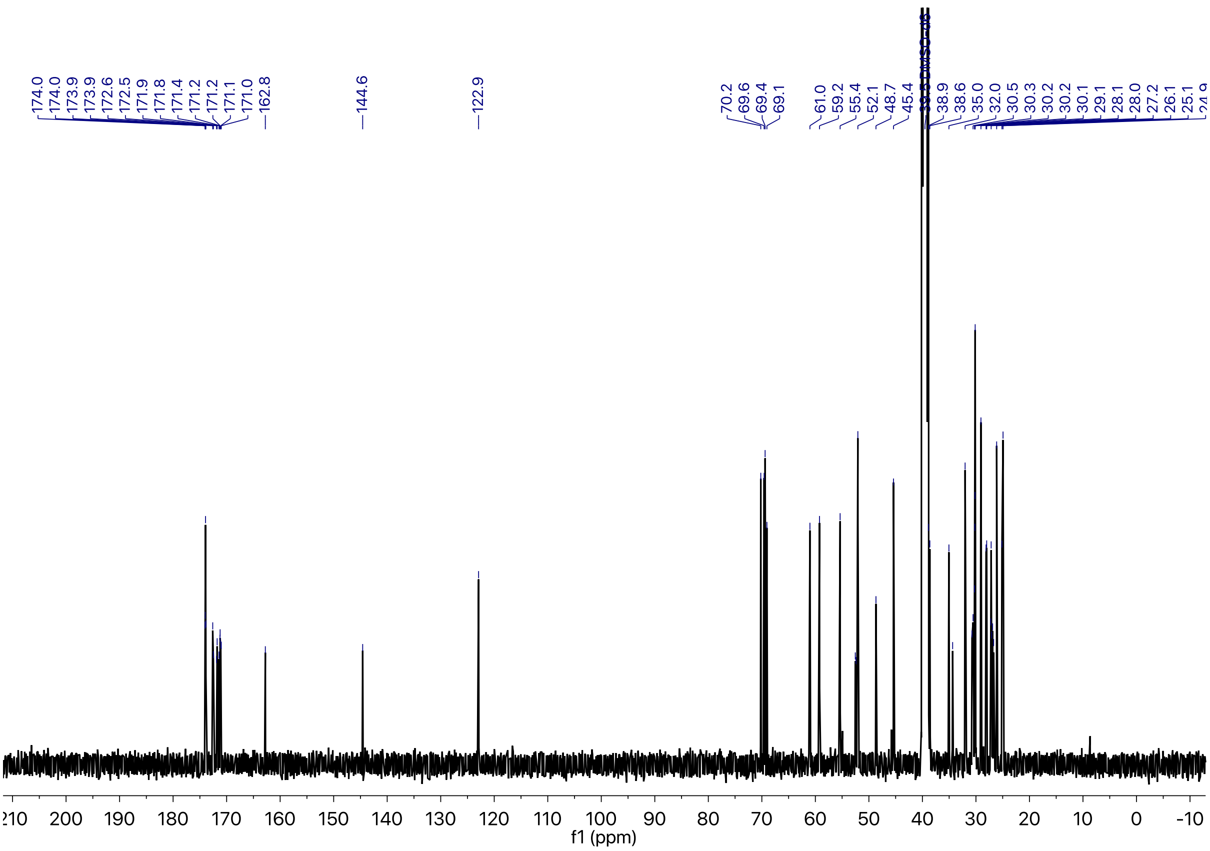

Fig. S128 ^{13}C NMR spectrum (126 MHz) of **13** in DMSO-d_6.
